# Constructing material network representations for intelligent amorphous alloy design

**DOI:** 10.1093/nsr/nwaf398

**Published:** 2025-09-19

**Authors:** Shiyun Zhang, Jiachuan Tian, Songling Liu, Huimin Zhang, Haiyang Bai, Yuan-Chao Hu, Wei-Hua Wang

**Affiliations:** Songshan Lake Materials Laboratory, Dongguan 523808, China; Lawrence Berkeley National Laboratory, Berkeley CA 94720, USA; Songshan Lake Materials Laboratory, Dongguan 523808, China; Songshan Lake Materials Laboratory, Dongguan 523808, China; Songshan Lake Materials Laboratory, Dongguan 523808, China; Institute of Physics, Chinese Academy of Sciences, Beijing 100190, China; Songshan Lake Materials Laboratory, Dongguan 523808, China; Songshan Lake Materials Laboratory, Dongguan 523808, China; Institute of Physics, Chinese Academy of Sciences, Beijing 100190, China

**Keywords:** amorphous alloy, material design, material network, artificial intelligence

## Abstract

Designing high-performance amorphous alloys for various applications is demanding, as the process heavily depends on empirical laws and unlimited attempts. The high-cost and low-efficiency nature of traditional strategies prevents effective sampling in the enormous material space. Here, we propose material networks to accelerate the discovery of binary and ternary amorphous alloys. The network topologies reveal hidden material candidates that were obscured by traditional tabular data representations. By scrutinizing the amorphous alloys synthesized in different years, we construct dynamical material networks to track the history of alloy discovery. We find that some innovative materials designed in the past were encoded in the networks, demonstrating their predictive power in guiding new alloy design. These material networks show physical similarities with several real-world networks in our everyday lives. Our findings pave a new path toward intelligent material design, especially for complex alloys.

## Teaser

Network representations of amorphous alloy experimental data reveal unprecedented material design insights from their dynamics and topologies, and will integrate cutting-edge deep learning with AI to advance future complex alloy development.

## INTRODUCTION

Advancing fundamental disciplines, such as materials science, physics and chemistry, to name a few, heavily depends on the discovery of new materials [1,2]. The successful fabrication of material in experiments quite often signifies the opening of an unknown world. It also usually serves as a carrier for the acquisition of theoretical knowledge to catalyze next-generation optimization. Iterating this process presents the common evolution pathway for critical scientific breakthroughs in natural science.

While the periodic table contains only a limited number of elements, the vast array of possible combinations among these elements forms the foundation of Earth, supporting the continuous existence of diverse biological groups. It is fair to say that the material space is infinite [2,3]. Ever since ancient times, materials were developed mainly by trial-and-error experiments, with empirical laws summarized from mostly the failure experiences. In modern times, there is undoubtedly high demand for materials with superior performances. Unfortunately, the traditional strategy fails to satisfy this requirement.

To circumvent this grand challenge, various methodologies have been proposed in the past. For example, high-throughput sputtering experiments are capable of synthesizing a library of more than 1000 ternary alloys with composition gradients in just one experiment [4–6]. Meanwhile, motivated by the rapid increase in computational power, parallel computer simulations using either density functional theory or molecular dynamics offer significant theoretical support for experimental material design [7–9]. With the growing availability of scientific data, advanced machine learning techniques have rapidly advanced in the domain of materials prediction [2,8]. In particular, the advent of artificial intelligence (AI) in computer vision and natural language processing greatly fostered the virtual screening of candidate materials in the large latent space [10–12].

However, several key problems remain to accelerate materials discovery. The most critical one is the severe shortage of high-quality data. This is inherent in the complex and costly process of material synthesis. Moreover, only successful trials have been reported in the literature.

One prominent example is amorphous alloys, which are mainly made of transition metals, but lack long-range translational periodicity. The infinite possibilities of atomic packing in space at the microscopic scale make this problem even more challenging to solve. Since its first discovery in 1960 [13], several decades have passed, yet glassiness has been successfully observed in only ${\sim }670$ systems—ranging from binary to multi-component alloys—out of ${\sim }7000$ compositions [14]. The largest sample size is still limited to several centimeters, but the general size is around millimeters and even smaller [15]. These limitations significantly constrain the practical use of amorphous alloys across various fields, despite their metal-like mechanics and glass-like functionalities. Ongoing efforts to address this problem involve high-throughput experiments [4,5], supervised machine learning applied to experimental data [14,16–25] or a combination of both approaches [6,26]. However, the candidate materials are far from fulfilling the demands.

To speed up this process, the emerging data-driven techniques are promising, with possibly the assistance of cutting-edge AI technologies [2,10]. Since amorphous alloys are the result of extensive research efforts, a vast amount of underlying physics is embedded within the relatively small available dataset [27]. How to design effective material representation is a crucial step in data mining, whether for human researchers or machine learning algorithms. To learn from these data, previous studies used the physical properties of alloys and their elements to create learnable features for machines in a supervised manner [14,16–25]. A tabular dataset is structured by using alloy compositions as rows and their features as columns. These instances are treated independently during learning. While this supervised learning provides some insights into inventing new amorphous alloys, the predictive power is still limited. One of the crucial reasons is the plain representation in the tabular form. The hidden physical connections among alloys are neglected. The information loss seriously deteriorates the model predictability. Physics-inspired data mining will be mandatory to build excellent machine learning models. Thus, a more sophisticated data representation, especially for a small amount of data with lots of physics, is critical for successful data learning to assist the optimal design of amorphous alloys.

In this work, we propose an effective network representation for amorphous alloys. We focus on the binary and ternary alloys that are prone to form glass by rapid-quenching experiments. The datasets are compiled from decades of collaborative research reported in the literature [3,26,28,29]. We construct both virtual and realistic networks from these data. They provide valuable scientific senses to better understand the materials and the associated hidden physics. By thorough network mining, we identify the possible material space and the evolution pathway of amorphous alloy development. The imbalanced contributions from various elements are revealed. Furthermore, by analyzing the dynamical material networks built in different years, the history of alloy discovery is unveiled. We find that some innovative materials fabricated in the past were actually encoded in the material networks. This demonstrates the predictive power of these networks. The topology analysis provides unique perspectives to further optimize the material design procedure. In addition, from the network analysis, these networks show features belonging to the abnormal scale-free class, which exhibits preferential attachment in the network growth. We argue that these material networks intrinsically suffer from physical constraints from the available constituent elements in the periodic table, which distinguishes them from the general scale-free class [30]. We find similar features in several real-world networks in our everyday lives. This connects material networks to realistic ones, promoting interdisciplinary collaboration and opening new pathways for intelligent discovery of complex alloys.

## Results

### Material networks of amorphous alloys

We start our investigation by carefully collecting the available data in the literature (see the section entitled ‘Methods’). As the data are widely dispersed in various journals, we may not have all the data reported so far, but should have the vast majority. Comprehensive data cleanings, such as redundancy removal, composition correction and quality alignment, are performed to create a high-quality database by combining the existing datasets [3,26,28,29]. This process is essential and straightforward, but demands careful and intensive effort. It is of paramount importance in determining the research quality afterward. In addition, other than the common data information used in previous studies, we scrutinize the relevant publications to extract the earliest reporting year for an alloy composition. This enables dynamic analysis of the material data. Dynamical material networks are thus generated. At the current stage, we neglect the specific alloy composition and only focus on the alloy system. It is beneficial to avoid unnoticed uncertainties and undesired parsing errors. Meanwhile, this strategy permits better tolerance for data analysis and future model prediction. We aim to fully understand this coarse-graining strategy before attempting to identify a specific alloy composition. This also distinguishes us from previous works.

We separate the database into two groups based on the number of constituents, i.e. binary and ternary alloys. Reports on alloys with more components are comparatively rare. There are 94 systems with 38 elements for binary alloys, and 352 systems with 47 elements for ternary alloys. Based on the fundamental entities—edges for binary alloys and triangles for ternary alloys—we construct the material networks for each alloy. That is, with the 38 elements as nodes, we add a link between two elements if there is a binary alloy that can form a metallic glass, i.e. an existing data point in the database. For the ternary systems, we add a triangle with three links if there is a ternary alloy that can form a metallic glass. For consistency, we refer to them below as the binary network and ternary network. Thus, we stress that all our following data analysis is based on edges for the binary network and on triangles for the ternary network, even though the basic components of a graph are nodes ($0d$, $d$: dimensionality) and edges ($1d$). A triangle ($2d$) is a high-order object, which is important in shaping topology dynamics [31].

**Figure 1.**
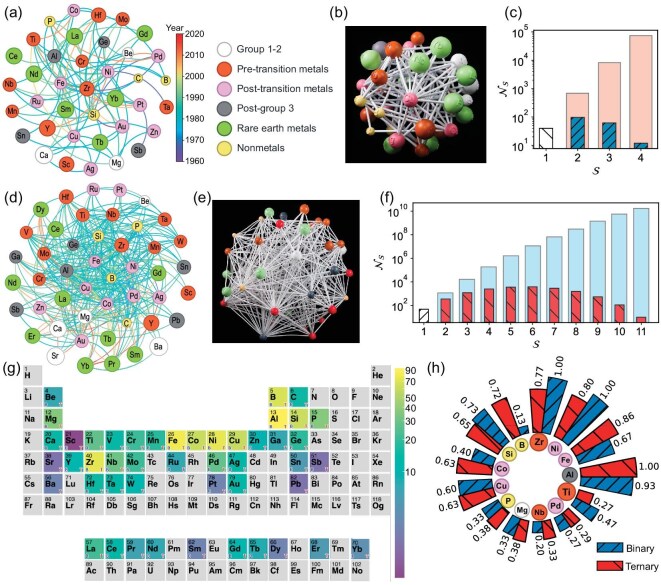
Network representations and analyses for amorphous alloys. (a) Network of binary alloys. The nodes are different elements colored by their periodic groups. Their diameters are scaled based on the atomic radii. Each edge dictates an experimentally discovered amorphous alloy with the two nodes at a certain year (see the color bar). There are 38 nodes and 94 edges. (b) The network of binary alloys fabricated by 3D printing. (c) Clique size distribution of the binary network. The number of cliques $\mathcal {N}_{\mathcal {S}}$ with $\mathcal {S}=1$ gives the number of nodes in (a). The light-shaded bars represent all possible geometric objects (${\bf C}_{38}^{\mathcal {S}}$) in the network, like rectangles for $\mathcal {S}=4$. (d) Network of ternary alloys, plotted in the same way as in (a), but based on triangles. Each triangle represents a ternary system. There are 47 nodes and 352 triangles, generating 348 edges. (e) The network of ternary alloys fabricated by 3D printing. The color schemes in (d) and (e) are the same as in (a) and (b), respectively. (f) Clique size distribution of the ternary network, similarly as in (c). The values represented by the light-shaded bars correspond to ${\bf C}_{47}^{\mathcal {S}}$. (g) A periodic table highlighting elements used in (d). The color bar indicates the number of ternary alloys containing each element. The capital letter “B” or “T” marks an element showing up in the binary or ternary network. (h) The numbers of edges and triangles of an element in the binary and ternary networks, normalized by the corresponding largest number. The largest degree in (a) is 16, and the largest number in (g) is 93.

The spatial layouts of these networks are optimized by using the Fruchterman-Reingold algorithm, as shown in Fig. 1a for the binary network and Fig. 1d for the ternary network (see the section entitled ‘Methods’). To enrich the visualization, the nodes are sized according to the metallic radii of the elements and then colored by their positions in the periodic table. Meanwhile, the edges are colored by the earliest invention year of the alloy system over different compositions. This strategy is utilized for both the binary network and the ternary network. Because of the large number of links in these networks—especially the ternary network—the visualizations become too cluttered to offer clear and meaningful insights. To address this problem, we propose constructing the corresponding realistic networks using three-dimensional (3D) printing. Panels (b) and (e) of Fig. 1 show the printed networks, with layouts carefully adjusted to ensure sufficient rigidity. Although the layout optimization is overlooked in the computational models, these examples highlight its importance [32]. The term ‘network’ typically refers to a realistic object, while ‘graph’ is used for a virtual representation; however, we use both interchangeably here for simplicity. These networks provide valuable three-dimensional insights to inspire our data analysis and graph mining in computation.

Motivated by these material networks, we begin to explore the material space. We first count the existing cliques in the networks. A clique is defined as a geometric object with each pair of its nodes connected. The number of nodes in a clique gives its size $\mathcal {S}$. For instance, a triangle has $\mathcal {S}=3$. In principle, $\mathcal {S}$ is mapped to the number of components of an alloy, but in a strict manner.

Figure 1c depicts the count distribution of the cliques in the binary network. The number of cliques $\mathcal {N}_\mathcal {S}$ with $\mathcal {S}=2$ in the binary network is 94. In fact, higher-order objects form automatically, even though only data with $\mathcal {S}=2$ are actually used. For example, fully connected triangles and rectangles exist, indicating the presence of three-component and four-component metallic glass formers. Furthermore, by directly considering all combinations of different numbers of elements (${\bf C}_{38}^\mathcal {S}$), we include the corresponding numbers in Fig. 1c for comparison. As the number of components increases, the alloy space expands exponentially, creating larger gaps between cliques. This highlights the growing difficulty of discovering multi-component systems, not to mention identifying a specific alloy composition The contrast in this plot also highlights promising first-trial candidates within the largely unexplored material space, as suggested by the binary network, which would likely be overlooked using conventional tabular data representations.

These analyses apply to the ternary network, as shown in Fig. 1f. Surprisingly, there are even more higher-order cliques forming automatically, with $\mathcal {S}$ up to 11. The numbers of cliques $\mathcal {N}_\mathcal {S}$ for $\mathcal {S}=1$ and $\mathcal {S}=3$ give the numbers of elements and ternary alloys, respectively. The other quantities depict the fully connected multi-component systems in the current network. Although the number of all possible combinations (${\bf C}_{47}^\mathcal {S}$) increases dramatically from ${\sim }10^3$ to ${\sim }10^{11}$ with ascending components, $\mathcal {N}_\mathcal {S}$ behaves like a normal distribution peaking at $\mathcal {S}=5$ or 6. Thus, the ternary network conveys more information and offers design advice for alloys with a wide range of components. Investigating these candidates can by itself serve as the first experimental advice. It also highlights the limited space that humans have explored. This aspect is naturally overlooked in traditional tabular representations. The difficulty in pinpointing a specific multi-component alloy aligns with efforts to uncover a hidden star in the Universe. We leave this challenge of quantitatively unveiling relationships between these groups and experimental discoveries to future studies.

In addition, the elemental contributions to the networks are justified. In Fig. 1g, we highlight the elements used in metallic glass formers in the periodic table. As expected, most of the elements are transition metals with rare-earth elements included. Some metalloids usually appear in amorphous alloys by minor alloying. The elements in the ternary network consist of those in the binary network, suggesting their hidden correlation. There are many more elements to be tested in future material design. More quantitatively, the top 13 elements that most frequently form amorphous alloys are shown in Fig. 1h for the ternary network, in comparison to the binary one. While Al appears in most of the ternary alloys, Ni and Zr are the most common in binary alloys. They can be defined as elemental hubs in the material networks (see below). This comparison indicates the unbalanced contribution of these elements in making up the material networks, implying limited knowledge or preferential attachment [33].

### Dynamical material networks

**Figure 2.**
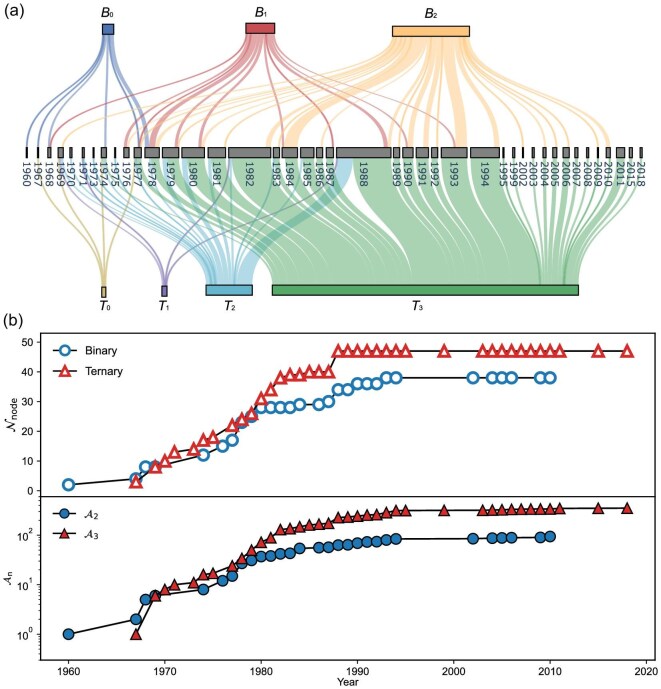
Discovery history of amorphous alloys. (a) A Sankey diagram illustrating the invention histories of binary ($B_n$) and ternary ($T_n$) amorphous alloys from 1960. They are classified based on how many elements have been used in previously developed amorphous alloys. For example, $B_1$ of 1988 demonstrates that one of the elements was used in the binary alloys developed before 1988. (b) Topology evolution of dynamical networks. The upper panel shows the number of nodes $\mathcal {N}_{\rm node}$, while the lower panel exhibits the number of links ($\mathcal {A}_2$) and triangles ($\mathcal {A}_3$) for the binary and ternary networks, respectively.

After recognizing the strength of network representation, we take the invention year of each system into consideration and construct the dynamical material networks by using the accumulated data to compensate data scarcity. In greater detail, the network of 1988 is constructed by all the nodes and edges/triangles discovered before 1988 and in 1988. This will help track the evolution path of amorphous alloys. Figure 2a presents a Sankey diagram illustrating the history of glass discovery in binary and ternary networks. Each set ($B_n$ or $T_n$) is further classified into sub-groups with different numbers of elements ($n$) that have been seen in previously invented alloys. For instance, $B_0$ and $T_0$ are brand-new alloy systems involving elements that have never been explored before; $T_3$ represents a class where all of the elements have been utilized before in other alloys, but not in the new one. In the early stages, mainly before 1978, brand-new alloy systems dominate, reflecting highly creative exploration. Over time, more and more systems are developed based on previously used elements in both networks. Especially after 1988, almost no new elements are introduced. This means that the nodes already appear in the network, but either a link or a triangle is missing. This verifies the effectiveness of the material candidates from the cliques in Fig. 1c and f.

This material invention pattern is further quantitatively proved by the yearly growth of the number of nodes $\mathcal {N}_{\rm node}$ (upper panel) and the number of entities $\mathcal {A}_n$ (link or triangle, lower panel) from the dynamical networks in Fig. 2b. In particular, $\mathcal {N}_{\rm node}$ saturates after around 1990. The period from 1970 to 1980 saw rapid growth in both $\mathcal {N}_{\rm node}$ and $\mathcal {A}_n$, leading to a plethora of new alloys that fueled the development of the field. After 2000, the alloy inventory enters a stagnant state. Investigating various properties of these amorphous alloys has become a main research focus since then.

These features highlight the effectiveness of the network representation, especially in its dynamical form. From a different perspective, an innovation trap emerges in the material design, inevitably after many elements have been tested. Knowledge gained from the literature motivates newcomers to explore hidden connections between existing elements in the invention pool, rather than truly exploring randomly. The designed materials may also already exist in the constructed material networks (see more discussion below). In essence, a knowledge graph was individually constructed to guide personal alloy design, which, to some extent, limits research creativity. We may expect this innovation trap to appear in many other material fields as well, which is an intriguing area for future exploration. It thrills us that introducing new elements may lead to unexpected breakthroughs [5]. Both enriching and enlarging the networks are crucial for future design.

### Topology analysis of material networks

**Figure 3.**
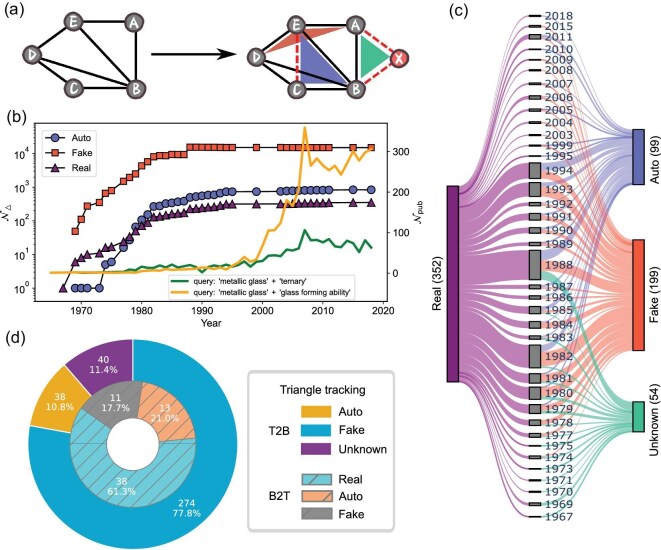
Triangle analysis in the ternary network. (a) Schematic illustration of triangle classification. By adding new triangles (*CDE* and *ABX*, marked by the dashed red lines), there are three types of triangles in addition to the existing ones. The blue triangle (*BCE*) automatically forms (Auto). The red triangle (*ADE*) lacks an edge, so it is a fake triangle (Fake). The green triangle (*ABX*) is created by adding a new node, so it is unknown from the left (Unknown). (b) The yearly growth of the number of classified triangles $\mathcal {N}_\Delta$. The number of publications $\mathcal {N}_{\rm pub}$ from the shown queries in the Web of Science database are plotted as lines for comparison. (c) A Sankey plot explaining the source of the developed ternary alloys (Real) at each year from the three groups. (d) Triangle tracking by cross comparing the binary and ternary networks. The outer ring shows the source of the 352 developed ternaries from the binary network (T2B). The inner ring illustrates the source of the 62 automatically formed triangles in the binary network from the ternary network (B2T).

In practice, ternary alloys usually have much better glass-forming ability than binary ones. This means that larger sample sizes are accessible from experiments, which is of great significance for property measurements and possible applications. This explains why the network in Fig. 1d and e is more complicated than that in Fig. 1a and b. In fact, the ternary network is considerably more complex in 3D printing. With this importance in mind, we carry out in-depth graph mining. We begin the discussion by defining several triangle units, illustrated in Fig. 3a. In the original schematic, there are five nodes with three triangle cliques, which represent the developed alloys. Starting with this topology, a new alloy, *CDE*, is explored. It automatically generates a candidate triangle clique *BCE*, which is thus defined as an auto triangle (‘Auto’). If not lacking a link, *ADE* would also become a clique, which is then defined as a fake triangle (‘Fake’). These are the possible new glass formers within the current network. In addition, by adding a new node X, there exists a triangle clique *ABX* that is unknown without X, so it is referred to as an unknown triangle (‘Unknown’). The former falls into the innovation trap, while the latter escapes it. Note that *ACE* belongs to Fake in the original plot. More explicitly, in the ternary network in Fig. 1d, there are 836 Auto triangles and 15 027 Fake triangles. If we look at the binary network in Fig. 1a, there are 62 Auto and 8374 Fake ones (see the online supplementary material for lists of these triangles). They may directly serve to guide experimental synthesis as a rule of thumb. In fact, when we check the most recent discoveries (Co-Ni-Ta [34] and Al-Fe-Pr [35]), they have been encoded in the ternary network (Auto for the former and Fake for the latter). Incorporating molecular dynamics simulations and density-functional-theory computations into the validation procedure is also of paramount significance in practice [36].

In Fig. 3b, we explore the triangle entity breakdown in dynamical material networks. In each year, we count the number of triangles falling into each class and show their numbers $\mathcal {N}_\Delta$ as a function of the year (see the Movies within the online supplementary material for the dynamical material networks). At first glance, there is rapid growth for all groups until approximately 1990. There are always many more Fake triangles in the network, indicating a large material space. Notably, there were initially more Real triangles, which, however, intersected with Auto around 1980. This reflects the ongoing exploration efforts of the network representation. The gaps among these groups persist to the present day because of the limited number of elements available for amorphous alloy design. There is still a huge space to motivate newly guided experimental synthesis. We also include the number of publications $\mathcal {N}_{\rm pub}$ queried in the Web of Science using different keywords. We note an evolving curve that slowly grows when considering ‘metallic glass’ and ‘ternary’ as the query keywords. The growth almost stops after reaching its peak in ${\sim }2007$. A rather different characteristic is observed when taking ‘metallic glass’ and ‘glass forming ability’ as the query keywords. In the period of intensive alloy discovery, there are publications that barely discuss the concept of glass-forming ability. The main research focus was on creating new amorphous alloys by trial-and-error experiments. After 2000, publications rose quickly, reaching saturation just before 2010. This growth pattern reflects the emergence of empirical rules and theoretical proposals for predicting glass-forming ability in the 2000s [15]. By summarizing the accumulated research experience and data before 2000, the field started to understand how to design new materials with a higher likelihood of success.

To unveil the innovation origin of the Real group, we show a Sankey plot in Fig. 3c. It shows when the 352 ternary alloys were invented: mostly in the 1980s and 1990s. Meaningfully, they are broken into groups of Unknown, Fake and Auto. Only a very small portion of them is from Unknown, which always shows up in the early stage. The other two groups span the past four decades. Strikingly, most of them are from Fake. The source from Auto indicates the effective design from the material network. Nevertheless, the success of Fake and Auto strongly supports the predictive power of the ternary network. Since 1989, all the developed metallic glasses (MGs) were already encoded in the network. For example, new ternary MGs discovered in 1994 all came from the material network constructed from ternary MGs developed before that year (Fake and Auto). This time-split validation strategy manifests the validation effectiveness in the past 20 years. Mining the candidates from Fig. 3c will be a valuable way to optimize the material design. For instance, Co-Ni-Ta and Al-Fe-Pr, the latest experimental discoveries in 2025 [34,35], have been encoded in the latest ternary network topology (see the online supplementary material for lists of these groups).

As a meaningful high-order object [31], the triangle entity naturally emerges in any network. We perform additional topology analysis to build hidden connections between the binary and ternary networks. In practice, we have two sets of triangles from these networks. By comparing them, we track the source of one group from the other. In the outer pie plot of Fig. 3d, we unearth how the 352 ternary amorphous alloys could be designed from the binary network. In detail, we find that, remarkably, nearly ${\sim } 78\%$ are from Fake triangles and ${\sim } 11\%$ from Auto. Only the left is from Unknown, demonstrating new elements in the ternary network. In turn, we examine how the 62 Auto triangles in the binary network can be predicted from the ternary network. Surprisingly, more than half actually form an amorphous alloy. Meanwhile, the rest are from the Auto and Fake groups, suggesting complete overlap between the two sets. This cross comparison conveys important messages. The strong overlap indicates the hidden connection between amorphous alloys with mutual elements but different numbers of components. The magic minor alloying could probably be understood from this new viewpoint. Additionally, we may have the capability to predict multi-component amorphous alloys by exploring the space of much simpler ones. This will enormously reduce the complexity. This is one of our most important ongoing research efforts assisted by advanced AI techniques.

Empirically, glass-forming ability is generally higher when more components are involved. At the same time, the material space grows logarithmically (see, for example, Fig. 1f). This renders limited available data for multi-component amorphous alloys. From the above cross-validations on the binary and ternary networks, beyond exploring the vast space of ternary alloys, material networks are also capable of predicting alloys with more components. For example, cliques with $\mathcal {S}>3$ can serve as such candidate materials for further screening. These network representations provide a promising alternative approach to addressing the glass-forming ability problem in metallic alloys with varying numbers of components.

### Material network analogies in everyday life

One of the most important properties of a given graph is the degree distribution of its nodes. The discovery of the scale-free feature in actor collaboration graphs, the World Wide Web and power grids opened a new era in network science [30]. It provides fundamental classifications of graphs. The preferential-attachment mechanism of network growth reveals the intriguing, trackable path of an evolving network, i.e. network dynamics. This has critical implications across many fields, including the internet, social networks, biological networks and pandemics.

**Figure 4.**
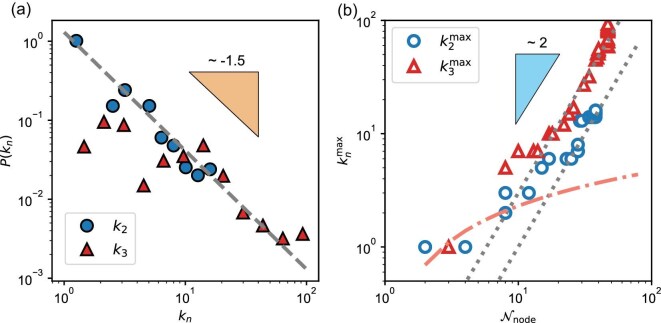
Scaling properties of the material networks. (a) Probability distribution of the network entity. We consider the degree of nodes ($k_2$) and the number of triangle nodes ($k_3$) in the binary and ternary networks, respectively. They are captured by a power-law relation, $P(k_n)\sim k_n^{-\gamma }$ with $\gamma \approx 1.5$ (the dashed line). (b) The dependence of the maximum of $k_n$ on the number of nodes $\mathcal {N}_{\rm node}$ from the dynamical networks (see Fig. 2). The dotted lines represent a power-law scaling, $k_{n}^{\rm max} \sim \mathcal {N}_{\rm node}^{1/(\gamma -1)}$, where $\gamma \approx 1.5$ for both. The orange dash–dot line represents a logarithmic scaling for a random network ($k_{n}^{\rm max} \sim \ln \mathcal {N}_{\rm node}$).

We show the probability distributions of the network entities in Fig. 4a. We emphasize that we consider the degree of nodes ($k_2$) for the binary network and the number of Real triangle nodes ($k_3$) for the ternary network. A scaling behavior of $P(k_n) \sim k_n^{-\gamma }$ with $\gamma \approx 1.5$ is observed. The exponent $\gamma$ does not fall into the normal scale-free regime ($\gamma \in [2,3]$). The Barabási–Albert model provides a foundational mechanism to generate scale-free networks with $\gamma =3$ [30]. Preferential attachment in network growth is its most critical feature. The scale-free feature implies that nodes with extremely large degrees are very rare, yet it does indicate the presence of hubs. A hub is a node whose degree is much higher than that of other nodes in the network. As the network grows, new nodes preferentially attach to these hubs, leading to the formation of graph communities—a typical characteristic of large networks.

From the aforementioned analyses, these features are preserved in our networks. On the one hand, the network grows with different probabilities for each node, which is driven by physical knowledge. On the other hand, there are ‘small’ elemental hubs—such as Al, Fe, Ni and Zr (see Fig. 1h)—that exhibit preferential-attachment features during alloy development. That is, amorphous alloys are typically designed to include these elements. Therefore, we classify our material networks as belonging to the abnormal scale-free class. This classification is inherently constrained by the limited number of elements in the periodic table. Even though all elements are supposed to be capable of being components in a metallic glass former, the maximum number of nodes is 118. The enrichment of links or triangles requires considerable experimental effort. But, at this stage, even high-throughput experiments are incapable of sampling the material space efficiently, let alone traditional methods.

To distinguish material networks from random networks, we compare the maximum degree $k_n^{\rm max}$ to the number of nodes $\mathcal {N}_{\rm node}$ in Fig. 4b. To complement the scarcity of the material data, here we consider dynamical networks for analysis. The outstanding observation is the faster growth of $k_n^{\rm max}$ following $\mathcal {N}_{\rm node}^{1/(\gamma -1)}$ with $\gamma \approx 1.5$ than the random growth following $k_n^{\rm max} \approx \ln \mathcal {N}_{\rm node}$. The increased discrepancy especially at large $\mathcal {N}_{\rm node}$ also indicates preferential attachment in the network growth.

**Figure 5.**
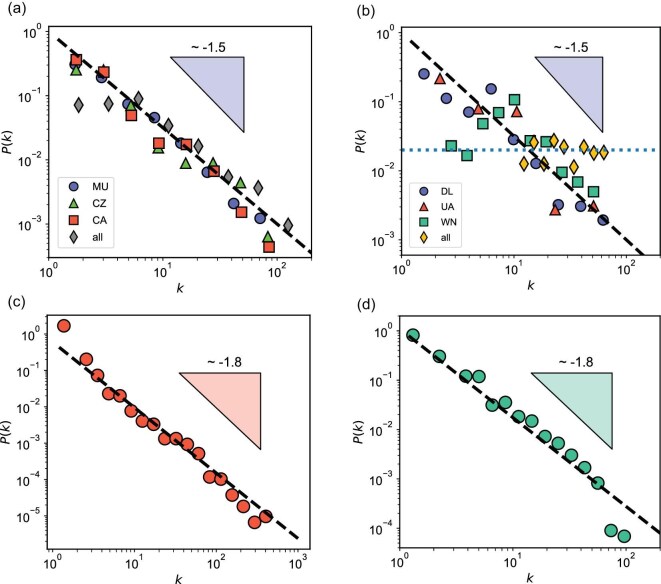
Scaling properties of real-world networks. The probability distributions of these networks are fitted to follow a power-law decay. (a) The Chinese flight network. The subgroup data for China Eastern Airlines (MU), China Southern Airlines (CZ) and Air China (CA) are shown for comparison. (b) The US flight network. The subgroup data for Delta Airlines (DL), United Airlines (UA) and Southwest Airlines (WN) are shown for comparison. (c) The email communication network. (d) The blog network. The power-law scaling, $P(k_n)\sim k_n^{-\gamma }$, holds for all these cases with $\gamma \approx 1.5$ for (a) and (b), and $\gamma \approx 1.8$ for (c) and (d).

We argue that these characteristics, driven by physical constraints, are not limited to our small physics-based material networks. In fact, networks whose nodes represent physical objects and whose links represent meaningful connections should exhibit similar behavior Clearly, the number of nodes and their corresponding degrees cannot be extrapolated to infinity. However, different networks operate at different length scales. Motivated by this idea, we explore various real-world networks and present some typical examples in Fig. 5, subject to data availability. The four examples are flight networks in China and in the United States, the email network and the blog network. In particular, the flight networks are broken down into different airlines. In all these cases, they faithfully obey a power-law decay with $P(k) \sim k^{-\gamma }$ and $\gamma < 2$, consistent with our material networks. This small exponent implies faster growth of links than nodes. The physical constraints are the airports in the flight networks and the users in the other two networks.

There are several possible factors that may affect the exponent. Firstly, note that $\gamma$ is slightly lower in smaller networks (material and flight networks) than larger networks (email and blog networks). This indicates that large networks may suffer less from physical constraints as there are many more nodes available and thus many more ways to grow. Secondly, we identified similar $\gamma$ values in the binary and ternary networks by examining edges and triangles, respectively. This demonstrates the effectiveness of the abnormal behavior under different effective edge definitions. Since our work is among the first to define higher-order objects as effective edges, there is considerable scope to quantitatively investigate how these definitions influence the scaling behavior. Thirdly, from our above discussion, adding nonexisting edges (or missing data) will increase the abnormality, because it increases the probability at higher node degrees (see Figs 4a and 5). Selectively pruning the networks may help reveal this hidden influence.

In Fig. 5b, when examining each major airline separately, the behavior $p(k) \sim k^{-1.5}$ is maintained. However, when combining the data from all airlines, $p(k)$ exhibits a plateau over a small range of $k$. With 70 nodes available, a fully connected network would require 2415 undirected edges (flights) to connect every pair of nodes (cities). In contrast, the flight network contains only 1262 undirected edges, which is sufficient to sustain traffic flow and give the observed $p(k)$ plateau. This may indicate that a fully connected network is not always necessary for optimal data flow. This scenario may apply to material networks. For example, for the current ternary network, there are 47 nodes, which give 16 215 ternary candidates if fully connected. Currently, the number is 352, far less than the hypothetical value. We thus speculate that, when a sufficient number of triangles (much less than 16 215) are created, we may get enough knowledge to understand the network. Then, we may get direct suggestions for many other candidates that are not generated. Nevertheless, how to generate such sufficient triangles in a proper order (the optimal pathway) is very challenging. Designing this pathway will require comprehensive domain knowledge, including expertise in amorphous alloys and network science. Once the sequence of alloy development is unveiled, it will be feasible to design targeted amorphous alloys in an efficient way. This deserves careful future investigations.

This will bridge network studies from different fields and enable knowledge sharing. Our material networks strongly depend on our understanding of fundamental physics at the atomic scale. However, real-world networks rely on large physical objects like airports or virtual human interactions. Apparently, they are distinct from each other. But, from the perspective of networks with physical constraints, they show very similar properties, highlighting universal principles governing constrained growth in complex networks. This is especially significant in materials science as there is only a limited number of elements available in the periodic table. By thoroughly investigating small networks, we may be able to understand networks at a larger length scale much better. A good strategy will be to consider small networks as hierarchically coarse-grained mappings of large networks.

## DISCUSSION

In this study, we propose material networks for amorphous alloys, focusing on binary and ternary metallic glass formers developed experimentally since the field’s inception. Network representations are superior to the traditional tabular data representation in many aspects. They provide alternative strategies for handling small datasets commonly encountered in materials science research. Unlike supervised machine learning methods suitable for tabular representation, networks can be more easily integrated with deep learning models such as graph neural networks.

With the assistance of 3D printing, we effectively performed graph mining over data-driven material networks. We also revealed the history of material discovery through dynamic material networks. This highlights the innovation trap in material discovery in which invented materials were already encoded in material networks. That is, material networks are capable of providing useful recommendations for new alloy design. This is further corroborated by the topology analysis of the ternary network. This implies an efficient design strategy to take advantage of the network representations. There are large unexplored material spaces revealed by material networks for binary and ternary amorphous alloys. We provided comprehensive recommendation lists for potential amorphous alloys from the network topology analysis. An alternative efficient way to making refined predictions or benchmarks is to use advanced machine learning techniques. We can build recommendation systems using graph neural networks with versatile algorithms and architectures. The predictive power of these models has been witnessed and will be reported separately.

In addition, the hidden connection between binary and ternary networks indicates the ability of the network representation to suggest multi-component amorphous alloys. These findings offer new insights for the optimal design of alloys in the future. More significantly, this network representation will play a crucial role in applying advanced artificial techniques in amorphous alloys design.

In current networks, we adopt a coarse-grained strategy by avoiding compositions to reduce complexity. Given the vast material space, it is quite challenging to directly identify an exact composition. Therefore, we instead follow a top-down approach: first identify a composition-free candidate system and then explore the phase diagram of the predicted system. A practical route for the latter is through high-throughput experiments (see, for example, [5]). In practice, for the binary network, we can incorporate the compositions into the edge features; for the ternary network, we can incorporate the compositions into either the involved edges or the triangle plane. In addition, we can design weights for edges, triangles or high-order objects, to reflect the properties of interest, such as maximum glass-forming ability, maximum critical size, width of the glass-forming composition range and mechanical, chemical or physical properties. We can then design graph neural networks to incorporate these features into the model architecture to make composition-resolved predictions with desired material properties. This is our ongoing effort to tackle the grand challenge of amorphous alloy design. The flexibility of integrating material properties into network representations opens a new avenue for designing additional properties in an intelligent way. In addition, including high-order object features is an important research topic in graph neural networks. We may expect cross-disciplinary breakthroughs in the future.

From the scaling analysis of the network degree of nodes defined by ordinary links and high-order triangles, we classify material networks as belonging to the abnormal scale-free class. Nevertheless, they display typical features that deviate from random networks, for example, element hubs and network growth with preferential attachment. This inherently originates from physical constraints, since there are a limited number of elements in the periodic table. The growth rate of the link or triangle is much faster than that of the node. We find analogous real-world networks that suffer from physical constraints, such as fight networks, the email communication network and the blog network. This similarity is likely to facilitate multi-disciplinary collaborative research and provide consulting clues for better decision-making. It also opens new avenues to uncover the mysteries of extremely large networks and to accelerate material discovery in a smarter way, especially in the era of AI.

Looking froward, the advent of AI techniques motivates text processing to create insightful knowledge graphs for materials science [37–40]. They reveal the complex semantic relationships between various material entities, such as name, properties, synthesis methods, characterizations and applications. The graph representation of these entities brings unprecedented insights hidden by the traditional plain tabular representation. From the perspective of graph representation, our material networks share many similarities with knowledge graphs. While knowledge graphs convey general semantic information, our material networks summarize amorphous alloys reported in the literature and offer focused information on alloy development. They capture the specific relationship among elements to deliver sophisticated insights for new alloy development. This is beyond the capability of knowledge graphs currently. However, careful distillation or refinement of comprehensive knowledge graphs will provide complementary strength to enhance the predictive power of material networks. Meanwhile, applying our strategies from material networks, like dynamical networks, to the design of knowledge graphs will also be beneficial. This offers an intriguing direction for future research aimed at integrating the two fields.

## METHODS

### Databases of amorphous alloys

We collect the experimental data for the developed binary and ternary amorphous alloys reported in the literature. We primarily combine four databases from previous works that are relevant to amorphous alloy research:

(1) the dataset used for machine learning prediction of ternary alloys to form glasses from Ren *et al.* [26];
(2) the dataset used to predict the glass-forming ability and supercooled liquid range of bulk amorphous alloys by Logan *et al.* [28];
(3) the dataset provided by Schultz *et al.* [29] that collects different physical properties for different amorphous alloys;
(4) the dataset of binary amorphous alloys created by Li *et al.* [3] to explore potential amorphous alloys.

To unify these datasets, we remove redundant items and further clean the data to keep unique records. Finally, a total of 12 206 data entries have been reported so far, with each composition identified as either capable of forming a glass or not. We create separate datasets by selecting the binary or ternary alloys for which at least one composition has been reported as amorphous. To track the development of these amorphous alloys, we also carefully checked these systems in the literature to extract the earliest year when a system is reported. We then consolidate these two datasets by neglecting the material compositions for further analysis.

### Material network of binary amorphous alloys

So far, our database lists 94 binary systems in which at least one composition has been fabricated into an amorphous alloy, whether in the form of a thin film, ribbon or bulk material. They are not differentiated here, because binary systems generally exhibit poor glass-forming ability and the available experimental data are scarce. We propose a graph representation of these systems by treating the involved elements as nodes and the binary systems as edges. Thus, a regular graph, as shown in Fig. 1a, is constructed. To inspire deeper insights, we build this graph as a real three-dimensional network by 3D printing (see Fig. 1b). The dynamical network is analyzed to acquire key scientific knowledge.

### Material network of ternary amorphous alloys

Our database currently reports a total of 352 ternary systems with at least one composition that has been fabricated into an amorphous alloy. We construct a graph representation for these systems by considering a higher-order unit, i.e. triangles, rather than links between pairs. Using the Fruchterman–Reingold algorithm [41], we generate the three-dimensional network layout in Fig. 1d, which contains 348 edges that connect any two elements as nodes. This structure is then printed to real-world objects by 3D printing to help gain better insights (see Fig. 1e). We then perform comprehensive data mining over the dynamical networks for ternary alloys.

### Material network construction algorithm

The spatial layout of the material networks is generated using the Fruchterman–Reingold algorithm [41]. It is a force-directed graph topology-built method that simulates a physical system. The algorithm conceptualizes nodes as charged particles repelling each other, while edges behave as springs attracting connected nodes. The pair repulsive force and attracting force are defined as $f_{\text{rep}}(R) = -{p^2}/{R}$ and $f_{\text{attr}}(R) = {R^2}/{p}$, respectively. Here $R$ denotes the Euclidean distance between nodes and $p$ is the optimal node separation constant. The final configuration is obtained by iteratively updating node positions based on the resultant forces until the system stabilizes, i.e. node displacements fall below a threshold. The network layout does not influence our current analyses.

### Real-world networks

To build hidden connections between scientific material networks and real-world instances, we came up with four analogous realistic networks that should suffer from physical constraints. These networks exist over different entities from totally different data resources. The first one is the Chinese flight network that combines data from multiple domestic airlines, collected from public online sources. In total, there are 187 nodes (airports) and 1761 edges (airlines). Note that different companies can fly the same route, but we do not take this edge attribute into account in this study, similar to how compositions are omitted in the material networks. The second one is the US airline flight network from major US carriers, developed using aviation datasets obtained from the OpenFlights database (https://openflights.org/). In total, there are 70 nodes and 1262 undirected edges. The third network is the email communication network, sourced from [42]. There are 1891 nodes and 4465 undirected edges. Each node dictates an email account and each link demonstrates an existing email communication between the users. The fourth is the blog network, sourced from [42,43]. There are 663 nodes and 2280 undirected edges. Each node represents a blog and each edge indicates citation existence between blogs. In our analysis, we focus on the scaling properties of the degree distribution of the nodes in these networks.

## Supplementary Material

nwaf398_Supplemental_Files
