# Supplementary material for: Constructing material network representations for intelligent amorphous alloy design
Source: Natl Sci Rev. 2025 Sep 19;12(11):nwaf398. doi: 10.1093/nsr/nwaf398 (PMC12573262; doi:10.1093/nsr/nwaf398)
Supplement: nwaf398_Supplemental_Files [file nwaf398_supplemental_files.zip › Supplemenatry data.pdf]

Supplementary Materials for ”Constructing material network representations for intelligent amorphous alloys design”

Shiyun Zhang†,[1] Jiachuan Tian†,[2] Songling Liu†,[1] Huimin Zhang,[1] Haiyang Bai,[1,3] Yuan-Chao Hu,[1,*] and Wei-Hua Wang[1,3]

[1]*Songshan Lake Materials Laboratory, Dongguan 523808, China*

[2]*Lawrence Berkeley National Laboratory, Berkeley CA 94720, USA*

[3]*Institute of Physics, Chinese Academy of Sciences, Beijing 100190, China*

* Email: yuanchao.hu@sslab.org.cn

†These authors contributed equally: S.Y.Z., J.T., L.S.L.

Contents

I. Real triangles and its breakdown in the ternary network 3
A. From Auto (99 triangles) 3
B. From Fake (199 triangles) 3
C. From Unknown (54 triangles) 4

II. Existing unexplored triangles in the ternary network 5
A. Auto (836 triangles) 5
B. Fake (15,027 triangles) 9

III. Existing unexplored triangles in the binary network 84
A. Auto (62 triagnles) 84
B. Fake (8,374 triangles) 84

I. Real triangles and its breakdown in the ternary network

A. From Auto (99 triangles)

Ag-Ca-Mg	Al-Ca-Cu	Al-Ca-Zn	Al-Co-Er	Al-Co-Fe	Al-Co-Gd	Al-Co-Ni	Al-Co-Si
Al-Co-Tb	Al-Co-Y	Al-Co-Zr	Al-Cr-Zr	Al-Cu-Fe	Al-Cu-La	Al-Cu-Nd	Al-Cu-Ti
Al-Cu-Y	Al-Fe-Gd	Al-Fe-Ge	Al-Fe-La	Al-Fe-Nd	Al-Fe-Ni	Al-Fe-Si	Al-Fe-Zr
Al-Mg-Zn	Al-Mn-Ni	Al-Mn-Si	Al-Mn-Zr	Al-Mo-Ni	Al-Mo-Si	Al-Nb-Ni	Al-Nb-Ti
Al-Ni-Si	Al-Ni-Zr	Au-Cu-Si	B-C-Co	B-C-Ni	B-Co-Ni	B-Co-P	B-Co-Si
B-Co-Ta	B-Co-V	B-Co-Zr	B-Cu-Zr	B-Fe-Nb	B-Fe-Pd	B-Fe-Ta	B-Fe-Ti
B-Fe-W	B-Fe-Y	B-Mo-Ni	B-Mo-Ru	B-Ni-P	C-Co-Si	C-Ni-Si	Ce-Cu-Mg
Co-Fe-P	Co-Fe-Zr	Co-Mn-Zr	Co-Nb-Zr	Co-Ni-Zr	Co-P-Zr	Co-Pd-Zr	Co-Si-Ti
Co-Si-Zr	Co-Ti-Zr	Cr-Fe-P	Cr-Fe-Zr	Cr-Ni-Zr	Cr-Si-Zr	Cu-Fe-Ti	Cu-Fe-Zr
Cu-Hf-Ni	Cu-La-Mg	Cu-Ni-P	Cu-Ni-Ti	Cu-Ni-Zr	Cu-P-Pd	Cu-Ti-Zr	Fe-Mn-P
Fe-Mo-P	Fe-Nb-Ti	Fe-Ni-P	Fe-Ni-Zr	Fe-P-Pd	Fe-Pd-Zr	Fe-Si-Zr	Mo-Ni-P
Mo-Si-Zr	Nb-Ni-Ta	Nb-Ni-Zr	Ni-P-W	Ni-P-Zr	Ni-Pd-Zr	Ni-Si-Zr	Ni-Ti-Zr
Si-Ti-Zr	Si-V-Zr	Si-W-Zr					

B. From Fake (199 triangles)

Ag-Al-Ca	Ag-Al-La	Ag-Al-Mg	Ag-Ce-Cu	Ag-Cu-Fe	Ag-Cu-Ge	Ag-Cu-Mg	Ag-Cu-P
Ag-Cu-Zr	Ag-Mg-Y	Ag-Mg-Yb	Al-Au-La	Al-B-Co	Al-B-Fe	Al-B-Ni	Al-B-Tb
Al-Be-Ti	Al-Ca-Co	Al-Ca-Fe	Al-Ca-Ga	Al-Ca-Mg	Al-Ca-Ni	Al-Co-Dy	Al-Co-Ge
Al-Co-La	Al-Co-Nd	Al-Co-Pr	Al-Co-Sm	Al-Cr-Ge	Al-Cr-La	Al-Cr-Si	Al-Cu-Gd
Al-Cu-Hf	Al-Cu-Pr	Al-Cu-V	Al-Cu-Zr	Al-Er-Ni	Al-Fe-Tb	Al-Fe-Y	Al-Ga-Mg
Al-Gd-Ni	Al-Ge-Mn	Al-Ge-Ni	Al-Ge-V	Al-Hf-Ni	Al-La-Mn	Al-La-Mo	Al-La-Nb
Al-La-Ni	Al-La-Pt	Al-La-Zn	Al-La-Zr	Al-Mg-Pd	Al-Mg-Ti	Al-Nd-Ni	Al-Ni-Sm
Al-Ni-Ti	Al-Ni-Y	Al-Si-Zr	Au-B-Fe	Au-B-Gd	Au-Cu-La	Au-Cu-Mg	Au-Mg-Zn

B-Be-Fe	B-C-Fe	B-Co-Cr	B-Co-Er	B-Co-Fe	B-Co-Mn	B-Co-Nb	B-Co-Ti
B-Cr-Fe	B-Cr-Ni	B-Cu-Fe	B-Cu-Ni	B-Fe-Ge	B-Fe-Hf	B-Fe-Mn	B-Fe-Mo
B-Fe-Ni	B-Fe-Si	B-Fe-Sn	B-Fe-Tb	B-Fe-V	B-Fe-Zr	B-Ga-Gd	B-Mo-P
B-Mo-Si	B-Nb-Ni	B-Ni-Si	B-Ni-Ta	B-Ni-Ti	B-Ni-W	B-Ni-Zr	B-Pd-Si
B-Si-W	Be-Cu-Zr	Be-Hf-Zr	Be-Si-Ti	C-Co-Cr	C-Co-Mo	C-Cr-Fe	C-Fe-Mo
C-Fe-Si	C-Mo-Ni	C-Nb-Si	Ca-Cu-La	Ca-Ga-Mg	Ca-Mg-Pd	Ca-Mg-Zn	Ce-Cu-Yb
Ce-Mg-Zn	Co-Cr-P	Co-Cr-Zr	Co-Fe-Nb	Co-Fe-Tb	Co-Hf-P	Co-Hf-Si	Co-Mn-Nb
Co-Mo-Zr	Co-Nb-Ni	Co-Ni-Ta	Co-V-Zr	Co-W-Zr	Cr-Ge-Pd	Cr-Mo-Ni	Cr-Ni-P
Cu-Dy-Mg	Cu-Er-Mg	Cu-Ga-Zr	Cu-Gd-Mg	Cu-Hf-Ti	Cu-Mg-Nd	Cu-Mg-Pr	Cu-Mg-Tb
Cu-Mg-Y	Cu-Nb-Sn	Cu-Nb-Ti	Cu-Nb-Zr	Cu-Ni-Pr	Cu-P-Zr	Fe-Ga-Gd	Fe-Gd-Zr
Fe-Ge-Zr	Fe-Hf-Ta	Fe-Hf-Zr	Fe-La-Zr	Fe-Mn-Zr	Fe-P-Si	Fe-P-V	Fe-Pd-Si
Fe-Pr-Zr	Fe-Ru-Zr	Fe-Si-Tb	Fe-Si-Ti	Fe-V-Zr	Gd-Mg-Ni	Ge-Mn-Pd	Ge-Mo-Zr
Ge-Nb-Si	Ge-Nb-Zr	Ge-Ta-Zr	Ge-Ti-Zr	Ge-V-Zr	Hf-Nb-Si	Hf-Ni-P	Hf-Si-V
Hf-Si-Zr	La-Mg-Ni	La-Mg-Zn	Mg-Mn-Y	Mg-Nd-Ni	Mg-Ni-Y	Mg-Pd-Yb	Mg-Sr-Zn
Mn-Ni-Zr	Mn-P-Pd	Mn-P-Si	Mo-Nb-Si	Nb-Ni-Pd	Nb-Ni-Pt	Nb-Ni-Sn	Nb-Si-Ti
Nb-Si-W	Nb-Si-Zr	Ni-P-Ta	Ni-P-Ti	Ni-Pd-Si	Ni-Pd-Ta	Ni-Ru-Ta	Ni-Si-Ti
Ni-V-Zr	P-Pd-Ti	Pd-Si-Zr	Ru-Si-Zr	Si-Ta-Ti	Si-Ta-Zr	Si-Ti-V	

C. From Unknown (54 triangles)

Ag-Pd-Si	Al-Ce-Co	Al-Ce-Cr	Al-Ce-Cu	Al-Ce-Fe	Al-Ce-Mn	Al-Ce-Nb	Al-Ce-Ni
Al-Ce-V	Al-Fe-P	Au-B-Er	Au-B-Pr	Au-B-Tb	Au-Ge-Si	Au-Pb-Sb	Au-Pd-Si
B-Co-Y	B-Fe-Ga	B-Fe-La	B-Fe-Nd	B-Fe-P	B-Fe-Sm	B-Ru-W	Ba-Cu-La
Ba-Cu-Y	Ba-Cu-Yb	Be-Nb-Zr	Be-Ti-Zr	C-Co-W	C-Fe-P	C-Fe-W	Ca-Cu-Mg
Ce-Mg-Ni	Co-Gd-Mo	Co-Ni-P	Co-Pd-Si	Cr-Pd-Si	Cu-Hf-Nb	Cu-La-Sr	Cu-Pb-Zn
Cu-Pd-Si	Dy-Fe-Zr	Fe-Sc-Zr	Ge-Pd-Si	Mg-Sn-Zn	Mn-Pd-Si	Mo-P-Ru	Nb-Si-Ta
Nb-Si-V	Ni-P-Pd	Ni-P-Pt	P-Ru-W	Pd-Sb-Si	Si-Sn-Zr		

II. Existing unexplored triangles in the ternary network

A. Auto (836 triangles)

Ag-Al-Ce	Ag-Al-Cu	Ag-Al-Fe	Ag-Al-Ge	Ag-Al-P	Ag-Al-Pd	Ag-Al-Si	Ag-Al-Y
Ag-Al-Zr	Ag-Ca-Cu	Ag-Ca-Fe	Ag-Ca-La	Ag-Ca-Pd	Ag-Ce-Fe	Ag-Ce-Mg	Ag-Ce-Yb
Ag-Cu-La	Ag-Cu-Pd	Ag-Cu-Si	Ag-Cu-Y	Ag-Cu-Yb	Ag-Fe-Ge	Ag-Fe-La	Ag-Fe-P
Ag-Fe-Pd	Ag-Fe-Si	Ag-Fe-Y	Ag-Fe-Zr	Ag-Ge-Pd	Ag-Ge-Si	Ag-Ge-Zr	Ag-La-Mg
Ag-La-Zr	Ag-Mg-Pd	Ag-P-Pd	Ag-P-Si	Ag-P-Zr	Ag-Pd-Yb	Ag-Pd-Zr	Ag-Si-Zr
Al-Au-B	Al-Au-Cu	Al-Au-Er	Al-Au-Fe	Al-Au-Gd	Al-Au-Ge	Al-Au-Mg	Al-Au-Pd
Al-Au-Pr	Al-Au-Si	Al-Au-Tb	Al-Au-Zn	Al-B-Be	Al-B-Cr	Al-B-Cu	Al-B-Er
Al-B-Ga	Al-B-Gd	Al-B-Ge	Al-B-Hf	Al-B-La	Al-B-Mn	Al-B-Mo	Al-B-Nb
Al-B-Nd	Al-B-P	Al-B-Pd	Al-B-Pr	Al-B-Si	Al-B-Sm	Al-B-Ti	Al-B-V
Al-B-Y	Al-B-Zr	Al-Be-Cu	Al-Be-Fe	Al-Be-Hf	Al-Be-Nb	Al-Be-Si	Al-Be-Zr
Al-Ca-La	Al-Ca-Pd	Al-Ce-Mg	Al-Ce-Zn	Al-Co-Cr	Al-Co-Hf	Al-Co-Mn	Al-Co-Mo
Al-Co-Nb	Al-Co-P	Al-Co-Pd	Al-Co-Ti	Al-Co-V	Al-Cr-Fe	Al-Cr-Mo	Al-Cr-Ni
Al-Cr-P	Al-Cr-Pd	Al-Cu-Dy	Al-Cu-Er	Al-Cu-Ga	Al-Cu-Ge	Al-Cu-Mg	Al-Cu-Nb
Al-Cu-Ni	Al-Cu-P	Al-Cu-Pd	Al-Cu-Si	Al-Cu-Tb	Al-Cu-Zn	Al-Dy-Fe	Al-Dy-Mg
Al-Dy-Zr	Al-Er-Mg	Al-Fe-Ga	Al-Fe-Hf	Al-Fe-Mn	Al-Fe-Mo	Al-Fe-Nb	Al-Fe-Pd
Al-Fe-Pr	Al-Fe-Sm	Al-Fe-Ti	Al-Fe-V	Al-Ga-Gd	Al-Ga-Zr	Al-Gd-Mg	Al-Gd-Mo
Al-Gd-Zr	Al-Ge-Mo	Al-Ge-Nb	Al-Ge-Pd	Al-Ge-Si	Al-Ge-Ti	Al-Ge-Zr	Al-Hf-Nb
Al-Hf-P	Al-Hf-Si	Al-Hf-Ti	Al-Hf-V	Al-Hf-Zr	Al-La-Mg	Al-Mg-Mn	Al-Mg-Nd
Al-Mg-Ni	Al-Mg-Pr	Al-Mg-Tb	Al-Mg-Y	Al-Mn-Nb	Al-Mn-P	Al-Mn-Pd	Al-Mn-Y
Al-Mo-Nb	Al-Mo-P	Al-Mo-Zr	Al-Nb-Pd	Al-Nb-Pt	Al-Nb-Si	Al-Nb-V	Al-Nb-Zr
Al-Ni-P	Al-Ni-Pd	Al-Ni-Pr	Al-Ni-Pt	Al-Ni-V	Al-P-Pd	Al-P-Pt	Al-P-Si
Al-P-Ti	Al-P-V	Al-P-Zr	Al-Pd-Si	Al-Pd-Ti	Al-Pd-Zr	Al-Pr-Zr	Al-Si-Tb
Al-Si-Ti	Al-Si-V	Al-Ti-V	Al-Ti-Zr	Al-V-Zr	Au-B-Cu	Au-B-Ge	Au-B-La

Au-B-Pd	Au-B-Si	Au-Cu-Er	Au-Cu-Fe	Au-Cu-Gd	Au-Cu-Ge	Au-Cu-Pb	Au-Cu-Pd
Au-Cu-Pr	Au-Cu-Tb	Au-Cu-Zn	Au-Er-Mg	Au-Fe-Gd	Au-Fe-Ge	Au-Fe-La	Au-Fe-Pd
Au-Fe-Pr	Au-Fe-Si	Au-Fe-Tb	Au-Gd-Mg	Au-Ge-Pd	Au-La-Mg	Au-La-Zn	Au-Mg-Pd
Au-Mg-Pr	Au-Mg-Tb	Au-Pb-Zn	Au-Pd-Sb	Au-Sb-Si	Au-Si-Tb	B-Be-Cu	B-Be-Hf
B-Be-Nb	B-Be-Si	B-Be-Ti	B-Be-Zr	B-C-Cr	B-C-Mo	B-C-Nb	B-C-P
B-C-Si	B-C-W	B-Co-Gd	B-Co-Ge	B-Co-Hf	B-Co-La	B-Co-Mo	B-Co-Nd
B-Co-Pd	B-Co-Pr	B-Co-Sm	B-Co-Tb	B-Co-W	B-Cr-Ge	B-Cr-La	B-Cr-Mo
B-Cr-P	B-Cr-Pd	B-Cr-Si	B-Cr-Zr	B-Cu-Er	B-Cu-Ga	B-Cu-Gd	B-Cu-Ge
B-Cu-Hf	B-Cu-La	B-Cu-Nb	B-Cu-Nd	B-Cu-P	B-Cu-Pd	B-Cu-Pr	B-Cu-Si
B-Cu-Sn	B-Cu-Tb	B-Cu-Ti	B-Cu-V	B-Cu-Y	B-Er-Ni	B-Fe-Gd	B-Fe-Pr
B-Fe-Ru	B-Ga-Zr	B-Gd-Mo	B-Gd-Ni	B-Gd-Zr	B-Ge-Mn	B-Ge-Mo	B-Ge-Nb
B-Ge-Ni	B-Ge-Pd	B-Ge-Si	B-Ge-Ta	B-Ge-Ti	B-Ge-V	B-Ge-Zr	B-Hf-Nb
B-Hf-Ni	B-Hf-P	B-Hf-Si	B-Hf-Ta	B-Hf-Ti	B-Hf-V	B-Hf-Zr	B-La-Mn
B-La-Mo	B-La-Nb	B-La-Ni	B-La-Zr	B-Mn-Nb	B-Mn-Ni	B-Mn-P	B-Mn-Pd
B-Mn-Si	B-Mn-Y	B-Mn-Zr	B-Mo-Nb	B-Mo-Zr	B-Nb-Pd	B-Nb-Si	B-Nb-Sn
B-Nb-Ta	B-Nb-Ti	B-Nb-V	B-Nb-W	B-Nb-Zr	B-Nd-Ni	B-Ni-Pd	B-Ni-Pr
B-Ni-Ru	B-Ni-Sm	B-Ni-Sn	B-Ni-V	B-Ni-Y	B-P-Pd	B-P-Ru	B-P-Si
B-P-Ta	B-P-Ti	B-P-V	B-P-W	B-P-Zr	B-Pd-Ta	B-Pd-Ti	B-Pd-Zr
B-Pr-Zr	B-Ru-Si	B-Ru-Ta	B-Ru-Zr	B-Si-Sn	B-Si-Ta	B-Si-Tb	B-Si-Ti
B-Si-V	B-Si-Zr	B-Sn-Zr	B-Ta-Ti	B-Ta-Zr	B-Ti-V	B-Ti-Zr	B-V-Zr
B-W-Zr	Be-Cu-Fe	Be-Cu-Hf	Be-Cu-Nb	Be-Cu-Si	Be-Cu-Ti	Be-Fe-Hf	Be-Fe-Nb
Be-Fe-Si	Be-Fe-Ti	Be-Fe-Zr	Be-Hf-Nb	Be-Hf-Si	Be-Hf-Ti	Be-Nb-Si	Be-Nb-Ti
Be-Si-Zr	C-Co-Fe	C-Co-Nb	C-Co-Ni	C-Co-P	C-Cr-Mo	C-Cr-Ni	C-Cr-P
C-Cr-Si	C-Fe-Nb	C-Fe-Ni	C-Mo-Nb	C-Mo-P	C-Mo-Si	C-Nb-Ni	C-Nb-W
C-Ni-P	C-Ni-W	C-P-Si	C-P-W	C-Si-W	Ca-Co-Fe	Ca-Co-La	Ca-Co-Ni

Ca-Co-Pd	Ca-Cu-Fe	Ca-Cu-Ga	Ca-Cu-Ni	Ca-Cu-Pd	Ca-Cu-Zn	Ca-Fe-Ga	Ca-Fe-La
Ca-Fe-Ni	Ca-Fe-Pd	Ca-La-Mg	Ca-La-Ni	Ca-La-Zn	Ca-Mg-Ni	Ca-Ni-Pd	Ce-Co-Cr
Ce-Co-Fe	Ce-Co-Mn	Ce-Co-Nb	Ce-Co-Ni	Ce-Co-V	Ce-Cr-Fe	Ce-Cr-Ni	Ce-Cu-Fe
Ce-Cu-Nb	Ce-Cu-Ni	Ce-Cu-V	Ce-Cu-Zn	Ce-Fe-Mn	Ce-Fe-Nb	Ce-Fe-Ni	Ce-Fe-V
Ce-Mg-Mn	Ce-Mg-Yb	Ce-Mn-Nb	Ce-Mn-Ni	Ce-Nb-Ni	Ce-Nb-V	Ce-Ni-V	Co-Cr-Fe
Co-Cr-Ge	Co-Cr-La	Co-Cr-Mo	Co-Cr-Ni	Co-Cr-Pd	Co-Cr-Si	Co-Dy-Fe	Co-Dy-Zr
Co-Er-Ni	Co-Fe-Gd	Co-Fe-Ge	Co-Fe-Hf	Co-Fe-La	Co-Fe-Mn	Co-Fe-Mo	Co-Fe-Nd
Co-Fe-Ni	Co-Fe-Pd	Co-Fe-Pr	Co-Fe-Si	Co-Fe-Sm	Co-Fe-Ta	Co-Fe-Ti	Co-Fe-V
Co-Fe-W	Co-Fe-Y	Co-Gd-Ni	Co-Gd-Zr	Co-Ge-Mn	Co-Ge-Mo	Co-Ge-Nb	Co-Ge-Ni
Co-Ge-Pd	Co-Ge-Si	Co-Ge-Ta	Co-Ge-Ti	Co-Ge-V	Co-Ge-Zr	Co-Hf-Nb	Co-Hf-Ni
Co-Hf-Ta	Co-Hf-Ti	Co-Hf-V	Co-Hf-Zr	Co-La-Mn	Co-La-Mo	Co-La-Nb	Co-La-Ni
Co-La-Zr	Co-Mn-Ni	Co-Mn-P	Co-Mn-Pd	Co-Mn-Si	Co-Mn-Y	Co-Mo-Nb	Co-Mo-Ni
Co-Mo-P	Co-Mo-Si	Co-Nb-Pd	Co-Nb-Si	Co-Nb-Ta	Co-Nb-Ti	Co-Nb-V	Co-Nb-W
Co-Nd-Ni	Co-Ni-Pd	Co-Ni-Pr	Co-Ni-Si	Co-Ni-Sm	Co-Ni-Ti	Co-Ni-V	Co-Ni-W
Co-Ni-Y	Co-P-Pd	Co-P-Si	Co-P-Ta	Co-P-Ti	Co-P-V	Co-P-W	Co-Pd-Ta
Co-Pd-Ti	Co-Pr-Zr	Co-Si-Ta	Co-Si-Tb	Co-Si-V	Co-Si-W	Co-Ta-Ti	Co-Ta-Zr
Co-Ti-V	Cr-Fe-Ge	Cr-Fe-La	Cr-Fe-Mo	Cr-Fe-Ni	Cr-Fe-Pd	Cr-Fe-Si	Cr-Ge-Mo
Cr-Ge-Ni	Cr-Ge-Si	Cr-Ge-Zr	Cr-La-Mo	Cr-La-Ni	Cr-La-Zr	Cr-Mo-P	Cr-Mo-Si
Cr-Mo-Zr	Cr-Ni-Pd	Cr-Ni-Si	Cr-P-Pd	Cr-P-Si	Cr-P-Zr	Cr-Pd-Zr	Cu-Dy-Fe
Cu-Dy-Zr	Cu-Er-Ni	Cu-Fe-Ga	Cu-Fe-Gd	Cu-Fe-Ge	Cu-Fe-Hf	Cu-Fe-La	Cu-Fe-Nb
Cu-Fe-Nd	Cu-Fe-Ni	Cu-Fe-P	Cu-Fe-Pd	Cu-Fe-Pr	Cu-Fe-Si	Cu-Fe-Sn	Cu-Fe-Tb
Cu-Fe-V	Cu-Fe-Y	Cu-Ga-Gd	Cu-Ga-Mg	Cu-Gd-Ni	Cu-Gd-Zr	Cu-Ge-Nb	Cu-Ge-Ni
Cu-Ge-Pd	Cu-Ge-Si	Cu-Ge-Ti	Cu-Ge-V	Cu-Ge-Zr	Cu-Hf-P	Cu-Hf-Si	Cu-Hf-V
Cu-Hf-Zr	Cu-La-Nb	Cu-La-Ni	Cu-La-Zn	Cu-La-Zr	Cu-Mg-Ni	Cu-Mg-Pd	Cu-Mg-Sn
Cu-Mg-Sr	Cu-Mg-Ti	Cu-Mg-Yb	Cu-Mg-Zn	Cu-Nb-Ni	Cu-Nb-Pd	Cu-Nb-Si	Cu-Nb-V

Cu-Nd-Ni	Cu-Ni-Pd	Cu-Ni-Si	Cu-Ni-Sn	Cu-Ni-V	Cu-Ni-Y	Cu-P-Si	Cu-P-Ti
Cu-P-V	Cu-Pd-Ti	Cu-Pd-Yb	Cu-Pd-Zr	Cu-Pr-Zr	Cu-Si-Sn	Cu-Si-Tb	Cu-Si-Ti
Cu-Si-V	Cu-Si-Zr	Cu-Sn-Zn	Cu-Sn-Zr	Cu-Sr-Zn	Cu-Ti-V	Cu-V-Zr	Er-Mg-Ni
Fe-Ga-Zr	Fe-Gd-Mo	Fe-Gd-Ni	Fe-Ge-Mn	Fe-Ge-Mo	Fe-Ge-Nb	Fe-Ge-Ni	Fe-Ge-Pd
Fe-Ge-Si	Fe-Ge-Ta	Fe-Ge-Ti	Fe-Ge-V	Fe-Hf-Nb	Fe-Hf-Ni	Fe-Hf-P	Fe-Hf-Si
Fe-Hf-Ti	Fe-Hf-V	Fe-La-Mn	Fe-La-Mo	Fe-La-Nb	Fe-La-Ni	Fe-Mn-Nb	Fe-Mn-Ni
Fe-Mn-Pd	Fe-Mn-Si	Fe-Mn-Y	Fe-Mo-Nb	Fe-Mo-Ni	Fe-Mo-Ru	Fe-Mo-Si	Fe-Mo-Zr
Fe-Nb-Ni	Fe-Nb-Pd	Fe-Nb-Si	Fe-Nb-Sn	Fe-Nb-Ta	Fe-Nb-V	Fe-Nb-W	Fe-Nb-Zr
Fe-Nd-Ni	Fe-Ni-Pd	Fe-Ni-Pr	Fe-Ni-Ru	Fe-Ni-Si	Fe-Ni-Sm	Fe-Ni-Sn	Fe-Ni-Ta
Fe-Ni-Ti	Fe-Ni-V	Fe-Ni-W	Fe-Ni-Y	Fe-P-Ru	Fe-P-Ta	Fe-P-Ti	Fe-P-W
Fe-P-Zr	Fe-Pd-Ta	Fe-Pd-Ti	Fe-Ru-Si	Fe-Ru-Ta	Fe-Ru-W	Fe-Si-Sn	Fe-Si-Ta
Fe-Si-V	Fe-Si-W	Fe-Sn-Zr	Fe-Ta-Ti	Fe-Ta-Zr	Fe-Ti-V	Fe-Ti-Zr	Fe-W-Zr
Ga-Gd-Mg	Ga-Gd-Zr	Gd-Mo-Ni	Gd-Mo-Zr	Gd-Ni-Zr	Ge-Mn-Nb	Ge-Mn-Ni	Ge-Mn-Si
Ge-Mn-Zr	Ge-Mo-Nb	Ge-Mo-Ni	Ge-Mo-Si	Ge-Nb-Ni	Ge-Nb-Pd	Ge-Nb-Ta	Ge-Nb-Ti
Ge-Nb-V	Ge-Ni-Pd	Ge-Ni-Si	Ge-Ni-Ta	Ge-Ni-Ti	Ge-Ni-V	Ge-Ni-Zr	Ge-Pd-Ta
Ge-Pd-Ti	Ge-Pd-Zr	Ge-Si-Ta	Ge-Si-Ti	Ge-Si-V	Ge-Si-Zr	Ge-Ta-Ti	Ge-Ti-V
Hf-Nb-Ni	Hf-Nb-Ta	Hf-Nb-Ti	Hf-Nb-V	Hf-Nb-Zr	Hf-Ni-Si	Hf-Ni-Ta	Hf-Ni-Ti
Hf-Ni-V	Hf-Ni-Zr	Hf-P-Si	Hf-P-Ta	Hf-P-Ti	Hf-P-V	Hf-P-Zr	Hf-Si-Ta
Hf-Si-Ti	Hf-Ta-Ti	Hf-Ta-Zr	Hf-Ti-V	Hf-Ti-Zr	Hf-V-Zr	La-Mg-Mn	La-Mg-Sr
La-Mn-Nb	La-Mn-Ni	La-Mn-Zr	La-Mo-Nb	La-Mo-Ni	La-Mo-Zr	La-Nb-Ni	La-Nb-Pt
La-Nb-Zr	La-Ni-Pt	La-Ni-Zr	La-Sr-Zn	Mg-Mn-Ni	Mg-Mn-Pd	Mg-Ni-Pd	Mg-Ni-Pr
Mg-Ni-Sn	Mg-Ni-Ti	Mg-Pd-Ti	Mn-Nb-Ni	Mn-Nb-Pd	Mn-Nb-Si	Mn-Nb-Zr	Mn-Ni-P
Mn-Ni-Pd	Mn-Ni-Si	Mn-Ni-Y	Mn-P-Zr	Mn-Pd-Zr	Mn-Si-Zr	Mo-Nb-Ni	Mo-Nb-Zr
Mo-Ni-Ru	Mo-Ni-Si	Mo-Ni-Zr	Mo-P-Si	Mo-P-Zr	Mo-Ru-Si	Mo-Ru-Zr	Nb-Ni-Si
Nb-Ni-Ti	Nb-Ni-V	Nb-Ni-W	Nb-Pd-Si	Nb-Pd-Ta	Nb-Pd-Ti	Nb-Pd-Zr	Nb-Si-Sn

Nb-Sn-Zr	Nb-Ta-Ti	Nb-Ta-Zr	Nb-Ti-V	Nb-Ti-Zr	Nb-V-Zr	Nb-W-Zr	Ni-P-Ru
Ni-P-Si	Ni-P-V	Ni-Pd-Ti	Ni-Pr-Zr	Ni-Ru-Si	Ni-Ru-W	Ni-Ru-Zr	Ni-Si-Sn
Ni-Si-Ta	Ni-Si-V	Ni-Si-W	Ni-Sn-Zr	Ni-Ta-Ti	Ni-Ta-Zr	Ni-Ti-V	Ni-W-Zr
P-Pd-Si	P-Pd-Ta	P-Pd-Zr	P-Ru-Si	P-Ru-Ta	P-Ru-Zr	P-Si-Ta	P-Si-Ti
P-Si-V	P-Si-W	P-Si-Zr	P-Ta-Ti	P-Ta-Zr	P-Ti-V	P-Ti-Zr	P-V-Zr
P-W-Zr	Pd-Si-Ta	Pd-Si-Ti	Pd-Ta-Ti	Pd-Ta-Zr	Pd-Ti-Zr	Ru-Si-Ta	Ru-Si-W
Ru-Ta-Zr	Ru-W-Zr	Ta-Ti-Zr	Ti-V-Zr				

B. Fake (15,027 triangles)

Ag-Al-Au	Ag-Al-B	Ag-Al-Ba	Ag-Al-Be	Ag-Al-C	Ag-Al-Co	Ag-Al-Cr	Ag-Al-Dy
Ag-Al-Er	Ag-Al-Ga	Ag-Al-Gd	Ag-Al-Hf	Ag-Al-Mn	Ag-Al-Mo	Ag-Al-Nb	Ag-Al-Nd
Ag-Al-Ni	Ag-Al-Pb	Ag-Al-Pr	Ag-Al-Pt	Ag-Al-Ru	Ag-Al-Sb	Ag-Al-Sc	Ag-Al-Sm
Ag-Al-Sn	Ag-Al-Sr	Ag-Al-Ta	Ag-Al-Tb	Ag-Al-Ti	Ag-Al-V	Ag-Al-W	Ag-Al-Yb
Ag-Al-Zn	Ag-Au-B	Ag-Au-Ba	Ag-Au-Be	Ag-Au-C	Ag-Au-Ca	Ag-Au-Ce	Ag-Au-Co
Ag-Au-Cr	Ag-Au-Cu	Ag-Au-Dy	Ag-Au-Er	Ag-Au-Fe	Ag-Au-Ga	Ag-Au-Gd	Ag-Au-Ge
Ag-Au-Hf	Ag-Au-La	Ag-Au-Mg	Ag-Au-Mn	Ag-Au-Mo	Ag-Au-Nb	Ag-Au-Nd	Ag-Au-Ni
Ag-Au-P	Ag-Au-Pb	Ag-Au-Pd	Ag-Au-Pr	Ag-Au-Pt	Ag-Au-Ru	Ag-Au-Sb	Ag-Au-Sc
Ag-Au-Si	Ag-Au-Sm	Ag-Au-Sn	Ag-Au-Sr	Ag-Au-Ta	Ag-Au-Tb	Ag-Au-Ti	Ag-Au-V
Ag-Au-W	Ag-Au-Y	Ag-Au-Yb	Ag-Au-Zn	Ag-Au-Zr	Ag-B-Ba	Ag-B-Be	Ag-B-C
Ag-B-Ca	Ag-B-Ce	Ag-B-Co	Ag-B-Cr	Ag-B-Cu	Ag-B-Dy	Ag-B-Er	Ag-B-Fe
Ag-B-Ga	Ag-B-Gd	Ag-B-Ge	Ag-B-Hf	Ag-B-La	Ag-B-Mg	Ag-B-Mn	Ag-B-Mo
Ag-B-Nb	Ag-B-Nd	Ag-B-Ni	Ag-B-P	Ag-B-Pb	Ag-B-Pd	Ag-B-Pr	Ag-B-Pt
Ag-B-Ru	Ag-B-Sb	Ag-B-Sc	Ag-B-Si	Ag-B-Sm	Ag-B-Sn	Ag-B-Sr	Ag-B-Ta
Ag-B-Tb	Ag-B-Ti	Ag-B-V	Ag-B-W	Ag-B-Y	Ag-B-Yb	Ag-B-Zn	Ag-B-Zr
Ag-Ba-Be	Ag-Ba-C	Ag-Ba-Ca	Ag-Ba-Ce	Ag-Ba-Co	Ag-Ba-Cr	Ag-Ba-Cu	Ag-Ba-Dy
Ag-Ba-Er	Ag-Ba-Fe	Ag-Ba-Ga	Ag-Ba-Gd	Ag-Ba-Ge	Ag-Ba-Hf	Ag-Ba-La	Ag-Ba-Mg

Ag-Ba-Mn	Ag-Ba-Mo	Ag-Ba-Nb	Ag-Ba-Nd	Ag-Ba-Ni	Ag-Ba-P	Ag-Ba-Pb	Ag-Ba-Pd
Ag-Ba-Pr	Ag-Ba-Pt	Ag-Ba-Ru	Ag-Ba-Sb	Ag-Ba-Sc	Ag-Ba-Si	Ag-Ba-Sm	Ag-Ba-Sn
Ag-Ba-Sr	Ag-Ba-Ta	Ag-Ba-Tb	Ag-Ba-Ti	Ag-Ba-V	Ag-Ba-W	Ag-Ba-Y	Ag-Ba-Yb
Ag-Ba-Zn	Ag-Ba-Zr	Ag-Be-C	Ag-Be-Ca	Ag-Be-Ce	Ag-Be-Co	Ag-Be-Cr	Ag-Be-Cu
Ag-Be-Dy	Ag-Be-Er	Ag-Be-Fe	Ag-Be-Ga	Ag-Be-Gd	Ag-Be-Ge	Ag-Be-Hf	Ag-Be-La
Ag-Be-Mg	Ag-Be-Mn	Ag-Be-Mo	Ag-Be-Nb	Ag-Be-Nd	Ag-Be-Ni	Ag-Be-P	Ag-Be-Pb
Ag-Be-Pd	Ag-Be-Pr	Ag-Be-Pt	Ag-Be-Ru	Ag-Be-Sb	Ag-Be-Sc	Ag-Be-Si	Ag-Be-Sm
Ag-Be-Sn	Ag-Be-Sr	Ag-Be-Ta	Ag-Be-Tb	Ag-Be-Ti	Ag-Be-V	Ag-Be-W	Ag-Be-Y
Ag-Be-Yb	Ag-Be-Zn	Ag-Be-Zr	Ag-C-Ca	Ag-C-Ce	Ag-C-Co	Ag-C-Cr	Ag-C-Cu
Ag-C-Dy	Ag-C-Er	Ag-C-Fe	Ag-C-Ga	Ag-C-Gd	Ag-C-Ge	Ag-C-Hf	Ag-C-La
Ag-C-Mg	Ag-C-Mn	Ag-C-Mo	Ag-C-Nb	Ag-C-Nd	Ag-C-Ni	Ag-C-P	Ag-C-Pb
Ag-C-Pd	Ag-C-Pr	Ag-C-Pt	Ag-C-Ru	Ag-C-Sb	Ag-C-Sc	Ag-C-Si	Ag-C-Sm
Ag-C-Sn	Ag-C-Sr	Ag-C-Ta	Ag-C-Tb	Ag-C-Ti	Ag-C-V	Ag-C-W	Ag-C-Y
Ag-C-Yb	Ag-C-Zn	Ag-C-Zr	Ag-Ca-Ce	Ag-Ca-Co	Ag-Ca-Cr	Ag-Ca-Dy	Ag-Ca-Er
Ag-Ca-Ga	Ag-Ca-Gd	Ag-Ca-Ge	Ag-Ca-Hf	Ag-Ca-Mn	Ag-Ca-Mo	Ag-Ca-Nb	Ag-Ca-Nd
Ag-Ca-Ni	Ag-Ca-P	Ag-Ca-Pb	Ag-Ca-Pr	Ag-Ca-Pt	Ag-Ca-Ru	Ag-Ca-Sb	Ag-Ca-Sc
Ag-Ca-Si	Ag-Ca-Sm	Ag-Ca-Sn	Ag-Ca-Sr	Ag-Ca-Ta	Ag-Ca-Tb	Ag-Ca-Ti	Ag-Ca-V
Ag-Ca-W	Ag-Ca-Y	Ag-Ca-Yb	Ag-Ca-Zn	Ag-Ca-Zr	Ag-Ce-Co	Ag-Ce-Cr	Ag-Ce-Dy
Ag-Ce-Er	Ag-Ce-Ga	Ag-Ce-Gd	Ag-Ce-Ge	Ag-Ce-Hf	Ag-Ce-La	Ag-Ce-Mn	Ag-Ce-Mo
Ag-Ce-Nb	Ag-Ce-Nd	Ag-Ce-Ni	Ag-Ce-P	Ag-Ce-Pb	Ag-Ce-Pd	Ag-Ce-Pr	Ag-Ce-Pt
Ag-Ce-Ru	Ag-Ce-Sb	Ag-Ce-Sc	Ag-Ce-Si	Ag-Ce-Sm	Ag-Ce-Sn	Ag-Ce-Sr	Ag-Ce-Ta
Ag-Ce-Tb	Ag-Ce-Ti	Ag-Ce-V	Ag-Ce-W	Ag-Ce-Y	Ag-Ce-Zn	Ag-Ce-Zr	Ag-Co-Cr
Ag-Co-Cu	Ag-Co-Dy	Ag-Co-Er	Ag-Co-Fe	Ag-Co-Ga	Ag-Co-Gd	Ag-Co-Ge	Ag-Co-Hf
Ag-Co-La	Ag-Co-Mg	Ag-Co-Mn	Ag-Co-Mo	Ag-Co-Nb	Ag-Co-Nd	Ag-Co-Ni	Ag-Co-P
Ag-Co-Pb	Ag-Co-Pd	Ag-Co-Pr	Ag-Co-Pt	Ag-Co-Ru	Ag-Co-Sb	Ag-Co-Sc	Ag-Co-Si

Ag-Co-Sm	Ag-Co-Sn	Ag-Co-Sr	Ag-Co-Ta	Ag-Co-Tb	Ag-Co-Ti	Ag-Co-V	Ag-Co-W
Ag-Co-Y	Ag-Co-Yb	Ag-Co-Zn	Ag-Co-Zr	Ag-Cr-Cu	Ag-Cr-Dy	Ag-Cr-Er	Ag-Cr-Fe
Ag-Cr-Ga	Ag-Cr-Gd	Ag-Cr-Ge	Ag-Cr-Hf	Ag-Cr-La	Ag-Cr-Mg	Ag-Cr-Mn	Ag-Cr-Mo
Ag-Cr-Nb	Ag-Cr-Nd	Ag-Cr-Ni	Ag-Cr-P	Ag-Cr-Pb	Ag-Cr-Pd	Ag-Cr-Pr	Ag-Cr-Pt
Ag-Cr-Ru	Ag-Cr-Sb	Ag-Cr-Sc	Ag-Cr-Si	Ag-Cr-Sm	Ag-Cr-Sn	Ag-Cr-Sr	Ag-Cr-Ta
Ag-Cr-Tb	Ag-Cr-Ti	Ag-Cr-V	Ag-Cr-W	Ag-Cr-Y	Ag-Cr-Yb	Ag-Cr-Zn	Ag-Cr-Zr
Ag-Cu-Dy	Ag-Cu-Er	Ag-Cu-Ga	Ag-Cu-Gd	Ag-Cu-Hf	Ag-Cu-Mn	Ag-Cu-Mo	Ag-Cu-Nb
Ag-Cu-Nd	Ag-Cu-Ni	Ag-Cu-Pb	Ag-Cu-Pr	Ag-Cu-Pt	Ag-Cu-Ru	Ag-Cu-Sb	Ag-Cu-Sc
Ag-Cu-Sm	Ag-Cu-Sn	Ag-Cu-Sr	Ag-Cu-Ta	Ag-Cu-Tb	Ag-Cu-Ti	Ag-Cu-V	Ag-Cu-W
Ag-Cu-Zn	Ag-Dy-Er	Ag-Dy-Fe	Ag-Dy-Ga	Ag-Dy-Gd	Ag-Dy-Ge	Ag-Dy-Hf	Ag-Dy-La
Ag-Dy-Mg	Ag-Dy-Mn	Ag-Dy-Mo	Ag-Dy-Nb	Ag-Dy-Nd	Ag-Dy-Ni	Ag-Dy-P	Ag-Dy-Pb
Ag-Dy-Pd	Ag-Dy-Pr	Ag-Dy-Pt	Ag-Dy-Ru	Ag-Dy-Sb	Ag-Dy-Sc	Ag-Dy-Si	Ag-Dy-Sm
Ag-Dy-Sn	Ag-Dy-Sr	Ag-Dy-Ta	Ag-Dy-Tb	Ag-Dy-Ti	Ag-Dy-V	Ag-Dy-W	Ag-Dy-Y
Ag-Dy-Yb	Ag-Dy-Zn	Ag-Dy-Zr	Ag-Er-Fe	Ag-Er-Ga	Ag-Er-Gd	Ag-Er-Ge	Ag-Er-Hf
Ag-Er-La	Ag-Er-Mg	Ag-Er-Mn	Ag-Er-Mo	Ag-Er-Nb	Ag-Er-Nd	Ag-Er-Ni	Ag-Er-P
Ag-Er-Pb	Ag-Er-Pd	Ag-Er-Pr	Ag-Er-Pt	Ag-Er-Ru	Ag-Er-Sb	Ag-Er-Sc	Ag-Er-Si
Ag-Er-Sm	Ag-Er-Sn	Ag-Er-Sr	Ag-Er-Ta	Ag-Er-Tb	Ag-Er-Ti	Ag-Er-V	Ag-Er-W
Ag-Er-Y	Ag-Er-Yb	Ag-Er-Zn	Ag-Er-Zr	Ag-Fe-Ga	Ag-Fe-Gd	Ag-Fe-Hf	Ag-Fe-Mg
Ag-Fe-Mn	Ag-Fe-Mo	Ag-Fe-Nb	Ag-Fe-Nd	Ag-Fe-Ni	Ag-Fe-Pb	Ag-Fe-Pr	Ag-Fe-Pt
Ag-Fe-Ru	Ag-Fe-Sb	Ag-Fe-Sc	Ag-Fe-Sm	Ag-Fe-Sn	Ag-Fe-Sr	Ag-Fe-Ta	Ag-Fe-Tb
Ag-Fe-Ti	Ag-Fe-V	Ag-Fe-W	Ag-Fe-Yb	Ag-Fe-Zn	Ag-Ga-Gd	Ag-Ga-Ge	Ag-Ga-Hf
Ag-Ga-La	Ag-Ga-Mg	Ag-Ga-Mn	Ag-Ga-Mo	Ag-Ga-Nb	Ag-Ga-Nd	Ag-Ga-Ni	Ag-Ga-P
Ag-Ga-Pb	Ag-Ga-Pd	Ag-Ga-Pr	Ag-Ga-Pt	Ag-Ga-Ru	Ag-Ga-Sb	Ag-Ga-Sc	Ag-Ga-Si
Ag-Ga-Sm	Ag-Ga-Sn	Ag-Ga-Sr	Ag-Ga-Ta	Ag-Ga-Tb	Ag-Ga-Ti	Ag-Ga-V	Ag-Ga-W
Ag-Ga-Y	Ag-Ga-Yb	Ag-Ga-Zn	Ag-Ga-Zr	Ag-Gd-Ge	Ag-Gd-Hf	Ag-Gd-La	Ag-Gd-Mg

Ag-Gd-Mn	Ag-Gd-Mo	Ag-Gd-Nb	Ag-Gd-Nd	Ag-Gd-Ni	Ag-Gd-P	Ag-Gd-Pb	Ag-Gd-Pd
Ag-Gd-Pr	Ag-Gd-Pt	Ag-Gd-Ru	Ag-Gd-Sb	Ag-Gd-Sc	Ag-Gd-Si	Ag-Gd-Sm	Ag-Gd-Sn
Ag-Gd-Sr	Ag-Gd-Ta	Ag-Gd-Tb	Ag-Gd-Ti	Ag-Gd-V	Ag-Gd-W	Ag-Gd-Y	Ag-Gd-Yb
Ag-Gd-Zn	Ag-Gd-Zr	Ag-Ge-Hf	Ag-Ge-La	Ag-Ge-Mg	Ag-Ge-Mn	Ag-Ge-Mo	Ag-Ge-Nb
Ag-Ge-Nd	Ag-Ge-Ni	Ag-Ge-P	Ag-Ge-Pb	Ag-Ge-Pr	Ag-Ge-Pt	Ag-Ge-Ru	Ag-Ge-Sb
Ag-Ge-Sc	Ag-Ge-Sm	Ag-Ge-Sn	Ag-Ge-Sr	Ag-Ge-Ta	Ag-Ge-Tb	Ag-Ge-Ti	Ag-Ge-V
Ag-Ge-W	Ag-Ge-Y	Ag-Ge-Yb	Ag-Ge-Zn	Ag-Hf-La	Ag-Hf-Mg	Ag-Hf-Mn	Ag-Hf-Mo
Ag-Hf-Nb	Ag-Hf-Nd	Ag-Hf-Ni	Ag-Hf-P	Ag-Hf-Pb	Ag-Hf-Pd	Ag-Hf-Pr	Ag-Hf-Pt
Ag-Hf-Ru	Ag-Hf-Sb	Ag-Hf-Sc	Ag-Hf-Si	Ag-Hf-Sm	Ag-Hf-Sn	Ag-Hf-Sr	Ag-Hf-Ta
Ag-Hf-Tb	Ag-Hf-Ti	Ag-Hf-V	Ag-Hf-W	Ag-Hf-Y	Ag-Hf-Yb	Ag-Hf-Zn	Ag-Hf-Zr
Ag-La-Mn	Ag-La-Mo	Ag-La-Nb	Ag-La-Nd	Ag-La-Ni	Ag-La-P	Ag-La-Pb	Ag-La-Pd
Ag-La-Pr	Ag-La-Pt	Ag-La-Ru	Ag-La-Sb	Ag-La-Sc	Ag-La-Si	Ag-La-Sm	Ag-La-Sn
Ag-La-Sr	Ag-La-Ta	Ag-La-Tb	Ag-La-Ti	Ag-La-V	Ag-La-W	Ag-La-Y	Ag-La-Yb
Ag-La-Zn	Ag-Mg-Mn	Ag-Mg-Mo	Ag-Mg-Nb	Ag-Mg-Nd	Ag-Mg-Ni	Ag-Mg-P	Ag-Mg-Pb
Ag-Mg-Pr	Ag-Mg-Pt	Ag-Mg-Ru	Ag-Mg-Sb	Ag-Mg-Sc	Ag-Mg-Si	Ag-Mg-Sm	Ag-Mg-Sn
Ag-Mg-Sr	Ag-Mg-Ta	Ag-Mg-Tb	Ag-Mg-Ti	Ag-Mg-V	Ag-Mg-W	Ag-Mg-Zn	Ag-Mg-Zr
Ag-Mn-Mo	Ag-Mn-Nb	Ag-Mn-Nd	Ag-Mn-Ni	Ag-Mn-P	Ag-Mn-Pb	Ag-Mn-Pd	Ag-Mn-Pr
Ag-Mn-Pt	Ag-Mn-Ru	Ag-Mn-Sb	Ag-Mn-Sc	Ag-Mn-Si	Ag-Mn-Sm	Ag-Mn-Sn	Ag-Mn-Sr
Ag-Mn-Ta	Ag-Mn-Tb	Ag-Mn-Ti	Ag-Mn-V	Ag-Mn-W	Ag-Mn-Y	Ag-Mn-Yb	Ag-Mn-Zn
Ag-Mn-Zr	Ag-Mo-Nb	Ag-Mo-Nd	Ag-Mo-Ni	Ag-Mo-P	Ag-Mo-Pb	Ag-Mo-Pd	Ag-Mo-Pr
Ag-Mo-Pt	Ag-Mo-Ru	Ag-Mo-Sb	Ag-Mo-Sc	Ag-Mo-Si	Ag-Mo-Sm	Ag-Mo-Sn	Ag-Mo-Sr
Ag-Mo-Ta	Ag-Mo-Tb	Ag-Mo-Ti	Ag-Mo-V	Ag-Mo-W	Ag-Mo-Y	Ag-Mo-Yb	Ag-Mo-Zn
Ag-Mo-Zr	Ag-Nb-Nd	Ag-Nb-Ni	Ag-Nb-P	Ag-Nb-Pb	Ag-Nb-Pd	Ag-Nb-Pr	Ag-Nb-Pt
Ag-Nb-Ru	Ag-Nb-Sb	Ag-Nb-Sc	Ag-Nb-Si	Ag-Nb-Sm	Ag-Nb-Sn	Ag-Nb-Sr	Ag-Nb-Ta
Ag-Nb-Tb	Ag-Nb-Ti	Ag-Nb-V	Ag-Nb-W	Ag-Nb-Y	Ag-Nb-Yb	Ag-Nb-Zn	Ag-Nb-Zr

Ag-Nd-Ni	Ag-Nd-P	Ag-Nd-Pb	Ag-Nd-Pd	Ag-Nd-Pr	Ag-Nd-Pt	Ag-Nd-Ru	Ag-Nd-Sb
Ag-Nd-Sc	Ag-Nd-Si	Ag-Nd-Sm	Ag-Nd-Sn	Ag-Nd-Sr	Ag-Nd-Ta	Ag-Nd-Tb	Ag-Nd-Ti
Ag-Nd-V	Ag-Nd-W	Ag-Nd-Y	Ag-Nd-Yb	Ag-Nd-Zn	Ag-Nd-Zr	Ag-Ni-P	Ag-Ni-Pb
Ag-Ni-Pd	Ag-Ni-Pr	Ag-Ni-Pt	Ag-Ni-Ru	Ag-Ni-Sb	Ag-Ni-Sc	Ag-Ni-Si	Ag-Ni-Sm
Ag-Ni-Sn	Ag-Ni-Sr	Ag-Ni-Ta	Ag-Ni-Tb	Ag-Ni-Ti	Ag-Ni-V	Ag-Ni-W	Ag-Ni-Y
Ag-Ni-Yb	Ag-Ni-Zn	Ag-Ni-Zr	Ag-P-Pb	Ag-P-Pr	Ag-P-Pt	Ag-P-Ru	Ag-P-Sb
Ag-P-Sc	Ag-P-Sm	Ag-P-Sn	Ag-P-Sr	Ag-P-Ta	Ag-P-Tb	Ag-P-Ti	Ag-P-V
Ag-P-W	Ag-P-Y	Ag-P-Yb	Ag-P-Zn	Ag-Pb-Pd	Ag-Pb-Pr	Ag-Pb-Pt	Ag-Pb-Ru
Ag-Pb-Sb	Ag-Pb-Sc	Ag-Pb-Si	Ag-Pb-Sm	Ag-Pb-Sn	Ag-Pb-Sr	Ag-Pb-Ta	Ag-Pb-Tb
Ag-Pb-Ti	Ag-Pb-V	Ag-Pb-W	Ag-Pb-Y	Ag-Pb-Yb	Ag-Pb-Zn	Ag-Pb-Zr	Ag-Pd-Pr
Ag-Pd-Pt	Ag-Pd-Ru	Ag-Pd-Sb	Ag-Pd-Sc	Ag-Pd-Sm	Ag-Pd-Sn	Ag-Pd-Sr	Ag-Pd-Ta
Ag-Pd-Tb	Ag-Pd-Ti	Ag-Pd-V	Ag-Pd-W	Ag-Pd-Y	Ag-Pd-Zn	Ag-Pr-Pt	Ag-Pr-Ru
Ag-Pr-Sb	Ag-Pr-Sc	Ag-Pr-Si	Ag-Pr-Sm	Ag-Pr-Sn	Ag-Pr-Sr	Ag-Pr-Ta	Ag-Pr-Tb
Ag-Pr-Ti	Ag-Pr-V	Ag-Pr-W	Ag-Pr-Y	Ag-Pr-Yb	Ag-Pr-Zn	Ag-Pr-Zr	Ag-Pt-Ru
Ag-Pt-Sb	Ag-Pt-Sc	Ag-Pt-Si	Ag-Pt-Sm	Ag-Pt-Sn	Ag-Pt-Sr	Ag-Pt-Ta	Ag-Pt-Tb
Ag-Pt-Ti	Ag-Pt-V	Ag-Pt-W	Ag-Pt-Y	Ag-Pt-Yb	Ag-Pt-Zn	Ag-Pt-Zr	Ag-Ru-Sb
Ag-Ru-Sc	Ag-Ru-Si	Ag-Ru-Sm	Ag-Ru-Sn	Ag-Ru-Sr	Ag-Ru-Ta	Ag-Ru-Tb	Ag-Ru-Ti
Ag-Ru-V	Ag-Ru-W	Ag-Ru-Y	Ag-Ru-Yb	Ag-Ru-Zn	Ag-Ru-Zr	Ag-Sb-Sc	Ag-Sb-Si
Ag-Sb-Sm	Ag-Sb-Sn	Ag-Sb-Sr	Ag-Sb-Ta	Ag-Sb-Tb	Ag-Sb-Ti	Ag-Sb-V	Ag-Sb-W
Ag-Sb-Y	Ag-Sb-Yb	Ag-Sb-Zn	Ag-Sb-Zr	Ag-Sc-Si	Ag-Sc-Sm	Ag-Sc-Sn	Ag-Sc-Sr
Ag-Sc-Ta	Ag-Sc-Tb	Ag-Sc-Ti	Ag-Sc-V	Ag-Sc-W	Ag-Sc-Y	Ag-Sc-Yb	Ag-Sc-Zn
Ag-Sc-Zr	Ag-Si-Sm	Ag-Si-Sn	Ag-Si-Sr	Ag-Si-Ta	Ag-Si-Tb	Ag-Si-Ti	Ag-Si-V
Ag-Si-W	Ag-Si-Y	Ag-Si-Yb	Ag-Si-Zn	Ag-Sm-Sn	Ag-Sm-Sr	Ag-Sm-Ta	Ag-Sm-Tb
Ag-Sm-Ti	Ag-Sm-V	Ag-Sm-W	Ag-Sm-Y	Ag-Sm-Yb	Ag-Sm-Zn	Ag-Sm-Zr	Ag-Sn-Sr
Ag-Sn-Ta	Ag-Sn-Tb	Ag-Sn-Ti	Ag-Sn-V	Ag-Sn-W	Ag-Sn-Y	Ag-Sn-Yb	Ag-Sn-Zn

Ag-Sn-Zr	Ag-Sr-Ta	Ag-Sr-Tb	Ag-Sr-Ti	Ag-Sr-V	Ag-Sr-W	Ag-Sr-Y	Ag-Sr-Yb
Ag-Sr-Zn	Ag-Sr-Zr	Ag-Ta-Tb	Ag-Ta-Ti	Ag-Ta-V	Ag-Ta-W	Ag-Ta-Y	Ag-Ta-Yb
Ag-Ta-Zn	Ag-Ta-Zr	Ag-Tb-Ti	Ag-Tb-V	Ag-Tb-W	Ag-Tb-Y	Ag-Tb-Yb	Ag-Tb-Zn
Ag-Tb-Zr	Ag-Ti-V	Ag-Ti-W	Ag-Ti-Y	Ag-Ti-Yb	Ag-Ti-Zn	Ag-Ti-Zr	Ag-V-W
Ag-V-Y	Ag-V-Yb	Ag-V-Zn	Ag-V-Zr	Ag-W-Y	Ag-W-Yb	Ag-W-Zn	Ag-W-Zr
Ag-Y-Yb	Ag-Y-Zn	Ag-Y-Zr	Ag-Yb-Zn	Ag-Yb-Zr	Ag-Zn-Zr	Al-Au-Ba	Al-Au-Be
Al-Au-C	Al-Au-Ca	Al-Au-Ce	Al-Au-Co	Al-Au-Cr	Al-Au-Dy	Al-Au-Ga	Al-Au-Hf
Al-Au-Mn	Al-Au-Mo	Al-Au-Nb	Al-Au-Nd	Al-Au-Ni	Al-Au-P	Al-Au-Pb	Al-Au-Pt
Al-Au-Ru	Al-Au-Sb	Al-Au-Sc	Al-Au-Sm	Al-Au-Sn	Al-Au-Sr	Al-Au-Ta	Al-Au-Ti
Al-Au-V	Al-Au-W	Al-Au-Y	Al-Au-Yb	Al-Au-Zr	Al-B-Ba	Al-B-C	Al-B-Ca
Al-B-Ce	Al-B-Dy	Al-B-Mg	Al-B-Pb	Al-B-Pt	Al-B-Ru	Al-B-Sb	Al-B-Sc
Al-B-Sn	Al-B-Sr	Al-B-Ta	Al-B-W	Al-B-Yb	Al-B-Zn	Al-Ba-Be	Al-Ba-C
Al-Ba-Ca	Al-Ba-Ce	Al-Ba-Co	Al-Ba-Cr	Al-Ba-Cu	Al-Ba-Dy	Al-Ba-Er	Al-Ba-Fe
Al-Ba-Ga	Al-Ba-Gd	Al-Ba-Ge	Al-Ba-Hf	Al-Ba-La	Al-Ba-Mg	Al-Ba-Mn	Al-Ba-Mo
Al-Ba-Nb	Al-Ba-Nd	Al-Ba-Ni	Al-Ba-P	Al-Ba-Pb	Al-Ba-Pd	Al-Ba-Pr	Al-Ba-Pt
Al-Ba-Ru	Al-Ba-Sb	Al-Ba-Sc	Al-Ba-Si	Al-Ba-Sm	Al-Ba-Sn	Al-Ba-Sr	Al-Ba-Ta
Al-Ba-Tb	Al-Ba-Ti	Al-Ba-V	Al-Ba-W	Al-Ba-Y	Al-Ba-Yb	Al-Ba-Zn	Al-Ba-Zr
Al-Be-C	Al-Be-Ca	Al-Be-Ce	Al-Be-Co	Al-Be-Cr	Al-Be-Dy	Al-Be-Er	Al-Be-Ga
Al-Be-Gd	Al-Be-Ge	Al-Be-La	Al-Be-Mg	Al-Be-Mn	Al-Be-Mo	Al-Be-Nd	Al-Be-Ni
Al-Be-P	Al-Be-Pb	Al-Be-Pd	Al-Be-Pr	Al-Be-Pt	Al-Be-Ru	Al-Be-Sb	Al-Be-Sc
Al-Be-Sm	Al-Be-Sn	Al-Be-Sr	Al-Be-Ta	Al-Be-Tb	Al-Be-V	Al-Be-W	Al-Be-Y
Al-Be-Yb	Al-Be-Zn	Al-C-Ca	Al-C-Ce	Al-C-Co	Al-C-Cr	Al-C-Cu	Al-C-Dy
Al-C-Er	Al-C-Fe	Al-C-Ga	Al-C-Gd	Al-C-Ge	Al-C-Hf	Al-C-La	Al-C-Mg
Al-C-Mn	Al-C-Mo	Al-C-Nb	Al-C-Nd	Al-C-Ni	Al-C-P	Al-C-Pb	Al-C-Pd
Al-C-Pr	Al-C-Pt	Al-C-Ru	Al-C-Sb	Al-C-Sc	Al-C-Si	Al-C-Sm	Al-C-Sn

Al-C-Sr	Al-C-Ta	Al-C-Tb	Al-C-Ti	Al-C-V	Al-C-W	Al-C-Y	Al-C-Yb
Al-C-Zn	Al-C-Zr	Al-Ca-Ce	Al-Ca-Cr	Al-Ca-Dy	Al-Ca-Er	Al-Ca-Gd	Al-Ca-Ge
Al-Ca-Hf	Al-Ca-Mn	Al-Ca-Mo	Al-Ca-Nb	Al-Ca-Nd	Al-Ca-P	Al-Ca-Pb	Al-Ca-Pr
Al-Ca-Pt	Al-Ca-Ru	Al-Ca-Sb	Al-Ca-Sc	Al-Ca-Si	Al-Ca-Sm	Al-Ca-Sn	Al-Ca-Sr
Al-Ca-Ta	Al-Ca-Tb	Al-Ca-Ti	Al-Ca-V	Al-Ca-W	Al-Ca-Y	Al-Ca-Yb	Al-Ca-Zr
Al-Ce-Dy	Al-Ce-Er	Al-Ce-Ga	Al-Ce-Gd	Al-Ce-Ge	Al-Ce-Hf	Al-Ce-La	Al-Ce-Mo
Al-Ce-Nd	Al-Ce-P	Al-Ce-Pb	Al-Ce-Pd	Al-Ce-Pr	Al-Ce-Pt	Al-Ce-Ru	Al-Ce-Sb
Al-Ce-Sc	Al-Ce-Si	Al-Ce-Sm	Al-Ce-Sn	Al-Ce-Sr	Al-Ce-Ta	Al-Ce-Tb	Al-Ce-Ti
Al-Ce-W	Al-Ce-Y	Al-Ce-Yb	Al-Ce-Zr	Al-Co-Cu	Al-Co-Ga	Al-Co-Mg	Al-Co-Pb
Al-Co-Pt	Al-Co-Ru	Al-Co-Sb	Al-Co-Sc	Al-Co-Sn	Al-Co-Sr	Al-Co-Ta	Al-Co-W
Al-Co-Yb	Al-Co-Zn	Al-Cr-Cu	Al-Cr-Dy	Al-Cr-Er	Al-Cr-Ga	Al-Cr-Gd	Al-Cr-Hf
Al-Cr-Mg	Al-Cr-Mn	Al-Cr-Nb	Al-Cr-Nd	Al-Cr-Pb	Al-Cr-Pr	Al-Cr-Pt	Al-Cr-Ru
Al-Cr-Sb	Al-Cr-Sc	Al-Cr-Sm	Al-Cr-Sn	Al-Cr-Sr	Al-Cr-Ta	Al-Cr-Tb	Al-Cr-Ti
Al-Cr-V	Al-Cr-W	Al-Cr-Y	Al-Cr-Yb	Al-Cr-Zn	Al-Cu-Mn	Al-Cu-Mo	Al-Cu-Pb
Al-Cu-Pt	Al-Cu-Ru	Al-Cu-Sb	Al-Cu-Sc	Al-Cu-Sm	Al-Cu-Sn	Al-Cu-Sr	Al-Cu-Ta
Al-Cu-W	Al-Cu-Yb	Al-Dy-Er	Al-Dy-Ga	Al-Dy-Gd	Al-Dy-Ge	Al-Dy-Hf	Al-Dy-La
Al-Dy-Mn	Al-Dy-Mo	Al-Dy-Nb	Al-Dy-Nd	Al-Dy-Ni	Al-Dy-P	Al-Dy-Pb	Al-Dy-Pd
Al-Dy-Pr	Al-Dy-Pt	Al-Dy-Ru	Al-Dy-Sb	Al-Dy-Sc	Al-Dy-Si	Al-Dy-Sm	Al-Dy-Sn
Al-Dy-Sr	Al-Dy-Ta	Al-Dy-Tb	Al-Dy-Ti	Al-Dy-V	Al-Dy-W	Al-Dy-Y	Al-Dy-Yb
Al-Dy-Zn	Al-Er-Fe	Al-Er-Ga	Al-Er-Gd	Al-Er-Ge	Al-Er-Hf	Al-Er-La	Al-Er-Mn
Al-Er-Mo	Al-Er-Nb	Al-Er-Nd	Al-Er-P	Al-Er-Pb	Al-Er-Pd	Al-Er-Pr	Al-Er-Pt
Al-Er-Ru	Al-Er-Sb	Al-Er-Sc	Al-Er-Si	Al-Er-Sm	Al-Er-Sn	Al-Er-Sr	Al-Er-Ta
Al-Er-Tb	Al-Er-Ti	Al-Er-V	Al-Er-W	Al-Er-Y	Al-Er-Yb	Al-Er-Zn	Al-Er-Zr
Al-Fe-Mg	Al-Fe-Pb	Al-Fe-Pt	Al-Fe-Ru	Al-Fe-Sb	Al-Fe-Sc	Al-Fe-Sn	Al-Fe-Sr
Al-Fe-Ta	Al-Fe-W	Al-Fe-Yb	Al-Fe-Zn	Al-Ga-Ge	Al-Ga-Hf	Al-Ga-La	Al-Ga-Mn

Al-Ga-Mo	Al-Ga-Nb	Al-Ga-Nd	Al-Ga-Ni	Al-Ga-P	Al-Ga-Pb	Al-Ga-Pd	Al-Ga-Pr
Al-Ga-Pt	Al-Ga-Ru	Al-Ga-Sb	Al-Ga-Sc	Al-Ga-Si	Al-Ga-Sm	Al-Ga-Sn	Al-Ga-Sr
Al-Ga-Ta	Al-Ga-Tb	Al-Ga-Ti	Al-Ga-V	Al-Ga-W	Al-Ga-Y	Al-Ga-Yb	Al-Ga-Zn
Al-Gd-Ge	Al-Gd-Hf	Al-Gd-La	Al-Gd-Mn	Al-Gd-Nb	Al-Gd-Nd	Al-Gd-P	Al-Gd-Pb
Al-Gd-Pd	Al-Gd-Pr	Al-Gd-Pt	Al-Gd-Ru	Al-Gd-Sb	Al-Gd-Sc	Al-Gd-Si	Al-Gd-Sm
Al-Gd-Sn	Al-Gd-Sr	Al-Gd-Ta	Al-Gd-Tb	Al-Gd-Ti	Al-Gd-V	Al-Gd-W	Al-Gd-Y
Al-Gd-Yb	Al-Gd-Zn	Al-Ge-Hf	Al-Ge-La	Al-Ge-Mg	Al-Ge-Nd	Al-Ge-P	Al-Ge-Pb
Al-Ge-Pr	Al-Ge-Pt	Al-Ge-Ru	Al-Ge-Sb	Al-Ge-Sc	Al-Ge-Sm	Al-Ge-Sn	Al-Ge-Sr
Al-Ge-Ta	Al-Ge-Tb	Al-Ge-W	Al-Ge-Y	Al-Ge-Yb	Al-Ge-Zn	Al-Hf-La	Al-Hf-Mg
Al-Hf-Mn	Al-Hf-Mo	Al-Hf-Nd	Al-Hf-Pb	Al-Hf-Pd	Al-Hf-Pr	Al-Hf-Pt	Al-Hf-Ru
Al-Hf-Sb	Al-Hf-Sc	Al-Hf-Sm	Al-Hf-Sn	Al-Hf-Sr	Al-Hf-Ta	Al-Hf-Tb	Al-Hf-W
Al-Hf-Y	Al-Hf-Yb	Al-Hf-Zn	Al-La-Nd	Al-La-P	Al-La-Pb	Al-La-Pd	Al-La-Pr
Al-La-Ru	Al-La-Sb	Al-La-Sc	Al-La-Si	Al-La-Sm	Al-La-Sn	Al-La-Sr	Al-La-Ta
Al-La-Tb	Al-La-Ti	Al-La-V	Al-La-W	Al-La-Y	Al-La-Yb	Al-Mg-Mo	Al-Mg-Nb
Al-Mg-P	Al-Mg-Pb	Al-Mg-Pt	Al-Mg-Ru	Al-Mg-Sb	Al-Mg-Sc	Al-Mg-Si	Al-Mg-Sm
Al-Mg-Sn	Al-Mg-Sr	Al-Mg-Ta	Al-Mg-V	Al-Mg-W	Al-Mg-Yb	Al-Mg-Zr	Al-Mn-Mo
Al-Mn-Nd	Al-Mn-Pb	Al-Mn-Pr	Al-Mn-Pt	Al-Mn-Ru	Al-Mn-Sb	Al-Mn-Sc	Al-Mn-Sm
Al-Mn-Sn	Al-Mn-Sr	Al-Mn-Ta	Al-Mn-Tb	Al-Mn-Ti	Al-Mn-V	Al-Mn-W	Al-Mn-Yb
Al-Mn-Zn	Al-Mo-Nd	Al-Mo-Pb	Al-Mo-Pd	Al-Mo-Pr	Al-Mo-Pt	Al-Mo-Ru	Al-Mo-Sb
Al-Mo-Sc	Al-Mo-Sm	Al-Mo-Sn	Al-Mo-Sr	Al-Mo-Ta	Al-Mo-Tb	Al-Mo-Ti	Al-Mo-V
Al-Mo-W	Al-Mo-Y	Al-Mo-Yb	Al-Mo-Zn	Al-Nb-Nd	Al-Nb-P	Al-Nb-Pb	Al-Nb-Pr
Al-Nb-Ru	Al-Nb-Sb	Al-Nb-Sc	Al-Nb-Sm	Al-Nb-Sn	Al-Nb-Sr	Al-Nb-Ta	Al-Nb-Tb
Al-Nb-W	Al-Nb-Y	Al-Nb-Yb	Al-Nb-Zn	Al-Nd-P	Al-Nd-Pb	Al-Nd-Pd	Al-Nd-Pr
Al-Nd-Pt	Al-Nd-Ru	Al-Nd-Sb	Al-Nd-Sc	Al-Nd-Si	Al-Nd-Sm	Al-Nd-Sn	Al-Nd-Sr
Al-Nd-Ta	Al-Nd-Tb	Al-Nd-Ti	Al-Nd-V	Al-Nd-W	Al-Nd-Y	Al-Nd-Yb	Al-Nd-Zn

Al-Nd-Zr	Al-Ni-Pb	Al-Ni-Ru	Al-Ni-Sb	Al-Ni-Sc	Al-Ni-Sn	Al-Ni-Sr	Al-Ni-Ta
Al-Ni-Tb	Al-Ni-W	Al-Ni-Yb	Al-Ni-Zn	Al-P-Pb	Al-P-Pr	Al-P-Ru	Al-P-Sb
Al-P-Sc	Al-P-Sm	Al-P-Sn	Al-P-Sr	Al-P-Ta	Al-P-Tb	Al-P-W	Al-P-Y
Al-P-Yb	Al-P-Zn	Al-Pb-Pd	Al-Pb-Pr	Al-Pb-Pt	Al-Pb-Ru	Al-Pb-Sb	Al-Pb-Sc
Al-Pb-Si	Al-Pb-Sm	Al-Pb-Sn	Al-Pb-Sr	Al-Pb-Ta	Al-Pb-Tb	Al-Pb-Ti	Al-Pb-V
Al-Pb-W	Al-Pb-Y	Al-Pb-Yb	Al-Pb-Zn	Al-Pb-Zr	Al-Pd-Pr	Al-Pd-Pt	Al-Pd-Ru
Al-Pd-Sb	Al-Pd-Sc	Al-Pd-Sm	Al-Pd-Sn	Al-Pd-Sr	Al-Pd-Ta	Al-Pd-Tb	Al-Pd-V
Al-Pd-W	Al-Pd-Y	Al-Pd-Yb	Al-Pd-Zn	Al-Pr-Pt	Al-Pr-Ru	Al-Pr-Sb	Al-Pr-Sc
Al-Pr-Si	Al-Pr-Sm	Al-Pr-Sn	Al-Pr-Sr	Al-Pr-Ta	Al-Pr-Tb	Al-Pr-Ti	Al-Pr-V
Al-Pr-W	Al-Pr-Y	Al-Pr-Yb	Al-Pr-Zn	Al-Pt-Ru	Al-Pt-Sb	Al-Pt-Sc	Al-Pt-Si
Al-Pt-Sm	Al-Pt-Sn	Al-Pt-Sr	Al-Pt-Ta	Al-Pt-Tb	Al-Pt-Ti	Al-Pt-V	Al-Pt-W
Al-Pt-Y	Al-Pt-Yb	Al-Pt-Zn	Al-Pt-Zr	Al-Ru-Sb	Al-Ru-Sc	Al-Ru-Si	Al-Ru-Sm
Al-Ru-Sn	Al-Ru-Sr	Al-Ru-Ta	Al-Ru-Tb	Al-Ru-Ti	Al-Ru-V	Al-Ru-W	Al-Ru-Y
Al-Ru-Yb	Al-Ru-Zn	Al-Ru-Zr	Al-Sb-Sc	Al-Sb-Si	Al-Sb-Sm	Al-Sb-Sn	Al-Sb-Sr
Al-Sb-Ta	Al-Sb-Tb	Al-Sb-Ti	Al-Sb-V	Al-Sb-W	Al-Sb-Y	Al-Sb-Yb	Al-Sb-Zn
Al-Sb-Zr	Al-Sc-Si	Al-Sc-Sm	Al-Sc-Sn	Al-Sc-Sr	Al-Sc-Ta	Al-Sc-Tb	Al-Sc-Ti
Al-Sc-V	Al-Sc-W	Al-Sc-Y	Al-Sc-Yb	Al-Sc-Zn	Al-Sc-Zr	Al-Si-Sm	Al-Si-Sn
Al-Si-Sr	Al-Si-Ta	Al-Si-W	Al-Si-Y	Al-Si-Yb	Al-Si-Zn	Al-Sm-Sn	Al-Sm-Sr
Al-Sm-Ta	Al-Sm-Tb	Al-Sm-Ti	Al-Sm-V	Al-Sm-W	Al-Sm-Y	Al-Sm-Yb	Al-Sm-Zn
Al-Sm-Zr	Al-Sn-Sr	Al-Sn-Ta	Al-Sn-Tb	Al-Sn-Ti	Al-Sn-V	Al-Sn-W	Al-Sn-Y
Al-Sn-Yb	Al-Sn-Zn	Al-Sn-Zr	Al-Sr-Ta	Al-Sr-Tb	Al-Sr-Ti	Al-Sr-V	Al-Sr-W
Al-Sr-Y	Al-Sr-Yb	Al-Sr-Zn	Al-Sr-Zr	Al-Ta-Tb	Al-Ta-Ti	Al-Ta-V	Al-Ta-W
Al-Ta-Y	Al-Ta-Yb	Al-Ta-Zn	Al-Ta-Zr	Al-Tb-Ti	Al-Tb-V	Al-Tb-W	Al-Tb-Y
Al-Tb-Yb	Al-Tb-Zn	Al-Tb-Zr	Al-Ti-W	Al-Ti-Y	Al-Ti-Yb	Al-Ti-Zn	Al-V-W
Al-V-Y	Al-V-Yb	Al-V-Zn	Al-W-Y	Al-W-Yb	Al-W-Zn	Al-W-Zr	Al-Y-Yb

Al-Y-Zn	Al-Y-Zr	Al-Yb-Zn	Al-Yb-Zr	Al-Zn-Zr	Au-B-Ba	Au-B-Be	Au-B-C
Au-B-Ca	Au-B-Ce	Au-B-Co	Au-B-Cr	Au-B-Dy	Au-B-Ga	Au-B-Hf	Au-B-Mg
Au-B-Mn	Au-B-Mo	Au-B-Nb	Au-B-Nd	Au-B-Ni	Au-B-P	Au-B-Pb	Au-B-Pt
Au-B-Ru	Au-B-Sb	Au-B-Sc	Au-B-Sm	Au-B-Sn	Au-B-Sr	Au-B-Ta	Au-B-Ti
Au-B-V	Au-B-W	Au-B-Y	Au-B-Yb	Au-B-Zn	Au-B-Zr	Au-Ba-Be	Au-Ba-C
Au-Ba-Ca	Au-Ba-Ce	Au-Ba-Co	Au-Ba-Cr	Au-Ba-Cu	Au-Ba-Dy	Au-Ba-Er	Au-Ba-Fe
Au-Ba-Ga	Au-Ba-Gd	Au-Ba-Ge	Au-Ba-Hf	Au-Ba-La	Au-Ba-Mg	Au-Ba-Mn	Au-Ba-Mo
Au-Ba-Nb	Au-Ba-Nd	Au-Ba-Ni	Au-Ba-P	Au-Ba-Pb	Au-Ba-Pd	Au-Ba-Pr	Au-Ba-Pt
Au-Ba-Ru	Au-Ba-Sb	Au-Ba-Sc	Au-Ba-Si	Au-Ba-Sm	Au-Ba-Sn	Au-Ba-Sr	Au-Ba-Ta
Au-Ba-Tb	Au-Ba-Ti	Au-Ba-V	Au-Ba-W	Au-Ba-Y	Au-Ba-Yb	Au-Ba-Zn	Au-Ba-Zr
Au-Be-C	Au-Be-Ca	Au-Be-Ce	Au-Be-Co	Au-Be-Cr	Au-Be-Cu	Au-Be-Dy	Au-Be-Er
Au-Be-Fe	Au-Be-Ga	Au-Be-Gd	Au-Be-Ge	Au-Be-Hf	Au-Be-La	Au-Be-Mg	Au-Be-Mn
Au-Be-Mo	Au-Be-Nb	Au-Be-Nd	Au-Be-Ni	Au-Be-P	Au-Be-Pb	Au-Be-Pd	Au-Be-Pr
Au-Be-Pt	Au-Be-Ru	Au-Be-Sb	Au-Be-Sc	Au-Be-Si	Au-Be-Sm	Au-Be-Sn	Au-Be-Sr
Au-Be-Ta	Au-Be-Tb	Au-Be-Ti	Au-Be-V	Au-Be-W	Au-Be-Y	Au-Be-Yb	Au-Be-Zn
Au-Be-Zr	Au-C-Ca	Au-C-Ce	Au-C-Co	Au-C-Cr	Au-C-Cu	Au-C-Dy	Au-C-Er
Au-C-Fe	Au-C-Ga	Au-C-Gd	Au-C-Ge	Au-C-Hf	Au-C-La	Au-C-Mg	Au-C-Mn
Au-C-Mo	Au-C-Nb	Au-C-Nd	Au-C-Ni	Au-C-P	Au-C-Pb	Au-C-Pd	Au-C-Pr
Au-C-Pt	Au-C-Ru	Au-C-Sb	Au-C-Sc	Au-C-Si	Au-C-Sm	Au-C-Sn	Au-C-Sr
Au-C-Ta	Au-C-Tb	Au-C-Ti	Au-C-V	Au-C-W	Au-C-Y	Au-C-Yb	Au-C-Zn
Au-C-Zr	Au-Ca-Ce	Au-Ca-Co	Au-Ca-Cr	Au-Ca-Cu	Au-Ca-Dy	Au-Ca-Er	Au-Ca-Fe
Au-Ca-Ga	Au-Ca-Gd	Au-Ca-Ge	Au-Ca-Hf	Au-Ca-La	Au-Ca-Mg	Au-Ca-Mn	Au-Ca-Mo
Au-Ca-Nb	Au-Ca-Nd	Au-Ca-Ni	Au-Ca-P	Au-Ca-Pb	Au-Ca-Pd	Au-Ca-Pr	Au-Ca-Pt
Au-Ca-Ru	Au-Ca-Sb	Au-Ca-Sc	Au-Ca-Si	Au-Ca-Sm	Au-Ca-Sn	Au-Ca-Sr	Au-Ca-Ta
Au-Ca-Tb	Au-Ca-Ti	Au-Ca-V	Au-Ca-W	Au-Ca-Y	Au-Ca-Yb	Au-Ca-Zn	Au-Ca-Zr

Au-Ce-Co	Au-Ce-Cr	Au-Ce-Cu	Au-Ce-Dy	Au-Ce-Er	Au-Ce-Fe	Au-Ce-Ga	Au-Ce-Gd
Au-Ce-Ge	Au-Ce-Hf	Au-Ce-La	Au-Ce-Mg	Au-Ce-Mn	Au-Ce-Mo	Au-Ce-Nb	Au-Ce-Nd
Au-Ce-Ni	Au-Ce-P	Au-Ce-Pb	Au-Ce-Pd	Au-Ce-Pr	Au-Ce-Pt	Au-Ce-Ru	Au-Ce-Sb
Au-Ce-Sc	Au-Ce-Si	Au-Ce-Sm	Au-Ce-Sn	Au-Ce-Sr	Au-Ce-Ta	Au-Ce-Tb	Au-Ce-Ti
Au-Ce-V	Au-Ce-W	Au-Ce-Y	Au-Ce-Yb	Au-Ce-Zn	Au-Ce-Zr	Au-Co-Cr	Au-Co-Cu
Au-Co-Dy	Au-Co-Er	Au-Co-Fe	Au-Co-Ga	Au-Co-Gd	Au-Co-Ge	Au-Co-Hf	Au-Co-La
Au-Co-Mg	Au-Co-Mn	Au-Co-Mo	Au-Co-Nb	Au-Co-Nd	Au-Co-Ni	Au-Co-P	Au-Co-Pb
Au-Co-Pd	Au-Co-Pr	Au-Co-Pt	Au-Co-Ru	Au-Co-Sb	Au-Co-Sc	Au-Co-Si	Au-Co-Sm
Au-Co-Sn	Au-Co-Sr	Au-Co-Ta	Au-Co-Tb	Au-Co-Ti	Au-Co-V	Au-Co-W	Au-Co-Y
Au-Co-Yb	Au-Co-Zn	Au-Co-Zr	Au-Cr-Cu	Au-Cr-Dy	Au-Cr-Er	Au-Cr-Fe	Au-Cr-Ga
Au-Cr-Gd	Au-Cr-Ge	Au-Cr-Hf	Au-Cr-La	Au-Cr-Mg	Au-Cr-Mn	Au-Cr-Mo	Au-Cr-Nb
Au-Cr-Nd	Au-Cr-Ni	Au-Cr-P	Au-Cr-Pb	Au-Cr-Pd	Au-Cr-Pr	Au-Cr-Pt	Au-Cr-Ru
Au-Cr-Sb	Au-Cr-Sc	Au-Cr-Si	Au-Cr-Sm	Au-Cr-Sn	Au-Cr-Sr	Au-Cr-Ta	Au-Cr-Tb
Au-Cr-Ti	Au-Cr-V	Au-Cr-W	Au-Cr-Y	Au-Cr-Yb	Au-Cr-Zn	Au-Cr-Zr	Au-Cu-Dy
Au-Cu-Ga	Au-Cu-Hf	Au-Cu-Mn	Au-Cu-Mo	Au-Cu-Nb	Au-Cu-Nd	Au-Cu-Ni	Au-Cu-P
Au-Cu-Pt	Au-Cu-Ru	Au-Cu-Sb	Au-Cu-Sc	Au-Cu-Sm	Au-Cu-Sn	Au-Cu-Sr	Au-Cu-Ta
Au-Cu-Ti	Au-Cu-V	Au-Cu-W	Au-Cu-Y	Au-Cu-Yb	Au-Cu-Zr	Au-Dy-Er	Au-Dy-Fe
Au-Dy-Ga	Au-Dy-Gd	Au-Dy-Ge	Au-Dy-Hf	Au-Dy-La	Au-Dy-Mg	Au-Dy-Mn	Au-Dy-Mo
Au-Dy-Nb	Au-Dy-Nd	Au-Dy-Ni	Au-Dy-P	Au-Dy-Pb	Au-Dy-Pd	Au-Dy-Pr	Au-Dy-Pt
Au-Dy-Ru	Au-Dy-Sb	Au-Dy-Sc	Au-Dy-Si	Au-Dy-Sm	Au-Dy-Sn	Au-Dy-Sr	Au-Dy-Ta
Au-Dy-Tb	Au-Dy-Ti	Au-Dy-V	Au-Dy-W	Au-Dy-Y	Au-Dy-Yb	Au-Dy-Zn	Au-Dy-Zr
Au-Er-Fe	Au-Er-Ga	Au-Er-Gd	Au-Er-Ge	Au-Er-Hf	Au-Er-La	Au-Er-Mn	Au-Er-Mo
Au-Er-Nb	Au-Er-Nd	Au-Er-Ni	Au-Er-P	Au-Er-Pb	Au-Er-Pd	Au-Er-Pr	Au-Er-Pt
Au-Er-Ru	Au-Er-Sb	Au-Er-Sc	Au-Er-Si	Au-Er-Sm	Au-Er-Sn	Au-Er-Sr	Au-Er-Ta
Au-Er-Tb	Au-Er-Ti	Au-Er-V	Au-Er-W	Au-Er-Y	Au-Er-Yb	Au-Er-Zn	Au-Er-Zr

Au-Fe-Ga	Au-Fe-Hf	Au-Fe-Mg	Au-Fe-Mn	Au-Fe-Mo	Au-Fe-Nb	Au-Fe-Nd	Au-Fe-Ni
Au-Fe-P	Au-Fe-Pb	Au-Fe-Pt	Au-Fe-Ru	Au-Fe-Sb	Au-Fe-Sc	Au-Fe-Sm	Au-Fe-Sn
Au-Fe-Sr	Au-Fe-Ta	Au-Fe-Ti	Au-Fe-V	Au-Fe-W	Au-Fe-Y	Au-Fe-Yb	Au-Fe-Zn
Au-Fe-Zr	Au-Ga-Gd	Au-Ga-Ge	Au-Ga-Hf	Au-Ga-La	Au-Ga-Mg	Au-Ga-Mn	Au-Ga-Mo
Au-Ga-Nb	Au-Ga-Nd	Au-Ga-Ni	Au-Ga-P	Au-Ga-Pb	Au-Ga-Pd	Au-Ga-Pr	Au-Ga-Pt
Au-Ga-Ru	Au-Ga-Sb	Au-Ga-Sc	Au-Ga-Si	Au-Ga-Sm	Au-Ga-Sn	Au-Ga-Sr	Au-Ga-Ta
Au-Ga-Tb	Au-Ga-Ti	Au-Ga-V	Au-Ga-W	Au-Ga-Y	Au-Ga-Yb	Au-Ga-Zn	Au-Ga-Zr
Au-Gd-Ge	Au-Gd-Hf	Au-Gd-La	Au-Gd-Mn	Au-Gd-Mo	Au-Gd-Nb	Au-Gd-Nd	Au-Gd-Ni
Au-Gd-P	Au-Gd-Pb	Au-Gd-Pd	Au-Gd-Pr	Au-Gd-Pt	Au-Gd-Ru	Au-Gd-Sb	Au-Gd-Sc
Au-Gd-Si	Au-Gd-Sm	Au-Gd-Sn	Au-Gd-Sr	Au-Gd-Ta	Au-Gd-Tb	Au-Gd-Ti	Au-Gd-V
Au-Gd-W	Au-Gd-Y	Au-Gd-Yb	Au-Gd-Zn	Au-Gd-Zr	Au-Ge-Hf	Au-Ge-La	Au-Ge-Mg
Au-Ge-Mn	Au-Ge-Mo	Au-Ge-Nb	Au-Ge-Nd	Au-Ge-Ni	Au-Ge-P	Au-Ge-Pb	Au-Ge-Pr
Au-Ge-Pt	Au-Ge-Ru	Au-Ge-Sb	Au-Ge-Sc	Au-Ge-Sm	Au-Ge-Sn	Au-Ge-Sr	Au-Ge-Ta
Au-Ge-Tb	Au-Ge-Ti	Au-Ge-V	Au-Ge-W	Au-Ge-Y	Au-Ge-Yb	Au-Ge-Zn	Au-Ge-Zr
Au-Hf-La	Au-Hf-Mg	Au-Hf-Mn	Au-Hf-Mo	Au-Hf-Nb	Au-Hf-Nd	Au-Hf-Ni	Au-Hf-P
Au-Hf-Pb	Au-Hf-Pd	Au-Hf-Pr	Au-Hf-Pt	Au-Hf-Ru	Au-Hf-Sb	Au-Hf-Sc	Au-Hf-Si
Au-Hf-Sm	Au-Hf-Sn	Au-Hf-Sr	Au-Hf-Ta	Au-Hf-Tb	Au-Hf-Ti	Au-Hf-V	Au-Hf-W
Au-Hf-Y	Au-Hf-Yb	Au-Hf-Zn	Au-Hf-Zr	Au-La-Mn	Au-La-Mo	Au-La-Nb	Au-La-Nd
Au-La-Ni	Au-La-P	Au-La-Pb	Au-La-Pd	Au-La-Pr	Au-La-Pt	Au-La-Ru	Au-La-Sb
Au-La-Sc	Au-La-Si	Au-La-Sm	Au-La-Sn	Au-La-Sr	Au-La-Ta	Au-La-Tb	Au-La-Ti
Au-La-V	Au-La-W	Au-La-Y	Au-La-Yb	Au-La-Zr	Au-Mg-Mn	Au-Mg-Mo	Au-Mg-Nb
Au-Mg-Nd	Au-Mg-Ni	Au-Mg-P	Au-Mg-Pb	Au-Mg-Pt	Au-Mg-Ru	Au-Mg-Sb	Au-Mg-Sc
Au-Mg-Si	Au-Mg-Sm	Au-Mg-Sn	Au-Mg-Sr	Au-Mg-Ta	Au-Mg-Ti	Au-Mg-V	Au-Mg-W
Au-Mg-Y	Au-Mg-Yb	Au-Mg-Zr	Au-Mn-Mo	Au-Mn-Nb	Au-Mn-Nd	Au-Mn-Ni	Au-Mn-P
Au-Mn-Pb	Au-Mn-Pd	Au-Mn-Pr	Au-Mn-Pt	Au-Mn-Ru	Au-Mn-Sb	Au-Mn-Sc	Au-Mn-Si

Au-Mn-Sm	Au-Mn-Sn	Au-Mn-Sr	Au-Mn-Ta	Au-Mn-Tb	Au-Mn-Ti	Au-Mn-V	Au-Mn-W
Au-Mn-Y	Au-Mn-Yb	Au-Mn-Zn	Au-Mn-Zr	Au-Mo-Nb	Au-Mo-Nd	Au-Mo-Ni	Au-Mo-P
Au-Mo-Pb	Au-Mo-Pd	Au-Mo-Pr	Au-Mo-Pt	Au-Mo-Ru	Au-Mo-Sb	Au-Mo-Sc	Au-Mo-Si
Au-Mo-Sm	Au-Mo-Sn	Au-Mo-Sr	Au-Mo-Ta	Au-Mo-Tb	Au-Mo-Ti	Au-Mo-V	Au-Mo-W
Au-Mo-Y	Au-Mo-Yb	Au-Mo-Zn	Au-Mo-Zr	Au-Nb-Nd	Au-Nb-Ni	Au-Nb-P	Au-Nb-Pb
Au-Nb-Pd	Au-Nb-Pr	Au-Nb-Pt	Au-Nb-Ru	Au-Nb-Sb	Au-Nb-Sc	Au-Nb-Si	Au-Nb-Sm
Au-Nb-Sn	Au-Nb-Sr	Au-Nb-Ta	Au-Nb-Tb	Au-Nb-Ti	Au-Nb-V	Au-Nb-W	Au-Nb-Y
Au-Nb-Yb	Au-Nb-Zn	Au-Nb-Zr	Au-Nd-Ni	Au-Nd-P	Au-Nd-Pb	Au-Nd-Pd	Au-Nd-Pr
Au-Nd-Pt	Au-Nd-Ru	Au-Nd-Sb	Au-Nd-Sc	Au-Nd-Si	Au-Nd-Sm	Au-Nd-Sn	Au-Nd-Sr
Au-Nd-Ta	Au-Nd-Tb	Au-Nd-Ti	Au-Nd-V	Au-Nd-W	Au-Nd-Y	Au-Nd-Yb	Au-Nd-Zn
Au-Nd-Zr	Au-Ni-P	Au-Ni-Pb	Au-Ni-Pd	Au-Ni-Pr	Au-Ni-Pt	Au-Ni-Ru	Au-Ni-Sb
Au-Ni-Sc	Au-Ni-Si	Au-Ni-Sm	Au-Ni-Sn	Au-Ni-Sr	Au-Ni-Ta	Au-Ni-Tb	Au-Ni-Ti
Au-Ni-V	Au-Ni-W	Au-Ni-Y	Au-Ni-Yb	Au-Ni-Zn	Au-Ni-Zr	Au-P-Pb	Au-P-Pd
Au-P-Pr	Au-P-Pt	Au-P-Ru	Au-P-Sb	Au-P-Sc	Au-P-Si	Au-P-Sm	Au-P-Sn
Au-P-Sr	Au-P-Ta	Au-P-Tb	Au-P-Ti	Au-P-V	Au-P-W	Au-P-Y	Au-P-Yb
Au-P-Zn	Au-P-Zr	Au-Pb-Pd	Au-Pb-Pr	Au-Pb-Pt	Au-Pb-Ru	Au-Pb-Sc	Au-Pb-Si
Au-Pb-Sm	Au-Pb-Sn	Au-Pb-Sr	Au-Pb-Ta	Au-Pb-Tb	Au-Pb-Ti	Au-Pb-V	Au-Pb-W
Au-Pb-Y	Au-Pb-Yb	Au-Pb-Zr	Au-Pd-Pr	Au-Pd-Pt	Au-Pd-Ru	Au-Pd-Sc	Au-Pd-Sm
Au-Pd-Sn	Au-Pd-Sr	Au-Pd-Ta	Au-Pd-Tb	Au-Pd-Ti	Au-Pd-V	Au-Pd-W	Au-Pd-Y
Au-Pd-Yb	Au-Pd-Zn	Au-Pd-Zr	Au-Pr-Pt	Au-Pr-Ru	Au-Pr-Sb	Au-Pr-Sc	Au-Pr-Si
Au-Pr-Sm	Au-Pr-Sn	Au-Pr-Sr	Au-Pr-Ta	Au-Pr-Tb	Au-Pr-Ti	Au-Pr-V	Au-Pr-W
Au-Pr-Y	Au-Pr-Yb	Au-Pr-Zn	Au-Pr-Zr	Au-Pt-Ru	Au-Pt-Sb	Au-Pt-Sc	Au-Pt-Si
Au-Pt-Sm	Au-Pt-Sn	Au-Pt-Sr	Au-Pt-Ta	Au-Pt-Tb	Au-Pt-Ti	Au-Pt-V	Au-Pt-W
Au-Pt-Y	Au-Pt-Yb	Au-Pt-Zn	Au-Pt-Zr	Au-Ru-Sb	Au-Ru-Sc	Au-Ru-Si	Au-Ru-Sm
Au-Ru-Sn	Au-Ru-Sr	Au-Ru-Ta	Au-Ru-Tb	Au-Ru-Ti	Au-Ru-V	Au-Ru-W	Au-Ru-Y

Au-Ru-Yb	Au-Ru-Zn	Au-Ru-Zr	Au-Sb-Sc	Au-Sb-Sm	Au-Sb-Sn	Au-Sb-Sr	Au-Sb-Ta
Au-Sb-Tb	Au-Sb-Ti	Au-Sb-V	Au-Sb-W	Au-Sb-Y	Au-Sb-Yb	Au-Sb-Zn	Au-Sb-Zr
Au-Sc-Si	Au-Sc-Sm	Au-Sc-Sn	Au-Sc-Sr	Au-Sc-Ta	Au-Sc-Tb	Au-Sc-Ti	Au-Sc-V
Au-Sc-W	Au-Sc-Y	Au-Sc-Yb	Au-Sc-Zn	Au-Sc-Zr	Au-Si-Sm	Au-Si-Sn	Au-Si-Sr
Au-Si-Ta	Au-Si-Ti	Au-Si-V	Au-Si-W	Au-Si-Y	Au-Si-Yb	Au-Si-Zn	Au-Si-Zr
Au-Sm-Sn	Au-Sm-Sr	Au-Sm-Ta	Au-Sm-Tb	Au-Sm-Ti	Au-Sm-V	Au-Sm-W	Au-Sm-Y
Au-Sm-Yb	Au-Sm-Zn	Au-Sm-Zr	Au-Sn-Sr	Au-Sn-Ta	Au-Sn-Tb	Au-Sn-Ti	Au-Sn-V
Au-Sn-W	Au-Sn-Y	Au-Sn-Yb	Au-Sn-Zn	Au-Sn-Zr	Au-Sr-Ta	Au-Sr-Tb	Au-Sr-Ti
Au-Sr-V	Au-Sr-W	Au-Sr-Y	Au-Sr-Yb	Au-Sr-Zn	Au-Sr-Zr	Au-Ta-Tb	Au-Ta-Ti
Au-Ta-V	Au-Ta-W	Au-Ta-Y	Au-Ta-Yb	Au-Ta-Zn	Au-Ta-Zr	Au-Tb-Ti	Au-Tb-V
Au-Tb-W	Au-Tb-Y	Au-Tb-Yb	Au-Tb-Zn	Au-Tb-Zr	Au-Ti-V	Au-Ti-W	Au-Ti-Y
Au-Ti-Yb	Au-Ti-Zn	Au-Ti-Zr	Au-V-W	Au-V-Y	Au-V-Yb	Au-V-Zn	Au-V-Zr
Au-W-Y	Au-W-Yb	Au-W-Zn	Au-W-Zr	Au-Y-Yb	Au-Y-Zn	Au-Y-Zr	Au-Yb-Zn
Au-Yb-Zr	Au-Zn-Zr	B-Ba-Be	B-Ba-C	B-Ba-Ca	B-Ba-Ce	B-Ba-Co	B-Ba-Cr
B-Ba-Cu	B-Ba-Dy	B-Ba-Er	B-Ba-Fe	B-Ba-Ga	B-Ba-Gd	B-Ba-Ge	B-Ba-Hf
B-Ba-La	B-Ba-Mg	B-Ba-Mn	B-Ba-Mo	B-Ba-Nb	B-Ba-Nd	B-Ba-Ni	B-Ba-P
B-Ba-Pb	B-Ba-Pd	B-Ba-Pr	B-Ba-Pt	B-Ba-Ru	B-Ba-Sb	B-Ba-Sc	B-Ba-Si
B-Ba-Sm	B-Ba-Sn	B-Ba-Sr	B-Ba-Ta	B-Ba-Tb	B-Ba-Ti	B-Ba-V	B-Ba-W
B-Ba-Y	B-Ba-Yb	B-Ba-Zn	B-Ba-Zr	B-Be-C	B-Be-Ca	B-Be-Ce	B-Be-Co
B-Be-Cr	B-Be-Dy	B-Be-Er	B-Be-Ga	B-Be-Gd	B-Be-Ge	B-Be-La	B-Be-Mg
B-Be-Mn	B-Be-Mo	B-Be-Nd	B-Be-Ni	B-Be-P	B-Be-Pb	B-Be-Pd	B-Be-Pr
B-Be-Pt	B-Be-Ru	B-Be-Sb	B-Be-Sc	B-Be-Sm	B-Be-Sn	B-Be-Sr	B-Be-Ta
B-Be-Tb	B-Be-V	B-Be-W	B-Be-Y	B-Be-Yb	B-Be-Zn	B-C-Ca	B-C-Ce
B-C-Cu	B-C-Dy	B-C-Er	B-C-Ga	B-C-Gd	B-C-Ge	B-C-Hf	B-C-La
B-C-Mg	B-C-Mn	B-C-Nd	B-C-Pb	B-C-Pd	B-C-Pr	B-C-Pt	B-C-Ru

B-C-Sb	B-C-Sc	B-C-Sm	B-C-Sn	B-C-Sr	B-C-Ta	B-C-Tb	B-C-Ti
B-C-V	B-C-Y	B-C-Yb	B-C-Zn	B-C-Zr	B-Ca-Ce	B-Ca-Co	B-Ca-Cr
B-Ca-Cu	B-Ca-Dy	B-Ca-Er	B-Ca-Fe	B-Ca-Ga	B-Ca-Gd	B-Ca-Ge	B-Ca-Hf
B-Ca-La	B-Ca-Mg	B-Ca-Mn	B-Ca-Mo	B-Ca-Nb	B-Ca-Nd	B-Ca-Ni	B-Ca-P
B-Ca-Pb	B-Ca-Pd	B-Ca-Pr	B-Ca-Pt	B-Ca-Ru	B-Ca-Sb	B-Ca-Sc	B-Ca-Si
B-Ca-Sm	B-Ca-Sn	B-Ca-Sr	B-Ca-Ta	B-Ca-Tb	B-Ca-Ti	B-Ca-V	B-Ca-W
B-Ca-Y	B-Ca-Yb	B-Ca-Zn	B-Ca-Zr	B-Ce-Co	B-Ce-Cr	B-Ce-Cu	B-Ce-Dy
B-Ce-Er	B-Ce-Fe	B-Ce-Ga	B-Ce-Gd	B-Ce-Ge	B-Ce-Hf	B-Ce-La	B-Ce-Mg
B-Ce-Mn	B-Ce-Mo	B-Ce-Nb	B-Ce-Nd	B-Ce-Ni	B-Ce-P	B-Ce-Pb	B-Ce-Pd
B-Ce-Pr	B-Ce-Pt	B-Ce-Ru	B-Ce-Sb	B-Ce-Sc	B-Ce-Si	B-Ce-Sm	B-Ce-Sn
B-Ce-Sr	B-Ce-Ta	B-Ce-Tb	B-Ce-Ti	B-Ce-V	B-Ce-W	B-Ce-Y	B-Ce-Yb
B-Ce-Zn	B-Ce-Zr	B-Co-Cu	B-Co-Dy	B-Co-Ga	B-Co-Mg	B-Co-Pb	B-Co-Pt
B-Co-Ru	B-Co-Sb	B-Co-Sc	B-Co-Sn	B-Co-Sr	B-Co-Yb	B-Co-Zn	B-Cr-Cu
B-Cr-Dy	B-Cr-Er	B-Cr-Ga	B-Cr-Gd	B-Cr-Hf	B-Cr-Mg	B-Cr-Mn	B-Cr-Nb
B-Cr-Nd	B-Cr-Pb	B-Cr-Pr	B-Cr-Pt	B-Cr-Ru	B-Cr-Sb	B-Cr-Sc	B-Cr-Sm
B-Cr-Sn	B-Cr-Sr	B-Cr-Ta	B-Cr-Tb	B-Cr-Ti	B-Cr-V	B-Cr-W	B-Cr-Y
B-Cr-Yb	B-Cr-Zn	B-Cu-Dy	B-Cu-Mg	B-Cu-Mn	B-Cu-Mo	B-Cu-Pb	B-Cu-Pt
B-Cu-Ru	B-Cu-Sb	B-Cu-Sc	B-Cu-Sm	B-Cu-Sr	B-Cu-Ta	B-Cu-W	B-Cu-Yb
B-Cu-Zn	B-Dy-Er	B-Dy-Fe	B-Dy-Ga	B-Dy-Gd	B-Dy-Ge	B-Dy-Hf	B-Dy-La
B-Dy-Mg	B-Dy-Mn	B-Dy-Mo	B-Dy-Nb	B-Dy-Nd	B-Dy-Ni	B-Dy-P	B-Dy-Pb
B-Dy-Pd	B-Dy-Pr	B-Dy-Pt	B-Dy-Ru	B-Dy-Sb	B-Dy-Sc	B-Dy-Si	B-Dy-Sm
B-Dy-Sn	B-Dy-Sr	B-Dy-Ta	B-Dy-Tb	B-Dy-Ti	B-Dy-V	B-Dy-W	B-Dy-Y
B-Dy-Yb	B-Dy-Zn	B-Dy-Zr	B-Er-Fe	B-Er-Ga	B-Er-Gd	B-Er-Ge	B-Er-Hf
B-Er-La	B-Er-Mg	B-Er-Mn	B-Er-Mo	B-Er-Nb	B-Er-Nd	B-Er-P	B-Er-Pb
B-Er-Pd	B-Er-Pr	B-Er-Pt	B-Er-Ru	B-Er-Sb	B-Er-Sc	B-Er-Si	B-Er-Sm

B-Er-Sn	B-Er-Sr	B-Er-Ta	B-Er-Tb	B-Er-Ti	B-Er-V	B-Er-W	B-Er-Y
B-Er-Yb	B-Er-Zn	B-Er-Zr	B-Fe-Mg	B-Fe-Pb	B-Fe-Pt	B-Fe-Sb	B-Fe-Sc
B-Fe-Sr	B-Fe-Yb	B-Fe-Zn	B-Ga-Ge	B-Ga-Hf	B-Ga-La	B-Ga-Mg	B-Ga-Mn
B-Ga-Mo	B-Ga-Nb	B-Ga-Nd	B-Ga-Ni	B-Ga-P	B-Ga-Pb	B-Ga-Pd	B-Ga-Pr
B-Ga-Pt	B-Ga-Ru	B-Ga-Sb	B-Ga-Sc	B-Ga-Si	B-Ga-Sm	B-Ga-Sn	B-Ga-Sr
B-Ga-Ta	B-Ga-Tb	B-Ga-Ti	B-Ga-V	B-Ga-W	B-Ga-Y	B-Ga-Yb	B-Ga-Zn
B-Gd-Ge	B-Gd-Hf	B-Gd-La	B-Gd-Mg	B-Gd-Mn	B-Gd-Nb	B-Gd-Nd	B-Gd-P
B-Gd-Pb	B-Gd-Pd	B-Gd-Pr	B-Gd-Pt	B-Gd-Ru	B-Gd-Sb	B-Gd-Sc	B-Gd-Si
B-Gd-Sm	B-Gd-Sn	B-Gd-Sr	B-Gd-Ta	B-Gd-Tb	B-Gd-Ti	B-Gd-V	B-Gd-W
B-Gd-Y	B-Gd-Yb	B-Gd-Zn	B-Ge-Hf	B-Ge-La	B-Ge-Mg	B-Ge-Nd	B-Ge-P
B-Ge-Pb	B-Ge-Pr	B-Ge-Pt	B-Ge-Ru	B-Ge-Sb	B-Ge-Sc	B-Ge-Sm	B-Ge-Sn
B-Ge-Sr	B-Ge-Tb	B-Ge-W	B-Ge-Y	B-Ge-Yb	B-Ge-Zn	B-Hf-La	B-Hf-Mg
B-Hf-Mn	B-Hf-Mo	B-Hf-Nd	B-Hf-Pb	B-Hf-Pd	B-Hf-Pr	B-Hf-Pt	B-Hf-Ru
B-Hf-Sb	B-Hf-Sc	B-Hf-Sm	B-Hf-Sn	B-Hf-Sr	B-Hf-Tb	B-Hf-W	B-Hf-Y
B-Hf-Yb	B-Hf-Zn	B-La-Mg	B-La-Nd	B-La-P	B-La-Pb	B-La-Pd	B-La-Pr
B-La-Pt	B-La-Ru	B-La-Sb	B-La-Sc	B-La-Si	B-La-Sm	B-La-Sn	B-La-Sr
B-La-Ta	B-La-Tb	B-La-Ti	B-La-V	B-La-W	B-La-Y	B-La-Yb	B-La-Zn
B-Mg-Mn	B-Mg-Mo	B-Mg-Nb	B-Mg-Nd	B-Mg-Ni	B-Mg-P	B-Mg-Pb	B-Mg-Pd
B-Mg-Pr	B-Mg-Pt	B-Mg-Ru	B-Mg-Sb	B-Mg-Sc	B-Mg-Si	B-Mg-Sm	B-Mg-Sn
B-Mg-Sr	B-Mg-Ta	B-Mg-Tb	B-Mg-Ti	B-Mg-V	B-Mg-W	B-Mg-Y	B-Mg-Yb
B-Mg-Zn	B-Mg-Zr	B-Mn-Mo	B-Mn-Nd	B-Mn-Pb	B-Mn-Pr	B-Mn-Pt	B-Mn-Ru
B-Mn-Sb	B-Mn-Sc	B-Mn-Sm	B-Mn-Sn	B-Mn-Sr	B-Mn-Ta	B-Mn-Tb	B-Mn-Ti
B-Mn-V	B-Mn-W	B-Mn-Yb	B-Mn-Zn	B-Mo-Nd	B-Mo-Pb	B-Mo-Pd	B-Mo-Pr
B-Mo-Pt	B-Mo-Sb	B-Mo-Sc	B-Mo-Sm	B-Mo-Sn	B-Mo-Sr	B-Mo-Ta	B-Mo-Tb
B-Mo-Ti	B-Mo-V	B-Mo-W	B-Mo-Y	B-Mo-Yb	B-Mo-Zn	B-Nb-Nd	B-Nb-P

B-Nb-Pb	B-Nb-Pr	B-Nb-Pt	B-Nb-Ru	B-Nb-Sb	B-Nb-Sc	B-Nb-Sm	B-Nb-Sr
B-Nb-Tb	B-Nb-Y	B-Nb-Yb	B-Nb-Zn	B-Nd-P	B-Nd-Pb	B-Nd-Pd	B-Nd-Pr
B-Nd-Pt	B-Nd-Ru	B-Nd-Sb	B-Nd-Sc	B-Nd-Si	B-Nd-Sm	B-Nd-Sn	B-Nd-Sr
B-Nd-Ta	B-Nd-Tb	B-Nd-Ti	B-Nd-V	B-Nd-W	B-Nd-Y	B-Nd-Yb	B-Nd-Zn
B-Nd-Zr	B-Ni-Pb	B-Ni-Pt	B-Ni-Sb	B-Ni-Sc	B-Ni-Sr	B-Ni-Tb	B-Ni-Yb
B-Ni-Zn	B-P-Pb	B-P-Pr	B-P-Pt	B-P-Sb	B-P-Sc	B-P-Sm	B-P-Sn
B-P-Sr	B-P-Tb	B-P-Y	B-P-Yb	B-P-Zn	B-Pb-Pd	B-Pb-Pr	B-Pb-Pt
B-Pb-Ru	B-Pb-Sb	B-Pb-Sc	B-Pb-Si	B-Pb-Sm	B-Pb-Sn	B-Pb-Sr	B-Pb-Ta
B-Pb-Tb	B-Pb-Ti	B-Pb-V	B-Pb-W	B-Pb-Y	B-Pb-Yb	B-Pb-Zn	B-Pb-Zr
B-Pd-Pr	B-Pd-Pt	B-Pd-Ru	B-Pd-Sb	B-Pd-Sc	B-Pd-Sm	B-Pd-Sn	B-Pd-Sr
B-Pd-Tb	B-Pd-V	B-Pd-W	B-Pd-Y	B-Pd-Yb	B-Pd-Zn	B-Pr-Pt	B-Pr-Ru
B-Pr-Sb	B-Pr-Sc	B-Pr-Si	B-Pr-Sm	B-Pr-Sn	B-Pr-Sr	B-Pr-Ta	B-Pr-Tb
B-Pr-Ti	B-Pr-V	B-Pr-W	B-Pr-Y	B-Pr-Yb	B-Pr-Zn	B-Pt-Ru	B-Pt-Sb
B-Pt-Sc	B-Pt-Si	B-Pt-Sm	B-Pt-Sn	B-Pt-Sr	B-Pt-Ta	B-Pt-Tb	B-Pt-Ti
B-Pt-V	B-Pt-W	B-Pt-Y	B-Pt-Yb	B-Pt-Zn	B-Pt-Zr	B-Ru-Sb	B-Ru-Sc
B-Ru-Sm	B-Ru-Sn	B-Ru-Sr	B-Ru-Tb	B-Ru-Ti	B-Ru-V	B-Ru-Y	B-Ru-Yb
B-Ru-Zn	B-Sb-Sc	B-Sb-Si	B-Sb-Sm	B-Sb-Sn	B-Sb-Sr	B-Sb-Ta	B-Sb-Tb
B-Sb-Ti	B-Sb-V	B-Sb-W	B-Sb-Y	B-Sb-Yb	B-Sb-Zn	B-Sb-Zr	B-Sc-Si
B-Sc-Sm	B-Sc-Sn	B-Sc-Sr	B-Sc-Ta	B-Sc-Tb	B-Sc-Ti	B-Sc-V	B-Sc-W
B-Sc-Y	B-Sc-Yb	B-Sc-Zn	B-Sc-Zr	B-Si-Sm	B-Si-Sr	B-Si-Y	B-Si-Yb
B-Si-Zn	B-Sm-Sn	B-Sm-Sr	B-Sm-Ta	B-Sm-Tb	B-Sm-Ti	B-Sm-V	B-Sm-W
B-Sm-Y	B-Sm-Yb	B-Sm-Zn	B-Sm-Zr	B-Sn-Sr	B-Sn-Ta	B-Sn-Tb	B-Sn-Ti
B-Sn-V	B-Sn-W	B-Sn-Y	B-Sn-Yb	B-Sn-Zn	B-Sr-Ta	B-Sr-Tb	B-Sr-Ti
B-Sr-V	B-Sr-W	B-Sr-Y	B-Sr-Yb	B-Sr-Zn	B-Sr-Zr	B-Ta-Tb	B-Ta-V
B-Ta-W	B-Ta-Y	B-Ta-Yb	B-Ta-Zn	B-Tb-Ti	B-Tb-V	B-Tb-W	B-Tb-Y

B-Tb-Yb	B-Tb-Zn	B-Tb-Zr	B-Ti-W	B-Ti-Y	B-Ti-Yb	B-Ti-Zn	B-V-W
B-V-Y	B-V-Yb	B-V-Zn	B-W-Y	B-W-Yb	B-W-Zn	B-Y-Yb	B-Y-Zn
B-Y-Zr	B-Yb-Zn	B-Yb-Zr	B-Zn-Zr	Ba-Be-C	Ba-Be-Ca	Ba-Be-Ce	Ba-Be-Co
Ba-Be-Cr	Ba-Be-Cu	Ba-Be-Dy	Ba-Be-Er	Ba-Be-Fe	Ba-Be-Ga	Ba-Be-Gd	Ba-Be-Ge
Ba-Be-Hf	Ba-Be-La	Ba-Be-Mg	Ba-Be-Mn	Ba-Be-Mo	Ba-Be-Nb	Ba-Be-Nd	Ba-Be-Ni
Ba-Be-P	Ba-Be-Pb	Ba-Be-Pd	Ba-Be-Pr	Ba-Be-Pt	Ba-Be-Ru	Ba-Be-Sb	Ba-Be-Sc
Ba-Be-Si	Ba-Be-Sm	Ba-Be-Sn	Ba-Be-Sr	Ba-Be-Ta	Ba-Be-Tb	Ba-Be-Ti	Ba-Be-V
Ba-Be-W	Ba-Be-Y	Ba-Be-Yb	Ba-Be-Zn	Ba-Be-Zr	Ba-C-Ca	Ba-C-Ce	Ba-C-Co
Ba-C-Cr	Ba-C-Cu	Ba-C-Dy	Ba-C-Er	Ba-C-Fe	Ba-C-Ga	Ba-C-Gd	Ba-C-Ge
Ba-C-Hf	Ba-C-La	Ba-C-Mg	Ba-C-Mn	Ba-C-Mo	Ba-C-Nb	Ba-C-Nd	Ba-C-Ni
Ba-C-P	Ba-C-Pb	Ba-C-Pd	Ba-C-Pr	Ba-C-Pt	Ba-C-Ru	Ba-C-Sb	Ba-C-Sc
Ba-C-Si	Ba-C-Sm	Ba-C-Sn	Ba-C-Sr	Ba-C-Ta	Ba-C-Tb	Ba-C-Ti	Ba-C-V
Ba-C-W	Ba-C-Y	Ba-C-Yb	Ba-C-Zn	Ba-C-Zr	Ba-Ca-Ce	Ba-Ca-Co	Ba-Ca-Cr
Ba-Ca-Cu	Ba-Ca-Dy	Ba-Ca-Er	Ba-Ca-Fe	Ba-Ca-Ga	Ba-Ca-Gd	Ba-Ca-Ge	Ba-Ca-Hf
Ba-Ca-La	Ba-Ca-Mg	Ba-Ca-Mn	Ba-Ca-Mo	Ba-Ca-Nb	Ba-Ca-Nd	Ba-Ca-Ni	Ba-Ca-P
Ba-Ca-Pb	Ba-Ca-Pd	Ba-Ca-Pr	Ba-Ca-Pt	Ba-Ca-Ru	Ba-Ca-Sb	Ba-Ca-Sc	Ba-Ca-Si
Ba-Ca-Sm	Ba-Ca-Sn	Ba-Ca-Sr	Ba-Ca-Ta	Ba-Ca-Tb	Ba-Ca-Ti	Ba-Ca-V	Ba-Ca-W
Ba-Ca-Y	Ba-Ca-Yb	Ba-Ca-Zn	Ba-Ca-Zr	Ba-Ce-Co	Ba-Ce-Cr	Ba-Ce-Cu	Ba-Ce-Dy
Ba-Ce-Er	Ba-Ce-Fe	Ba-Ce-Ga	Ba-Ce-Gd	Ba-Ce-Ge	Ba-Ce-Hf	Ba-Ce-La	Ba-Ce-Mg
Ba-Ce-Mn	Ba-Ce-Mo	Ba-Ce-Nb	Ba-Ce-Nd	Ba-Ce-Ni	Ba-Ce-P	Ba-Ce-Pb	Ba-Ce-Pd
Ba-Ce-Pr	Ba-Ce-Pt	Ba-Ce-Ru	Ba-Ce-Sb	Ba-Ce-Sc	Ba-Ce-Si	Ba-Ce-Sm	Ba-Ce-Sn
Ba-Ce-Sr	Ba-Ce-Ta	Ba-Ce-Tb	Ba-Ce-Ti	Ba-Ce-V	Ba-Ce-W	Ba-Ce-Y	Ba-Ce-Yb
Ba-Ce-Zn	Ba-Ce-Zr	Ba-Co-Cr	Ba-Co-Cu	Ba-Co-Dy	Ba-Co-Er	Ba-Co-Fe	Ba-Co-Ga
Ba-Co-Gd	Ba-Co-Ge	Ba-Co-Hf	Ba-Co-La	Ba-Co-Mg	Ba-Co-Mn	Ba-Co-Mo	Ba-Co-Nb
Ba-Co-Nd	Ba-Co-Ni	Ba-Co-P	Ba-Co-Pb	Ba-Co-Pd	Ba-Co-Pr	Ba-Co-Pt	Ba-Co-Ru

Ba-Co-Sb	Ba-Co-Sc	Ba-Co-Si	Ba-Co-Sm	Ba-Co-Sn	Ba-Co-Sr	Ba-Co-Ta	Ba-Co-Tb
Ba-Co-Ti	Ba-Co-V	Ba-Co-W	Ba-Co-Y	Ba-Co-Yb	Ba-Co-Zn	Ba-Co-Zr	Ba-Cr-Cu
Ba-Cr-Dy	Ba-Cr-Er	Ba-Cr-Fe	Ba-Cr-Ga	Ba-Cr-Gd	Ba-Cr-Ge	Ba-Cr-Hf	Ba-Cr-La
Ba-Cr-Mg	Ba-Cr-Mn	Ba-Cr-Mo	Ba-Cr-Nb	Ba-Cr-Nd	Ba-Cr-Ni	Ba-Cr-P	Ba-Cr-Pb
Ba-Cr-Pd	Ba-Cr-Pr	Ba-Cr-Pt	Ba-Cr-Ru	Ba-Cr-Sb	Ba-Cr-Sc	Ba-Cr-Si	Ba-Cr-Sm
Ba-Cr-Sn	Ba-Cr-Sr	Ba-Cr-Ta	Ba-Cr-Tb	Ba-Cr-Ti	Ba-Cr-V	Ba-Cr-W	Ba-Cr-Y
Ba-Cr-Yb	Ba-Cr-Zn	Ba-Cr-Zr	Ba-Cu-Dy	Ba-Cu-Er	Ba-Cu-Fe	Ba-Cu-Ga	Ba-Cu-Gd
Ba-Cu-Ge	Ba-Cu-Hf	Ba-Cu-Mg	Ba-Cu-Mn	Ba-Cu-Mo	Ba-Cu-Nb	Ba-Cu-Nd	Ba-Cu-Ni
Ba-Cu-P	Ba-Cu-Pb	Ba-Cu-Pd	Ba-Cu-Pr	Ba-Cu-Pt	Ba-Cu-Ru	Ba-Cu-Sb	Ba-Cu-Sc
Ba-Cu-Si	Ba-Cu-Sm	Ba-Cu-Sn	Ba-Cu-Sr	Ba-Cu-Ta	Ba-Cu-Tb	Ba-Cu-Ti	Ba-Cu-V
Ba-Cu-W	Ba-Cu-Zn	Ba-Cu-Zr	Ba-Dy-Er	Ba-Dy-Fe	Ba-Dy-Ga	Ba-Dy-Gd	Ba-Dy-Ge
Ba-Dy-Hf	Ba-Dy-La	Ba-Dy-Mg	Ba-Dy-Mn	Ba-Dy-Mo	Ba-Dy-Nb	Ba-Dy-Nd	Ba-Dy-Ni
Ba-Dy-P	Ba-Dy-Pb	Ba-Dy-Pd	Ba-Dy-Pr	Ba-Dy-Pt	Ba-Dy-Ru	Ba-Dy-Sb	Ba-Dy-Sc
Ba-Dy-Si	Ba-Dy-Sm	Ba-Dy-Sn	Ba-Dy-Sr	Ba-Dy-Ta	Ba-Dy-Tb	Ba-Dy-Ti	Ba-Dy-V
Ba-Dy-W	Ba-Dy-Y	Ba-Dy-Yb	Ba-Dy-Zn	Ba-Dy-Zr	Ba-Er-Fe	Ba-Er-Ga	Ba-Er-Gd
Ba-Er-Ge	Ba-Er-Hf	Ba-Er-La	Ba-Er-Mg	Ba-Er-Mn	Ba-Er-Mo	Ba-Er-Nb	Ba-Er-Nd
Ba-Er-Ni	Ba-Er-P	Ba-Er-Pb	Ba-Er-Pd	Ba-Er-Pr	Ba-Er-Pt	Ba-Er-Ru	Ba-Er-Sb
Ba-Er-Sc	Ba-Er-Si	Ba-Er-Sm	Ba-Er-Sn	Ba-Er-Sr	Ba-Er-Ta	Ba-Er-Tb	Ba-Er-Ti
Ba-Er-V	Ba-Er-W	Ba-Er-Y	Ba-Er-Yb	Ba-Er-Zn	Ba-Er-Zr	Ba-Fe-Ga	Ba-Fe-Gd
Ba-Fe-Ge	Ba-Fe-Hf	Ba-Fe-La	Ba-Fe-Mg	Ba-Fe-Mn	Ba-Fe-Mo	Ba-Fe-Nb	Ba-Fe-Nd
Ba-Fe-Ni	Ba-Fe-P	Ba-Fe-Pb	Ba-Fe-Pd	Ba-Fe-Pr	Ba-Fe-Pt	Ba-Fe-Ru	Ba-Fe-Sb
Ba-Fe-Sc	Ba-Fe-Si	Ba-Fe-Sm	Ba-Fe-Sn	Ba-Fe-Sr	Ba-Fe-Ta	Ba-Fe-Tb	Ba-Fe-Ti
Ba-Fe-V	Ba-Fe-W	Ba-Fe-Y	Ba-Fe-Yb	Ba-Fe-Zn	Ba-Fe-Zr	Ba-Ga-Gd	Ba-Ga-Ge
Ba-Ga-Hf	Ba-Ga-La	Ba-Ga-Mg	Ba-Ga-Mn	Ba-Ga-Mo	Ba-Ga-Nb	Ba-Ga-Nd	Ba-Ga-Ni
Ba-Ga-P	Ba-Ga-Pb	Ba-Ga-Pd	Ba-Ga-Pr	Ba-Ga-Pt	Ba-Ga-Ru	Ba-Ga-Sb	Ba-Ga-Sc

Ba-Ga-Si	Ba-Ga-Sm	Ba-Ga-Sn	Ba-Ga-Sr	Ba-Ga-Ta	Ba-Ga-Tb	Ba-Ga-Ti	Ba-Ga-V
Ba-Ga-W	Ba-Ga-Y	Ba-Ga-Yb	Ba-Ga-Zn	Ba-Ga-Zr	Ba-Gd-Ge	Ba-Gd-Hf	Ba-Gd-La
Ba-Gd-Mg	Ba-Gd-Mn	Ba-Gd-Mo	Ba-Gd-Nb	Ba-Gd-Nd	Ba-Gd-Ni	Ba-Gd-P	Ba-Gd-Pb
Ba-Gd-Pd	Ba-Gd-Pr	Ba-Gd-Pt	Ba-Gd-Ru	Ba-Gd-Sb	Ba-Gd-Sc	Ba-Gd-Si	Ba-Gd-Sm
Ba-Gd-Sn	Ba-Gd-Sr	Ba-Gd-Ta	Ba-Gd-Tb	Ba-Gd-Ti	Ba-Gd-V	Ba-Gd-W	Ba-Gd-Y
Ba-Gd-Yb	Ba-Gd-Zn	Ba-Gd-Zr	Ba-Ge-Hf	Ba-Ge-La	Ba-Ge-Mg	Ba-Ge-Mn	Ba-Ge-Mo
Ba-Ge-Nb	Ba-Ge-Nd	Ba-Ge-Ni	Ba-Ge-P	Ba-Ge-Pb	Ba-Ge-Pd	Ba-Ge-Pr	Ba-Ge-Pt
Ba-Ge-Ru	Ba-Ge-Sb	Ba-Ge-Sc	Ba-Ge-Si	Ba-Ge-Sm	Ba-Ge-Sn	Ba-Ge-Sr	Ba-Ge-Ta
Ba-Ge-Tb	Ba-Ge-Ti	Ba-Ge-V	Ba-Ge-W	Ba-Ge-Y	Ba-Ge-Yb	Ba-Ge-Zn	Ba-Ge-Zr
Ba-Hf-La	Ba-Hf-Mg	Ba-Hf-Mn	Ba-Hf-Mo	Ba-Hf-Nb	Ba-Hf-Nd	Ba-Hf-Ni	Ba-Hf-P
Ba-Hf-Pb	Ba-Hf-Pd	Ba-Hf-Pr	Ba-Hf-Pt	Ba-Hf-Ru	Ba-Hf-Sb	Ba-Hf-Sc	Ba-Hf-Si
Ba-Hf-Sm	Ba-Hf-Sn	Ba-Hf-Sr	Ba-Hf-Ta	Ba-Hf-Tb	Ba-Hf-Ti	Ba-Hf-V	Ba-Hf-W
Ba-Hf-Y	Ba-Hf-Yb	Ba-Hf-Zn	Ba-Hf-Zr	Ba-La-Mg	Ba-La-Mn	Ba-La-Mo	Ba-La-Nb
Ba-La-Nd	Ba-La-Ni	Ba-La-P	Ba-La-Pb	Ba-La-Pd	Ba-La-Pr	Ba-La-Pt	Ba-La-Ru
Ba-La-Sb	Ba-La-Sc	Ba-La-Si	Ba-La-Sm	Ba-La-Sn	Ba-La-Sr	Ba-La-Ta	Ba-La-Tb
Ba-La-Ti	Ba-La-V	Ba-La-W	Ba-La-Y	Ba-La-Yb	Ba-La-Zn	Ba-La-Zr	Ba-Mg-Mn
Ba-Mg-Mo	Ba-Mg-Nb	Ba-Mg-Nd	Ba-Mg-Ni	Ba-Mg-P	Ba-Mg-Pb	Ba-Mg-Pd	Ba-Mg-Pr
Ba-Mg-Pt	Ba-Mg-Ru	Ba-Mg-Sb	Ba-Mg-Sc	Ba-Mg-Si	Ba-Mg-Sm	Ba-Mg-Sn	Ba-Mg-Sr
Ba-Mg-Ta	Ba-Mg-Tb	Ba-Mg-Ti	Ba-Mg-V	Ba-Mg-W	Ba-Mg-Y	Ba-Mg-Yb	Ba-Mg-Zn
Ba-Mg-Zr	Ba-Mn-Mo	Ba-Mn-Nb	Ba-Mn-Nd	Ba-Mn-Ni	Ba-Mn-P	Ba-Mn-Pb	Ba-Mn-Pd
Ba-Mn-Pr	Ba-Mn-Pt	Ba-Mn-Ru	Ba-Mn-Sb	Ba-Mn-Sc	Ba-Mn-Si	Ba-Mn-Sm	Ba-Mn-Sn
Ba-Mn-Sr	Ba-Mn-Ta	Ba-Mn-Tb	Ba-Mn-Ti	Ba-Mn-V	Ba-Mn-W	Ba-Mn-Y	Ba-Mn-Yb
Ba-Mn-Zn	Ba-Mn-Zr	Ba-Mo-Nb	Ba-Mo-Nd	Ba-Mo-Ni	Ba-Mo-P	Ba-Mo-Pb	Ba-Mo-Pd
Ba-Mo-Pr	Ba-Mo-Pt	Ba-Mo-Ru	Ba-Mo-Sb	Ba-Mo-Sc	Ba-Mo-Si	Ba-Mo-Sm	Ba-Mo-Sn
Ba-Mo-Sr	Ba-Mo-Ta	Ba-Mo-Tb	Ba-Mo-Ti	Ba-Mo-V	Ba-Mo-W	Ba-Mo-Y	Ba-Mo-Yb

Ba-Mo-Zn	Ba-Mo-Zr	Ba-Nb-Nd	Ba-Nb-Ni	Ba-Nb-P	Ba-Nb-Pb	Ba-Nb-Pd	Ba-Nb-Pr
Ba-Nb-Pt	Ba-Nb-Ru	Ba-Nb-Sb	Ba-Nb-Sc	Ba-Nb-Si	Ba-Nb-Sm	Ba-Nb-Sn	Ba-Nb-Sr
Ba-Nb-Ta	Ba-Nb-Tb	Ba-Nb-Ti	Ba-Nb-V	Ba-Nb-W	Ba-Nb-Y	Ba-Nb-Yb	Ba-Nb-Zn
Ba-Nb-Zr	Ba-Nd-Ni	Ba-Nd-P	Ba-Nd-Pb	Ba-Nd-Pd	Ba-Nd-Pr	Ba-Nd-Pt	Ba-Nd-Ru
Ba-Nd-Sb	Ba-Nd-Sc	Ba-Nd-Si	Ba-Nd-Sm	Ba-Nd-Sn	Ba-Nd-Sr	Ba-Nd-Ta	Ba-Nd-Tb
Ba-Nd-Ti	Ba-Nd-V	Ba-Nd-W	Ba-Nd-Y	Ba-Nd-Yb	Ba-Nd-Zn	Ba-Nd-Zr	Ba-Ni-P
Ba-Ni-Pb	Ba-Ni-Pd	Ba-Ni-Pr	Ba-Ni-Pt	Ba-Ni-Ru	Ba-Ni-Sb	Ba-Ni-Sc	Ba-Ni-Si
Ba-Ni-Sm	Ba-Ni-Sn	Ba-Ni-Sr	Ba-Ni-Ta	Ba-Ni-Tb	Ba-Ni-Ti	Ba-Ni-V	Ba-Ni-W
Ba-Ni-Y	Ba-Ni-Yb	Ba-Ni-Zn	Ba-Ni-Zr	Ba-P-Pb	Ba-P-Pd	Ba-P-Pr	Ba-P-Pt
Ba-P-Ru	Ba-P-Sb	Ba-P-Sc	Ba-P-Si	Ba-P-Sm	Ba-P-Sn	Ba-P-Sr	Ba-P-Ta
Ba-P-Tb	Ba-P-Ti	Ba-P-V	Ba-P-W	Ba-P-Y	Ba-P-Yb	Ba-P-Zn	Ba-P-Zr
Ba-Pb-Pd	Ba-Pb-Pr	Ba-Pb-Pt	Ba-Pb-Ru	Ba-Pb-Sb	Ba-Pb-Sc	Ba-Pb-Si	Ba-Pb-Sm
Ba-Pb-Sn	Ba-Pb-Sr	Ba-Pb-Ta	Ba-Pb-Tb	Ba-Pb-Ti	Ba-Pb-V	Ba-Pb-W	Ba-Pb-Y
Ba-Pb-Yb	Ba-Pb-Zn	Ba-Pb-Zr	Ba-Pd-Pr	Ba-Pd-Pt	Ba-Pd-Ru	Ba-Pd-Sb	Ba-Pd-Sc
Ba-Pd-Si	Ba-Pd-Sm	Ba-Pd-Sn	Ba-Pd-Sr	Ba-Pd-Ta	Ba-Pd-Tb	Ba-Pd-Ti	Ba-Pd-V
Ba-Pd-W	Ba-Pd-Y	Ba-Pd-Yb	Ba-Pd-Zn	Ba-Pd-Zr	Ba-Pr-Pt	Ba-Pr-Ru	Ba-Pr-Sb
Ba-Pr-Sc	Ba-Pr-Si	Ba-Pr-Sm	Ba-Pr-Sn	Ba-Pr-Sr	Ba-Pr-Ta	Ba-Pr-Tb	Ba-Pr-Ti
Ba-Pr-V	Ba-Pr-W	Ba-Pr-Y	Ba-Pr-Yb	Ba-Pr-Zn	Ba-Pr-Zr	Ba-Pt-Ru	Ba-Pt-Sb
Ba-Pt-Sc	Ba-Pt-Si	Ba-Pt-Sm	Ba-Pt-Sn	Ba-Pt-Sr	Ba-Pt-Ta	Ba-Pt-Tb	Ba-Pt-Ti
Ba-Pt-V	Ba-Pt-W	Ba-Pt-Y	Ba-Pt-Yb	Ba-Pt-Zn	Ba-Pt-Zr	Ba-Ru-Sb	Ba-Ru-Sc
Ba-Ru-Si	Ba-Ru-Sm	Ba-Ru-Sn	Ba-Ru-Sr	Ba-Ru-Ta	Ba-Ru-Tb	Ba-Ru-Ti	Ba-Ru-V
Ba-Ru-W	Ba-Ru-Y	Ba-Ru-Yb	Ba-Ru-Zn	Ba-Ru-Zr	Ba-Sb-Sc	Ba-Sb-Si	Ba-Sb-Sm
Ba-Sb-Sn	Ba-Sb-Sr	Ba-Sb-Ta	Ba-Sb-Tb	Ba-Sb-Ti	Ba-Sb-V	Ba-Sb-W	Ba-Sb-Y
Ba-Sb-Yb	Ba-Sb-Zn	Ba-Sb-Zr	Ba-Sc-Si	Ba-Sc-Sm	Ba-Sc-Sn	Ba-Sc-Sr	Ba-Sc-Ta
Ba-Sc-Tb	Ba-Sc-Ti	Ba-Sc-V	Ba-Sc-W	Ba-Sc-Y	Ba-Sc-Yb	Ba-Sc-Zn	Ba-Sc-Zr

Ba-Si-Sm	Ba-Si-Sn	Ba-Si-Sr	Ba-Si-Ta	Ba-Si-Tb	Ba-Si-Ti	Ba-Si-V	Ba-Si-W
Ba-Si-Y	Ba-Si-Yb	Ba-Si-Zn	Ba-Si-Zr	Ba-Sm-Sn	Ba-Sm-Sr	Ba-Sm-Ta	Ba-Sm-Tb
Ba-Sm-Ti	Ba-Sm-V	Ba-Sm-W	Ba-Sm-Y	Ba-Sm-Yb	Ba-Sm-Zn	Ba-Sm-Zr	Ba-Sn-Sr
Ba-Sn-Ta	Ba-Sn-Tb	Ba-Sn-Ti	Ba-Sn-V	Ba-Sn-W	Ba-Sn-Y	Ba-Sn-Yb	Ba-Sn-Zn
Ba-Sn-Zr	Ba-Sr-Ta	Ba-Sr-Tb	Ba-Sr-Ti	Ba-Sr-V	Ba-Sr-W	Ba-Sr-Y	Ba-Sr-Yb
Ba-Sr-Zn	Ba-Sr-Zr	Ba-Ta-Tb	Ba-Ta-Ti	Ba-Ta-V	Ba-Ta-W	Ba-Ta-Y	Ba-Ta-Yb
Ba-Ta-Zn	Ba-Ta-Zr	Ba-Tb-Ti	Ba-Tb-V	Ba-Tb-W	Ba-Tb-Y	Ba-Tb-Yb	Ba-Tb-Zn
Ba-Tb-Zr	Ba-Ti-V	Ba-Ti-W	Ba-Ti-Y	Ba-Ti-Yb	Ba-Ti-Zn	Ba-Ti-Zr	Ba-V-W
Ba-V-Y	Ba-V-Yb	Ba-V-Zn	Ba-V-Zr	Ba-W-Y	Ba-W-Yb	Ba-W-Zn	Ba-W-Zr
Ba-Y-Yb	Ba-Y-Zn	Ba-Y-Zr	Ba-Yb-Zn	Ba-Yb-Zr	Ba-Zn-Zr	Be-C-Ca	Be-C-Ce
Be-C-Co	Be-C-Cr	Be-C-Cu	Be-C-Dy	Be-C-Er	Be-C-Fe	Be-C-Ga	Be-C-Gd
Be-C-Ge	Be-C-Hf	Be-C-La	Be-C-Mg	Be-C-Mn	Be-C-Mo	Be-C-Nb	Be-C-Nd
Be-C-Ni	Be-C-P	Be-C-Pb	Be-C-Pd	Be-C-Pr	Be-C-Pt	Be-C-Ru	Be-C-Sb
Be-C-Sc	Be-C-Si	Be-C-Sm	Be-C-Sn	Be-C-Sr	Be-C-Ta	Be-C-Tb	Be-C-Ti
Be-C-V	Be-C-W	Be-C-Y	Be-C-Yb	Be-C-Zn	Be-C-Zr	Be-Ca-Ce	Be-Ca-Co
Be-Ca-Cr	Be-Ca-Cu	Be-Ca-Dy	Be-Ca-Er	Be-Ca-Fe	Be-Ca-Ga	Be-Ca-Gd	Be-Ca-Ge
Be-Ca-Hf	Be-Ca-La	Be-Ca-Mg	Be-Ca-Mn	Be-Ca-Mo	Be-Ca-Nb	Be-Ca-Nd	Be-Ca-Ni
Be-Ca-P	Be-Ca-Pb	Be-Ca-Pd	Be-Ca-Pr	Be-Ca-Pt	Be-Ca-Ru	Be-Ca-Sb	Be-Ca-Sc
Be-Ca-Si	Be-Ca-Sm	Be-Ca-Sn	Be-Ca-Sr	Be-Ca-Ta	Be-Ca-Tb	Be-Ca-Ti	Be-Ca-V
Be-Ca-W	Be-Ca-Y	Be-Ca-Yb	Be-Ca-Zn	Be-Ca-Zr	Be-Ce-Co	Be-Ce-Cr	Be-Ce-Cu
Be-Ce-Dy	Be-Ce-Er	Be-Ce-Fe	Be-Ce-Ga	Be-Ce-Gd	Be-Ce-Ge	Be-Ce-Hf	Be-Ce-La
Be-Ce-Mg	Be-Ce-Mn	Be-Ce-Mo	Be-Ce-Nb	Be-Ce-Nd	Be-Ce-Ni	Be-Ce-P	Be-Ce-Pb
Be-Ce-Pd	Be-Ce-Pr	Be-Ce-Pt	Be-Ce-Ru	Be-Ce-Sb	Be-Ce-Sc	Be-Ce-Si	Be-Ce-Sm
Be-Ce-Sn	Be-Ce-Sr	Be-Ce-Ta	Be-Ce-Tb	Be-Ce-Ti	Be-Ce-V	Be-Ce-W	Be-Ce-Y
Be-Ce-Yb	Be-Ce-Zn	Be-Ce-Zr	Be-Co-Cr	Be-Co-Cu	Be-Co-Dy	Be-Co-Er	Be-Co-Fe

Be-Co-Ga	Be-Co-Gd	Be-Co-Ge	Be-Co-Hf	Be-Co-La	Be-Co-Mg	Be-Co-Mn	Be-Co-Mo
Be-Co-Nb	Be-Co-Nd	Be-Co-Ni	Be-Co-P	Be-Co-Pb	Be-Co-Pd	Be-Co-Pr	Be-Co-Pt
Be-Co-Ru	Be-Co-Sb	Be-Co-Sc	Be-Co-Si	Be-Co-Sm	Be-Co-Sn	Be-Co-Sr	Be-Co-Ta
Be-Co-Tb	Be-Co-Ti	Be-Co-V	Be-Co-W	Be-Co-Y	Be-Co-Yb	Be-Co-Zn	Be-Co-Zr
Be-Cr-Cu	Be-Cr-Dy	Be-Cr-Er	Be-Cr-Fe	Be-Cr-Ga	Be-Cr-Gd	Be-Cr-Ge	Be-Cr-Hf
Be-Cr-La	Be-Cr-Mg	Be-Cr-Mn	Be-Cr-Mo	Be-Cr-Nb	Be-Cr-Nd	Be-Cr-Ni	Be-Cr-P
Be-Cr-Pb	Be-Cr-Pd	Be-Cr-Pr	Be-Cr-Pt	Be-Cr-Ru	Be-Cr-Sb	Be-Cr-Sc	Be-Cr-Si
Be-Cr-Sm	Be-Cr-Sn	Be-Cr-Sr	Be-Cr-Ta	Be-Cr-Tb	Be-Cr-Ti	Be-Cr-V	Be-Cr-W
Be-Cr-Y	Be-Cr-Yb	Be-Cr-Zn	Be-Cr-Zr	Be-Cu-Dy	Be-Cu-Er	Be-Cu-Ga	Be-Cu-Gd
Be-Cu-Ge	Be-Cu-La	Be-Cu-Mg	Be-Cu-Mn	Be-Cu-Mo	Be-Cu-Nd	Be-Cu-Ni	Be-Cu-P
Be-Cu-Pb	Be-Cu-Pd	Be-Cu-Pr	Be-Cu-Pt	Be-Cu-Ru	Be-Cu-Sb	Be-Cu-Sc	Be-Cu-Sm
Be-Cu-Sn	Be-Cu-Sr	Be-Cu-Ta	Be-Cu-Tb	Be-Cu-V	Be-Cu-W	Be-Cu-Y	Be-Cu-Yb
Be-Cu-Zn	Be-Dy-Er	Be-Dy-Fe	Be-Dy-Ga	Be-Dy-Gd	Be-Dy-Ge	Be-Dy-Hf	Be-Dy-La
Be-Dy-Mg	Be-Dy-Mn	Be-Dy-Mo	Be-Dy-Nb	Be-Dy-Nd	Be-Dy-Ni	Be-Dy-P	Be-Dy-Pb
Be-Dy-Pd	Be-Dy-Pr	Be-Dy-Pt	Be-Dy-Ru	Be-Dy-Sb	Be-Dy-Sc	Be-Dy-Si	Be-Dy-Sm
Be-Dy-Sn	Be-Dy-Sr	Be-Dy-Ta	Be-Dy-Tb	Be-Dy-Ti	Be-Dy-V	Be-Dy-W	Be-Dy-Y
Be-Dy-Yb	Be-Dy-Zn	Be-Dy-Zr	Be-Er-Fe	Be-Er-Ga	Be-Er-Gd	Be-Er-Ge	Be-Er-Hf
Be-Er-La	Be-Er-Mg	Be-Er-Mn	Be-Er-Mo	Be-Er-Nb	Be-Er-Nd	Be-Er-Ni	Be-Er-P
Be-Er-Pb	Be-Er-Pd	Be-Er-Pr	Be-Er-Pt	Be-Er-Ru	Be-Er-Sb	Be-Er-Sc	Be-Er-Si
Be-Er-Sm	Be-Er-Sn	Be-Er-Sr	Be-Er-Ta	Be-Er-Tb	Be-Er-Ti	Be-Er-V	Be-Er-W
Be-Er-Y	Be-Er-Yb	Be-Er-Zn	Be-Er-Zr	Be-Fe-Ga	Be-Fe-Gd	Be-Fe-Ge	Be-Fe-La
Be-Fe-Mg	Be-Fe-Mn	Be-Fe-Mo	Be-Fe-Nd	Be-Fe-Ni	Be-Fe-P	Be-Fe-Pb	Be-Fe-Pd
Be-Fe-Pr	Be-Fe-Pt	Be-Fe-Ru	Be-Fe-Sb	Be-Fe-Sc	Be-Fe-Sm	Be-Fe-Sn	Be-Fe-Sr
Be-Fe-Ta	Be-Fe-Tb	Be-Fe-V	Be-Fe-W	Be-Fe-Y	Be-Fe-Yb	Be-Fe-Zn	Be-Ga-Gd
Be-Ga-Ge	Be-Ga-Hf	Be-Ga-La	Be-Ga-Mg	Be-Ga-Mn	Be-Ga-Mo	Be-Ga-Nb	Be-Ga-Nd

Be-Ga-Ni	Be-Ga-P	Be-Ga-Pb	Be-Ga-Pd	Be-Ga-Pr	Be-Ga-Pt	Be-Ga-Ru	Be-Ga-Sb
Be-Ga-Sc	Be-Ga-Si	Be-Ga-Sm	Be-Ga-Sn	Be-Ga-Sr	Be-Ga-Ta	Be-Ga-Tb	Be-Ga-Ti
Be-Ga-V	Be-Ga-W	Be-Ga-Y	Be-Ga-Yb	Be-Ga-Zn	Be-Ga-Zr	Be-Gd-Ge	Be-Gd-Hf
Be-Gd-La	Be-Gd-Mg	Be-Gd-Mn	Be-Gd-Mo	Be-Gd-Nb	Be-Gd-Nd	Be-Gd-Ni	Be-Gd-P
Be-Gd-Pb	Be-Gd-Pd	Be-Gd-Pr	Be-Gd-Pt	Be-Gd-Ru	Be-Gd-Sb	Be-Gd-Sc	Be-Gd-Si
Be-Gd-Sm	Be-Gd-Sn	Be-Gd-Sr	Be-Gd-Ta	Be-Gd-Tb	Be-Gd-Ti	Be-Gd-V	Be-Gd-W
Be-Gd-Y	Be-Gd-Yb	Be-Gd-Zn	Be-Gd-Zr	Be-Ge-Hf	Be-Ge-La	Be-Ge-Mg	Be-Ge-Mn
Be-Ge-Mo	Be-Ge-Nb	Be-Ge-Nd	Be-Ge-Ni	Be-Ge-P	Be-Ge-Pb	Be-Ge-Pd	Be-Ge-Pr
Be-Ge-Pt	Be-Ge-Ru	Be-Ge-Sb	Be-Ge-Sc	Be-Ge-Si	Be-Ge-Sm	Be-Ge-Sn	Be-Ge-Sr
Be-Ge-Ta	Be-Ge-Tb	Be-Ge-Ti	Be-Ge-V	Be-Ge-W	Be-Ge-Y	Be-Ge-Yb	Be-Ge-Zn
Be-Ge-Zr	Be-Hf-La	Be-Hf-Mg	Be-Hf-Mn	Be-Hf-Mo	Be-Hf-Nd	Be-Hf-Ni	Be-Hf-P
Be-Hf-Pb	Be-Hf-Pd	Be-Hf-Pr	Be-Hf-Pt	Be-Hf-Ru	Be-Hf-Sb	Be-Hf-Sc	Be-Hf-Sm
Be-Hf-Sn	Be-Hf-Sr	Be-Hf-Ta	Be-Hf-Tb	Be-Hf-V	Be-Hf-W	Be-Hf-Y	Be-Hf-Yb
Be-Hf-Zn	Be-La-Mg	Be-La-Mn	Be-La-Mo	Be-La-Nb	Be-La-Nd	Be-La-Ni	Be-La-P
Be-La-Pb	Be-La-Pd	Be-La-Pr	Be-La-Pt	Be-La-Ru	Be-La-Sb	Be-La-Sc	Be-La-Si
Be-La-Sm	Be-La-Sn	Be-La-Sr	Be-La-Ta	Be-La-Tb	Be-La-Ti	Be-La-V	Be-La-W
Be-La-Y	Be-La-Yb	Be-La-Zn	Be-La-Zr	Be-Mg-Mn	Be-Mg-Mo	Be-Mg-Nb	Be-Mg-Nd
Be-Mg-Ni	Be-Mg-P	Be-Mg-Pb	Be-Mg-Pd	Be-Mg-Pr	Be-Mg-Pt	Be-Mg-Ru	Be-Mg-Sb
Be-Mg-Sc	Be-Mg-Si	Be-Mg-Sm	Be-Mg-Sn	Be-Mg-Sr	Be-Mg-Ta	Be-Mg-Tb	Be-Mg-Ti
Be-Mg-V	Be-Mg-W	Be-Mg-Y	Be-Mg-Yb	Be-Mg-Zn	Be-Mg-Zr	Be-Mn-Mo	Be-Mn-Nb
Be-Mn-Nd	Be-Mn-Ni	Be-Mn-P	Be-Mn-Pb	Be-Mn-Pd	Be-Mn-Pr	Be-Mn-Pt	Be-Mn-Ru
Be-Mn-Sb	Be-Mn-Sc	Be-Mn-Si	Be-Mn-Sm	Be-Mn-Sn	Be-Mn-Sr	Be-Mn-Ta	Be-Mn-Tb
Be-Mn-Ti	Be-Mn-V	Be-Mn-W	Be-Mn-Y	Be-Mn-Yb	Be-Mn-Zn	Be-Mn-Zr	Be-Mo-Nb
Be-Mo-Nd	Be-Mo-Ni	Be-Mo-P	Be-Mo-Pb	Be-Mo-Pd	Be-Mo-Pr	Be-Mo-Pt	Be-Mo-Ru
Be-Mo-Sb	Be-Mo-Sc	Be-Mo-Si	Be-Mo-Sm	Be-Mo-Sn	Be-Mo-Sr	Be-Mo-Ta	Be-Mo-Tb

Be-Mo-Ti	Be-Mo-V	Be-Mo-W	Be-Mo-Y	Be-Mo-Yb	Be-Mo-Zn	Be-Mo-Zr	Be-Nb-Nd
Be-Nb-Ni	Be-Nb-P	Be-Nb-Pb	Be-Nb-Pd	Be-Nb-Pr	Be-Nb-Pt	Be-Nb-Ru	Be-Nb-Sb
Be-Nb-Sc	Be-Nb-Sm	Be-Nb-Sn	Be-Nb-Sr	Be-Nb-Ta	Be-Nb-Tb	Be-Nb-V	Be-Nb-W
Be-Nb-Y	Be-Nb-Yb	Be-Nb-Zn	Be-Nd-Ni	Be-Nd-P	Be-Nd-Pb	Be-Nd-Pd	Be-Nd-Pr
Be-Nd-Pt	Be-Nd-Ru	Be-Nd-Sb	Be-Nd-Sc	Be-Nd-Si	Be-Nd-Sm	Be-Nd-Sn	Be-Nd-Sr
Be-Nd-Ta	Be-Nd-Tb	Be-Nd-Ti	Be-Nd-V	Be-Nd-W	Be-Nd-Y	Be-Nd-Yb	Be-Nd-Zn
Be-Nd-Zr	Be-Ni-P	Be-Ni-Pb	Be-Ni-Pd	Be-Ni-Pr	Be-Ni-Pt	Be-Ni-Ru	Be-Ni-Sb
Be-Ni-Sc	Be-Ni-Si	Be-Ni-Sm	Be-Ni-Sn	Be-Ni-Sr	Be-Ni-Ta	Be-Ni-Tb	Be-Ni-Ti
Be-Ni-V	Be-Ni-W	Be-Ni-Y	Be-Ni-Yb	Be-Ni-Zn	Be-Ni-Zr	Be-P-Pb	Be-P-Pd
Be-P-Pr	Be-P-Pt	Be-P-Ru	Be-P-Sb	Be-P-Sc	Be-P-Si	Be-P-Sm	Be-P-Sn
Be-P-Sr	Be-P-Ta	Be-P-Tb	Be-P-Ti	Be-P-V	Be-P-W	Be-P-Y	Be-P-Yb
Be-P-Zn	Be-P-Zr	Be-Pb-Pd	Be-Pb-Pr	Be-Pb-Pt	Be-Pb-Ru	Be-Pb-Sb	Be-Pb-Sc
Be-Pb-Si	Be-Pb-Sm	Be-Pb-Sn	Be-Pb-Sr	Be-Pb-Ta	Be-Pb-Tb	Be-Pb-Ti	Be-Pb-V
Be-Pb-W	Be-Pb-Y	Be-Pb-Yb	Be-Pb-Zn	Be-Pb-Zr	Be-Pd-Pr	Be-Pd-Pt	Be-Pd-Ru
Be-Pd-Sb	Be-Pd-Sc	Be-Pd-Si	Be-Pd-Sm	Be-Pd-Sn	Be-Pd-Sr	Be-Pd-Ta	Be-Pd-Tb
Be-Pd-Ti	Be-Pd-V	Be-Pd-W	Be-Pd-Y	Be-Pd-Yb	Be-Pd-Zn	Be-Pd-Zr	Be-Pr-Pt
Be-Pr-Ru	Be-Pr-Sb	Be-Pr-Sc	Be-Pr-Si	Be-Pr-Sm	Be-Pr-Sn	Be-Pr-Sr	Be-Pr-Ta
Be-Pr-Tb	Be-Pr-Ti	Be-Pr-V	Be-Pr-W	Be-Pr-Y	Be-Pr-Yb	Be-Pr-Zn	Be-Pr-Zr
Be-Pt-Ru	Be-Pt-Sb	Be-Pt-Sc	Be-Pt-Si	Be-Pt-Sm	Be-Pt-Sn	Be-Pt-Sr	Be-Pt-Ta
Be-Pt-Tb	Be-Pt-Ti	Be-Pt-V	Be-Pt-W	Be-Pt-Y	Be-Pt-Yb	Be-Pt-Zn	Be-Pt-Zr
Be-Ru-Sb	Be-Ru-Sc	Be-Ru-Si	Be-Ru-Sm	Be-Ru-Sn	Be-Ru-Sr	Be-Ru-Ta	Be-Ru-Tb
Be-Ru-Ti	Be-Ru-V	Be-Ru-W	Be-Ru-Y	Be-Ru-Yb	Be-Ru-Zn	Be-Ru-Zr	Be-Sb-Sc
Be-Sb-Si	Be-Sb-Sm	Be-Sb-Sn	Be-Sb-Sr	Be-Sb-Ta	Be-Sb-Tb	Be-Sb-Ti	Be-Sb-V
Be-Sb-W	Be-Sb-Y	Be-Sb-Yb	Be-Sb-Zn	Be-Sb-Zr	Be-Sc-Si	Be-Sc-Sm	Be-Sc-Sn
Be-Sc-Sr	Be-Sc-Ta	Be-Sc-Tb	Be-Sc-Ti	Be-Sc-V	Be-Sc-W	Be-Sc-Y	Be-Sc-Yb

Be-Sc-Zn	Be-Sc-Zr	Be-Si-Sm	Be-Si-Sn	Be-Si-Sr	Be-Si-Ta	Be-Si-Tb	Be-Si-V
Be-Si-W	Be-Si-Y	Be-Si-Yb	Be-Si-Zn	Be-Sm-Sn	Be-Sm-Sr	Be-Sm-Ta	Be-Sm-Tb
Be-Sm-Ti	Be-Sm-V	Be-Sm-W	Be-Sm-Y	Be-Sm-Yb	Be-Sm-Zn	Be-Sm-Zr	Be-Sn-Sr
Be-Sn-Ta	Be-Sn-Tb	Be-Sn-Ti	Be-Sn-V	Be-Sn-W	Be-Sn-Y	Be-Sn-Yb	Be-Sn-Zn
Be-Sn-Zr	Be-Sr-Ta	Be-Sr-Tb	Be-Sr-Ti	Be-Sr-V	Be-Sr-W	Be-Sr-Y	Be-Sr-Yb
Be-Sr-Zn	Be-Sr-Zr	Be-Ta-Tb	Be-Ta-Ti	Be-Ta-V	Be-Ta-W	Be-Ta-Y	Be-Ta-Yb
Be-Ta-Zn	Be-Ta-Zr	Be-Tb-Ti	Be-Tb-V	Be-Tb-W	Be-Tb-Y	Be-Tb-Yb	Be-Tb-Zn
Be-Tb-Zr	Be-Ti-V	Be-Ti-W	Be-Ti-Y	Be-Ti-Yb	Be-Ti-Zn	Be-V-W	Be-V-Y
Be-V-Yb	Be-V-Zn	Be-V-Zr	Be-W-Y	Be-W-Yb	Be-W-Zn	Be-W-Zr	Be-Y-Yb
Be-Y-Zn	Be-Y-Zr	Be-Yb-Zn	Be-Yb-Zr	Be-Zn-Zr	C-Ca-Ce	C-Ca-Co	C-Ca-Cr
C-Ca-Cu	C-Ca-Dy	C-Ca-Er	C-Ca-Fe	C-Ca-Ga	C-Ca-Gd	C-Ca-Ge	C-Ca-Hf
C-Ca-La	C-Ca-Mg	C-Ca-Mn	C-Ca-Mo	C-Ca-Nb	C-Ca-Nd	C-Ca-Ni	C-Ca-P
C-Ca-Pb	C-Ca-Pd	C-Ca-Pr	C-Ca-Pt	C-Ca-Ru	C-Ca-Sb	C-Ca-Sc	C-Ca-Si
C-Ca-Sm	C-Ca-Sn	C-Ca-Sr	C-Ca-Ta	C-Ca-Tb	C-Ca-Ti	C-Ca-V	C-Ca-W
C-Ca-Y	C-Ca-Yb	C-Ca-Zn	C-Ca-Zr	C-Ce-Co	C-Ce-Cr	C-Ce-Cu	C-Ce-Dy
C-Ce-Er	C-Ce-Fe	C-Ce-Ga	C-Ce-Gd	C-Ce-Ge	C-Ce-Hf	C-Ce-La	C-Ce-Mg
C-Ce-Mn	C-Ce-Mo	C-Ce-Nb	C-Ce-Nd	C-Ce-Ni	C-Ce-P	C-Ce-Pb	C-Ce-Pd
C-Ce-Pr	C-Ce-Pt	C-Ce-Ru	C-Ce-Sb	C-Ce-Sc	C-Ce-Si	C-Ce-Sm	C-Ce-Sn
C-Ce-Sr	C-Ce-Ta	C-Ce-Tb	C-Ce-Ti	C-Ce-V	C-Ce-W	C-Ce-Y	C-Ce-Yb
C-Ce-Zn	C-Ce-Zr	C-Co-Cu	C-Co-Dy	C-Co-Er	C-Co-Ga	C-Co-Gd	C-Co-Ge
C-Co-Hf	C-Co-La	C-Co-Mg	C-Co-Mn	C-Co-Nd	C-Co-Pb	C-Co-Pd	C-Co-Pr
C-Co-Pt	C-Co-Ru	C-Co-Sb	C-Co-Sc	C-Co-Sm	C-Co-Sn	C-Co-Sr	C-Co-Ta
C-Co-Tb	C-Co-Ti	C-Co-V	C-Co-Y	C-Co-Yb	C-Co-Zn	C-Co-Zr	C-Cr-Cu
C-Cr-Dy	C-Cr-Er	C-Cr-Ga	C-Cr-Gd	C-Cr-Ge	C-Cr-Hf	C-Cr-La	C-Cr-Mg
C-Cr-Mn	C-Cr-Nb	C-Cr-Nd	C-Cr-Pb	C-Cr-Pd	C-Cr-Pr	C-Cr-Pt	C-Cr-Ru

C-Cr-Sb	C-Cr-Sc	C-Cr-Sm	C-Cr-Sn	C-Cr-Sr	C-Cr-Ta	C-Cr-Tb	C-Cr-Ti
C-Cr-V	C-Cr-W	C-Cr-Y	C-Cr-Yb	C-Cr-Zn	C-Cr-Zr	C-Cu-Dy	C-Cu-Er
C-Cu-Fe	C-Cu-Ga	C-Cu-Gd	C-Cu-Ge	C-Cu-Hf	C-Cu-La	C-Cu-Mg	C-Cu-Mn
C-Cu-Mo	C-Cu-Nb	C-Cu-Nd	C-Cu-Ni	C-Cu-P	C-Cu-Pb	C-Cu-Pd	C-Cu-Pr
C-Cu-Pt	C-Cu-Ru	C-Cu-Sb	C-Cu-Sc	C-Cu-Si	C-Cu-Sm	C-Cu-Sn	C-Cu-Sr
C-Cu-Ta	C-Cu-Tb	C-Cu-Ti	C-Cu-V	C-Cu-W	C-Cu-Y	C-Cu-Yb	C-Cu-Zn
C-Cu-Zr	C-Dy-Er	C-Dy-Fe	C-Dy-Ga	C-Dy-Gd	C-Dy-Ge	C-Dy-Hf	C-Dy-La
C-Dy-Mg	C-Dy-Mn	C-Dy-Mo	C-Dy-Nb	C-Dy-Nd	C-Dy-Ni	C-Dy-P	C-Dy-Pb
C-Dy-Pd	C-Dy-Pr	C-Dy-Pt	C-Dy-Ru	C-Dy-Sb	C-Dy-Sc	C-Dy-Si	C-Dy-Sm
C-Dy-Sn	C-Dy-Sr	C-Dy-Ta	C-Dy-Tb	C-Dy-Ti	C-Dy-V	C-Dy-W	C-Dy-Y
C-Dy-Yb	C-Dy-Zn	C-Dy-Zr	C-Er-Fe	C-Er-Ga	C-Er-Gd	C-Er-Ge	C-Er-Hf
C-Er-La	C-Er-Mg	C-Er-Mn	C-Er-Mo	C-Er-Nb	C-Er-Nd	C-Er-Ni	C-Er-P
C-Er-Pb	C-Er-Pd	C-Er-Pr	C-Er-Pt	C-Er-Ru	C-Er-Sb	C-Er-Sc	C-Er-Si
C-Er-Sm	C-Er-Sn	C-Er-Sr	C-Er-Ta	C-Er-Tb	C-Er-Ti	C-Er-V	C-Er-W
C-Er-Y	C-Er-Yb	C-Er-Zn	C-Er-Zr	C-Fe-Ga	C-Fe-Gd	C-Fe-Ge	C-Fe-Hf
C-Fe-La	C-Fe-Mg	C-Fe-Mn	C-Fe-Nd	C-Fe-Pb	C-Fe-Pd	C-Fe-Pr	C-Fe-Pt
C-Fe-Ru	C-Fe-Sb	C-Fe-Sc	C-Fe-Sm	C-Fe-Sn	C-Fe-Sr	C-Fe-Ta	C-Fe-Tb
C-Fe-Ti	C-Fe-V	C-Fe-Y	C-Fe-Yb	C-Fe-Zn	C-Fe-Zr	C-Ga-Gd	C-Ga-Ge
C-Ga-Hf	C-Ga-La	C-Ga-Mg	C-Ga-Mn	C-Ga-Mo	C-Ga-Nb	C-Ga-Nd	C-Ga-Ni
C-Ga-P	C-Ga-Pb	C-Ga-Pd	C-Ga-Pr	C-Ga-Pt	C-Ga-Ru	C-Ga-Sb	C-Ga-Sc
C-Ga-Si	C-Ga-Sm	C-Ga-Sn	C-Ga-Sr	C-Ga-Ta	C-Ga-Tb	C-Ga-Ti	C-Ga-V
C-Ga-W	C-Ga-Y	C-Ga-Yb	C-Ga-Zn	C-Ga-Zr	C-Gd-Ge	C-Gd-Hf	C-Gd-La
C-Gd-Mg	C-Gd-Mn	C-Gd-Mo	C-Gd-Nb	C-Gd-Nd	C-Gd-Ni	C-Gd-P	C-Gd-Pb
C-Gd-Pd	C-Gd-Pr	C-Gd-Pt	C-Gd-Ru	C-Gd-Sb	C-Gd-Sc	C-Gd-Si	C-Gd-Sm
C-Gd-Sn	C-Gd-Sr	C-Gd-Ta	C-Gd-Tb	C-Gd-Ti	C-Gd-V	C-Gd-W	C-Gd-Y

C-Gd-Yb	C-Gd-Zn	C-Gd-Zr	C-Ge-Hf	C-Ge-La	C-Ge-Mg	C-Ge-Mn	C-Ge-Mo
C-Ge-Nb	C-Ge-Nd	C-Ge-Ni	C-Ge-P	C-Ge-Pb	C-Ge-Pd	C-Ge-Pr	C-Ge-Pt
C-Ge-Ru	C-Ge-Sb	C-Ge-Sc	C-Ge-Si	C-Ge-Sm	C-Ge-Sn	C-Ge-Sr	C-Ge-Ta
C-Ge-Tb	C-Ge-Ti	C-Ge-V	C-Ge-W	C-Ge-Y	C-Ge-Yb	C-Ge-Zn	C-Ge-Zr
C-Hf-La	C-Hf-Mg	C-Hf-Mn	C-Hf-Mo	C-Hf-Nb	C-Hf-Nd	C-Hf-Ni	C-Hf-P
C-Hf-Pb	C-Hf-Pd	C-Hf-Pr	C-Hf-Pt	C-Hf-Ru	C-Hf-Sb	C-Hf-Sc	C-Hf-Si
C-Hf-Sm	C-Hf-Sn	C-Hf-Sr	C-Hf-Ta	C-Hf-Tb	C-Hf-Ti	C-Hf-V	C-Hf-W
C-Hf-Y	C-Hf-Yb	C-Hf-Zn	C-Hf-Zr	C-La-Mg	C-La-Mn	C-La-Mo	C-La-Nb
C-La-Nd	C-La-Ni	C-La-P	C-La-Pb	C-La-Pd	C-La-Pr	C-La-Pt	C-La-Ru
C-La-Sb	C-La-Sc	C-La-Si	C-La-Sm	C-La-Sn	C-La-Sr	C-La-Ta	C-La-Tb
C-La-Ti	C-La-V	C-La-W	C-La-Y	C-La-Yb	C-La-Zn	C-La-Zr	C-Mg-Mn
C-Mg-Mo	C-Mg-Nb	C-Mg-Nd	C-Mg-Ni	C-Mg-P	C-Mg-Pb	C-Mg-Pd	C-Mg-Pr
C-Mg-Pt	C-Mg-Ru	C-Mg-Sb	C-Mg-Sc	C-Mg-Si	C-Mg-Sm	C-Mg-Sn	C-Mg-Sr
C-Mg-Ta	C-Mg-Tb	C-Mg-Ti	C-Mg-V	C-Mg-W	C-Mg-Y	C-Mg-Yb	C-Mg-Zn
C-Mg-Zr	C-Mn-Mo	C-Mn-Nb	C-Mn-Nd	C-Mn-Ni	C-Mn-P	C-Mn-Pb	C-Mn-Pd
C-Mn-Pr	C-Mn-Pt	C-Mn-Ru	C-Mn-Sb	C-Mn-Sc	C-Mn-Si	C-Mn-Sm	C-Mn-Sn
C-Mn-Sr	C-Mn-Ta	C-Mn-Tb	C-Mn-Ti	C-Mn-V	C-Mn-W	C-Mn-Y	C-Mn-Yb
C-Mn-Zn	C-Mn-Zr	C-Mo-Nd	C-Mo-Pb	C-Mo-Pd	C-Mo-Pr	C-Mo-Pt	C-Mo-Ru
C-Mo-Sb	C-Mo-Sc	C-Mo-Sm	C-Mo-Sn	C-Mo-Sr	C-Mo-Ta	C-Mo-Tb	C-Mo-Ti
C-Mo-V	C-Mo-W	C-Mo-Y	C-Mo-Yb	C-Mo-Zn	C-Mo-Zr	C-Nb-Nd	C-Nb-P
C-Nb-Pb	C-Nb-Pd	C-Nb-Pr	C-Nb-Pt	C-Nb-Ru	C-Nb-Sb	C-Nb-Sc	C-Nb-Sm
C-Nb-Sn	C-Nb-Sr	C-Nb-Ta	C-Nb-Tb	C-Nb-Ti	C-Nb-V	C-Nb-Y	C-Nb-Yb
C-Nb-Zn	C-Nb-Zr	C-Nd-Ni	C-Nd-P	C-Nd-Pb	C-Nd-Pd	C-Nd-Pr	C-Nd-Pt
C-Nd-Ru	C-Nd-Sb	C-Nd-Sc	C-Nd-Si	C-Nd-Sm	C-Nd-Sn	C-Nd-Sr	C-Nd-Ta
C-Nd-Tb	C-Nd-Ti	C-Nd-V	C-Nd-W	C-Nd-Y	C-Nd-Yb	C-Nd-Zn	C-Nd-Zr

C-Ni-Pb	C-Ni-Pd	C-Ni-Pr	C-Ni-Pt	C-Ni-Ru	C-Ni-Sb	C-Ni-Sc	C-Ni-Sm
C-Ni-Sn	C-Ni-Sr	C-Ni-Ta	C-Ni-Tb	C-Ni-Ti	C-Ni-V	C-Ni-Y	C-Ni-Yb
C-Ni-Zn	C-Ni-Zr	C-P-Pb	C-P-Pd	C-P-Pr	C-P-Pt	C-P-Ru	C-P-Sb
C-P-Sc	C-P-Sm	C-P-Sn	C-P-Sr	C-P-Ta	C-P-Tb	C-P-Ti	C-P-V
C-P-Y	C-P-Yb	C-P-Zn	C-P-Zr	C-Pb-Pd	C-Pb-Pr	C-Pb-Pt	C-Pb-Ru
C-Pb-Sb	C-Pb-Sc	C-Pb-Si	C-Pb-Sm	C-Pb-Sn	C-Pb-Sr	C-Pb-Ta	C-Pb-Tb
C-Pb-Ti	C-Pb-V	C-Pb-W	C-Pb-Y	C-Pb-Yb	C-Pb-Zn	C-Pb-Zr	C-Pd-Pr
C-Pd-Pt	C-Pd-Ru	C-Pd-Sb	C-Pd-Sc	C-Pd-Si	C-Pd-Sm	C-Pd-Sn	C-Pd-Sr
C-Pd-Ta	C-Pd-Tb	C-Pd-Ti	C-Pd-V	C-Pd-W	C-Pd-Y	C-Pd-Yb	C-Pd-Zn
C-Pd-Zr	C-Pr-Pt	C-Pr-Ru	C-Pr-Sb	C-Pr-Sc	C-Pr-Si	C-Pr-Sm	C-Pr-Sn
C-Pr-Sr	C-Pr-Ta	C-Pr-Tb	C-Pr-Ti	C-Pr-V	C-Pr-W	C-Pr-Y	C-Pr-Yb
C-Pr-Zn	C-Pr-Zr	C-Pt-Ru	C-Pt-Sb	C-Pt-Sc	C-Pt-Si	C-Pt-Sm	C-Pt-Sn
C-Pt-Sr	C-Pt-Ta	C-Pt-Tb	C-Pt-Ti	C-Pt-V	C-Pt-W	C-Pt-Y	C-Pt-Yb
C-Pt-Zn	C-Pt-Zr	C-Ru-Sb	C-Ru-Sc	C-Ru-Si	C-Ru-Sm	C-Ru-Sn	C-Ru-Sr
C-Ru-Ta	C-Ru-Tb	C-Ru-Ti	C-Ru-V	C-Ru-W	C-Ru-Y	C-Ru-Yb	C-Ru-Zn
C-Ru-Zr	C-Sb-Sc	C-Sb-Si	C-Sb-Sm	C-Sb-Sn	C-Sb-Sr	C-Sb-Ta	C-Sb-Tb
C-Sb-Ti	C-Sb-V	C-Sb-W	C-Sb-Y	C-Sb-Yb	C-Sb-Zn	C-Sb-Zr	C-Sc-Si
C-Sc-Sm	C-Sc-Sn	C-Sc-Sr	C-Sc-Ta	C-Sc-Tb	C-Sc-Ti	C-Sc-V	C-Sc-W
C-Sc-Y	C-Sc-Yb	C-Sc-Zn	C-Sc-Zr	C-Si-Sm	C-Si-Sn	C-Si-Sr	C-Si-Ta
C-Si-Tb	C-Si-Ti	C-Si-V	C-Si-Y	C-Si-Yb	C-Si-Zn	C-Si-Zr	C-Sm-Sn
C-Sm-Sr	C-Sm-Ta	C-Sm-Tb	C-Sm-Ti	C-Sm-V	C-Sm-W	C-Sm-Y	C-Sm-Yb
C-Sm-Zn	C-Sm-Zr	C-Sn-Sr	C-Sn-Ta	C-Sn-Tb	C-Sn-Ti	C-Sn-V	C-Sn-W
C-Sn-Y	C-Sn-Yb	C-Sn-Zn	C-Sn-Zr	C-Sr-Ta	C-Sr-Tb	C-Sr-Ti	C-Sr-V
C-Sr-W	C-Sr-Y	C-Sr-Yb	C-Sr-Zn	C-Sr-Zr	C-Ta-Tb	C-Ta-Ti	C-Ta-V
C-Ta-W	C-Ta-Y	C-Ta-Yb	C-Ta-Zn	C-Ta-Zr	C-Tb-Ti	C-Tb-V	C-Tb-W

C-Tb-Y	C-Tb-Yb	C-Tb-Zn	C-Tb-Zr	C-Ti-V	C-Ti-W	C-Ti-Y	C-Ti-Yb
C-Ti-Zn	C-Ti-Zr	C-V-W	C-V-Y	C-V-Yb	C-V-Zn	C-V-Zr	C-W-Y
C-W-Yb	C-W-Zn	C-W-Zr	C-Y-Yb	C-Y-Zn	C-Y-Zr	C-Yb-Zn	C-Yb-Zr
C-Zn-Zr	Ca-Ce-Co	Ca-Ce-Cr	Ca-Ce-Cu	Ca-Ce-Dy	Ca-Ce-Er	Ca-Ce-Fe	Ca-Ce-Ga
Ca-Ce-Gd	Ca-Ce-Ge	Ca-Ce-Hf	Ca-Ce-La	Ca-Ce-Mg	Ca-Ce-Mn	Ca-Ce-Mo	Ca-Ce-Nb
Ca-Ce-Nd	Ca-Ce-Ni	Ca-Ce-P	Ca-Ce-Pb	Ca-Ce-Pd	Ca-Ce-Pr	Ca-Ce-Pt	Ca-Ce-Ru
Ca-Ce-Sb	Ca-Ce-Sc	Ca-Ce-Si	Ca-Ce-Sm	Ca-Ce-Sn	Ca-Ce-Sr	Ca-Ce-Ta	Ca-Ce-Tb
Ca-Ce-Ti	Ca-Ce-V	Ca-Ce-W	Ca-Ce-Y	Ca-Ce-Yb	Ca-Ce-Zn	Ca-Ce-Zr	Ca-Co-Cr
Ca-Co-Cu	Ca-Co-Dy	Ca-Co-Er	Ca-Co-Ga	Ca-Co-Gd	Ca-Co-Ge	Ca-Co-Hf	Ca-Co-Mg
Ca-Co-Mn	Ca-Co-Mo	Ca-Co-Nb	Ca-Co-Nd	Ca-Co-P	Ca-Co-Pb	Ca-Co-Pr	Ca-Co-Pt
Ca-Co-Ru	Ca-Co-Sb	Ca-Co-Sc	Ca-Co-Si	Ca-Co-Sm	Ca-Co-Sn	Ca-Co-Sr	Ca-Co-Ta
Ca-Co-Tb	Ca-Co-Ti	Ca-Co-V	Ca-Co-W	Ca-Co-Y	Ca-Co-Yb	Ca-Co-Zn	Ca-Co-Zr
Ca-Cr-Cu	Ca-Cr-Dy	Ca-Cr-Er	Ca-Cr-Fe	Ca-Cr-Ga	Ca-Cr-Gd	Ca-Cr-Ge	Ca-Cr-Hf
Ca-Cr-La	Ca-Cr-Mg	Ca-Cr-Mn	Ca-Cr-Mo	Ca-Cr-Nb	Ca-Cr-Nd	Ca-Cr-Ni	Ca-Cr-P
Ca-Cr-Pb	Ca-Cr-Pd	Ca-Cr-Pr	Ca-Cr-Pt	Ca-Cr-Ru	Ca-Cr-Sb	Ca-Cr-Sc	Ca-Cr-Si
Ca-Cr-Sm	Ca-Cr-Sn	Ca-Cr-Sr	Ca-Cr-Ta	Ca-Cr-Tb	Ca-Cr-Ti	Ca-Cr-V	Ca-Cr-W
Ca-Cr-Y	Ca-Cr-Yb	Ca-Cr-Zn	Ca-Cr-Zr	Ca-Cu-Dy	Ca-Cu-Er	Ca-Cu-Gd	Ca-Cu-Ge
Ca-Cu-Hf	Ca-Cu-Mn	Ca-Cu-Mo	Ca-Cu-Nb	Ca-Cu-Nd	Ca-Cu-P	Ca-Cu-Pb	Ca-Cu-Pr
Ca-Cu-Pt	Ca-Cu-Ru	Ca-Cu-Sb	Ca-Cu-Sc	Ca-Cu-Si	Ca-Cu-Sm	Ca-Cu-Sn	Ca-Cu-Sr
Ca-Cu-Ta	Ca-Cu-Tb	Ca-Cu-Ti	Ca-Cu-V	Ca-Cu-W	Ca-Cu-Y	Ca-Cu-Yb	Ca-Cu-Zr
Ca-Dy-Er	Ca-Dy-Fe	Ca-Dy-Ga	Ca-Dy-Gd	Ca-Dy-Ge	Ca-Dy-Hf	Ca-Dy-La	Ca-Dy-Mg
Ca-Dy-Mn	Ca-Dy-Mo	Ca-Dy-Nb	Ca-Dy-Nd	Ca-Dy-Ni	Ca-Dy-P	Ca-Dy-Pb	Ca-Dy-Pd
Ca-Dy-Pr	Ca-Dy-Pt	Ca-Dy-Ru	Ca-Dy-Sb	Ca-Dy-Sc	Ca-Dy-Si	Ca-Dy-Sm	Ca-Dy-Sn
Ca-Dy-Sr	Ca-Dy-Ta	Ca-Dy-Tb	Ca-Dy-Ti	Ca-Dy-V	Ca-Dy-W	Ca-Dy-Y	Ca-Dy-Yb
Ca-Dy-Zn	Ca-Dy-Zr	Ca-Er-Fe	Ca-Er-Ga	Ca-Er-Gd	Ca-Er-Ge	Ca-Er-Hf	Ca-Er-La

Ca-Er-Mg	Ca-Er-Mn	Ca-Er-Mo	Ca-Er-Nb	Ca-Er-Nd	Ca-Er-Ni	Ca-Er-P	Ca-Er-Pb
Ca-Er-Pd	Ca-Er-Pr	Ca-Er-Pt	Ca-Er-Ru	Ca-Er-Sb	Ca-Er-Sc	Ca-Er-Si	Ca-Er-Sm
Ca-Er-Sn	Ca-Er-Sr	Ca-Er-Ta	Ca-Er-Tb	Ca-Er-Ti	Ca-Er-V	Ca-Er-W	Ca-Er-Y
Ca-Er-Yb	Ca-Er-Zn	Ca-Er-Zr	Ca-Fe-Gd	Ca-Fe-Ge	Ca-Fe-Hf	Ca-Fe-Mg	Ca-Fe-Mn
Ca-Fe-Mo	Ca-Fe-Nb	Ca-Fe-Nd	Ca-Fe-P	Ca-Fe-Pb	Ca-Fe-Pr	Ca-Fe-Pt	Ca-Fe-Ru
Ca-Fe-Sb	Ca-Fe-Sc	Ca-Fe-Si	Ca-Fe-Sm	Ca-Fe-Sn	Ca-Fe-Sr	Ca-Fe-Ta	Ca-Fe-Tb
Ca-Fe-Ti	Ca-Fe-V	Ca-Fe-W	Ca-Fe-Y	Ca-Fe-Yb	Ca-Fe-Zn	Ca-Fe-Zr	Ca-Ga-Gd
Ca-Ga-Ge	Ca-Ga-Hf	Ca-Ga-La	Ca-Ga-Mn	Ca-Ga-Mo	Ca-Ga-Nb	Ca-Ga-Nd	Ca-Ga-Ni
Ca-Ga-P	Ca-Ga-Pb	Ca-Ga-Pd	Ca-Ga-Pr	Ca-Ga-Pt	Ca-Ga-Ru	Ca-Ga-Sb	Ca-Ga-Sc
Ca-Ga-Si	Ca-Ga-Sm	Ca-Ga-Sn	Ca-Ga-Sr	Ca-Ga-Ta	Ca-Ga-Tb	Ca-Ga-Ti	Ca-Ga-V
Ca-Ga-W	Ca-Ga-Y	Ca-Ga-Yb	Ca-Ga-Zn	Ca-Ga-Zr	Ca-Gd-Ge	Ca-Gd-Hf	Ca-Gd-La
Ca-Gd-Mg	Ca-Gd-Mn	Ca-Gd-Mo	Ca-Gd-Nb	Ca-Gd-Nd	Ca-Gd-Ni	Ca-Gd-P	Ca-Gd-Pb
Ca-Gd-Pd	Ca-Gd-Pr	Ca-Gd-Pt	Ca-Gd-Ru	Ca-Gd-Sb	Ca-Gd-Sc	Ca-Gd-Si	Ca-Gd-Sm
Ca-Gd-Sn	Ca-Gd-Sr	Ca-Gd-Ta	Ca-Gd-Tb	Ca-Gd-Ti	Ca-Gd-V	Ca-Gd-W	Ca-Gd-Y
Ca-Gd-Yb	Ca-Gd-Zn	Ca-Gd-Zr	Ca-Ge-Hf	Ca-Ge-La	Ca-Ge-Mg	Ca-Ge-Mn	Ca-Ge-Mo
Ca-Ge-Nb	Ca-Ge-Nd	Ca-Ge-Ni	Ca-Ge-P	Ca-Ge-Pb	Ca-Ge-Pd	Ca-Ge-Pr	Ca-Ge-Pt
Ca-Ge-Ru	Ca-Ge-Sb	Ca-Ge-Sc	Ca-Ge-Si	Ca-Ge-Sm	Ca-Ge-Sn	Ca-Ge-Sr	Ca-Ge-Ta
Ca-Ge-Tb	Ca-Ge-Ti	Ca-Ge-V	Ca-Ge-W	Ca-Ge-Y	Ca-Ge-Yb	Ca-Ge-Zn	Ca-Ge-Zr
Ca-Hf-La	Ca-Hf-Mg	Ca-Hf-Mn	Ca-Hf-Mo	Ca-Hf-Nb	Ca-Hf-Nd	Ca-Hf-Ni	Ca-Hf-P
Ca-Hf-Pb	Ca-Hf-Pd	Ca-Hf-Pr	Ca-Hf-Pt	Ca-Hf-Ru	Ca-Hf-Sb	Ca-Hf-Sc	Ca-Hf-Si
Ca-Hf-Sm	Ca-Hf-Sn	Ca-Hf-Sr	Ca-Hf-Ta	Ca-Hf-Tb	Ca-Hf-Ti	Ca-Hf-V	Ca-Hf-W
Ca-Hf-Y	Ca-Hf-Yb	Ca-Hf-Zn	Ca-Hf-Zr	Ca-La-Mn	Ca-La-Mo	Ca-La-Nb	Ca-La-Nd
Ca-La-P	Ca-La-Pb	Ca-La-Pd	Ca-La-Pr	Ca-La-Pt	Ca-La-Ru	Ca-La-Sb	Ca-La-Sc
Ca-La-Si	Ca-La-Sm	Ca-La-Sn	Ca-La-Sr	Ca-La-Ta	Ca-La-Tb	Ca-La-Ti	Ca-La-V
Ca-La-W	Ca-La-Y	Ca-La-Yb	Ca-La-Zr	Ca-Mg-Mn	Ca-Mg-Mo	Ca-Mg-Nb	Ca-Mg-Nd

Ca-Mg-P	Ca-Mg-Pb	Ca-Mg-Pr	Ca-Mg-Pt	Ca-Mg-Ru	Ca-Mg-Sb	Ca-Mg-Sc	Ca-Mg-Si
Ca-Mg-Sm	Ca-Mg-Sn	Ca-Mg-Sr	Ca-Mg-Ta	Ca-Mg-Tb	Ca-Mg-Ti	Ca-Mg-V	Ca-Mg-W
Ca-Mg-Y	Ca-Mg-Yb	Ca-Mg-Zr	Ca-Mn-Mo	Ca-Mn-Nb	Ca-Mn-Nd	Ca-Mn-Ni	Ca-Mn-P
Ca-Mn-Pb	Ca-Mn-Pd	Ca-Mn-Pr	Ca-Mn-Pt	Ca-Mn-Ru	Ca-Mn-Sb	Ca-Mn-Sc	Ca-Mn-Si
Ca-Mn-Sm	Ca-Mn-Sn	Ca-Mn-Sr	Ca-Mn-Ta	Ca-Mn-Tb	Ca-Mn-Ti	Ca-Mn-V	Ca-Mn-W
Ca-Mn-Y	Ca-Mn-Yb	Ca-Mn-Zn	Ca-Mn-Zr	Ca-Mo-Nb	Ca-Mo-Nd	Ca-Mo-Ni	Ca-Mo-P
Ca-Mo-Pb	Ca-Mo-Pd	Ca-Mo-Pr	Ca-Mo-Pt	Ca-Mo-Ru	Ca-Mo-Sb	Ca-Mo-Sc	Ca-Mo-Si
Ca-Mo-Sm	Ca-Mo-Sn	Ca-Mo-Sr	Ca-Mo-Ta	Ca-Mo-Tb	Ca-Mo-Ti	Ca-Mo-V	Ca-Mo-W
Ca-Mo-Y	Ca-Mo-Yb	Ca-Mo-Zn	Ca-Mo-Zr	Ca-Nb-Nd	Ca-Nb-Ni	Ca-Nb-P	Ca-Nb-Pb
Ca-Nb-Pd	Ca-Nb-Pr	Ca-Nb-Pt	Ca-Nb-Ru	Ca-Nb-Sb	Ca-Nb-Sc	Ca-Nb-Si	Ca-Nb-Sm
Ca-Nb-Sn	Ca-Nb-Sr	Ca-Nb-Ta	Ca-Nb-Tb	Ca-Nb-Ti	Ca-Nb-V	Ca-Nb-W	Ca-Nb-Y
Ca-Nb-Yb	Ca-Nb-Zn	Ca-Nb-Zr	Ca-Nd-Ni	Ca-Nd-P	Ca-Nd-Pb	Ca-Nd-Pd	Ca-Nd-Pr
Ca-Nd-Pt	Ca-Nd-Ru	Ca-Nd-Sb	Ca-Nd-Sc	Ca-Nd-Si	Ca-Nd-Sm	Ca-Nd-Sn	Ca-Nd-Sr
Ca-Nd-Ta	Ca-Nd-Tb	Ca-Nd-Ti	Ca-Nd-V	Ca-Nd-W	Ca-Nd-Y	Ca-Nd-Yb	Ca-Nd-Zn
Ca-Nd-Zr	Ca-Ni-P	Ca-Ni-Pb	Ca-Ni-Pr	Ca-Ni-Pt	Ca-Ni-Ru	Ca-Ni-Sb	Ca-Ni-Sc
Ca-Ni-Si	Ca-Ni-Sm	Ca-Ni-Sn	Ca-Ni-Sr	Ca-Ni-Ta	Ca-Ni-Tb	Ca-Ni-Ti	Ca-Ni-V
Ca-Ni-W	Ca-Ni-Y	Ca-Ni-Yb	Ca-Ni-Zn	Ca-Ni-Zr	Ca-P-Pb	Ca-P-Pd	Ca-P-Pr
Ca-P-Pt	Ca-P-Ru	Ca-P-Sb	Ca-P-Sc	Ca-P-Si	Ca-P-Sm	Ca-P-Sn	Ca-P-Sr
Ca-P-Ta	Ca-P-Tb	Ca-P-Ti	Ca-P-V	Ca-P-W	Ca-P-Y	Ca-P-Yb	Ca-P-Zn
Ca-P-Zr	Ca-Pb-Pd	Ca-Pb-Pr	Ca-Pb-Pt	Ca-Pb-Ru	Ca-Pb-Sb	Ca-Pb-Sc	Ca-Pb-Si
Ca-Pb-Sm	Ca-Pb-Sn	Ca-Pb-Sr	Ca-Pb-Ta	Ca-Pb-Tb	Ca-Pb-Ti	Ca-Pb-V	Ca-Pb-W
Ca-Pb-Y	Ca-Pb-Yb	Ca-Pb-Zn	Ca-Pb-Zr	Ca-Pd-Pr	Ca-Pd-Pt	Ca-Pd-Ru	Ca-Pd-Sb
Ca-Pd-Sc	Ca-Pd-Si	Ca-Pd-Sm	Ca-Pd-Sn	Ca-Pd-Sr	Ca-Pd-Ta	Ca-Pd-Tb	Ca-Pd-Ti
Ca-Pd-V	Ca-Pd-W	Ca-Pd-Y	Ca-Pd-Yb	Ca-Pd-Zn	Ca-Pd-Zr	Ca-Pr-Pt	Ca-Pr-Ru
Ca-Pr-Sb	Ca-Pr-Sc	Ca-Pr-Si	Ca-Pr-Sm	Ca-Pr-Sn	Ca-Pr-Sr	Ca-Pr-Ta	Ca-Pr-Tb

Ca-Pr-Ti	Ca-Pr-V	Ca-Pr-W	Ca-Pr-Y	Ca-Pr-Yb	Ca-Pr-Zn	Ca-Pr-Zr	Ca-Pt-Ru
Ca-Pt-Sb	Ca-Pt-Sc	Ca-Pt-Si	Ca-Pt-Sm	Ca-Pt-Sn	Ca-Pt-Sr	Ca-Pt-Ta	Ca-Pt-Tb
Ca-Pt-Ti	Ca-Pt-V	Ca-Pt-W	Ca-Pt-Y	Ca-Pt-Yb	Ca-Pt-Zn	Ca-Pt-Zr	Ca-Ru-Sb
Ca-Ru-Sc	Ca-Ru-Si	Ca-Ru-Sm	Ca-Ru-Sn	Ca-Ru-Sr	Ca-Ru-Ta	Ca-Ru-Tb	Ca-Ru-Ti
Ca-Ru-V	Ca-Ru-W	Ca-Ru-Y	Ca-Ru-Yb	Ca-Ru-Zn	Ca-Ru-Zr	Ca-Sb-Sc	Ca-Sb-Si
Ca-Sb-Sm	Ca-Sb-Sn	Ca-Sb-Sr	Ca-Sb-Ta	Ca-Sb-Tb	Ca-Sb-Ti	Ca-Sb-V	Ca-Sb-W
Ca-Sb-Y	Ca-Sb-Yb	Ca-Sb-Zn	Ca-Sb-Zr	Ca-Sc-Si	Ca-Sc-Sm	Ca-Sc-Sn	Ca-Sc-Sr
Ca-Sc-Ta	Ca-Sc-Tb	Ca-Sc-Ti	Ca-Sc-V	Ca-Sc-W	Ca-Sc-Y	Ca-Sc-Yb	Ca-Sc-Zn
Ca-Sc-Zr	Ca-Si-Sm	Ca-Si-Sn	Ca-Si-Sr	Ca-Si-Ta	Ca-Si-Tb	Ca-Si-Ti	Ca-Si-V
Ca-Si-W	Ca-Si-Y	Ca-Si-Yb	Ca-Si-Zn	Ca-Si-Zr	Ca-Sm-Sn	Ca-Sm-Sr	Ca-Sm-Ta
Ca-Sm-Tb	Ca-Sm-Ti	Ca-Sm-V	Ca-Sm-W	Ca-Sm-Y	Ca-Sm-Yb	Ca-Sm-Zn	Ca-Sm-Zr
Ca-Sn-Sr	Ca-Sn-Ta	Ca-Sn-Tb	Ca-Sn-Ti	Ca-Sn-V	Ca-Sn-W	Ca-Sn-Y	Ca-Sn-Yb
Ca-Sn-Zn	Ca-Sn-Zr	Ca-Sr-Ta	Ca-Sr-Tb	Ca-Sr-Ti	Ca-Sr-V	Ca-Sr-W	Ca-Sr-Y
Ca-Sr-Yb	Ca-Sr-Zn	Ca-Sr-Zr	Ca-Ta-Tb	Ca-Ta-Ti	Ca-Ta-V	Ca-Ta-W	Ca-Ta-Y
Ca-Ta-Yb	Ca-Ta-Zn	Ca-Ta-Zr	Ca-Tb-Ti	Ca-Tb-V	Ca-Tb-W	Ca-Tb-Y	Ca-Tb-Yb
Ca-Tb-Zn	Ca-Tb-Zr	Ca-Ti-V	Ca-Ti-W	Ca-Ti-Y	Ca-Ti-Yb	Ca-Ti-Zn	Ca-Ti-Zr
Ca-V-W	Ca-V-Y	Ca-V-Yb	Ca-V-Zn	Ca-V-Zr	Ca-W-Y	Ca-W-Yb	Ca-W-Zn
Ca-W-Zr	Ca-Y-Yb	Ca-Y-Zn	Ca-Y-Zr	Ca-Yb-Zn	Ca-Yb-Zr	Ca-Zn-Zr	Ce-Co-Cu
Ce-Co-Dy	Ce-Co-Er	Ce-Co-Ga	Ce-Co-Gd	Ce-Co-Ge	Ce-Co-Hf	Ce-Co-La	Ce-Co-Mg
Ce-Co-Mo	Ce-Co-Nd	Ce-Co-P	Ce-Co-Pb	Ce-Co-Pd	Ce-Co-Pr	Ce-Co-Pt	Ce-Co-Ru
Ce-Co-Sb	Ce-Co-Sc	Ce-Co-Si	Ce-Co-Sm	Ce-Co-Sn	Ce-Co-Sr	Ce-Co-Ta	Ce-Co-Tb
Ce-Co-Ti	Ce-Co-W	Ce-Co-Y	Ce-Co-Yb	Ce-Co-Zn	Ce-Co-Zr	Ce-Cr-Cu	Ce-Cr-Dy
Ce-Cr-Er	Ce-Cr-Ga	Ce-Cr-Gd	Ce-Cr-Ge	Ce-Cr-Hf	Ce-Cr-La	Ce-Cr-Mg	Ce-Cr-Mn
Ce-Cr-Mo	Ce-Cr-Nb	Ce-Cr-Nd	Ce-Cr-P	Ce-Cr-Pb	Ce-Cr-Pd	Ce-Cr-Pr	Ce-Cr-Pt
Ce-Cr-Ru	Ce-Cr-Sb	Ce-Cr-Sc	Ce-Cr-Si	Ce-Cr-Sm	Ce-Cr-Sn	Ce-Cr-Sr	Ce-Cr-Ta

Ce-Cr-Tb	Ce-Cr-Ti	Ce-Cr-V	Ce-Cr-W	Ce-Cr-Y	Ce-Cr-Yb	Ce-Cr-Zn	Ce-Cr-Zr
Ce-Cu-Dy	Ce-Cu-Er	Ce-Cu-Ga	Ce-Cu-Gd	Ce-Cu-Ge	Ce-Cu-Hf	Ce-Cu-La	Ce-Cu-Mn
Ce-Cu-Mo	Ce-Cu-Nd	Ce-Cu-P	Ce-Cu-Pb	Ce-Cu-Pd	Ce-Cu-Pr	Ce-Cu-Pt	Ce-Cu-Ru
Ce-Cu-Sb	Ce-Cu-Sc	Ce-Cu-Si	Ce-Cu-Sm	Ce-Cu-Sn	Ce-Cu-Sr	Ce-Cu-Ta	Ce-Cu-Tb
Ce-Cu-Ti	Ce-Cu-W	Ce-Cu-Y	Ce-Cu-Zr	Ce-Dy-Er	Ce-Dy-Fe	Ce-Dy-Ga	Ce-Dy-Gd
Ce-Dy-Ge	Ce-Dy-Hf	Ce-Dy-La	Ce-Dy-Mg	Ce-Dy-Mn	Ce-Dy-Mo	Ce-Dy-Nb	Ce-Dy-Nd
Ce-Dy-Ni	Ce-Dy-P	Ce-Dy-Pb	Ce-Dy-Pd	Ce-Dy-Pr	Ce-Dy-Pt	Ce-Dy-Ru	Ce-Dy-Sb
Ce-Dy-Sc	Ce-Dy-Si	Ce-Dy-Sm	Ce-Dy-Sn	Ce-Dy-Sr	Ce-Dy-Ta	Ce-Dy-Tb	Ce-Dy-Ti
Ce-Dy-V	Ce-Dy-W	Ce-Dy-Y	Ce-Dy-Yb	Ce-Dy-Zn	Ce-Dy-Zr	Ce-Er-Fe	Ce-Er-Ga
Ce-Er-Gd	Ce-Er-Ge	Ce-Er-Hf	Ce-Er-La	Ce-Er-Mg	Ce-Er-Mn	Ce-Er-Mo	Ce-Er-Nb
Ce-Er-Nd	Ce-Er-Ni	Ce-Er-P	Ce-Er-Pb	Ce-Er-Pd	Ce-Er-Pr	Ce-Er-Pt	Ce-Er-Ru
Ce-Er-Sb	Ce-Er-Sc	Ce-Er-Si	Ce-Er-Sm	Ce-Er-Sn	Ce-Er-Sr	Ce-Er-Ta	Ce-Er-Tb
Ce-Er-Ti	Ce-Er-V	Ce-Er-W	Ce-Er-Y	Ce-Er-Yb	Ce-Er-Zn	Ce-Er-Zr	Ce-Fe-Ga
Ce-Fe-Gd	Ce-Fe-Ge	Ce-Fe-Hf	Ce-Fe-La	Ce-Fe-Mg	Ce-Fe-Mo	Ce-Fe-Nd	Ce-Fe-P
Ce-Fe-Pb	Ce-Fe-Pd	Ce-Fe-Pr	Ce-Fe-Pt	Ce-Fe-Ru	Ce-Fe-Sb	Ce-Fe-Sc	Ce-Fe-Si
Ce-Fe-Sm	Ce-Fe-Sn	Ce-Fe-Sr	Ce-Fe-Ta	Ce-Fe-Tb	Ce-Fe-Ti	Ce-Fe-W	Ce-Fe-Y
Ce-Fe-Yb	Ce-Fe-Zn	Ce-Fe-Zr	Ce-Ga-Gd	Ce-Ga-Ge	Ce-Ga-Hf	Ce-Ga-La	Ce-Ga-Mg
Ce-Ga-Mn	Ce-Ga-Mo	Ce-Ga-Nb	Ce-Ga-Nd	Ce-Ga-Ni	Ce-Ga-P	Ce-Ga-Pb	Ce-Ga-Pd
Ce-Ga-Pr	Ce-Ga-Pt	Ce-Ga-Ru	Ce-Ga-Sb	Ce-Ga-Sc	Ce-Ga-Si	Ce-Ga-Sm	Ce-Ga-Sn
Ce-Ga-Sr	Ce-Ga-Ta	Ce-Ga-Tb	Ce-Ga-Ti	Ce-Ga-V	Ce-Ga-W	Ce-Ga-Y	Ce-Ga-Yb
Ce-Ga-Zn	Ce-Ga-Zr	Ce-Gd-Ge	Ce-Gd-Hf	Ce-Gd-La	Ce-Gd-Mg	Ce-Gd-Mn	Ce-Gd-Mo
Ce-Gd-Nb	Ce-Gd-Nd	Ce-Gd-Ni	Ce-Gd-P	Ce-Gd-Pb	Ce-Gd-Pd	Ce-Gd-Pr	Ce-Gd-Pt
Ce-Gd-Ru	Ce-Gd-Sb	Ce-Gd-Sc	Ce-Gd-Si	Ce-Gd-Sm	Ce-Gd-Sn	Ce-Gd-Sr	Ce-Gd-Ta
Ce-Gd-Tb	Ce-Gd-Ti	Ce-Gd-V	Ce-Gd-W	Ce-Gd-Y	Ce-Gd-Yb	Ce-Gd-Zn	Ce-Gd-Zr
Ce-Ge-Hf	Ce-Ge-La	Ce-Ge-Mg	Ce-Ge-Mn	Ce-Ge-Mo	Ce-Ge-Nb	Ce-Ge-Nd	Ce-Ge-Ni

Ce-Ge-P	Ce-Ge-Pb	Ce-Ge-Pd	Ce-Ge-Pr	Ce-Ge-Pt	Ce-Ge-Ru	Ce-Ge-Sb	Ce-Ge-Sc
Ce-Ge-Si	Ce-Ge-Sm	Ce-Ge-Sn	Ce-Ge-Sr	Ce-Ge-Ta	Ce-Ge-Tb	Ce-Ge-Ti	Ce-Ge-V
Ce-Ge-W	Ce-Ge-Y	Ce-Ge-Yb	Ce-Ge-Zn	Ce-Ge-Zr	Ce-Hf-La	Ce-Hf-Mg	Ce-Hf-Mn
Ce-Hf-Mo	Ce-Hf-Nb	Ce-Hf-Nd	Ce-Hf-Ni	Ce-Hf-P	Ce-Hf-Pb	Ce-Hf-Pd	Ce-Hf-Pr
Ce-Hf-Pt	Ce-Hf-Ru	Ce-Hf-Sb	Ce-Hf-Sc	Ce-Hf-Si	Ce-Hf-Sm	Ce-Hf-Sn	Ce-Hf-Sr
Ce-Hf-Ta	Ce-Hf-Tb	Ce-Hf-Ti	Ce-Hf-V	Ce-Hf-W	Ce-Hf-Y	Ce-Hf-Yb	Ce-Hf-Zn
Ce-Hf-Zr	Ce-La-Mg	Ce-La-Mn	Ce-La-Mo	Ce-La-Nb	Ce-La-Nd	Ce-La-Ni	Ce-La-P
Ce-La-Pb	Ce-La-Pd	Ce-La-Pr	Ce-La-Pt	Ce-La-Ru	Ce-La-Sb	Ce-La-Sc	Ce-La-Si
Ce-La-Sm	Ce-La-Sn	Ce-La-Sr	Ce-La-Ta	Ce-La-Tb	Ce-La-Ti	Ce-La-V	Ce-La-W
Ce-La-Y	Ce-La-Yb	Ce-La-Zn	Ce-La-Zr	Ce-Mg-Mo	Ce-Mg-Nb	Ce-Mg-Nd	Ce-Mg-P
Ce-Mg-Pb	Ce-Mg-Pd	Ce-Mg-Pr	Ce-Mg-Pt	Ce-Mg-Ru	Ce-Mg-Sb	Ce-Mg-Sc	Ce-Mg-Si
Ce-Mg-Sm	Ce-Mg-Sn	Ce-Mg-Sr	Ce-Mg-Ta	Ce-Mg-Tb	Ce-Mg-Ti	Ce-Mg-V	Ce-Mg-W
Ce-Mg-Y	Ce-Mg-Zr	Ce-Mn-Mo	Ce-Mn-Nd	Ce-Mn-P	Ce-Mn-Pb	Ce-Mn-Pd	Ce-Mn-Pr
Ce-Mn-Pt	Ce-Mn-Ru	Ce-Mn-Sb	Ce-Mn-Sc	Ce-Mn-Si	Ce-Mn-Sm	Ce-Mn-Sn	Ce-Mn-Sr
Ce-Mn-Ta	Ce-Mn-Tb	Ce-Mn-Ti	Ce-Mn-V	Ce-Mn-W	Ce-Mn-Y	Ce-Mn-Yb	Ce-Mn-Zn
Ce-Mn-Zr	Ce-Mo-Nb	Ce-Mo-Nd	Ce-Mo-Ni	Ce-Mo-P	Ce-Mo-Pb	Ce-Mo-Pd	Ce-Mo-Pr
Ce-Mo-Pt	Ce-Mo-Ru	Ce-Mo-Sb	Ce-Mo-Sc	Ce-Mo-Si	Ce-Mo-Sm	Ce-Mo-Sn	Ce-Mo-Sr
Ce-Mo-Ta	Ce-Mo-Tb	Ce-Mo-Ti	Ce-Mo-V	Ce-Mo-W	Ce-Mo-Y	Ce-Mo-Yb	Ce-Mo-Zn
Ce-Mo-Zr	Ce-Nb-Nd	Ce-Nb-P	Ce-Nb-Pb	Ce-Nb-Pd	Ce-Nb-Pr	Ce-Nb-Pt	Ce-Nb-Ru
Ce-Nb-Sb	Ce-Nb-Sc	Ce-Nb-Si	Ce-Nb-Sm	Ce-Nb-Sn	Ce-Nb-Sr	Ce-Nb-Ta	Ce-Nb-Tb
Ce-Nb-Ti	Ce-Nb-W	Ce-Nb-Y	Ce-Nb-Yb	Ce-Nb-Zn	Ce-Nb-Zr	Ce-Nd-Ni	Ce-Nd-P
Ce-Nd-Pb	Ce-Nd-Pd	Ce-Nd-Pr	Ce-Nd-Pt	Ce-Nd-Ru	Ce-Nd-Sb	Ce-Nd-Sc	Ce-Nd-Si
Ce-Nd-Sm	Ce-Nd-Sn	Ce-Nd-Sr	Ce-Nd-Ta	Ce-Nd-Tb	Ce-Nd-Ti	Ce-Nd-V	Ce-Nd-W
Ce-Nd-Y	Ce-Nd-Yb	Ce-Nd-Zn	Ce-Nd-Zr	Ce-Ni-P	Ce-Ni-Pb	Ce-Ni-Pd	Ce-Ni-Pr
Ce-Ni-Pt	Ce-Ni-Ru	Ce-Ni-Sb	Ce-Ni-Sc	Ce-Ni-Si	Ce-Ni-Sm	Ce-Ni-Sn	Ce-Ni-Sr

Ce-Ni-Ta	Ce-Ni-Tb	Ce-Ni-Ti	Ce-Ni-W	Ce-Ni-Y	Ce-Ni-Yb	Ce-Ni-Zn	Ce-Ni-Zr
Ce-P-Pb	Ce-P-Pd	Ce-P-Pr	Ce-P-Pt	Ce-P-Ru	Ce-P-Sb	Ce-P-Sc	Ce-P-Si
Ce-P-Sm	Ce-P-Sn	Ce-P-Sr	Ce-P-Ta	Ce-P-Tb	Ce-P-Ti	Ce-P-V	Ce-P-W
Ce-P-Y	Ce-P-Yb	Ce-P-Zn	Ce-P-Zr	Ce-Pb-Pd	Ce-Pb-Pr	Ce-Pb-Pt	Ce-Pb-Ru
Ce-Pb-Sb	Ce-Pb-Sc	Ce-Pb-Si	Ce-Pb-Sm	Ce-Pb-Sn	Ce-Pb-Sr	Ce-Pb-Ta	Ce-Pb-Tb
Ce-Pb-Ti	Ce-Pb-V	Ce-Pb-W	Ce-Pb-Y	Ce-Pb-Yb	Ce-Pb-Zn	Ce-Pb-Zr	Ce-Pd-Pr
Ce-Pd-Pt	Ce-Pd-Ru	Ce-Pd-Sb	Ce-Pd-Sc	Ce-Pd-Si	Ce-Pd-Sm	Ce-Pd-Sn	Ce-Pd-Sr
Ce-Pd-Ta	Ce-Pd-Tb	Ce-Pd-Ti	Ce-Pd-V	Ce-Pd-W	Ce-Pd-Y	Ce-Pd-Yb	Ce-Pd-Zn
Ce-Pd-Zr	Ce-Pr-Pt	Ce-Pr-Ru	Ce-Pr-Sb	Ce-Pr-Sc	Ce-Pr-Si	Ce-Pr-Sm	Ce-Pr-Sn
Ce-Pr-Sr	Ce-Pr-Ta	Ce-Pr-Tb	Ce-Pr-Ti	Ce-Pr-V	Ce-Pr-W	Ce-Pr-Y	Ce-Pr-Yb
Ce-Pr-Zn	Ce-Pr-Zr	Ce-Pt-Ru	Ce-Pt-Sb	Ce-Pt-Sc	Ce-Pt-Si	Ce-Pt-Sm	Ce-Pt-Sn
Ce-Pt-Sr	Ce-Pt-Ta	Ce-Pt-Tb	Ce-Pt-Ti	Ce-Pt-V	Ce-Pt-W	Ce-Pt-Y	Ce-Pt-Yb
Ce-Pt-Zn	Ce-Pt-Zr	Ce-Ru-Sb	Ce-Ru-Sc	Ce-Ru-Si	Ce-Ru-Sm	Ce-Ru-Sn	Ce-Ru-Sr
Ce-Ru-Ta	Ce-Ru-Tb	Ce-Ru-Ti	Ce-Ru-V	Ce-Ru-W	Ce-Ru-Y	Ce-Ru-Yb	Ce-Ru-Zn
Ce-Ru-Zr	Ce-Sb-Sc	Ce-Sb-Si	Ce-Sb-Sm	Ce-Sb-Sn	Ce-Sb-Sr	Ce-Sb-Ta	Ce-Sb-Tb
Ce-Sb-Ti	Ce-Sb-V	Ce-Sb-W	Ce-Sb-Y	Ce-Sb-Yb	Ce-Sb-Zn	Ce-Sb-Zr	Ce-Sc-Si
Ce-Sc-Sm	Ce-Sc-Sn	Ce-Sc-Sr	Ce-Sc-Ta	Ce-Sc-Tb	Ce-Sc-Ti	Ce-Sc-V	Ce-Sc-W
Ce-Sc-Y	Ce-Sc-Yb	Ce-Sc-Zn	Ce-Sc-Zr	Ce-Si-Sm	Ce-Si-Sn	Ce-Si-Sr	Ce-Si-Ta
Ce-Si-Tb	Ce-Si-Ti	Ce-Si-V	Ce-Si-W	Ce-Si-Y	Ce-Si-Yb	Ce-Si-Zn	Ce-Si-Zr
Ce-Sm-Sn	Ce-Sm-Sr	Ce-Sm-Ta	Ce-Sm-Tb	Ce-Sm-Ti	Ce-Sm-V	Ce-Sm-W	Ce-Sm-Y
Ce-Sm-Yb	Ce-Sm-Zn	Ce-Sm-Zr	Ce-Sn-Sr	Ce-Sn-Ta	Ce-Sn-Tb	Ce-Sn-Ti	Ce-Sn-V
Ce-Sn-W	Ce-Sn-Y	Ce-Sn-Yb	Ce-Sn-Zn	Ce-Sn-Zr	Ce-Sr-Ta	Ce-Sr-Tb	Ce-Sr-Ti
Ce-Sr-V	Ce-Sr-W	Ce-Sr-Y	Ce-Sr-Yb	Ce-Sr-Zn	Ce-Sr-Zr	Ce-Ta-Tb	Ce-Ta-Ti
Ce-Ta-V	Ce-Ta-W	Ce-Ta-Y	Ce-Ta-Yb	Ce-Ta-Zn	Ce-Ta-Zr	Ce-Tb-Ti	Ce-Tb-V
Ce-Tb-W	Ce-Tb-Y	Ce-Tb-Yb	Ce-Tb-Zn	Ce-Tb-Zr	Ce-Ti-V	Ce-Ti-W	Ce-Ti-Y

Ce-Ti-Yb	Ce-Ti-Zn	Ce-Ti-Zr	Ce-V-W	Ce-V-Y	Ce-V-Yb	Ce-V-Zn	Ce-V-Zr
Ce-W-Y	Ce-W-Yb	Ce-W-Zn	Ce-W-Zr	Ce-Y-Yb	Ce-Y-Zn	Ce-Y-Zr	Ce-Yb-Zn
Ce-Yb-Zr	Ce-Zn-Zr	Co-Cr-Cu	Co-Cr-Dy	Co-Cr-Er	Co-Cr-Ga	Co-Cr-Gd	Co-Cr-Hf
Co-Cr-Mg	Co-Cr-Mn	Co-Cr-Nb	Co-Cr-Nd	Co-Cr-Pb	Co-Cr-Pr	Co-Cr-Pt	Co-Cr-Ru
Co-Cr-Sb	Co-Cr-Sc	Co-Cr-Sm	Co-Cr-Sn	Co-Cr-Sr	Co-Cr-Ta	Co-Cr-Tb	Co-Cr-Ti
Co-Cr-V	Co-Cr-W	Co-Cr-Y	Co-Cr-Yb	Co-Cr-Zn	Co-Cu-Dy	Co-Cu-Er	Co-Cu-Fe
Co-Cu-Ga	Co-Cu-Gd	Co-Cu-Ge	Co-Cu-Hf	Co-Cu-La	Co-Cu-Mg	Co-Cu-Mn	Co-Cu-Mo
Co-Cu-Nb	Co-Cu-Nd	Co-Cu-Ni	Co-Cu-P	Co-Cu-Pb	Co-Cu-Pd	Co-Cu-Pr	Co-Cu-Pt
Co-Cu-Ru	Co-Cu-Sb	Co-Cu-Sc	Co-Cu-Si	Co-Cu-Sm	Co-Cu-Sn	Co-Cu-Sr	Co-Cu-Ta
Co-Cu-Tb	Co-Cu-Ti	Co-Cu-V	Co-Cu-W	Co-Cu-Y	Co-Cu-Yb	Co-Cu-Zn	Co-Cu-Zr
Co-Dy-Er	Co-Dy-Ga	Co-Dy-Gd	Co-Dy-Ge	Co-Dy-Hf	Co-Dy-La	Co-Dy-Mg	Co-Dy-Mn
Co-Dy-Mo	Co-Dy-Nb	Co-Dy-Nd	Co-Dy-Ni	Co-Dy-P	Co-Dy-Pb	Co-Dy-Pd	Co-Dy-Pr
Co-Dy-Pt	Co-Dy-Ru	Co-Dy-Sb	Co-Dy-Sc	Co-Dy-Si	Co-Dy-Sm	Co-Dy-Sn	Co-Dy-Sr
Co-Dy-Ta	Co-Dy-Tb	Co-Dy-Ti	Co-Dy-V	Co-Dy-W	Co-Dy-Y	Co-Dy-Yb	Co-Dy-Zn
Co-Er-Fe	Co-Er-Ga	Co-Er-Gd	Co-Er-Ge	Co-Er-Hf	Co-Er-La	Co-Er-Mg	Co-Er-Mn
Co-Er-Mo	Co-Er-Nb	Co-Er-Nd	Co-Er-P	Co-Er-Pb	Co-Er-Pd	Co-Er-Pr	Co-Er-Pt
Co-Er-Ru	Co-Er-Sb	Co-Er-Sc	Co-Er-Si	Co-Er-Sm	Co-Er-Sn	Co-Er-Sr	Co-Er-Ta
Co-Er-Tb	Co-Er-Ti	Co-Er-V	Co-Er-W	Co-Er-Y	Co-Er-Yb	Co-Er-Zn	Co-Er-Zr
Co-Fe-Ga	Co-Fe-Mg	Co-Fe-Pb	Co-Fe-Pt	Co-Fe-Ru	Co-Fe-Sb	Co-Fe-Sc	Co-Fe-Sn
Co-Fe-Sr	Co-Fe-Yb	Co-Fe-Zn	Co-Ga-Gd	Co-Ga-Ge	Co-Ga-Hf	Co-Ga-La	Co-Ga-Mg
Co-Ga-Mn	Co-Ga-Mo	Co-Ga-Nb	Co-Ga-Nd	Co-Ga-Ni	Co-Ga-P	Co-Ga-Pb	Co-Ga-Pd
Co-Ga-Pr	Co-Ga-Pt	Co-Ga-Ru	Co-Ga-Sb	Co-Ga-Sc	Co-Ga-Si	Co-Ga-Sm	Co-Ga-Sn
Co-Ga-Sr	Co-Ga-Ta	Co-Ga-Tb	Co-Ga-Ti	Co-Ga-V	Co-Ga-W	Co-Ga-Y	Co-Ga-Yb
Co-Ga-Zn	Co-Ga-Zr	Co-Gd-Ge	Co-Gd-Hf	Co-Gd-La	Co-Gd-Mg	Co-Gd-Mn	Co-Gd-Nb
Co-Gd-Nd	Co-Gd-P	Co-Gd-Pb	Co-Gd-Pd	Co-Gd-Pr	Co-Gd-Pt	Co-Gd-Ru	Co-Gd-Sb

Co-Gd-Sc	Co-Gd-Si	Co-Gd-Sm	Co-Gd-Sn	Co-Gd-Sr	Co-Gd-Ta	Co-Gd-Tb	Co-Gd-Ti
Co-Gd-V	Co-Gd-W	Co-Gd-Y	Co-Gd-Yb	Co-Gd-Zn	Co-Ge-Hf	Co-Ge-La	Co-Ge-Mg
Co-Ge-Nd	Co-Ge-P	Co-Ge-Pb	Co-Ge-Pr	Co-Ge-Pt	Co-Ge-Ru	Co-Ge-Sb	Co-Ge-Sc
Co-Ge-Sm	Co-Ge-Sn	Co-Ge-Sr	Co-Ge-Tb	Co-Ge-W	Co-Ge-Y	Co-Ge-Yb	Co-Ge-Zn
Co-Hf-La	Co-Hf-Mg	Co-Hf-Mn	Co-Hf-Mo	Co-Hf-Nd	Co-Hf-Pb	Co-Hf-Pd	Co-Hf-Pr
Co-Hf-Pt	Co-Hf-Ru	Co-Hf-Sb	Co-Hf-Sc	Co-Hf-Sm	Co-Hf-Sn	Co-Hf-Sr	Co-Hf-Tb
Co-Hf-W	Co-Hf-Y	Co-Hf-Yb	Co-Hf-Zn	Co-La-Mg	Co-La-Nd	Co-La-P	Co-La-Pb
Co-La-Pd	Co-La-Pr	Co-La-Pt	Co-La-Ru	Co-La-Sb	Co-La-Sc	Co-La-Si	Co-La-Sm
Co-La-Sn	Co-La-Sr	Co-La-Ta	Co-La-Tb	Co-La-Ti	Co-La-V	Co-La-W	Co-La-Y
Co-La-Yb	Co-La-Zn	Co-Mg-Mn	Co-Mg-Mo	Co-Mg-Nb	Co-Mg-Nd	Co-Mg-Ni	Co-Mg-P
Co-Mg-Pb	Co-Mg-Pd	Co-Mg-Pr	Co-Mg-Pt	Co-Mg-Ru	Co-Mg-Sb	Co-Mg-Sc	Co-Mg-Si
Co-Mg-Sm	Co-Mg-Sn	Co-Mg-Sr	Co-Mg-Ta	Co-Mg-Tb	Co-Mg-Ti	Co-Mg-V	Co-Mg-W
Co-Mg-Y	Co-Mg-Yb	Co-Mg-Zn	Co-Mg-Zr	Co-Mn-Mo	Co-Mn-Nd	Co-Mn-Pb	Co-Mn-Pr
Co-Mn-Pt	Co-Mn-Ru	Co-Mn-Sb	Co-Mn-Sc	Co-Mn-Sm	Co-Mn-Sn	Co-Mn-Sr	Co-Mn-Ta
Co-Mn-Tb	Co-Mn-Ti	Co-Mn-V	Co-Mn-W	Co-Mn-Yb	Co-Mn-Zn	Co-Mo-Nd	Co-Mo-Pb
Co-Mo-Pd	Co-Mo-Pr	Co-Mo-Pt	Co-Mo-Ru	Co-Mo-Sb	Co-Mo-Sc	Co-Mo-Sm	Co-Mo-Sn
Co-Mo-Sr	Co-Mo-Ta	Co-Mo-Tb	Co-Mo-Ti	Co-Mo-V	Co-Mo-W	Co-Mo-Y	Co-Mo-Yb
Co-Mo-Zn	Co-Nb-Nd	Co-Nb-P	Co-Nb-Pb	Co-Nb-Pr	Co-Nb-Pt	Co-Nb-Ru	Co-Nb-Sb
Co-Nb-Sc	Co-Nb-Sm	Co-Nb-Sn	Co-Nb-Sr	Co-Nb-Tb	Co-Nb-Y	Co-Nb-Yb	Co-Nb-Zn
Co-Nd-P	Co-Nd-Pb	Co-Nd-Pd	Co-Nd-Pr	Co-Nd-Pt	Co-Nd-Ru	Co-Nd-Sb	Co-Nd-Sc
Co-Nd-Si	Co-Nd-Sm	Co-Nd-Sn	Co-Nd-Sr	Co-Nd-Ta	Co-Nd-Tb	Co-Nd-Ti	Co-Nd-V
Co-Nd-W	Co-Nd-Y	Co-Nd-Yb	Co-Nd-Zn	Co-Nd-Zr	Co-Ni-Pb	Co-Ni-Pt	Co-Ni-Ru
Co-Ni-Sb	Co-Ni-Sc	Co-Ni-Sn	Co-Ni-Sr	Co-Ni-Tb	Co-Ni-Yb	Co-Ni-Zn	Co-P-Pb
Co-P-Pr	Co-P-Pt	Co-P-Ru	Co-P-Sb	Co-P-Sc	Co-P-Sm	Co-P-Sn	Co-P-Sr
Co-P-Tb	Co-P-Y	Co-P-Yb	Co-P-Zn	Co-Pb-Pd	Co-Pb-Pr	Co-Pb-Pt	Co-Pb-Ru

Co-Pb-Sb	Co-Pb-Sc	Co-Pb-Si	Co-Pb-Sm	Co-Pb-Sn	Co-Pb-Sr	Co-Pb-Ta	Co-Pb-Tb
Co-Pb-Ti	Co-Pb-V	Co-Pb-W	Co-Pb-Y	Co-Pb-Yb	Co-Pb-Zn	Co-Pb-Zr	Co-Pd-Pr
Co-Pd-Pt	Co-Pd-Ru	Co-Pd-Sb	Co-Pd-Sc	Co-Pd-Sm	Co-Pd-Sn	Co-Pd-Sr	Co-Pd-Tb
Co-Pd-V	Co-Pd-W	Co-Pd-Y	Co-Pd-Yb	Co-Pd-Zn	Co-Pr-Pt	Co-Pr-Ru	Co-Pr-Sb
Co-Pr-Sc	Co-Pr-Si	Co-Pr-Sm	Co-Pr-Sn	Co-Pr-Sr	Co-Pr-Ta	Co-Pr-Tb	Co-Pr-Ti
Co-Pr-V	Co-Pr-W	Co-Pr-Y	Co-Pr-Yb	Co-Pr-Zn	Co-Pt-Ru	Co-Pt-Sb	Co-Pt-Sc
Co-Pt-Si	Co-Pt-Sm	Co-Pt-Sn	Co-Pt-Sr	Co-Pt-Ta	Co-Pt-Tb	Co-Pt-Ti	Co-Pt-V
Co-Pt-W	Co-Pt-Y	Co-Pt-Yb	Co-Pt-Zn	Co-Pt-Zr	Co-Ru-Sb	Co-Ru-Sc	Co-Ru-Si
Co-Ru-Sm	Co-Ru-Sn	Co-Ru-Sr	Co-Ru-Ta	Co-Ru-Tb	Co-Ru-Ti	Co-Ru-V	Co-Ru-W
Co-Ru-Y	Co-Ru-Yb	Co-Ru-Zn	Co-Ru-Zr	Co-Sb-Sc	Co-Sb-Si	Co-Sb-Sm	Co-Sb-Sn
Co-Sb-Sr	Co-Sb-Ta	Co-Sb-Tb	Co-Sb-Ti	Co-Sb-V	Co-Sb-W	Co-Sb-Y	Co-Sb-Yb
Co-Sb-Zn	Co-Sb-Zr	Co-Sc-Si	Co-Sc-Sm	Co-Sc-Sn	Co-Sc-Sr	Co-Sc-Ta	Co-Sc-Tb
Co-Sc-Ti	Co-Sc-V	Co-Sc-W	Co-Sc-Y	Co-Sc-Yb	Co-Sc-Zn	Co-Sc-Zr	Co-Si-Sm
Co-Si-Sn	Co-Si-Sr	Co-Si-Y	Co-Si-Yb	Co-Si-Zn	Co-Sm-Sn	Co-Sm-Sr	Co-Sm-Ta
Co-Sm-Tb	Co-Sm-Ti	Co-Sm-V	Co-Sm-W	Co-Sm-Y	Co-Sm-Yb	Co-Sm-Zn	Co-Sm-Zr
Co-Sn-Sr	Co-Sn-Ta	Co-Sn-Tb	Co-Sn-Ti	Co-Sn-V	Co-Sn-W	Co-Sn-Y	Co-Sn-Yb
Co-Sn-Zn	Co-Sn-Zr	Co-Sr-Ta	Co-Sr-Tb	Co-Sr-Ti	Co-Sr-V	Co-Sr-W	Co-Sr-Y
Co-Sr-Yb	Co-Sr-Zn	Co-Sr-Zr	Co-Ta-Tb	Co-Ta-V	Co-Ta-W	Co-Ta-Y	Co-Ta-Yb
Co-Ta-Zn	Co-Tb-Ti	Co-Tb-V	Co-Tb-W	Co-Tb-Y	Co-Tb-Yb	Co-Tb-Zn	Co-Tb-Zr
Co-Ti-W	Co-Ti-Y	Co-Ti-Yb	Co-Ti-Zn	Co-V-W	Co-V-Y	Co-V-Yb	Co-V-Zn
Co-W-Y	Co-W-Yb	Co-W-Zn	Co-Y-Yb	Co-Y-Zn	Co-Y-Zr	Co-Yb-Zn	Co-Yb-Zr
Co-Zn-Zr	Cr-Cu-Dy	Cr-Cu-Er	Cr-Cu-Fe	Cr-Cu-Ga	Cr-Cu-Gd	Cr-Cu-Ge	Cr-Cu-Hf
Cr-Cu-La	Cr-Cu-Mg	Cr-Cu-Mn	Cr-Cu-Mo	Cr-Cu-Nb	Cr-Cu-Nd	Cr-Cu-Ni	Cr-Cu-P
Cr-Cu-Pb	Cr-Cu-Pd	Cr-Cu-Pr	Cr-Cu-Pt	Cr-Cu-Ru	Cr-Cu-Sb	Cr-Cu-Sc	Cr-Cu-Si
Cr-Cu-Sm	Cr-Cu-Sn	Cr-Cu-Sr	Cr-Cu-Ta	Cr-Cu-Tb	Cr-Cu-Ti	Cr-Cu-V	Cr-Cu-W

Cr-Cu-Y	Cr-Cu-Yb	Cr-Cu-Zn	Cr-Cu-Zr	Cr-Dy-Er	Cr-Dy-Fe	Cr-Dy-Ga	Cr-Dy-Gd
Cr-Dy-Ge	Cr-Dy-Hf	Cr-Dy-La	Cr-Dy-Mg	Cr-Dy-Mn	Cr-Dy-Mo	Cr-Dy-Nb	Cr-Dy-Nd
Cr-Dy-Ni	Cr-Dy-P	Cr-Dy-Pb	Cr-Dy-Pd	Cr-Dy-Pr	Cr-Dy-Pt	Cr-Dy-Ru	Cr-Dy-Sb
Cr-Dy-Sc	Cr-Dy-Si	Cr-Dy-Sm	Cr-Dy-Sn	Cr-Dy-Sr	Cr-Dy-Ta	Cr-Dy-Tb	Cr-Dy-Ti
Cr-Dy-V	Cr-Dy-W	Cr-Dy-Y	Cr-Dy-Yb	Cr-Dy-Zn	Cr-Dy-Zr	Cr-Er-Fe	Cr-Er-Ga
Cr-Er-Gd	Cr-Er-Ge	Cr-Er-Hf	Cr-Er-La	Cr-Er-Mg	Cr-Er-Mn	Cr-Er-Mo	Cr-Er-Nb
Cr-Er-Nd	Cr-Er-Ni	Cr-Er-P	Cr-Er-Pb	Cr-Er-Pd	Cr-Er-Pr	Cr-Er-Pt	Cr-Er-Ru
Cr-Er-Sb	Cr-Er-Sc	Cr-Er-Si	Cr-Er-Sm	Cr-Er-Sn	Cr-Er-Sr	Cr-Er-Ta	Cr-Er-Tb
Cr-Er-Ti	Cr-Er-V	Cr-Er-W	Cr-Er-Y	Cr-Er-Yb	Cr-Er-Zn	Cr-Er-Zr	Cr-Fe-Ga
Cr-Fe-Gd	Cr-Fe-Hf	Cr-Fe-Mg	Cr-Fe-Mn	Cr-Fe-Nb	Cr-Fe-Nd	Cr-Fe-Pb	Cr-Fe-Pr
Cr-Fe-Pt	Cr-Fe-Ru	Cr-Fe-Sb	Cr-Fe-Sc	Cr-Fe-Sm	Cr-Fe-Sn	Cr-Fe-Sr	Cr-Fe-Ta
Cr-Fe-Tb	Cr-Fe-Ti	Cr-Fe-V	Cr-Fe-W	Cr-Fe-Y	Cr-Fe-Yb	Cr-Fe-Zn	Cr-Ga-Gd
Cr-Ga-Ge	Cr-Ga-Hf	Cr-Ga-La	Cr-Ga-Mg	Cr-Ga-Mn	Cr-Ga-Mo	Cr-Ga-Nb	Cr-Ga-Nd
Cr-Ga-Ni	Cr-Ga-P	Cr-Ga-Pb	Cr-Ga-Pd	Cr-Ga-Pr	Cr-Ga-Pt	Cr-Ga-Ru	Cr-Ga-Sb
Cr-Ga-Sc	Cr-Ga-Si	Cr-Ga-Sm	Cr-Ga-Sn	Cr-Ga-Sr	Cr-Ga-Ta	Cr-Ga-Tb	Cr-Ga-Ti
Cr-Ga-V	Cr-Ga-W	Cr-Ga-Y	Cr-Ga-Yb	Cr-Ga-Zn	Cr-Ga-Zr	Cr-Gd-Ge	Cr-Gd-Hf
Cr-Gd-La	Cr-Gd-Mg	Cr-Gd-Mn	Cr-Gd-Mo	Cr-Gd-Nb	Cr-Gd-Nd	Cr-Gd-Ni	Cr-Gd-P
Cr-Gd-Pb	Cr-Gd-Pd	Cr-Gd-Pr	Cr-Gd-Pt	Cr-Gd-Ru	Cr-Gd-Sb	Cr-Gd-Sc	Cr-Gd-Si
Cr-Gd-Sm	Cr-Gd-Sn	Cr-Gd-Sr	Cr-Gd-Ta	Cr-Gd-Tb	Cr-Gd-Ti	Cr-Gd-V	Cr-Gd-W
Cr-Gd-Y	Cr-Gd-Yb	Cr-Gd-Zn	Cr-Gd-Zr	Cr-Ge-Hf	Cr-Ge-La	Cr-Ge-Mg	Cr-Ge-Mn
Cr-Ge-Nb	Cr-Ge-Nd	Cr-Ge-P	Cr-Ge-Pb	Cr-Ge-Pr	Cr-Ge-Pt	Cr-Ge-Ru	Cr-Ge-Sb
Cr-Ge-Sc	Cr-Ge-Sm	Cr-Ge-Sn	Cr-Ge-Sr	Cr-Ge-Ta	Cr-Ge-Tb	Cr-Ge-Ti	Cr-Ge-V
Cr-Ge-W	Cr-Ge-Y	Cr-Ge-Yb	Cr-Ge-Zn	Cr-Hf-La	Cr-Hf-Mg	Cr-Hf-Mn	Cr-Hf-Mo
Cr-Hf-Nb	Cr-Hf-Nd	Cr-Hf-Ni	Cr-Hf-P	Cr-Hf-Pb	Cr-Hf-Pd	Cr-Hf-Pr	Cr-Hf-Pt
Cr-Hf-Ru	Cr-Hf-Sb	Cr-Hf-Sc	Cr-Hf-Si	Cr-Hf-Sm	Cr-Hf-Sn	Cr-Hf-Sr	Cr-Hf-Ta

Cr-Hf-Tb	Cr-Hf-Ti	Cr-Hf-V	Cr-Hf-W	Cr-Hf-Y	Cr-Hf-Yb	Cr-Hf-Zn	Cr-Hf-Zr
Cr-La-Mg	Cr-La-Mn	Cr-La-Nb	Cr-La-Nd	Cr-La-P	Cr-La-Pb	Cr-La-Pd	Cr-La-Pr
Cr-La-Pt	Cr-La-Ru	Cr-La-Sb	Cr-La-Sc	Cr-La-Si	Cr-La-Sm	Cr-La-Sn	Cr-La-Sr
Cr-La-Ta	Cr-La-Tb	Cr-La-Ti	Cr-La-V	Cr-La-W	Cr-La-Y	Cr-La-Yb	Cr-La-Zn
Cr-Mg-Mn	Cr-Mg-Mo	Cr-Mg-Nb	Cr-Mg-Nd	Cr-Mg-Ni	Cr-Mg-P	Cr-Mg-Pb	Cr-Mg-Pd
Cr-Mg-Pr	Cr-Mg-Pt	Cr-Mg-Ru	Cr-Mg-Sb	Cr-Mg-Sc	Cr-Mg-Si	Cr-Mg-Sm	Cr-Mg-Sn
Cr-Mg-Sr	Cr-Mg-Ta	Cr-Mg-Tb	Cr-Mg-Ti	Cr-Mg-V	Cr-Mg-W	Cr-Mg-Y	Cr-Mg-Yb
Cr-Mg-Zn	Cr-Mg-Zr	Cr-Mn-Mo	Cr-Mn-Nb	Cr-Mn-Nd	Cr-Mn-Ni	Cr-Mn-P	Cr-Mn-Pb
Cr-Mn-Pd	Cr-Mn-Pr	Cr-Mn-Pt	Cr-Mn-Ru	Cr-Mn-Sb	Cr-Mn-Sc	Cr-Mn-Si	Cr-Mn-Sm
Cr-Mn-Sn	Cr-Mn-Sr	Cr-Mn-Ta	Cr-Mn-Tb	Cr-Mn-Ti	Cr-Mn-V	Cr-Mn-W	Cr-Mn-Y
Cr-Mn-Yb	Cr-Mn-Zn	Cr-Mn-Zr	Cr-Mo-Nb	Cr-Mo-Nd	Cr-Mo-Pb	Cr-Mo-Pd	Cr-Mo-Pr
Cr-Mo-Pt	Cr-Mo-Ru	Cr-Mo-Sb	Cr-Mo-Sc	Cr-Mo-Sm	Cr-Mo-Sn	Cr-Mo-Sr	Cr-Mo-Ta
Cr-Mo-Tb	Cr-Mo-Ti	Cr-Mo-V	Cr-Mo-W	Cr-Mo-Y	Cr-Mo-Yb	Cr-Mo-Zn	Cr-Nb-Nd
Cr-Nb-Ni	Cr-Nb-P	Cr-Nb-Pb	Cr-Nb-Pd	Cr-Nb-Pr	Cr-Nb-Pt	Cr-Nb-Ru	Cr-Nb-Sb
Cr-Nb-Sc	Cr-Nb-Si	Cr-Nb-Sm	Cr-Nb-Sn	Cr-Nb-Sr	Cr-Nb-Ta	Cr-Nb-Tb	Cr-Nb-Ti
Cr-Nb-V	Cr-Nb-W	Cr-Nb-Y	Cr-Nb-Yb	Cr-Nb-Zn	Cr-Nb-Zr	Cr-Nd-Ni	Cr-Nd-P
Cr-Nd-Pb	Cr-Nd-Pd	Cr-Nd-Pr	Cr-Nd-Pt	Cr-Nd-Ru	Cr-Nd-Sb	Cr-Nd-Sc	Cr-Nd-Si
Cr-Nd-Sm	Cr-Nd-Sn	Cr-Nd-Sr	Cr-Nd-Ta	Cr-Nd-Tb	Cr-Nd-Ti	Cr-Nd-V	Cr-Nd-W
Cr-Nd-Y	Cr-Nd-Yb	Cr-Nd-Zn	Cr-Nd-Zr	Cr-Ni-Pb	Cr-Ni-Pr	Cr-Ni-Pt	Cr-Ni-Ru
Cr-Ni-Sb	Cr-Ni-Sc	Cr-Ni-Sm	Cr-Ni-Sn	Cr-Ni-Sr	Cr-Ni-Ta	Cr-Ni-Tb	Cr-Ni-Ti
Cr-Ni-V	Cr-Ni-W	Cr-Ni-Y	Cr-Ni-Yb	Cr-Ni-Zn	Cr-P-Pb	Cr-P-Pr	Cr-P-Pt
Cr-P-Ru	Cr-P-Sb	Cr-P-Sc	Cr-P-Sm	Cr-P-Sn	Cr-P-Sr	Cr-P-Ta	Cr-P-Tb
Cr-P-Ti	Cr-P-V	Cr-P-W	Cr-P-Y	Cr-P-Yb	Cr-P-Zn	Cr-Pb-Pd	Cr-Pb-Pr
Cr-Pb-Pt	Cr-Pb-Ru	Cr-Pb-Sb	Cr-Pb-Sc	Cr-Pb-Si	Cr-Pb-Sm	Cr-Pb-Sn	Cr-Pb-Sr
Cr-Pb-Ta	Cr-Pb-Tb	Cr-Pb-Ti	Cr-Pb-V	Cr-Pb-W	Cr-Pb-Y	Cr-Pb-Yb	Cr-Pb-Zn

Cr-Pb-Zr	Cr-Pd-Pr	Cr-Pd-Pt	Cr-Pd-Ru	Cr-Pd-Sb	Cr-Pd-Sc	Cr-Pd-Sm	Cr-Pd-Sn
Cr-Pd-Sr	Cr-Pd-Ta	Cr-Pd-Tb	Cr-Pd-Ti	Cr-Pd-V	Cr-Pd-W	Cr-Pd-Y	Cr-Pd-Yb
Cr-Pd-Zn	Cr-Pr-Pt	Cr-Pr-Ru	Cr-Pr-Sb	Cr-Pr-Sc	Cr-Pr-Si	Cr-Pr-Sm	Cr-Pr-Sn
Cr-Pr-Sr	Cr-Pr-Ta	Cr-Pr-Tb	Cr-Pr-Ti	Cr-Pr-V	Cr-Pr-W	Cr-Pr-Y	Cr-Pr-Yb
Cr-Pr-Zn	Cr-Pr-Zr	Cr-Pt-Ru	Cr-Pt-Sb	Cr-Pt-Sc	Cr-Pt-Si	Cr-Pt-Sm	Cr-Pt-Sn
Cr-Pt-Sr	Cr-Pt-Ta	Cr-Pt-Tb	Cr-Pt-Ti	Cr-Pt-V	Cr-Pt-W	Cr-Pt-Y	Cr-Pt-Yb
Cr-Pt-Zn	Cr-Pt-Zr	Cr-Ru-Sb	Cr-Ru-Sc	Cr-Ru-Si	Cr-Ru-Sm	Cr-Ru-Sn	Cr-Ru-Sr
Cr-Ru-Ta	Cr-Ru-Tb	Cr-Ru-Ti	Cr-Ru-V	Cr-Ru-W	Cr-Ru-Y	Cr-Ru-Yb	Cr-Ru-Zn
Cr-Ru-Zr	Cr-Sb-Sc	Cr-Sb-Si	Cr-Sb-Sm	Cr-Sb-Sn	Cr-Sb-Sr	Cr-Sb-Ta	Cr-Sb-Tb
Cr-Sb-Ti	Cr-Sb-V	Cr-Sb-W	Cr-Sb-Y	Cr-Sb-Yb	Cr-Sb-Zn	Cr-Sb-Zr	Cr-Sc-Si
Cr-Sc-Sm	Cr-Sc-Sn	Cr-Sc-Sr	Cr-Sc-Ta	Cr-Sc-Tb	Cr-Sc-Ti	Cr-Sc-V	Cr-Sc-W
Cr-Sc-Y	Cr-Sc-Yb	Cr-Sc-Zn	Cr-Sc-Zr	Cr-Si-Sm	Cr-Si-Sn	Cr-Si-Sr	Cr-Si-Ta
Cr-Si-Tb	Cr-Si-Ti	Cr-Si-V	Cr-Si-W	Cr-Si-Y	Cr-Si-Yb	Cr-Si-Zn	Cr-Sm-Sn
Cr-Sm-Sr	Cr-Sm-Ta	Cr-Sm-Tb	Cr-Sm-Ti	Cr-Sm-V	Cr-Sm-W	Cr-Sm-Y	Cr-Sm-Yb
Cr-Sm-Zn	Cr-Sm-Zr	Cr-Sn-Sr	Cr-Sn-Ta	Cr-Sn-Tb	Cr-Sn-Ti	Cr-Sn-V	Cr-Sn-W
Cr-Sn-Y	Cr-Sn-Yb	Cr-Sn-Zn	Cr-Sn-Zr	Cr-Sr-Ta	Cr-Sr-Tb	Cr-Sr-Ti	Cr-Sr-V
Cr-Sr-W	Cr-Sr-Y	Cr-Sr-Yb	Cr-Sr-Zn	Cr-Sr-Zr	Cr-Ta-Tb	Cr-Ta-Ti	Cr-Ta-V
Cr-Ta-W	Cr-Ta-Y	Cr-Ta-Yb	Cr-Ta-Zn	Cr-Ta-Zr	Cr-Tb-Ti	Cr-Tb-V	Cr-Tb-W
Cr-Tb-Y	Cr-Tb-Yb	Cr-Tb-Zn	Cr-Tb-Zr	Cr-Ti-V	Cr-Ti-W	Cr-Ti-Y	Cr-Ti-Yb
Cr-Ti-Zn	Cr-Ti-Zr	Cr-V-W	Cr-V-Y	Cr-V-Yb	Cr-V-Zn	Cr-V-Zr	Cr-W-Y
Cr-W-Yb	Cr-W-Zn	Cr-W-Zr	Cr-Y-Yb	Cr-Y-Zn	Cr-Y-Zr	Cr-Yb-Zn	Cr-Yb-Zr
Cr-Zn-Zr	Cu-Dy-Er	Cu-Dy-Ga	Cu-Dy-Gd	Cu-Dy-Ge	Cu-Dy-Hf	Cu-Dy-La	Cu-Dy-Mn
Cu-Dy-Mo	Cu-Dy-Nb	Cu-Dy-Nd	Cu-Dy-Ni	Cu-Dy-P	Cu-Dy-Pb	Cu-Dy-Pd	Cu-Dy-Pr
Cu-Dy-Pt	Cu-Dy-Ru	Cu-Dy-Sb	Cu-Dy-Sc	Cu-Dy-Si	Cu-Dy-Sm	Cu-Dy-Sn	Cu-Dy-Sr
Cu-Dy-Ta	Cu-Dy-Tb	Cu-Dy-Ti	Cu-Dy-V	Cu-Dy-W	Cu-Dy-Y	Cu-Dy-Yb	Cu-Dy-Zn

Cu-Er-Fe	Cu-Er-Ga	Cu-Er-Gd	Cu-Er-Ge	Cu-Er-Hf	Cu-Er-La	Cu-Er-Mn	Cu-Er-Mo
Cu-Er-Nb	Cu-Er-Nd	Cu-Er-P	Cu-Er-Pb	Cu-Er-Pd	Cu-Er-Pr	Cu-Er-Pt	Cu-Er-Ru
Cu-Er-Sb	Cu-Er-Sc	Cu-Er-Si	Cu-Er-Sm	Cu-Er-Sn	Cu-Er-Sr	Cu-Er-Ta	Cu-Er-Tb
Cu-Er-Ti	Cu-Er-V	Cu-Er-W	Cu-Er-Y	Cu-Er-Yb	Cu-Er-Zn	Cu-Er-Zr	Cu-Fe-Mg
Cu-Fe-Mn	Cu-Fe-Mo	Cu-Fe-Pb	Cu-Fe-Pt	Cu-Fe-Ru	Cu-Fe-Sb	Cu-Fe-Sc	Cu-Fe-Sm
Cu-Fe-Sr	Cu-Fe-Ta	Cu-Fe-W	Cu-Fe-Yb	Cu-Fe-Zn	Cu-Ga-Ge	Cu-Ga-Hf	Cu-Ga-La
Cu-Ga-Mn	Cu-Ga-Mo	Cu-Ga-Nb	Cu-Ga-Nd	Cu-Ga-Ni	Cu-Ga-P	Cu-Ga-Pb	Cu-Ga-Pd
Cu-Ga-Pr	Cu-Ga-Pt	Cu-Ga-Ru	Cu-Ga-Sb	Cu-Ga-Sc	Cu-Ga-Si	Cu-Ga-Sm	Cu-Ga-Sn
Cu-Ga-Sr	Cu-Ga-Ta	Cu-Ga-Tb	Cu-Ga-Ti	Cu-Ga-V	Cu-Ga-W	Cu-Ga-Y	Cu-Ga-Yb
Cu-Ga-Zn	Cu-Gd-Ge	Cu-Gd-Hf	Cu-Gd-La	Cu-Gd-Mn	Cu-Gd-Mo	Cu-Gd-Nb	Cu-Gd-Nd
Cu-Gd-P	Cu-Gd-Pb	Cu-Gd-Pd	Cu-Gd-Pr	Cu-Gd-Pt	Cu-Gd-Ru	Cu-Gd-Sb	Cu-Gd-Sc
Cu-Gd-Si	Cu-Gd-Sm	Cu-Gd-Sn	Cu-Gd-Sr	Cu-Gd-Ta	Cu-Gd-Tb	Cu-Gd-Ti	Cu-Gd-V
Cu-Gd-W	Cu-Gd-Y	Cu-Gd-Yb	Cu-Gd-Zn	Cu-Ge-Hf	Cu-Ge-La	Cu-Ge-Mg	Cu-Ge-Mn
Cu-Ge-Mo	Cu-Ge-Nd	Cu-Ge-P	Cu-Ge-Pb	Cu-Ge-Pr	Cu-Ge-Pt	Cu-Ge-Ru	Cu-Ge-Sb
Cu-Ge-Sc	Cu-Ge-Sm	Cu-Ge-Sn	Cu-Ge-Sr	Cu-Ge-Ta	Cu-Ge-Tb	Cu-Ge-W	Cu-Ge-Y
Cu-Ge-Yb	Cu-Ge-Zn	Cu-Hf-La	Cu-Hf-Mg	Cu-Hf-Mn	Cu-Hf-Mo	Cu-Hf-Nd	Cu-Hf-Pb
Cu-Hf-Pd	Cu-Hf-Pr	Cu-Hf-Pt	Cu-Hf-Ru	Cu-Hf-Sb	Cu-Hf-Sc	Cu-Hf-Sm	Cu-Hf-Sn
Cu-Hf-Sr	Cu-Hf-Ta	Cu-Hf-Tb	Cu-Hf-W	Cu-Hf-Y	Cu-Hf-Yb	Cu-Hf-Zn	Cu-La-Mn
Cu-La-Mo	Cu-La-Nd	Cu-La-P	Cu-La-Pb	Cu-La-Pd	Cu-La-Pr	Cu-La-Pt	Cu-La-Ru
Cu-La-Sb	Cu-La-Sc	Cu-La-Si	Cu-La-Sm	Cu-La-Sn	Cu-La-Ta	Cu-La-Tb	Cu-La-Ti
Cu-La-V	Cu-La-W	Cu-La-Y	Cu-La-Yb	Cu-Mg-Mn	Cu-Mg-Mo	Cu-Mg-Nb	Cu-Mg-P
Cu-Mg-Pb	Cu-Mg-Pt	Cu-Mg-Ru	Cu-Mg-Sb	Cu-Mg-Sc	Cu-Mg-Si	Cu-Mg-Sm	Cu-Mg-Ta
Cu-Mg-V	Cu-Mg-W	Cu-Mg-Zr	Cu-Mn-Mo	Cu-Mn-Nb	Cu-Mn-Nd	Cu-Mn-Ni	Cu-Mn-P
Cu-Mn-Pb	Cu-Mn-Pd	Cu-Mn-Pr	Cu-Mn-Pt	Cu-Mn-Ru	Cu-Mn-Sb	Cu-Mn-Sc	Cu-Mn-Si
Cu-Mn-Sm	Cu-Mn-Sn	Cu-Mn-Sr	Cu-Mn-Ta	Cu-Mn-Tb	Cu-Mn-Ti	Cu-Mn-V	Cu-Mn-W

Cu-Mn-Y	Cu-Mn-Yb	Cu-Mn-Zn	Cu-Mn-Zr	Cu-Mo-Nb	Cu-Mo-Nd	Cu-Mo-Ni	Cu-Mo-P
Cu-Mo-Pb	Cu-Mo-Pd	Cu-Mo-Pr	Cu-Mo-Pt	Cu-Mo-Ru	Cu-Mo-Sb	Cu-Mo-Sc	Cu-Mo-Si
Cu-Mo-Sm	Cu-Mo-Sn	Cu-Mo-Sr	Cu-Mo-Ta	Cu-Mo-Tb	Cu-Mo-Ti	Cu-Mo-V	Cu-Mo-W
Cu-Mo-Y	Cu-Mo-Yb	Cu-Mo-Zn	Cu-Mo-Zr	Cu-Nb-Nd	Cu-Nb-P	Cu-Nb-Pb	Cu-Nb-Pr
Cu-Nb-Pt	Cu-Nb-Ru	Cu-Nb-Sb	Cu-Nb-Sc	Cu-Nb-Sm	Cu-Nb-Sr	Cu-Nb-Ta	Cu-Nb-Tb
Cu-Nb-W	Cu-Nb-Y	Cu-Nb-Yb	Cu-Nb-Zn	Cu-Nd-P	Cu-Nd-Pb	Cu-Nd-Pd	Cu-Nd-Pr
Cu-Nd-Pt	Cu-Nd-Ru	Cu-Nd-Sb	Cu-Nd-Sc	Cu-Nd-Si	Cu-Nd-Sm	Cu-Nd-Sn	Cu-Nd-Sr
Cu-Nd-Ta	Cu-Nd-Tb	Cu-Nd-Ti	Cu-Nd-V	Cu-Nd-W	Cu-Nd-Y	Cu-Nd-Yb	Cu-Nd-Zn
Cu-Nd-Zr	Cu-Ni-Pb	Cu-Ni-Pt	Cu-Ni-Ru	Cu-Ni-Sb	Cu-Ni-Sc	Cu-Ni-Sm	Cu-Ni-Sr
Cu-Ni-Ta	Cu-Ni-Tb	Cu-Ni-W	Cu-Ni-Yb	Cu-Ni-Zn	Cu-P-Pb	Cu-P-Pr	Cu-P-Pt
Cu-P-Ru	Cu-P-Sb	Cu-P-Sc	Cu-P-Sm	Cu-P-Sn	Cu-P-Sr	Cu-P-Ta	Cu-P-Tb
Cu-P-W	Cu-P-Y	Cu-P-Yb	Cu-P-Zn	Cu-Pb-Pd	Cu-Pb-Pr	Cu-Pb-Pt	Cu-Pb-Ru
Cu-Pb-Sb	Cu-Pb-Sc	Cu-Pb-Si	Cu-Pb-Sm	Cu-Pb-Sn	Cu-Pb-Sr	Cu-Pb-Ta	Cu-Pb-Tb
Cu-Pb-Ti	Cu-Pb-V	Cu-Pb-W	Cu-Pb-Y	Cu-Pb-Yb	Cu-Pb-Zr	Cu-Pd-Pr	Cu-Pd-Pt
Cu-Pd-Ru	Cu-Pd-Sb	Cu-Pd-Sc	Cu-Pd-Sm	Cu-Pd-Sn	Cu-Pd-Sr	Cu-Pd-Ta	Cu-Pd-Tb
Cu-Pd-V	Cu-Pd-W	Cu-Pd-Y	Cu-Pd-Zn	Cu-Pr-Pt	Cu-Pr-Ru	Cu-Pr-Sb	Cu-Pr-Sc
Cu-Pr-Si	Cu-Pr-Sm	Cu-Pr-Sn	Cu-Pr-Sr	Cu-Pr-Ta	Cu-Pr-Tb	Cu-Pr-Ti	Cu-Pr-V
Cu-Pr-W	Cu-Pr-Y	Cu-Pr-Yb	Cu-Pr-Zn	Cu-Pt-Ru	Cu-Pt-Sb	Cu-Pt-Sc	Cu-Pt-Si
Cu-Pt-Sm	Cu-Pt-Sn	Cu-Pt-Sr	Cu-Pt-Ta	Cu-Pt-Tb	Cu-Pt-Ti	Cu-Pt-V	Cu-Pt-W
Cu-Pt-Y	Cu-Pt-Yb	Cu-Pt-Zn	Cu-Pt-Zr	Cu-Ru-Sb	Cu-Ru-Sc	Cu-Ru-Si	Cu-Ru-Sm
Cu-Ru-Sn	Cu-Ru-Sr	Cu-Ru-Ta	Cu-Ru-Tb	Cu-Ru-Ti	Cu-Ru-V	Cu-Ru-W	Cu-Ru-Y
Cu-Ru-Yb	Cu-Ru-Zn	Cu-Ru-Zr	Cu-Sb-Sc	Cu-Sb-Si	Cu-Sb-Sm	Cu-Sb-Sn	Cu-Sb-Sr
Cu-Sb-Ta	Cu-Sb-Tb	Cu-Sb-Ti	Cu-Sb-V	Cu-Sb-W	Cu-Sb-Y	Cu-Sb-Yb	Cu-Sb-Zn
Cu-Sb-Zr	Cu-Sc-Si	Cu-Sc-Sm	Cu-Sc-Sn	Cu-Sc-Sr	Cu-Sc-Ta	Cu-Sc-Tb	Cu-Sc-Ti
Cu-Sc-V	Cu-Sc-W	Cu-Sc-Y	Cu-Sc-Yb	Cu-Sc-Zn	Cu-Sc-Zr	Cu-Si-Sm	Cu-Si-Sr

Cu-Si-Ta	Cu-Si-W	Cu-Si-Y	Cu-Si-Yb	Cu-Si-Zn	Cu-Sm-Sn	Cu-Sm-Sr	Cu-Sm-Ta
Cu-Sm-Tb	Cu-Sm-Ti	Cu-Sm-V	Cu-Sm-W	Cu-Sm-Y	Cu-Sm-Yb	Cu-Sm-Zn	Cu-Sm-Zr
Cu-Sn-Sr	Cu-Sn-Ta	Cu-Sn-Tb	Cu-Sn-Ti	Cu-Sn-V	Cu-Sn-W	Cu-Sn-Y	Cu-Sn-Yb
Cu-Sr-Ta	Cu-Sr-Tb	Cu-Sr-Ti	Cu-Sr-V	Cu-Sr-W	Cu-Sr-Y	Cu-Sr-Yb	Cu-Sr-Zr
Cu-Ta-Tb	Cu-Ta-Ti	Cu-Ta-V	Cu-Ta-W	Cu-Ta-Y	Cu-Ta-Yb	Cu-Ta-Zn	Cu-Ta-Zr
Cu-Tb-Ti	Cu-Tb-V	Cu-Tb-W	Cu-Tb-Y	Cu-Tb-Yb	Cu-Tb-Zn	Cu-Tb-Zr	Cu-Ti-W
Cu-Ti-Y	Cu-Ti-Yb	Cu-Ti-Zn	Cu-V-W	Cu-V-Y	Cu-V-Yb	Cu-V-Zn	Cu-W-Y
Cu-W-Yb	Cu-W-Zn	Cu-W-Zr	Cu-Y-Yb	Cu-Y-Zn	Cu-Y-Zr	Cu-Yb-Zn	Cu-Yb-Zr
Cu-Zn-Zr	Dy-Er-Fe	Dy-Er-Ga	Dy-Er-Gd	Dy-Er-Ge	Dy-Er-Hf	Dy-Er-La	Dy-Er-Mg
Dy-Er-Mn	Dy-Er-Mo	Dy-Er-Nb	Dy-Er-Nd	Dy-Er-Ni	Dy-Er-P	Dy-Er-Pb	Dy-Er-Pd
Dy-Er-Pr	Dy-Er-Pt	Dy-Er-Ru	Dy-Er-Sb	Dy-Er-Sc	Dy-Er-Si	Dy-Er-Sm	Dy-Er-Sn
Dy-Er-Sr	Dy-Er-Ta	Dy-Er-Tb	Dy-Er-Ti	Dy-Er-V	Dy-Er-W	Dy-Er-Y	Dy-Er-Yb
Dy-Er-Zn	Dy-Er-Zr	Dy-Fe-Ga	Dy-Fe-Gd	Dy-Fe-Ge	Dy-Fe-Hf	Dy-Fe-La	Dy-Fe-Mg
Dy-Fe-Mn	Dy-Fe-Mo	Dy-Fe-Nb	Dy-Fe-Nd	Dy-Fe-Ni	Dy-Fe-P	Dy-Fe-Pb	Dy-Fe-Pd
Dy-Fe-Pr	Dy-Fe-Pt	Dy-Fe-Ru	Dy-Fe-Sb	Dy-Fe-Sc	Dy-Fe-Si	Dy-Fe-Sm	Dy-Fe-Sn
Dy-Fe-Sr	Dy-Fe-Ta	Dy-Fe-Tb	Dy-Fe-Ti	Dy-Fe-V	Dy-Fe-W	Dy-Fe-Y	Dy-Fe-Yb
Dy-Fe-Zn	Dy-Ga-Gd	Dy-Ga-Ge	Dy-Ga-Hf	Dy-Ga-La	Dy-Ga-Mg	Dy-Ga-Mn	Dy-Ga-Mo
Dy-Ga-Nb	Dy-Ga-Nd	Dy-Ga-Ni	Dy-Ga-P	Dy-Ga-Pb	Dy-Ga-Pd	Dy-Ga-Pr	Dy-Ga-Pt
Dy-Ga-Ru	Dy-Ga-Sb	Dy-Ga-Sc	Dy-Ga-Si	Dy-Ga-Sm	Dy-Ga-Sn	Dy-Ga-Sr	Dy-Ga-Ta
Dy-Ga-Tb	Dy-Ga-Ti	Dy-Ga-V	Dy-Ga-W	Dy-Ga-Y	Dy-Ga-Yb	Dy-Ga-Zn	Dy-Ga-Zr
Dy-Gd-Ge	Dy-Gd-Hf	Dy-Gd-La	Dy-Gd-Mg	Dy-Gd-Mn	Dy-Gd-Mo	Dy-Gd-Nb	Dy-Gd-Nd
Dy-Gd-Ni	Dy-Gd-P	Dy-Gd-Pb	Dy-Gd-Pd	Dy-Gd-Pr	Dy-Gd-Pt	Dy-Gd-Ru	Dy-Gd-Sb
Dy-Gd-Sc	Dy-Gd-Si	Dy-Gd-Sm	Dy-Gd-Sn	Dy-Gd-Sr	Dy-Gd-Ta	Dy-Gd-Tb	Dy-Gd-Ti
Dy-Gd-V	Dy-Gd-W	Dy-Gd-Y	Dy-Gd-Yb	Dy-Gd-Zn	Dy-Gd-Zr	Dy-Ge-Hf	Dy-Ge-La
Dy-Ge-Mg	Dy-Ge-Mn	Dy-Ge-Mo	Dy-Ge-Nb	Dy-Ge-Nd	Dy-Ge-Ni	Dy-Ge-P	Dy-Ge-Pb

Dy-Ge-Pd	Dy-Ge-Pr	Dy-Ge-Pt	Dy-Ge-Ru	Dy-Ge-Sb	Dy-Ge-Sc	Dy-Ge-Si	Dy-Ge-Sm
Dy-Ge-Sn	Dy-Ge-Sr	Dy-Ge-Ta	Dy-Ge-Tb	Dy-Ge-Ti	Dy-Ge-V	Dy-Ge-W	Dy-Ge-Y
Dy-Ge-Yb	Dy-Ge-Zn	Dy-Ge-Zr	Dy-Hf-La	Dy-Hf-Mg	Dy-Hf-Mn	Dy-Hf-Mo	Dy-Hf-Nb
Dy-Hf-Nd	Dy-Hf-Ni	Dy-Hf-P	Dy-Hf-Pb	Dy-Hf-Pd	Dy-Hf-Pr	Dy-Hf-Pt	Dy-Hf-Ru
Dy-Hf-Sb	Dy-Hf-Sc	Dy-Hf-Si	Dy-Hf-Sm	Dy-Hf-Sn	Dy-Hf-Sr	Dy-Hf-Ta	Dy-Hf-Tb
Dy-Hf-Ti	Dy-Hf-V	Dy-Hf-W	Dy-Hf-Y	Dy-Hf-Yb	Dy-Hf-Zn	Dy-Hf-Zr	Dy-La-Mg
Dy-La-Mn	Dy-La-Mo	Dy-La-Nb	Dy-La-Nd	Dy-La-Ni	Dy-La-P	Dy-La-Pb	Dy-La-Pd
Dy-La-Pr	Dy-La-Pt	Dy-La-Ru	Dy-La-Sb	Dy-La-Sc	Dy-La-Si	Dy-La-Sm	Dy-La-Sn
Dy-La-Sr	Dy-La-Ta	Dy-La-Tb	Dy-La-Ti	Dy-La-V	Dy-La-W	Dy-La-Y	Dy-La-Yb
Dy-La-Zn	Dy-La-Zr	Dy-Mg-Mn	Dy-Mg-Mo	Dy-Mg-Nb	Dy-Mg-Nd	Dy-Mg-Ni	Dy-Mg-P
Dy-Mg-Pb	Dy-Mg-Pd	Dy-Mg-Pr	Dy-Mg-Pt	Dy-Mg-Ru	Dy-Mg-Sb	Dy-Mg-Sc	Dy-Mg-Si
Dy-Mg-Sm	Dy-Mg-Sn	Dy-Mg-Sr	Dy-Mg-Ta	Dy-Mg-Tb	Dy-Mg-Ti	Dy-Mg-V	Dy-Mg-W
Dy-Mg-Y	Dy-Mg-Yb	Dy-Mg-Zn	Dy-Mg-Zr	Dy-Mn-Mo	Dy-Mn-Nb	Dy-Mn-Nd	Dy-Mn-Ni
Dy-Mn-P	Dy-Mn-Pb	Dy-Mn-Pd	Dy-Mn-Pr	Dy-Mn-Pt	Dy-Mn-Ru	Dy-Mn-Sb	Dy-Mn-Sc
Dy-Mn-Si	Dy-Mn-Sm	Dy-Mn-Sn	Dy-Mn-Sr	Dy-Mn-Ta	Dy-Mn-Tb	Dy-Mn-Ti	Dy-Mn-V
Dy-Mn-W	Dy-Mn-Y	Dy-Mn-Yb	Dy-Mn-Zn	Dy-Mn-Zr	Dy-Mo-Nb	Dy-Mo-Nd	Dy-Mo-Ni
Dy-Mo-P	Dy-Mo-Pb	Dy-Mo-Pd	Dy-Mo-Pr	Dy-Mo-Pt	Dy-Mo-Ru	Dy-Mo-Sb	Dy-Mo-Sc
Dy-Mo-Si	Dy-Mo-Sm	Dy-Mo-Sn	Dy-Mo-Sr	Dy-Mo-Ta	Dy-Mo-Tb	Dy-Mo-Ti	Dy-Mo-V
Dy-Mo-W	Dy-Mo-Y	Dy-Mo-Yb	Dy-Mo-Zn	Dy-Mo-Zr	Dy-Nb-Nd	Dy-Nb-Ni	Dy-Nb-P
Dy-Nb-Pb	Dy-Nb-Pd	Dy-Nb-Pr	Dy-Nb-Pt	Dy-Nb-Ru	Dy-Nb-Sb	Dy-Nb-Sc	Dy-Nb-Si
Dy-Nb-Sm	Dy-Nb-Sn	Dy-Nb-Sr	Dy-Nb-Ta	Dy-Nb-Tb	Dy-Nb-Ti	Dy-Nb-V	Dy-Nb-W
Dy-Nb-Y	Dy-Nb-Yb	Dy-Nb-Zn	Dy-Nb-Zr	Dy-Nd-Ni	Dy-Nd-P	Dy-Nd-Pb	Dy-Nd-Pd
Dy-Nd-Pr	Dy-Nd-Pt	Dy-Nd-Ru	Dy-Nd-Sb	Dy-Nd-Sc	Dy-Nd-Si	Dy-Nd-Sm	Dy-Nd-Sn
Dy-Nd-Sr	Dy-Nd-Ta	Dy-Nd-Tb	Dy-Nd-Ti	Dy-Nd-V	Dy-Nd-W	Dy-Nd-Y	Dy-Nd-Yb
Dy-Nd-Zn	Dy-Nd-Zr	Dy-Ni-P	Dy-Ni-Pb	Dy-Ni-Pd	Dy-Ni-Pr	Dy-Ni-Pt	Dy-Ni-Ru

Dy-Ni-Sb	Dy-Ni-Sc	Dy-Ni-Si	Dy-Ni-Sm	Dy-Ni-Sn	Dy-Ni-Sr	Dy-Ni-Ta	Dy-Ni-Tb
Dy-Ni-Ti	Dy-Ni-V	Dy-Ni-W	Dy-Ni-Y	Dy-Ni-Yb	Dy-Ni-Zn	Dy-Ni-Zr	Dy-P-Pb
Dy-P-Pd	Dy-P-Pr	Dy-P-Pt	Dy-P-Ru	Dy-P-Sb	Dy-P-Sc	Dy-P-Si	Dy-P-Sm
Dy-P-Sn	Dy-P-Sr	Dy-P-Ta	Dy-P-Tb	Dy-P-Ti	Dy-P-V	Dy-P-W	Dy-P-Y
Dy-P-Yb	Dy-P-Zn	Dy-P-Zr	Dy-Pb-Pd	Dy-Pb-Pr	Dy-Pb-Pt	Dy-Pb-Ru	Dy-Pb-Sb
Dy-Pb-Sc	Dy-Pb-Si	Dy-Pb-Sm	Dy-Pb-Sn	Dy-Pb-Sr	Dy-Pb-Ta	Dy-Pb-Tb	Dy-Pb-Ti
Dy-Pb-V	Dy-Pb-W	Dy-Pb-Y	Dy-Pb-Yb	Dy-Pb-Zn	Dy-Pb-Zr	Dy-Pd-Pr	Dy-Pd-Pt
Dy-Pd-Ru	Dy-Pd-Sb	Dy-Pd-Sc	Dy-Pd-Si	Dy-Pd-Sm	Dy-Pd-Sn	Dy-Pd-Sr	Dy-Pd-Ta
Dy-Pd-Tb	Dy-Pd-Ti	Dy-Pd-V	Dy-Pd-W	Dy-Pd-Y	Dy-Pd-Yb	Dy-Pd-Zn	Dy-Pd-Zr
Dy-Pr-Pt	Dy-Pr-Ru	Dy-Pr-Sb	Dy-Pr-Sc	Dy-Pr-Si	Dy-Pr-Sm	Dy-Pr-Sn	Dy-Pr-Sr
Dy-Pr-Ta	Dy-Pr-Tb	Dy-Pr-Ti	Dy-Pr-V	Dy-Pr-W	Dy-Pr-Y	Dy-Pr-Yb	Dy-Pr-Zn
Dy-Pr-Zr	Dy-Pt-Ru	Dy-Pt-Sb	Dy-Pt-Sc	Dy-Pt-Si	Dy-Pt-Sm	Dy-Pt-Sn	Dy-Pt-Sr
Dy-Pt-Ta	Dy-Pt-Tb	Dy-Pt-Ti	Dy-Pt-V	Dy-Pt-W	Dy-Pt-Y	Dy-Pt-Yb	Dy-Pt-Zn
Dy-Pt-Zr	Dy-Ru-Sb	Dy-Ru-Sc	Dy-Ru-Si	Dy-Ru-Sm	Dy-Ru-Sn	Dy-Ru-Sr	Dy-Ru-Ta
Dy-Ru-Tb	Dy-Ru-Ti	Dy-Ru-V	Dy-Ru-W	Dy-Ru-Y	Dy-Ru-Yb	Dy-Ru-Zn	Dy-Ru-Zr
Dy-Sb-Sc	Dy-Sb-Si	Dy-Sb-Sm	Dy-Sb-Sn	Dy-Sb-Sr	Dy-Sb-Ta	Dy-Sb-Tb	Dy-Sb-Ti
Dy-Sb-V	Dy-Sb-W	Dy-Sb-Y	Dy-Sb-Yb	Dy-Sb-Zn	Dy-Sb-Zr	Dy-Sc-Si	Dy-Sc-Sm
Dy-Sc-Sn	Dy-Sc-Sr	Dy-Sc-Ta	Dy-Sc-Tb	Dy-Sc-Ti	Dy-Sc-V	Dy-Sc-W	Dy-Sc-Y
Dy-Sc-Yb	Dy-Sc-Zn	Dy-Sc-Zr	Dy-Si-Sm	Dy-Si-Sn	Dy-Si-Sr	Dy-Si-Ta	Dy-Si-Tb
Dy-Si-Ti	Dy-Si-V	Dy-Si-W	Dy-Si-Y	Dy-Si-Yb	Dy-Si-Zn	Dy-Si-Zr	Dy-Sm-Sn
Dy-Sm-Sr	Dy-Sm-Ta	Dy-Sm-Tb	Dy-Sm-Ti	Dy-Sm-V	Dy-Sm-W	Dy-Sm-Y	Dy-Sm-Yb
Dy-Sm-Zn	Dy-Sm-Zr	Dy-Sn-Sr	Dy-Sn-Ta	Dy-Sn-Tb	Dy-Sn-Ti	Dy-Sn-V	Dy-Sn-W
Dy-Sn-Y	Dy-Sn-Yb	Dy-Sn-Zn	Dy-Sn-Zr	Dy-Sr-Ta	Dy-Sr-Tb	Dy-Sr-Ti	Dy-Sr-V
Dy-Sr-W	Dy-Sr-Y	Dy-Sr-Yb	Dy-Sr-Zn	Dy-Sr-Zr	Dy-Ta-Tb	Dy-Ta-Ti	Dy-Ta-V
Dy-Ta-W	Dy-Ta-Y	Dy-Ta-Yb	Dy-Ta-Zn	Dy-Ta-Zr	Dy-Tb-Ti	Dy-Tb-V	Dy-Tb-W

Dy-Tb-Y	Dy-Tb-Yb	Dy-Tb-Zn	Dy-Tb-Zr	Dy-Ti-V	Dy-Ti-W	Dy-Ti-Y	Dy-Ti-Yb
Dy-Ti-Zn	Dy-Ti-Zr	Dy-V-W	Dy-V-Y	Dy-V-Yb	Dy-V-Zn	Dy-V-Zr	Dy-W-Y
Dy-W-Yb	Dy-W-Zn	Dy-W-Zr	Dy-Y-Yb	Dy-Y-Zn	Dy-Y-Zr	Dy-Yb-Zn	Dy-Yb-Zr
Dy-Zn-Zr	Er-Fe-Ga	Er-Fe-Gd	Er-Fe-Ge	Er-Fe-Hf	Er-Fe-La	Er-Fe-Mg	Er-Fe-Mn
Er-Fe-Mo	Er-Fe-Nb	Er-Fe-Nd	Er-Fe-Ni	Er-Fe-P	Er-Fe-Pb	Er-Fe-Pd	Er-Fe-Pr
Er-Fe-Pt	Er-Fe-Ru	Er-Fe-Sb	Er-Fe-Sc	Er-Fe-Si	Er-Fe-Sm	Er-Fe-Sn	Er-Fe-Sr
Er-Fe-Ta	Er-Fe-Tb	Er-Fe-Ti	Er-Fe-V	Er-Fe-W	Er-Fe-Y	Er-Fe-Yb	Er-Fe-Zn
Er-Fe-Zr	Er-Ga-Gd	Er-Ga-Ge	Er-Ga-Hf	Er-Ga-La	Er-Ga-Mg	Er-Ga-Mn	Er-Ga-Mo
Er-Ga-Nb	Er-Ga-Nd	Er-Ga-Ni	Er-Ga-P	Er-Ga-Pb	Er-Ga-Pd	Er-Ga-Pr	Er-Ga-Pt
Er-Ga-Ru	Er-Ga-Sb	Er-Ga-Sc	Er-Ga-Si	Er-Ga-Sm	Er-Ga-Sn	Er-Ga-Sr	Er-Ga-Ta
Er-Ga-Tb	Er-Ga-Ti	Er-Ga-V	Er-Ga-W	Er-Ga-Y	Er-Ga-Yb	Er-Ga-Zn	Er-Ga-Zr
Er-Gd-Ge	Er-Gd-Hf	Er-Gd-La	Er-Gd-Mg	Er-Gd-Mn	Er-Gd-Mo	Er-Gd-Nb	Er-Gd-Nd
Er-Gd-Ni	Er-Gd-P	Er-Gd-Pb	Er-Gd-Pd	Er-Gd-Pr	Er-Gd-Pt	Er-Gd-Ru	Er-Gd-Sb
Er-Gd-Sc	Er-Gd-Si	Er-Gd-Sm	Er-Gd-Sn	Er-Gd-Sr	Er-Gd-Ta	Er-Gd-Tb	Er-Gd-Ti
Er-Gd-V	Er-Gd-W	Er-Gd-Y	Er-Gd-Yb	Er-Gd-Zn	Er-Gd-Zr	Er-Ge-Hf	Er-Ge-La
Er-Ge-Mg	Er-Ge-Mn	Er-Ge-Mo	Er-Ge-Nb	Er-Ge-Nd	Er-Ge-Ni	Er-Ge-P	Er-Ge-Pb
Er-Ge-Pd	Er-Ge-Pr	Er-Ge-Pt	Er-Ge-Ru	Er-Ge-Sb	Er-Ge-Sc	Er-Ge-Si	Er-Ge-Sm
Er-Ge-Sn	Er-Ge-Sr	Er-Ge-Ta	Er-Ge-Tb	Er-Ge-Ti	Er-Ge-V	Er-Ge-W	Er-Ge-Y
Er-Ge-Yb	Er-Ge-Zn	Er-Ge-Zr	Er-Hf-La	Er-Hf-Mg	Er-Hf-Mn	Er-Hf-Mo	Er-Hf-Nb
Er-Hf-Nd	Er-Hf-Ni	Er-Hf-P	Er-Hf-Pb	Er-Hf-Pd	Er-Hf-Pr	Er-Hf-Pt	Er-Hf-Ru
Er-Hf-Sb	Er-Hf-Sc	Er-Hf-Si	Er-Hf-Sm	Er-Hf-Sn	Er-Hf-Sr	Er-Hf-Ta	Er-Hf-Tb
Er-Hf-Ti	Er-Hf-V	Er-Hf-W	Er-Hf-Y	Er-Hf-Yb	Er-Hf-Zn	Er-Hf-Zr	Er-La-Mg
Er-La-Mn	Er-La-Mo	Er-La-Nb	Er-La-Nd	Er-La-Ni	Er-La-P	Er-La-Pb	Er-La-Pd
Er-La-Pr	Er-La-Pt	Er-La-Ru	Er-La-Sb	Er-La-Sc	Er-La-Si	Er-La-Sm	Er-La-Sn
Er-La-Sr	Er-La-Ta	Er-La-Tb	Er-La-Ti	Er-La-V	Er-La-W	Er-La-Y	Er-La-Yb

Er-La-Zn	Er-La-Zr	Er-Mg-Mn	Er-Mg-Mo	Er-Mg-Nb	Er-Mg-Nd	Er-Mg-P	Er-Mg-Pb
Er-Mg-Pd	Er-Mg-Pr	Er-Mg-Pt	Er-Mg-Ru	Er-Mg-Sb	Er-Mg-Sc	Er-Mg-Si	Er-Mg-Sm
Er-Mg-Sn	Er-Mg-Sr	Er-Mg-Ta	Er-Mg-Tb	Er-Mg-Ti	Er-Mg-V	Er-Mg-W	Er-Mg-Y
Er-Mg-Yb	Er-Mg-Zn	Er-Mg-Zr	Er-Mn-Mo	Er-Mn-Nb	Er-Mn-Nd	Er-Mn-Ni	Er-Mn-P
Er-Mn-Pb	Er-Mn-Pd	Er-Mn-Pr	Er-Mn-Pt	Er-Mn-Ru	Er-Mn-Sb	Er-Mn-Sc	Er-Mn-Si
Er-Mn-Sm	Er-Mn-Sn	Er-Mn-Sr	Er-Mn-Ta	Er-Mn-Tb	Er-Mn-Ti	Er-Mn-V	Er-Mn-W
Er-Mn-Y	Er-Mn-Yb	Er-Mn-Zn	Er-Mn-Zr	Er-Mo-Nb	Er-Mo-Nd	Er-Mo-Ni	Er-Mo-P
Er-Mo-Pb	Er-Mo-Pd	Er-Mo-Pr	Er-Mo-Pt	Er-Mo-Ru	Er-Mo-Sb	Er-Mo-Sc	Er-Mo-Si
Er-Mo-Sm	Er-Mo-Sn	Er-Mo-Sr	Er-Mo-Ta	Er-Mo-Tb	Er-Mo-Ti	Er-Mo-V	Er-Mo-W
Er-Mo-Y	Er-Mo-Yb	Er-Mo-Zn	Er-Mo-Zr	Er-Nb-Nd	Er-Nb-Ni	Er-Nb-P	Er-Nb-Pb
Er-Nb-Pd	Er-Nb-Pr	Er-Nb-Pt	Er-Nb-Ru	Er-Nb-Sb	Er-Nb-Sc	Er-Nb-Si	Er-Nb-Sm
Er-Nb-Sn	Er-Nb-Sr	Er-Nb-Ta	Er-Nb-Tb	Er-Nb-Ti	Er-Nb-V	Er-Nb-W	Er-Nb-Y
Er-Nb-Yb	Er-Nb-Zn	Er-Nb-Zr	Er-Nd-Ni	Er-Nd-P	Er-Nd-Pb	Er-Nd-Pd	Er-Nd-Pr
Er-Nd-Pt	Er-Nd-Ru	Er-Nd-Sb	Er-Nd-Sc	Er-Nd-Si	Er-Nd-Sm	Er-Nd-Sn	Er-Nd-Sr
Er-Nd-Ta	Er-Nd-Tb	Er-Nd-Ti	Er-Nd-V	Er-Nd-W	Er-Nd-Y	Er-Nd-Yb	Er-Nd-Zn
Er-Nd-Zr	Er-Ni-P	Er-Ni-Pb	Er-Ni-Pd	Er-Ni-Pr	Er-Ni-Pt	Er-Ni-Ru	Er-Ni-Sb
Er-Ni-Sc	Er-Ni-Si	Er-Ni-Sm	Er-Ni-Sn	Er-Ni-Sr	Er-Ni-Ta	Er-Ni-Tb	Er-Ni-Ti
Er-Ni-V	Er-Ni-W	Er-Ni-Y	Er-Ni-Yb	Er-Ni-Zn	Er-Ni-Zr	Er-P-Pb	Er-P-Pd
Er-P-Pr	Er-P-Pt	Er-P-Ru	Er-P-Sb	Er-P-Sc	Er-P-Si	Er-P-Sm	Er-P-Sn
Er-P-Sr	Er-P-Ta	Er-P-Tb	Er-P-Ti	Er-P-V	Er-P-W	Er-P-Y	Er-P-Yb
Er-P-Zn	Er-P-Zr	Er-Pb-Pd	Er-Pb-Pr	Er-Pb-Pt	Er-Pb-Ru	Er-Pb-Sb	Er-Pb-Sc
Er-Pb-Si	Er-Pb-Sm	Er-Pb-Sn	Er-Pb-Sr	Er-Pb-Ta	Er-Pb-Tb	Er-Pb-Ti	Er-Pb-V
Er-Pb-W	Er-Pb-Y	Er-Pb-Yb	Er-Pb-Zn	Er-Pb-Zr	Er-Pd-Pr	Er-Pd-Pt	Er-Pd-Ru
Er-Pd-Sb	Er-Pd-Sc	Er-Pd-Si	Er-Pd-Sm	Er-Pd-Sn	Er-Pd-Sr	Er-Pd-Ta	Er-Pd-Tb
Er-Pd-Ti	Er-Pd-V	Er-Pd-W	Er-Pd-Y	Er-Pd-Yb	Er-Pd-Zn	Er-Pd-Zr	Er-Pr-Pt

Er-Pr-Ru	Er-Pr-Sb	Er-Pr-Sc	Er-Pr-Si	Er-Pr-Sm	Er-Pr-Sn	Er-Pr-Sr	Er-Pr-Ta
Er-Pr-Tb	Er-Pr-Ti	Er-Pr-V	Er-Pr-W	Er-Pr-Y	Er-Pr-Yb	Er-Pr-Zn	Er-Pr-Zr
Er-Pt-Ru	Er-Pt-Sb	Er-Pt-Sc	Er-Pt-Si	Er-Pt-Sm	Er-Pt-Sn	Er-Pt-Sr	Er-Pt-Ta
Er-Pt-Tb	Er-Pt-Ti	Er-Pt-V	Er-Pt-W	Er-Pt-Y	Er-Pt-Yb	Er-Pt-Zn	Er-Pt-Zr
Er-Ru-Sb	Er-Ru-Sc	Er-Ru-Si	Er-Ru-Sm	Er-Ru-Sn	Er-Ru-Sr	Er-Ru-Ta	Er-Ru-Tb
Er-Ru-Ti	Er-Ru-V	Er-Ru-W	Er-Ru-Y	Er-Ru-Yb	Er-Ru-Zn	Er-Ru-Zr	Er-Sb-Sc
Er-Sb-Si	Er-Sb-Sm	Er-Sb-Sn	Er-Sb-Sr	Er-Sb-Ta	Er-Sb-Tb	Er-Sb-Ti	Er-Sb-V
Er-Sb-W	Er-Sb-Y	Er-Sb-Yb	Er-Sb-Zn	Er-Sb-Zr	Er-Sc-Si	Er-Sc-Sm	Er-Sc-Sn
Er-Sc-Sr	Er-Sc-Ta	Er-Sc-Tb	Er-Sc-Ti	Er-Sc-V	Er-Sc-W	Er-Sc-Y	Er-Sc-Yb
Er-Sc-Zn	Er-Sc-Zr	Er-Si-Sm	Er-Si-Sn	Er-Si-Sr	Er-Si-Ta	Er-Si-Tb	Er-Si-Ti
Er-Si-V	Er-Si-W	Er-Si-Y	Er-Si-Yb	Er-Si-Zn	Er-Si-Zr	Er-Sm-Sn	Er-Sm-Sr
Er-Sm-Ta	Er-Sm-Tb	Er-Sm-Ti	Er-Sm-V	Er-Sm-W	Er-Sm-Y	Er-Sm-Yb	Er-Sm-Zn
Er-Sm-Zr	Er-Sn-Sr	Er-Sn-Ta	Er-Sn-Tb	Er-Sn-Ti	Er-Sn-V	Er-Sn-W	Er-Sn-Y
Er-Sn-Yb	Er-Sn-Zn	Er-Sn-Zr	Er-Sr-Ta	Er-Sr-Tb	Er-Sr-Ti	Er-Sr-V	Er-Sr-W
Er-Sr-Y	Er-Sr-Yb	Er-Sr-Zn	Er-Sr-Zr	Er-Ta-Tb	Er-Ta-Ti	Er-Ta-V	Er-Ta-W
Er-Ta-Y	Er-Ta-Yb	Er-Ta-Zn	Er-Ta-Zr	Er-Tb-Ti	Er-Tb-V	Er-Tb-W	Er-Tb-Y
Er-Tb-Yb	Er-Tb-Zn	Er-Tb-Zr	Er-Ti-V	Er-Ti-W	Er-Ti-Y	Er-Ti-Yb	Er-Ti-Zn
Er-Ti-Zr	Er-V-W	Er-V-Y	Er-V-Yb	Er-V-Zn	Er-V-Zr	Er-W-Y	Er-W-Yb
Er-W-Zn	Er-W-Zr	Er-Y-Yb	Er-Y-Zn	Er-Y-Zr	Er-Yb-Zn	Er-Yb-Zr	Er-Zn-Zr
Fe-Ga-Ge	Fe-Ga-Hf	Fe-Ga-La	Fe-Ga-Mg	Fe-Ga-Mn	Fe-Ga-Mo	Fe-Ga-Nb	Fe-Ga-Nd
Fe-Ga-Ni	Fe-Ga-P	Fe-Ga-Pb	Fe-Ga-Pd	Fe-Ga-Pr	Fe-Ga-Pt	Fe-Ga-Ru	Fe-Ga-Sb
Fe-Ga-Sc	Fe-Ga-Si	Fe-Ga-Sm	Fe-Ga-Sn	Fe-Ga-Sr	Fe-Ga-Ta	Fe-Ga-Tb	Fe-Ga-Ti
Fe-Ga-V	Fe-Ga-W	Fe-Ga-Y	Fe-Ga-Yb	Fe-Ga-Zn	Fe-Gd-Ge	Fe-Gd-Hf	Fe-Gd-La
Fe-Gd-Mg	Fe-Gd-Mn	Fe-Gd-Nb	Fe-Gd-Nd	Fe-Gd-P	Fe-Gd-Pb	Fe-Gd-Pd	Fe-Gd-Pr
Fe-Gd-Pt	Fe-Gd-Ru	Fe-Gd-Sb	Fe-Gd-Sc	Fe-Gd-Si	Fe-Gd-Sm	Fe-Gd-Sn	Fe-Gd-Sr

Fe-Gd-Ta	Fe-Gd-Tb	Fe-Gd-Ti	Fe-Gd-V	Fe-Gd-W	Fe-Gd-Y	Fe-Gd-Yb	Fe-Gd-Zn
Fe-Ge-Hf	Fe-Ge-La	Fe-Ge-Mg	Fe-Ge-Nd	Fe-Ge-P	Fe-Ge-Pb	Fe-Ge-Pr	Fe-Ge-Pt
Fe-Ge-Ru	Fe-Ge-Sb	Fe-Ge-Sc	Fe-Ge-Sm	Fe-Ge-Sn	Fe-Ge-Sr	Fe-Ge-Tb	Fe-Ge-W
Fe-Ge-Y	Fe-Ge-Yb	Fe-Ge-Zn	Fe-Hf-La	Fe-Hf-Mg	Fe-Hf-Mn	Fe-Hf-Mo	Fe-Hf-Nd
Fe-Hf-Pb	Fe-Hf-Pd	Fe-Hf-Pr	Fe-Hf-Pt	Fe-Hf-Ru	Fe-Hf-Sb	Fe-Hf-Sc	Fe-Hf-Sm
Fe-Hf-Sn	Fe-Hf-Sr	Fe-Hf-Tb	Fe-Hf-W	Fe-Hf-Y	Fe-Hf-Yb	Fe-Hf-Zn	Fe-La-Mg
Fe-La-Nd	Fe-La-P	Fe-La-Pb	Fe-La-Pd	Fe-La-Pr	Fe-La-Pt	Fe-La-Ru	Fe-La-Sb
Fe-La-Sc	Fe-La-Si	Fe-La-Sm	Fe-La-Sn	Fe-La-Sr	Fe-La-Ta	Fe-La-Tb	Fe-La-Ti
Fe-La-V	Fe-La-W	Fe-La-Y	Fe-La-Yb	Fe-La-Zn	Fe-Mg-Mn	Fe-Mg-Mo	Fe-Mg-Nb
Fe-Mg-Nd	Fe-Mg-Ni	Fe-Mg-P	Fe-Mg-Pb	Fe-Mg-Pd	Fe-Mg-Pr	Fe-Mg-Pt	Fe-Mg-Ru
Fe-Mg-Sb	Fe-Mg-Sc	Fe-Mg-Si	Fe-Mg-Sm	Fe-Mg-Sn	Fe-Mg-Sr	Fe-Mg-Ta	Fe-Mg-Tb
Fe-Mg-Ti	Fe-Mg-V	Fe-Mg-W	Fe-Mg-Y	Fe-Mg-Yb	Fe-Mg-Zn	Fe-Mg-Zr	Fe-Mn-Mo
Fe-Mn-Nd	Fe-Mn-Pb	Fe-Mn-Pr	Fe-Mn-Pt	Fe-Mn-Ru	Fe-Mn-Sb	Fe-Mn-Sc	Fe-Mn-Sm
Fe-Mn-Sn	Fe-Mn-Sr	Fe-Mn-Ta	Fe-Mn-Tb	Fe-Mn-Ti	Fe-Mn-V	Fe-Mn-W	Fe-Mn-Yb
Fe-Mn-Zn	Fe-Mo-Nd	Fe-Mo-Pb	Fe-Mo-Pd	Fe-Mo-Pr	Fe-Mo-Pt	Fe-Mo-Sb	Fe-Mo-Sc
Fe-Mo-Sm	Fe-Mo-Sn	Fe-Mo-Sr	Fe-Mo-Ta	Fe-Mo-Tb	Fe-Mo-Ti	Fe-Mo-V	Fe-Mo-W
Fe-Mo-Y	Fe-Mo-Yb	Fe-Mo-Zn	Fe-Nb-Nd	Fe-Nb-P	Fe-Nb-Pb	Fe-Nb-Pr	Fe-Nb-Pt
Fe-Nb-Ru	Fe-Nb-Sb	Fe-Nb-Sc	Fe-Nb-Sm	Fe-Nb-Sr	Fe-Nb-Tb	Fe-Nb-Y	Fe-Nb-Yb
Fe-Nb-Zn	Fe-Nd-P	Fe-Nd-Pb	Fe-Nd-Pd	Fe-Nd-Pr	Fe-Nd-Pt	Fe-Nd-Ru	Fe-Nd-Sb
Fe-Nd-Sc	Fe-Nd-Si	Fe-Nd-Sm	Fe-Nd-Sn	Fe-Nd-Sr	Fe-Nd-Ta	Fe-Nd-Tb	Fe-Nd-Ti
Fe-Nd-V	Fe-Nd-W	Fe-Nd-Y	Fe-Nd-Yb	Fe-Nd-Zn	Fe-Nd-Zr	Fe-Ni-Pb	Fe-Ni-Pt
Fe-Ni-Sb	Fe-Ni-Sc	Fe-Ni-Sr	Fe-Ni-Tb	Fe-Ni-Yb	Fe-Ni-Zn	Fe-P-Pb	Fe-P-Pr
Fe-P-Pt	Fe-P-Sb	Fe-P-Sc	Fe-P-Sm	Fe-P-Sn	Fe-P-Sr	Fe-P-Tb	Fe-P-Y
Fe-P-Yb	Fe-P-Zn	Fe-Pb-Pd	Fe-Pb-Pr	Fe-Pb-Pt	Fe-Pb-Ru	Fe-Pb-Sb	Fe-Pb-Sc
Fe-Pb-Si	Fe-Pb-Sm	Fe-Pb-Sn	Fe-Pb-Sr	Fe-Pb-Ta	Fe-Pb-Tb	Fe-Pb-Ti	Fe-Pb-V

Fe-Pb-W	Fe-Pb-Y	Fe-Pb-Yb	Fe-Pb-Zn	Fe-Pb-Zr	Fe-Pd-Pr	Fe-Pd-Pt	Fe-Pd-Ru
Fe-Pd-Sb	Fe-Pd-Sc	Fe-Pd-Sm	Fe-Pd-Sn	Fe-Pd-Sr	Fe-Pd-Tb	Fe-Pd-V	Fe-Pd-W
Fe-Pd-Y	Fe-Pd-Yb	Fe-Pd-Zn	Fe-Pr-Pt	Fe-Pr-Ru	Fe-Pr-Sb	Fe-Pr-Sc	Fe-Pr-Si
Fe-Pr-Sm	Fe-Pr-Sn	Fe-Pr-Sr	Fe-Pr-Ta	Fe-Pr-Tb	Fe-Pr-Ti	Fe-Pr-V	Fe-Pr-W
Fe-Pr-Y	Fe-Pr-Yb	Fe-Pr-Zn	Fe-Pt-Ru	Fe-Pt-Sb	Fe-Pt-Sc	Fe-Pt-Si	Fe-Pt-Sm
Fe-Pt-Sn	Fe-Pt-Sr	Fe-Pt-Ta	Fe-Pt-Tb	Fe-Pt-Ti	Fe-Pt-V	Fe-Pt-W	Fe-Pt-Y
Fe-Pt-Yb	Fe-Pt-Zn	Fe-Pt-Zr	Fe-Ru-Sb	Fe-Ru-Sc	Fe-Ru-Sm	Fe-Ru-Sn	Fe-Ru-Sr
Fe-Ru-Tb	Fe-Ru-Ti	Fe-Ru-V	Fe-Ru-Y	Fe-Ru-Yb	Fe-Ru-Zn	Fe-Sb-Sc	Fe-Sb-Si
Fe-Sb-Sm	Fe-Sb-Sn	Fe-Sb-Sr	Fe-Sb-Ta	Fe-Sb-Tb	Fe-Sb-Ti	Fe-Sb-V	Fe-Sb-W
Fe-Sb-Y	Fe-Sb-Yb	Fe-Sb-Zn	Fe-Sb-Zr	Fe-Sc-Si	Fe-Sc-Sm	Fe-Sc-Sn	Fe-Sc-Sr
Fe-Sc-Ta	Fe-Sc-Tb	Fe-Sc-Ti	Fe-Sc-V	Fe-Sc-W	Fe-Sc-Y	Fe-Sc-Yb	Fe-Sc-Zn
Fe-Si-Sm	Fe-Si-Sr	Fe-Si-Y	Fe-Si-Yb	Fe-Si-Zn	Fe-Sm-Sn	Fe-Sm-Sr	Fe-Sm-Ta
Fe-Sm-Tb	Fe-Sm-Ti	Fe-Sm-V	Fe-Sm-W	Fe-Sm-Y	Fe-Sm-Yb	Fe-Sm-Zn	Fe-Sm-Zr
Fe-Sn-Sr	Fe-Sn-Ta	Fe-Sn-Tb	Fe-Sn-Ti	Fe-Sn-V	Fe-Sn-W	Fe-Sn-Y	Fe-Sn-Yb
Fe-Sn-Zn	Fe-Sr-Ta	Fe-Sr-Tb	Fe-Sr-Ti	Fe-Sr-V	Fe-Sr-W	Fe-Sr-Y	Fe-Sr-Yb
Fe-Sr-Zn	Fe-Sr-Zr	Fe-Ta-Tb	Fe-Ta-V	Fe-Ta-W	Fe-Ta-Y	Fe-Ta-Yb	Fe-Ta-Zn
Fe-Tb-Ti	Fe-Tb-V	Fe-Tb-W	Fe-Tb-Y	Fe-Tb-Yb	Fe-Tb-Zn	Fe-Tb-Zr	Fe-Ti-W
Fe-Ti-Y	Fe-Ti-Yb	Fe-Ti-Zn	Fe-V-W	Fe-V-Y	Fe-V-Yb	Fe-V-Zn	Fe-W-Y
Fe-W-Yb	Fe-W-Zn	Fe-Y-Yb	Fe-Y-Zn	Fe-Y-Zr	Fe-Yb-Zn	Fe-Yb-Zr	Fe-Zn-Zr
Ga-Gd-Ge	Ga-Gd-Hf	Ga-Gd-La	Ga-Gd-Mn	Ga-Gd-Mo	Ga-Gd-Nb	Ga-Gd-Nd	Ga-Gd-Ni
Ga-Gd-P	Ga-Gd-Pb	Ga-Gd-Pd	Ga-Gd-Pr	Ga-Gd-Pt	Ga-Gd-Ru	Ga-Gd-Sb	Ga-Gd-Sc
Ga-Gd-Si	Ga-Gd-Sm	Ga-Gd-Sn	Ga-Gd-Sr	Ga-Gd-Ta	Ga-Gd-Tb	Ga-Gd-Ti	Ga-Gd-V
Ga-Gd-W	Ga-Gd-Y	Ga-Gd-Yb	Ga-Gd-Zn	Ga-Ge-Hf	Ga-Ge-La	Ga-Ge-Mg	Ga-Ge-Mn
Ga-Ge-Mo	Ga-Ge-Nb	Ga-Ge-Nd	Ga-Ge-Ni	Ga-Ge-P	Ga-Ge-Pb	Ga-Ge-Pd	Ga-Ge-Pr
Ga-Ge-Pt	Ga-Ge-Ru	Ga-Ge-Sb	Ga-Ge-Sc	Ga-Ge-Si	Ga-Ge-Sm	Ga-Ge-Sn	Ga-Ge-Sr

Ga-Ge-Ta	Ga-Ge-Tb	Ga-Ge-Ti	Ga-Ge-V	Ga-Ge-W	Ga-Ge-Y	Ga-Ge-Yb	Ga-Ge-Zn
Ga-Ge-Zr	Ga-Hf-La	Ga-Hf-Mg	Ga-Hf-Mn	Ga-Hf-Mo	Ga-Hf-Nb	Ga-Hf-Nd	Ga-Hf-Ni
Ga-Hf-P	Ga-Hf-Pb	Ga-Hf-Pd	Ga-Hf-Pr	Ga-Hf-Pt	Ga-Hf-Ru	Ga-Hf-Sb	Ga-Hf-Sc
Ga-Hf-Si	Ga-Hf-Sm	Ga-Hf-Sn	Ga-Hf-Sr	Ga-Hf-Ta	Ga-Hf-Tb	Ga-Hf-Ti	Ga-Hf-V
Ga-Hf-W	Ga-Hf-Y	Ga-Hf-Yb	Ga-Hf-Zn	Ga-Hf-Zr	Ga-La-Mg	Ga-La-Mn	Ga-La-Mo
Ga-La-Nb	Ga-La-Nd	Ga-La-Ni	Ga-La-P	Ga-La-Pb	Ga-La-Pd	Ga-La-Pr	Ga-La-Pt
Ga-La-Ru	Ga-La-Sb	Ga-La-Sc	Ga-La-Si	Ga-La-Sm	Ga-La-Sn	Ga-La-Sr	Ga-La-Ta
Ga-La-Tb	Ga-La-Ti	Ga-La-V	Ga-La-W	Ga-La-Y	Ga-La-Yb	Ga-La-Zn	Ga-La-Zr
Ga-Mg-Mn	Ga-Mg-Mo	Ga-Mg-Nb	Ga-Mg-Nd	Ga-Mg-Ni	Ga-Mg-P	Ga-Mg-Pb	Ga-Mg-Pd
Ga-Mg-Pr	Ga-Mg-Pt	Ga-Mg-Ru	Ga-Mg-Sb	Ga-Mg-Sc	Ga-Mg-Si	Ga-Mg-Sm	Ga-Mg-Sn
Ga-Mg-Sr	Ga-Mg-Ta	Ga-Mg-Tb	Ga-Mg-Ti	Ga-Mg-V	Ga-Mg-W	Ga-Mg-Y	Ga-Mg-Yb
Ga-Mg-Zn	Ga-Mg-Zr	Ga-Mn-Mo	Ga-Mn-Nb	Ga-Mn-Nd	Ga-Mn-Ni	Ga-Mn-P	Ga-Mn-Pb
Ga-Mn-Pd	Ga-Mn-Pr	Ga-Mn-Pt	Ga-Mn-Ru	Ga-Mn-Sb	Ga-Mn-Sc	Ga-Mn-Si	Ga-Mn-Sm
Ga-Mn-Sn	Ga-Mn-Sr	Ga-Mn-Ta	Ga-Mn-Tb	Ga-Mn-Ti	Ga-Mn-V	Ga-Mn-W	Ga-Mn-Y
Ga-Mn-Yb	Ga-Mn-Zn	Ga-Mn-Zr	Ga-Mo-Nb	Ga-Mo-Nd	Ga-Mo-Ni	Ga-Mo-P	Ga-Mo-Pb
Ga-Mo-Pd	Ga-Mo-Pr	Ga-Mo-Pt	Ga-Mo-Ru	Ga-Mo-Sb	Ga-Mo-Sc	Ga-Mo-Si	Ga-Mo-Sm
Ga-Mo-Sn	Ga-Mo-Sr	Ga-Mo-Ta	Ga-Mo-Tb	Ga-Mo-Ti	Ga-Mo-V	Ga-Mo-W	Ga-Mo-Y
Ga-Mo-Yb	Ga-Mo-Zn	Ga-Mo-Zr	Ga-Nb-Nd	Ga-Nb-Ni	Ga-Nb-P	Ga-Nb-Pb	Ga-Nb-Pd
Ga-Nb-Pr	Ga-Nb-Pt	Ga-Nb-Ru	Ga-Nb-Sb	Ga-Nb-Sc	Ga-Nb-Si	Ga-Nb-Sm	Ga-Nb-Sn
Ga-Nb-Sr	Ga-Nb-Ta	Ga-Nb-Tb	Ga-Nb-Ti	Ga-Nb-V	Ga-Nb-W	Ga-Nb-Y	Ga-Nb-Yb
Ga-Nb-Zn	Ga-Nb-Zr	Ga-Nd-Ni	Ga-Nd-P	Ga-Nd-Pb	Ga-Nd-Pd	Ga-Nd-Pr	Ga-Nd-Pt
Ga-Nd-Ru	Ga-Nd-Sb	Ga-Nd-Sc	Ga-Nd-Si	Ga-Nd-Sm	Ga-Nd-Sn	Ga-Nd-Sr	Ga-Nd-Ta
Ga-Nd-Tb	Ga-Nd-Ti	Ga-Nd-V	Ga-Nd-W	Ga-Nd-Y	Ga-Nd-Yb	Ga-Nd-Zn	Ga-Nd-Zr
Ga-Ni-P	Ga-Ni-Pb	Ga-Ni-Pd	Ga-Ni-Pr	Ga-Ni-Pt	Ga-Ni-Ru	Ga-Ni-Sb	Ga-Ni-Sc
Ga-Ni-Si	Ga-Ni-Sm	Ga-Ni-Sn	Ga-Ni-Sr	Ga-Ni-Ta	Ga-Ni-Tb	Ga-Ni-Ti	Ga-Ni-V

Ga-Ni-W	Ga-Ni-Y	Ga-Ni-Yb	Ga-Ni-Zn	Ga-Ni-Zr	Ga-P-Pb	Ga-P-Pd	Ga-P-Pr
Ga-P-Pt	Ga-P-Ru	Ga-P-Sb	Ga-P-Sc	Ga-P-Si	Ga-P-Sm	Ga-P-Sn	Ga-P-Sr
Ga-P-Ta	Ga-P-Tb	Ga-P-Ti	Ga-P-V	Ga-P-W	Ga-P-Y	Ga-P-Yb	Ga-P-Zn
Ga-P-Zr	Ga-Pb-Pd	Ga-Pb-Pr	Ga-Pb-Pt	Ga-Pb-Ru	Ga-Pb-Sb	Ga-Pb-Sc	Ga-Pb-Si
Ga-Pb-Sm	Ga-Pb-Sn	Ga-Pb-Sr	Ga-Pb-Ta	Ga-Pb-Tb	Ga-Pb-Ti	Ga-Pb-V	Ga-Pb-W
Ga-Pb-Y	Ga-Pb-Yb	Ga-Pb-Zn	Ga-Pb-Zr	Ga-Pd-Pr	Ga-Pd-Pt	Ga-Pd-Ru	Ga-Pd-Sb
Ga-Pd-Sc	Ga-Pd-Si	Ga-Pd-Sm	Ga-Pd-Sn	Ga-Pd-Sr	Ga-Pd-Ta	Ga-Pd-Tb	Ga-Pd-Ti
Ga-Pd-V	Ga-Pd-W	Ga-Pd-Y	Ga-Pd-Yb	Ga-Pd-Zn	Ga-Pd-Zr	Ga-Pr-Pt	Ga-Pr-Ru
Ga-Pr-Sb	Ga-Pr-Sc	Ga-Pr-Si	Ga-Pr-Sm	Ga-Pr-Sn	Ga-Pr-Sr	Ga-Pr-Ta	Ga-Pr-Tb
Ga-Pr-Ti	Ga-Pr-V	Ga-Pr-W	Ga-Pr-Y	Ga-Pr-Yb	Ga-Pr-Zn	Ga-Pr-Zr	Ga-Pt-Ru
Ga-Pt-Sb	Ga-Pt-Sc	Ga-Pt-Si	Ga-Pt-Sm	Ga-Pt-Sn	Ga-Pt-Sr	Ga-Pt-Ta	Ga-Pt-Tb
Ga-Pt-Ti	Ga-Pt-V	Ga-Pt-W	Ga-Pt-Y	Ga-Pt-Yb	Ga-Pt-Zn	Ga-Pt-Zr	Ga-Ru-Sb
Ga-Ru-Sc	Ga-Ru-Si	Ga-Ru-Sm	Ga-Ru-Sn	Ga-Ru-Sr	Ga-Ru-Ta	Ga-Ru-Tb	Ga-Ru-Ti
Ga-Ru-V	Ga-Ru-W	Ga-Ru-Y	Ga-Ru-Yb	Ga-Ru-Zn	Ga-Ru-Zr	Ga-Sb-Sc	Ga-Sb-Si
Ga-Sb-Sm	Ga-Sb-Sn	Ga-Sb-Sr	Ga-Sb-Ta	Ga-Sb-Tb	Ga-Sb-Ti	Ga-Sb-V	Ga-Sb-W
Ga-Sb-Y	Ga-Sb-Yb	Ga-Sb-Zn	Ga-Sb-Zr	Ga-Sc-Si	Ga-Sc-Sm	Ga-Sc-Sn	Ga-Sc-Sr
Ga-Sc-Ta	Ga-Sc-Tb	Ga-Sc-Ti	Ga-Sc-V	Ga-Sc-W	Ga-Sc-Y	Ga-Sc-Yb	Ga-Sc-Zn
Ga-Sc-Zr	Ga-Si-Sm	Ga-Si-Sn	Ga-Si-Sr	Ga-Si-Ta	Ga-Si-Tb	Ga-Si-Ti	Ga-Si-V
Ga-Si-W	Ga-Si-Y	Ga-Si-Yb	Ga-Si-Zn	Ga-Si-Zr	Ga-Sm-Sn	Ga-Sm-Sr	Ga-Sm-Ta
Ga-Sm-Tb	Ga-Sm-Ti	Ga-Sm-V	Ga-Sm-W	Ga-Sm-Y	Ga-Sm-Yb	Ga-Sm-Zn	Ga-Sm-Zr
Ga-Sn-Sr	Ga-Sn-Ta	Ga-Sn-Tb	Ga-Sn-Ti	Ga-Sn-V	Ga-Sn-W	Ga-Sn-Y	Ga-Sn-Yb
Ga-Sn-Zn	Ga-Sn-Zr	Ga-Sr-Ta	Ga-Sr-Tb	Ga-Sr-Ti	Ga-Sr-V	Ga-Sr-W	Ga-Sr-Y
Ga-Sr-Yb	Ga-Sr-Zn	Ga-Sr-Zr	Ga-Ta-Tb	Ga-Ta-Ti	Ga-Ta-V	Ga-Ta-W	Ga-Ta-Y
Ga-Ta-Yb	Ga-Ta-Zn	Ga-Ta-Zr	Ga-Tb-Ti	Ga-Tb-V	Ga-Tb-W	Ga-Tb-Y	Ga-Tb-Yb
Ga-Tb-Zn	Ga-Tb-Zr	Ga-Ti-V	Ga-Ti-W	Ga-Ti-Y	Ga-Ti-Yb	Ga-Ti-Zn	Ga-Ti-Zr

Ga-V-W	Ga-V-Y	Ga-V-Yb	Ga-V-Zn	Ga-V-Zr	Ga-W-Y	Ga-W-Yb	Ga-W-Zn
Ga-W-Zr	Ga-Y-Yb	Ga-Y-Zn	Ga-Y-Zr	Ga-Yb-Zn	Ga-Yb-Zr	Ga-Zn-Zr	Gd-Ge-Hf
Gd-Ge-La	Gd-Ge-Mg	Gd-Ge-Mn	Gd-Ge-Mo	Gd-Ge-Nb	Gd-Ge-Nd	Gd-Ge-Ni	Gd-Ge-P
Gd-Ge-Pb	Gd-Ge-Pd	Gd-Ge-Pr	Gd-Ge-Pt	Gd-Ge-Ru	Gd-Ge-Sb	Gd-Ge-Sc	Gd-Ge-Si
Gd-Ge-Sm	Gd-Ge-Sn	Gd-Ge-Sr	Gd-Ge-Ta	Gd-Ge-Tb	Gd-Ge-Ti	Gd-Ge-V	Gd-Ge-W
Gd-Ge-Y	Gd-Ge-Yb	Gd-Ge-Zn	Gd-Ge-Zr	Gd-Hf-La	Gd-Hf-Mg	Gd-Hf-Mn	Gd-Hf-Mo
Gd-Hf-Nb	Gd-Hf-Nd	Gd-Hf-Ni	Gd-Hf-P	Gd-Hf-Pb	Gd-Hf-Pd	Gd-Hf-Pr	Gd-Hf-Pt
Gd-Hf-Ru	Gd-Hf-Sb	Gd-Hf-Sc	Gd-Hf-Si	Gd-Hf-Sm	Gd-Hf-Sn	Gd-Hf-Sr	Gd-Hf-Ta
Gd-Hf-Tb	Gd-Hf-Ti	Gd-Hf-V	Gd-Hf-W	Gd-Hf-Y	Gd-Hf-Yb	Gd-Hf-Zn	Gd-Hf-Zr
Gd-La-Mg	Gd-La-Mn	Gd-La-Mo	Gd-La-Nb	Gd-La-Nd	Gd-La-Ni	Gd-La-P	Gd-La-Pb
Gd-La-Pd	Gd-La-Pr	Gd-La-Pt	Gd-La-Ru	Gd-La-Sb	Gd-La-Sc	Gd-La-Si	Gd-La-Sm
Gd-La-Sn	Gd-La-Sr	Gd-La-Ta	Gd-La-Tb	Gd-La-Ti	Gd-La-V	Gd-La-W	Gd-La-Y
Gd-La-Yb	Gd-La-Zn	Gd-La-Zr	Gd-Mg-Mn	Gd-Mg-Mo	Gd-Mg-Nb	Gd-Mg-Nd	Gd-Mg-P
Gd-Mg-Pb	Gd-Mg-Pd	Gd-Mg-Pr	Gd-Mg-Pt	Gd-Mg-Ru	Gd-Mg-Sb	Gd-Mg-Sc	Gd-Mg-Si
Gd-Mg-Sm	Gd-Mg-Sn	Gd-Mg-Sr	Gd-Mg-Ta	Gd-Mg-Tb	Gd-Mg-Ti	Gd-Mg-V	Gd-Mg-W
Gd-Mg-Y	Gd-Mg-Yb	Gd-Mg-Zn	Gd-Mg-Zr	Gd-Mn-Mo	Gd-Mn-Nb	Gd-Mn-Nd	Gd-Mn-Ni
Gd-Mn-P	Gd-Mn-Pb	Gd-Mn-Pd	Gd-Mn-Pr	Gd-Mn-Pt	Gd-Mn-Ru	Gd-Mn-Sb	Gd-Mn-Sc
Gd-Mn-Si	Gd-Mn-Sm	Gd-Mn-Sn	Gd-Mn-Sr	Gd-Mn-Ta	Gd-Mn-Tb	Gd-Mn-Ti	Gd-Mn-V
Gd-Mn-W	Gd-Mn-Y	Gd-Mn-Yb	Gd-Mn-Zn	Gd-Mn-Zr	Gd-Mo-Nb	Gd-Mo-Nd	Gd-Mo-P
Gd-Mo-Pb	Gd-Mo-Pd	Gd-Mo-Pr	Gd-Mo-Pt	Gd-Mo-Ru	Gd-Mo-Sb	Gd-Mo-Sc	Gd-Mo-Si
Gd-Mo-Sm	Gd-Mo-Sn	Gd-Mo-Sr	Gd-Mo-Ta	Gd-Mo-Tb	Gd-Mo-Ti	Gd-Mo-V	Gd-Mo-W
Gd-Mo-Y	Gd-Mo-Yb	Gd-Mo-Zn	Gd-Nb-Nd	Gd-Nb-Ni	Gd-Nb-P	Gd-Nb-Pb	Gd-Nb-Pd
Gd-Nb-Pr	Gd-Nb-Pt	Gd-Nb-Ru	Gd-Nb-Sb	Gd-Nb-Sc	Gd-Nb-Si	Gd-Nb-Sm	Gd-Nb-Sn
Gd-Nb-Sr	Gd-Nb-Ta	Gd-Nb-Tb	Gd-Nb-Ti	Gd-Nb-V	Gd-Nb-W	Gd-Nb-Y	Gd-Nb-Yb
Gd-Nb-Zn	Gd-Nb-Zr	Gd-Nd-Ni	Gd-Nd-P	Gd-Nd-Pb	Gd-Nd-Pd	Gd-Nd-Pr	Gd-Nd-Pt

Gd-Nd-Ru	Gd-Nd-Sb	Gd-Nd-Sc	Gd-Nd-Si	Gd-Nd-Sm	Gd-Nd-Sn	Gd-Nd-Sr	Gd-Nd-Ta
Gd-Nd-Tb	Gd-Nd-Ti	Gd-Nd-V	Gd-Nd-W	Gd-Nd-Y	Gd-Nd-Yb	Gd-Nd-Zn	Gd-Nd-Zr
Gd-Ni-P	Gd-Ni-Pb	Gd-Ni-Pd	Gd-Ni-Pr	Gd-Ni-Pt	Gd-Ni-Ru	Gd-Ni-Sb	Gd-Ni-Sc
Gd-Ni-Si	Gd-Ni-Sm	Gd-Ni-Sn	Gd-Ni-Sr	Gd-Ni-Ta	Gd-Ni-Tb	Gd-Ni-Ti	Gd-Ni-V
Gd-Ni-W	Gd-Ni-Y	Gd-Ni-Yb	Gd-Ni-Zn	Gd-P-Pb	Gd-P-Pd	Gd-P-Pr	Gd-P-Pt
Gd-P-Ru	Gd-P-Sb	Gd-P-Sc	Gd-P-Si	Gd-P-Sm	Gd-P-Sn	Gd-P-Sr	Gd-P-Ta
Gd-P-Tb	Gd-P-Ti	Gd-P-V	Gd-P-W	Gd-P-Y	Gd-P-Yb	Gd-P-Zn	Gd-P-Zr
Gd-Pb-Pd	Gd-Pb-Pr	Gd-Pb-Pt	Gd-Pb-Ru	Gd-Pb-Sb	Gd-Pb-Sc	Gd-Pb-Si	Gd-Pb-Sm
Gd-Pb-Sn	Gd-Pb-Sr	Gd-Pb-Ta	Gd-Pb-Tb	Gd-Pb-Ti	Gd-Pb-V	Gd-Pb-W	Gd-Pb-Y
Gd-Pb-Yb	Gd-Pb-Zn	Gd-Pb-Zr	Gd-Pd-Pr	Gd-Pd-Pt	Gd-Pd-Ru	Gd-Pd-Sb	Gd-Pd-Sc
Gd-Pd-Si	Gd-Pd-Sm	Gd-Pd-Sn	Gd-Pd-Sr	Gd-Pd-Ta	Gd-Pd-Tb	Gd-Pd-Ti	Gd-Pd-V
Gd-Pd-W	Gd-Pd-Y	Gd-Pd-Yb	Gd-Pd-Zn	Gd-Pd-Zr	Gd-Pr-Pt	Gd-Pr-Ru	Gd-Pr-Sb
Gd-Pr-Sc	Gd-Pr-Si	Gd-Pr-Sm	Gd-Pr-Sn	Gd-Pr-Sr	Gd-Pr-Ta	Gd-Pr-Tb	Gd-Pr-Ti
Gd-Pr-V	Gd-Pr-W	Gd-Pr-Y	Gd-Pr-Yb	Gd-Pr-Zn	Gd-Pr-Zr	Gd-Pt-Ru	Gd-Pt-Sb
Gd-Pt-Sc	Gd-Pt-Si	Gd-Pt-Sm	Gd-Pt-Sn	Gd-Pt-Sr	Gd-Pt-Ta	Gd-Pt-Tb	Gd-Pt-Ti
Gd-Pt-V	Gd-Pt-W	Gd-Pt-Y	Gd-Pt-Yb	Gd-Pt-Zn	Gd-Pt-Zr	Gd-Ru-Sb	Gd-Ru-Sc
Gd-Ru-Si	Gd-Ru-Sm	Gd-Ru-Sn	Gd-Ru-Sr	Gd-Ru-Ta	Gd-Ru-Tb	Gd-Ru-Ti	Gd-Ru-V
Gd-Ru-W	Gd-Ru-Y	Gd-Ru-Yb	Gd-Ru-Zn	Gd-Ru-Zr	Gd-Sb-Sc	Gd-Sb-Si	Gd-Sb-Sm
Gd-Sb-Sn	Gd-Sb-Sr	Gd-Sb-Ta	Gd-Sb-Tb	Gd-Sb-Ti	Gd-Sb-V	Gd-Sb-W	Gd-Sb-Y
Gd-Sb-Yb	Gd-Sb-Zn	Gd-Sb-Zr	Gd-Sc-Si	Gd-Sc-Sm	Gd-Sc-Sn	Gd-Sc-Sr	Gd-Sc-Ta
Gd-Sc-Tb	Gd-Sc-Ti	Gd-Sc-V	Gd-Sc-W	Gd-Sc-Y	Gd-Sc-Yb	Gd-Sc-Zn	Gd-Sc-Zr
Gd-Si-Sm	Gd-Si-Sn	Gd-Si-Sr	Gd-Si-Ta	Gd-Si-Tb	Gd-Si-Ti	Gd-Si-V	Gd-Si-W
Gd-Si-Y	Gd-Si-Yb	Gd-Si-Zn	Gd-Si-Zr	Gd-Sm-Sn	Gd-Sm-Sr	Gd-Sm-Ta	Gd-Sm-Tb
Gd-Sm-Ti	Gd-Sm-V	Gd-Sm-W	Gd-Sm-Y	Gd-Sm-Yb	Gd-Sm-Zn	Gd-Sm-Zr	Gd-Sn-Sr
Gd-Sn-Ta	Gd-Sn-Tb	Gd-Sn-Ti	Gd-Sn-V	Gd-Sn-W	Gd-Sn-Y	Gd-Sn-Yb	Gd-Sn-Zn

Gd-Sn-Zr	Gd-Sr-Ta	Gd-Sr-Tb	Gd-Sr-Ti	Gd-Sr-V	Gd-Sr-W	Gd-Sr-Y	Gd-Sr-Yb
Gd-Sr-Zn	Gd-Sr-Zr	Gd-Ta-Tb	Gd-Ta-Ti	Gd-Ta-V	Gd-Ta-W	Gd-Ta-Y	Gd-Ta-Yb
Gd-Ta-Zn	Gd-Ta-Zr	Gd-Tb-Ti	Gd-Tb-V	Gd-Tb-W	Gd-Tb-Y	Gd-Tb-Yb	Gd-Tb-Zn
Gd-Tb-Zr	Gd-Ti-V	Gd-Ti-W	Gd-Ti-Y	Gd-Ti-Yb	Gd-Ti-Zn	Gd-Ti-Zr	Gd-V-W
Gd-V-Y	Gd-V-Yb	Gd-V-Zn	Gd-V-Zr	Gd-W-Y	Gd-W-Yb	Gd-W-Zn	Gd-W-Zr
Gd-Y-Yb	Gd-Y-Zn	Gd-Y-Zr	Gd-Yb-Zn	Gd-Yb-Zr	Gd-Zn-Zr	Ge-Hf-La	Ge-Hf-Mg
Ge-Hf-Mn	Ge-Hf-Mo	Ge-Hf-Nb	Ge-Hf-Nd	Ge-Hf-Ni	Ge-Hf-P	Ge-Hf-Pb	Ge-Hf-Pd
Ge-Hf-Pr	Ge-Hf-Pt	Ge-Hf-Ru	Ge-Hf-Sb	Ge-Hf-Sc	Ge-Hf-Si	Ge-Hf-Sm	Ge-Hf-Sn
Ge-Hf-Sr	Ge-Hf-Ta	Ge-Hf-Tb	Ge-Hf-Ti	Ge-Hf-V	Ge-Hf-W	Ge-Hf-Y	Ge-Hf-Yb
Ge-Hf-Zn	Ge-Hf-Zr	Ge-La-Mg	Ge-La-Mn	Ge-La-Mo	Ge-La-Nb	Ge-La-Nd	Ge-La-Ni
Ge-La-P	Ge-La-Pb	Ge-La-Pd	Ge-La-Pr	Ge-La-Pt	Ge-La-Ru	Ge-La-Sb	Ge-La-Sc
Ge-La-Si	Ge-La-Sm	Ge-La-Sn	Ge-La-Sr	Ge-La-Ta	Ge-La-Tb	Ge-La-Ti	Ge-La-V
Ge-La-W	Ge-La-Y	Ge-La-Yb	Ge-La-Zn	Ge-La-Zr	Ge-Mg-Mn	Ge-Mg-Mo	Ge-Mg-Nb
Ge-Mg-Nd	Ge-Mg-Ni	Ge-Mg-P	Ge-Mg-Pb	Ge-Mg-Pd	Ge-Mg-Pr	Ge-Mg-Pt	Ge-Mg-Ru
Ge-Mg-Sb	Ge-Mg-Sc	Ge-Mg-Si	Ge-Mg-Sm	Ge-Mg-Sn	Ge-Mg-Sr	Ge-Mg-Ta	Ge-Mg-Tb
Ge-Mg-Ti	Ge-Mg-V	Ge-Mg-W	Ge-Mg-Y	Ge-Mg-Yb	Ge-Mg-Zn	Ge-Mg-Zr	Ge-Mn-Mo
Ge-Mn-Nd	Ge-Mn-P	Ge-Mn-Pb	Ge-Mn-Pr	Ge-Mn-Pt	Ge-Mn-Ru	Ge-Mn-Sb	Ge-Mn-Sc
Ge-Mn-Sm	Ge-Mn-Sn	Ge-Mn-Sr	Ge-Mn-Ta	Ge-Mn-Tb	Ge-Mn-Ti	Ge-Mn-V	Ge-Mn-W
Ge-Mn-Y	Ge-Mn-Yb	Ge-Mn-Zn	Ge-Mo-Nd	Ge-Mo-P	Ge-Mo-Pb	Ge-Mo-Pd	Ge-Mo-Pr
Ge-Mo-Pt	Ge-Mo-Ru	Ge-Mo-Sb	Ge-Mo-Sc	Ge-Mo-Sm	Ge-Mo-Sn	Ge-Mo-Sr	Ge-Mo-Ta
Ge-Mo-Tb	Ge-Mo-Ti	Ge-Mo-V	Ge-Mo-W	Ge-Mo-Y	Ge-Mo-Yb	Ge-Mo-Zn	Ge-Nb-Nd
Ge-Nb-P	Ge-Nb-Pb	Ge-Nb-Pr	Ge-Nb-Pt	Ge-Nb-Ru	Ge-Nb-Sb	Ge-Nb-Sc	Ge-Nb-Sm
Ge-Nb-Sn	Ge-Nb-Sr	Ge-Nb-Tb	Ge-Nb-W	Ge-Nb-Y	Ge-Nb-Yb	Ge-Nb-Zn	Ge-Nd-Ni
Ge-Nd-P	Ge-Nd-Pb	Ge-Nd-Pd	Ge-Nd-Pr	Ge-Nd-Pt	Ge-Nd-Ru	Ge-Nd-Sb	Ge-Nd-Sc
Ge-Nd-Si	Ge-Nd-Sm	Ge-Nd-Sn	Ge-Nd-Sr	Ge-Nd-Ta	Ge-Nd-Tb	Ge-Nd-Ti	Ge-Nd-V

Ge-Nd-W	Ge-Nd-Y	Ge-Nd-Yb	Ge-Nd-Zn	Ge-Nd-Zr	Ge-Ni-P	Ge-Ni-Pb	Ge-Ni-Pr
Ge-Ni-Pt	Ge-Ni-Ru	Ge-Ni-Sb	Ge-Ni-Sc	Ge-Ni-Sm	Ge-Ni-Sn	Ge-Ni-Sr	Ge-Ni-Tb
Ge-Ni-W	Ge-Ni-Y	Ge-Ni-Yb	Ge-Ni-Zn	Ge-P-Pb	Ge-P-Pd	Ge-P-Pr	Ge-P-Pt
Ge-P-Ru	Ge-P-Sb	Ge-P-Sc	Ge-P-Si	Ge-P-Sm	Ge-P-Sn	Ge-P-Sr	Ge-P-Ta
Ge-P-Tb	Ge-P-Ti	Ge-P-V	Ge-P-W	Ge-P-Y	Ge-P-Yb	Ge-P-Zn	Ge-P-Zr
Ge-Pb-Pd	Ge-Pb-Pr	Ge-Pb-Pt	Ge-Pb-Ru	Ge-Pb-Sb	Ge-Pb-Sc	Ge-Pb-Si	Ge-Pb-Sm
Ge-Pb-Sn	Ge-Pb-Sr	Ge-Pb-Ta	Ge-Pb-Tb	Ge-Pb-Ti	Ge-Pb-V	Ge-Pb-W	Ge-Pb-Y
Ge-Pb-Yb	Ge-Pb-Zn	Ge-Pb-Zr	Ge-Pd-Pr	Ge-Pd-Pt	Ge-Pd-Ru	Ge-Pd-Sb	Ge-Pd-Sc
Ge-Pd-Sm	Ge-Pd-Sn	Ge-Pd-Sr	Ge-Pd-Tb	Ge-Pd-V	Ge-Pd-W	Ge-Pd-Y	Ge-Pd-Yb
Ge-Pd-Zn	Ge-Pr-Pt	Ge-Pr-Ru	Ge-Pr-Sb	Ge-Pr-Sc	Ge-Pr-Si	Ge-Pr-Sm	Ge-Pr-Sn
Ge-Pr-Sr	Ge-Pr-Ta	Ge-Pr-Tb	Ge-Pr-Ti	Ge-Pr-V	Ge-Pr-W	Ge-Pr-Y	Ge-Pr-Yb
Ge-Pr-Zn	Ge-Pr-Zr	Ge-Pt-Ru	Ge-Pt-Sb	Ge-Pt-Sc	Ge-Pt-Si	Ge-Pt-Sm	Ge-Pt-Sn
Ge-Pt-Sr	Ge-Pt-Ta	Ge-Pt-Tb	Ge-Pt-Ti	Ge-Pt-V	Ge-Pt-W	Ge-Pt-Y	Ge-Pt-Yb
Ge-Pt-Zn	Ge-Pt-Zr	Ge-Ru-Sb	Ge-Ru-Sc	Ge-Ru-Si	Ge-Ru-Sm	Ge-Ru-Sn	Ge-Ru-Sr
Ge-Ru-Ta	Ge-Ru-Tb	Ge-Ru-Ti	Ge-Ru-V	Ge-Ru-W	Ge-Ru-Y	Ge-Ru-Yb	Ge-Ru-Zn
Ge-Ru-Zr	Ge-Sb-Sc	Ge-Sb-Si	Ge-Sb-Sm	Ge-Sb-Sn	Ge-Sb-Sr	Ge-Sb-Ta	Ge-Sb-Tb
Ge-Sb-Ti	Ge-Sb-V	Ge-Sb-W	Ge-Sb-Y	Ge-Sb-Yb	Ge-Sb-Zn	Ge-Sb-Zr	Ge-Sc-Si
Ge-Sc-Sm	Ge-Sc-Sn	Ge-Sc-Sr	Ge-Sc-Ta	Ge-Sc-Tb	Ge-Sc-Ti	Ge-Sc-V	Ge-Sc-W
Ge-Sc-Y	Ge-Sc-Yb	Ge-Sc-Zn	Ge-Sc-Zr	Ge-Si-Sm	Ge-Si-Sn	Ge-Si-Sr	Ge-Si-Tb
Ge-Si-W	Ge-Si-Y	Ge-Si-Yb	Ge-Si-Zn	Ge-Sm-Sn	Ge-Sm-Sr	Ge-Sm-Ta	Ge-Sm-Tb
Ge-Sm-Ti	Ge-Sm-V	Ge-Sm-W	Ge-Sm-Y	Ge-Sm-Yb	Ge-Sm-Zn	Ge-Sm-Zr	Ge-Sn-Sr
Ge-Sn-Ta	Ge-Sn-Tb	Ge-Sn-Ti	Ge-Sn-V	Ge-Sn-W	Ge-Sn-Y	Ge-Sn-Yb	Ge-Sn-Zn
Ge-Sn-Zr	Ge-Sr-Ta	Ge-Sr-Tb	Ge-Sr-Ti	Ge-Sr-V	Ge-Sr-W	Ge-Sr-Y	Ge-Sr-Yb
Ge-Sr-Zn	Ge-Sr-Zr	Ge-Ta-Tb	Ge-Ta-V	Ge-Ta-W	Ge-Ta-Y	Ge-Ta-Yb	Ge-Ta-Zn
Ge-Tb-Ti	Ge-Tb-V	Ge-Tb-W	Ge-Tb-Y	Ge-Tb-Yb	Ge-Tb-Zn	Ge-Tb-Zr	Ge-Ti-W

Ge-Ti-Y	Ge-Ti-Yb	Ge-Ti-Zn	Ge-V-W	Ge-V-Y	Ge-V-Yb	Ge-V-Zn	Ge-W-Y
Ge-W-Yb	Ge-W-Zn	Ge-W-Zr	Ge-Y-Yb	Ge-Y-Zn	Ge-Y-Zr	Ge-Yb-Zn	Ge-Yb-Zr
Ge-Zn-Zr	Hf-La-Mg	Hf-La-Mn	Hf-La-Mo	Hf-La-Nb	Hf-La-Nd	Hf-La-Ni	Hf-La-P
Hf-La-Pb	Hf-La-Pd	Hf-La-Pr	Hf-La-Pt	Hf-La-Ru	Hf-La-Sb	Hf-La-Sc	Hf-La-Si
Hf-La-Sm	Hf-La-Sn	Hf-La-Sr	Hf-La-Ta	Hf-La-Tb	Hf-La-Ti	Hf-La-V	Hf-La-W
Hf-La-Y	Hf-La-Yb	Hf-La-Zn	Hf-La-Zr	Hf-Mg-Mn	Hf-Mg-Mo	Hf-Mg-Nb	Hf-Mg-Nd
Hf-Mg-Ni	Hf-Mg-P	Hf-Mg-Pb	Hf-Mg-Pd	Hf-Mg-Pr	Hf-Mg-Pt	Hf-Mg-Ru	Hf-Mg-Sb
Hf-Mg-Sc	Hf-Mg-Si	Hf-Mg-Sm	Hf-Mg-Sn	Hf-Mg-Sr	Hf-Mg-Ta	Hf-Mg-Tb	Hf-Mg-Ti
Hf-Mg-V	Hf-Mg-W	Hf-Mg-Y	Hf-Mg-Yb	Hf-Mg-Zn	Hf-Mg-Zr	Hf-Mn-Mo	Hf-Mn-Nb
Hf-Mn-Nd	Hf-Mn-Ni	Hf-Mn-P	Hf-Mn-Pb	Hf-Mn-Pd	Hf-Mn-Pr	Hf-Mn-Pt	Hf-Mn-Ru
Hf-Mn-Sb	Hf-Mn-Sc	Hf-Mn-Si	Hf-Mn-Sm	Hf-Mn-Sn	Hf-Mn-Sr	Hf-Mn-Ta	Hf-Mn-Tb
Hf-Mn-Ti	Hf-Mn-V	Hf-Mn-W	Hf-Mn-Y	Hf-Mn-Yb	Hf-Mn-Zn	Hf-Mn-Zr	Hf-Mo-Nb
Hf-Mo-Nd	Hf-Mo-Ni	Hf-Mo-P	Hf-Mo-Pb	Hf-Mo-Pd	Hf-Mo-Pr	Hf-Mo-Pt	Hf-Mo-Ru
Hf-Mo-Sb	Hf-Mo-Sc	Hf-Mo-Si	Hf-Mo-Sm	Hf-Mo-Sn	Hf-Mo-Sr	Hf-Mo-Ta	Hf-Mo-Tb
Hf-Mo-Ti	Hf-Mo-V	Hf-Mo-W	Hf-Mo-Y	Hf-Mo-Yb	Hf-Mo-Zn	Hf-Mo-Zr	Hf-Nb-Nd
Hf-Nb-P	Hf-Nb-Pb	Hf-Nb-Pd	Hf-Nb-Pr	Hf-Nb-Pt	Hf-Nb-Ru	Hf-Nb-Sb	Hf-Nb-Sc
Hf-Nb-Sm	Hf-Nb-Sn	Hf-Nb-Sr	Hf-Nb-Tb	Hf-Nb-W	Hf-Nb-Y	Hf-Nb-Yb	Hf-Nb-Zn
Hf-Nd-Ni	Hf-Nd-P	Hf-Nd-Pb	Hf-Nd-Pd	Hf-Nd-Pr	Hf-Nd-Pt	Hf-Nd-Ru	Hf-Nd-Sb
Hf-Nd-Sc	Hf-Nd-Si	Hf-Nd-Sm	Hf-Nd-Sn	Hf-Nd-Sr	Hf-Nd-Ta	Hf-Nd-Tb	Hf-Nd-Ti
Hf-Nd-V	Hf-Nd-W	Hf-Nd-Y	Hf-Nd-Yb	Hf-Nd-Zn	Hf-Nd-Zr	Hf-Ni-Pb	Hf-Ni-Pd
Hf-Ni-Pr	Hf-Ni-Pt	Hf-Ni-Ru	Hf-Ni-Sb	Hf-Ni-Sc	Hf-Ni-Sm	Hf-Ni-Sn	Hf-Ni-Sr
Hf-Ni-Tb	Hf-Ni-W	Hf-Ni-Y	Hf-Ni-Yb	Hf-Ni-Zn	Hf-P-Pb	Hf-P-Pd	Hf-P-Pr
Hf-P-Pt	Hf-P-Ru	Hf-P-Sb	Hf-P-Sc	Hf-P-Sm	Hf-P-Sn	Hf-P-Sr	Hf-P-Tb
Hf-P-W	Hf-P-Y	Hf-P-Yb	Hf-P-Zn	Hf-Pb-Pd	Hf-Pb-Pr	Hf-Pb-Pt	Hf-Pb-Ru
Hf-Pb-Sb	Hf-Pb-Sc	Hf-Pb-Si	Hf-Pb-Sm	Hf-Pb-Sn	Hf-Pb-Sr	Hf-Pb-Ta	Hf-Pb-Tb

Hf-Pb-Ti	Hf-Pb-V	Hf-Pb-W	Hf-Pb-Y	Hf-Pb-Yb	Hf-Pb-Zn	Hf-Pb-Zr	Hf-Pd-Pr
Hf-Pd-Pt	Hf-Pd-Ru	Hf-Pd-Sb	Hf-Pd-Sc	Hf-Pd-Si	Hf-Pd-Sm	Hf-Pd-Sn	Hf-Pd-Sr
Hf-Pd-Ta	Hf-Pd-Tb	Hf-Pd-Ti	Hf-Pd-V	Hf-Pd-W	Hf-Pd-Y	Hf-Pd-Yb	Hf-Pd-Zn
Hf-Pd-Zr	Hf-Pr-Pt	Hf-Pr-Ru	Hf-Pr-Sb	Hf-Pr-Sc	Hf-Pr-Si	Hf-Pr-Sm	Hf-Pr-Sn
Hf-Pr-Sr	Hf-Pr-Ta	Hf-Pr-Tb	Hf-Pr-Ti	Hf-Pr-V	Hf-Pr-W	Hf-Pr-Y	Hf-Pr-Yb
Hf-Pr-Zn	Hf-Pr-Zr	Hf-Pt-Ru	Hf-Pt-Sb	Hf-Pt-Sc	Hf-Pt-Si	Hf-Pt-Sm	Hf-Pt-Sn
Hf-Pt-Sr	Hf-Pt-Ta	Hf-Pt-Tb	Hf-Pt-Ti	Hf-Pt-V	Hf-Pt-W	Hf-Pt-Y	Hf-Pt-Yb
Hf-Pt-Zn	Hf-Pt-Zr	Hf-Ru-Sb	Hf-Ru-Sc	Hf-Ru-Si	Hf-Ru-Sm	Hf-Ru-Sn	Hf-Ru-Sr
Hf-Ru-Ta	Hf-Ru-Tb	Hf-Ru-Ti	Hf-Ru-V	Hf-Ru-W	Hf-Ru-Y	Hf-Ru-Yb	Hf-Ru-Zn
Hf-Ru-Zr	Hf-Sb-Sc	Hf-Sb-Si	Hf-Sb-Sm	Hf-Sb-Sn	Hf-Sb-Sr	Hf-Sb-Ta	Hf-Sb-Tb
Hf-Sb-Ti	Hf-Sb-V	Hf-Sb-W	Hf-Sb-Y	Hf-Sb-Yb	Hf-Sb-Zn	Hf-Sb-Zr	Hf-Sc-Si
Hf-Sc-Sm	Hf-Sc-Sn	Hf-Sc-Sr	Hf-Sc-Ta	Hf-Sc-Tb	Hf-Sc-Ti	Hf-Sc-V	Hf-Sc-W
Hf-Sc-Y	Hf-Sc-Yb	Hf-Sc-Zn	Hf-Sc-Zr	Hf-Si-Sm	Hf-Si-Sn	Hf-Si-Sr	Hf-Si-Tb
Hf-Si-W	Hf-Si-Y	Hf-Si-Yb	Hf-Si-Zn	Hf-Sm-Sn	Hf-Sm-Sr	Hf-Sm-Ta	Hf-Sm-Tb
Hf-Sm-Ti	Hf-Sm-V	Hf-Sm-W	Hf-Sm-Y	Hf-Sm-Yb	Hf-Sm-Zn	Hf-Sm-Zr	Hf-Sn-Sr
Hf-Sn-Ta	Hf-Sn-Tb	Hf-Sn-Ti	Hf-Sn-V	Hf-Sn-W	Hf-Sn-Y	Hf-Sn-Yb	Hf-Sn-Zn
Hf-Sn-Zr	Hf-Sr-Ta	Hf-Sr-Tb	Hf-Sr-Ti	Hf-Sr-V	Hf-Sr-W	Hf-Sr-Y	Hf-Sr-Yb
Hf-Sr-Zn	Hf-Sr-Zr	Hf-Ta-Tb	Hf-Ta-V	Hf-Ta-W	Hf-Ta-Y	Hf-Ta-Yb	Hf-Ta-Zn
Hf-Tb-Ti	Hf-Tb-V	Hf-Tb-W	Hf-Tb-Y	Hf-Tb-Yb	Hf-Tb-Zn	Hf-Tb-Zr	Hf-Ti-W
Hf-Ti-Y	Hf-Ti-Yb	Hf-Ti-Zn	Hf-V-W	Hf-V-Y	Hf-V-Yb	Hf-V-Zn	Hf-W-Y
Hf-W-Yb	Hf-W-Zn	Hf-W-Zr	Hf-Y-Yb	Hf-Y-Zn	Hf-Y-Zr	Hf-Yb-Zn	Hf-Yb-Zr
Hf-Zn-Zr	La-Mg-Mo	La-Mg-Nb	La-Mg-Nd	La-Mg-P	La-Mg-Pb	La-Mg-Pd	La-Mg-Pr
La-Mg-Pt	La-Mg-Ru	La-Mg-Sb	La-Mg-Sc	La-Mg-Si	La-Mg-Sm	La-Mg-Sn	La-Mg-Ta
La-Mg-Tb	La-Mg-Ti	La-Mg-V	La-Mg-W	La-Mg-Y	La-Mg-Yb	La-Mg-Zr	La-Mn-Mo
La-Mn-Nd	La-Mn-P	La-Mn-Pb	La-Mn-Pd	La-Mn-Pr	La-Mn-Pt	La-Mn-Ru	La-Mn-Sb

La-Mn-Sc	La-Mn-Si	La-Mn-Sm	La-Mn-Sn	La-Mn-Sr	La-Mn-Ta	La-Mn-Tb	La-Mn-Ti
La-Mn-V	La-Mn-W	La-Mn-Y	La-Mn-Yb	La-Mn-Zn	La-Mo-Nd	La-Mo-P	La-Mo-Pb
La-Mo-Pd	La-Mo-Pr	La-Mo-Pt	La-Mo-Ru	La-Mo-Sb	La-Mo-Sc	La-Mo-Si	La-Mo-Sm
La-Mo-Sn	La-Mo-Sr	La-Mo-Ta	La-Mo-Tb	La-Mo-Ti	La-Mo-V	La-Mo-W	La-Mo-Y
La-Mo-Yb	La-Mo-Zn	La-Nb-Nd	La-Nb-P	La-Nb-Pb	La-Nb-Pd	La-Nb-Pr	La-Nb-Ru
La-Nb-Sb	La-Nb-Sc	La-Nb-Si	La-Nb-Sm	La-Nb-Sn	La-Nb-Sr	La-Nb-Ta	La-Nb-Tb
La-Nb-Ti	La-Nb-V	La-Nb-W	La-Nb-Y	La-Nb-Yb	La-Nb-Zn	La-Nd-Ni	La-Nd-P
La-Nd-Pb	La-Nd-Pd	La-Nd-Pr	La-Nd-Pt	La-Nd-Ru	La-Nd-Sb	La-Nd-Sc	La-Nd-Si
La-Nd-Sm	La-Nd-Sn	La-Nd-Sr	La-Nd-Ta	La-Nd-Tb	La-Nd-Ti	La-Nd-V	La-Nd-W
La-Nd-Y	La-Nd-Yb	La-Nd-Zn	La-Nd-Zr	La-Ni-P	La-Ni-Pb	La-Ni-Pd	La-Ni-Pr
La-Ni-Ru	La-Ni-Sb	La-Ni-Sc	La-Ni-Si	La-Ni-Sm	La-Ni-Sn	La-Ni-Sr	La-Ni-Ta
La-Ni-Tb	La-Ni-Ti	La-Ni-V	La-Ni-W	La-Ni-Y	La-Ni-Yb	La-Ni-Zn	La-P-Pb
La-P-Pd	La-P-Pr	La-P-Pt	La-P-Ru	La-P-Sb	La-P-Sc	La-P-Si	La-P-Sm
La-P-Sn	La-P-Sr	La-P-Ta	La-P-Tb	La-P-Ti	La-P-V	La-P-W	La-P-Y
La-P-Yb	La-P-Zn	La-P-Zr	La-Pb-Pd	La-Pb-Pr	La-Pb-Pt	La-Pb-Ru	La-Pb-Sb
La-Pb-Sc	La-Pb-Si	La-Pb-Sm	La-Pb-Sn	La-Pb-Sr	La-Pb-Ta	La-Pb-Tb	La-Pb-Ti
La-Pb-V	La-Pb-W	La-Pb-Y	La-Pb-Yb	La-Pb-Zn	La-Pb-Zr	La-Pd-Pr	La-Pd-Pt
La-Pd-Ru	La-Pd-Sb	La-Pd-Sc	La-Pd-Si	La-Pd-Sm	La-Pd-Sn	La-Pd-Sr	La-Pd-Ta
La-Pd-Tb	La-Pd-Ti	La-Pd-V	La-Pd-W	La-Pd-Y	La-Pd-Yb	La-Pd-Zn	La-Pd-Zr
La-Pr-Pt	La-Pr-Ru	La-Pr-Sb	La-Pr-Sc	La-Pr-Si	La-Pr-Sm	La-Pr-Sn	La-Pr-Sr
La-Pr-Ta	La-Pr-Tb	La-Pr-Ti	La-Pr-V	La-Pr-W	La-Pr-Y	La-Pr-Yb	La-Pr-Zn
La-Pr-Zr	La-Pt-Ru	La-Pt-Sb	La-Pt-Sc	La-Pt-Si	La-Pt-Sm	La-Pt-Sn	La-Pt-Sr
La-Pt-Ta	La-Pt-Tb	La-Pt-Ti	La-Pt-V	La-Pt-W	La-Pt-Y	La-Pt-Yb	La-Pt-Zn
La-Pt-Zr	La-Ru-Sb	La-Ru-Sc	La-Ru-Si	La-Ru-Sm	La-Ru-Sn	La-Ru-Sr	La-Ru-Ta
La-Ru-Tb	La-Ru-Ti	La-Ru-V	La-Ru-W	La-Ru-Y	La-Ru-Yb	La-Ru-Zn	La-Ru-Zr

La-Sb-Sc	La-Sb-Si	La-Sb-Sm	La-Sb-Sn	La-Sb-Sr	La-Sb-Ta	La-Sb-Tb	La-Sb-Ti
La-Sb-V	La-Sb-W	La-Sb-Y	La-Sb-Yb	La-Sb-Zn	La-Sb-Zr	La-Sc-Si	La-Sc-Sm
La-Sc-Sn	La-Sc-Sr	La-Sc-Ta	La-Sc-Tb	La-Sc-Ti	La-Sc-V	La-Sc-W	La-Sc-Y
La-Sc-Yb	La-Sc-Zn	La-Sc-Zr	La-Si-Sm	La-Si-Sn	La-Si-Sr	La-Si-Ta	La-Si-Tb
La-Si-Ti	La-Si-V	La-Si-W	La-Si-Y	La-Si-Yb	La-Si-Zn	La-Si-Zr	La-Sm-Sn
La-Sm-Sr	La-Sm-Ta	La-Sm-Tb	La-Sm-Ti	La-Sm-V	La-Sm-W	La-Sm-Y	La-Sm-Yb
La-Sm-Zn	La-Sm-Zr	La-Sn-Sr	La-Sn-Ta	La-Sn-Tb	La-Sn-Ti	La-Sn-V	La-Sn-W
La-Sn-Y	La-Sn-Yb	La-Sn-Zn	La-Sn-Zr	La-Sr-Ta	La-Sr-Tb	La-Sr-Ti	La-Sr-V
La-Sr-W	La-Sr-Y	La-Sr-Yb	La-Sr-Zr	La-Ta-Tb	La-Ta-Ti	La-Ta-V	La-Ta-W
La-Ta-Y	La-Ta-Yb	La-Ta-Zn	La-Ta-Zr	La-Tb-Ti	La-Tb-V	La-Tb-W	La-Tb-Y
La-Tb-Yb	La-Tb-Zn	La-Tb-Zr	La-Ti-V	La-Ti-W	La-Ti-Y	La-Ti-Yb	La-Ti-Zn
La-Ti-Zr	La-V-W	La-V-Y	La-V-Yb	La-V-Zn	La-V-Zr	La-W-Y	La-W-Yb
La-W-Zn	La-W-Zr	La-Y-Yb	La-Y-Zn	La-Y-Zr	La-Yb-Zn	La-Yb-Zr	La-Zn-Zr
Mg-Mn-Mo	Mg-Mn-Nb	Mg-Mn-Nd	Mg-Mn-P	Mg-Mn-Pb	Mg-Mn-Pr	Mg-Mn-Pt	Mg-Mn-Ru
Mg-Mn-Sb	Mg-Mn-Sc	Mg-Mn-Si	Mg-Mn-Sm	Mg-Mn-Sn	Mg-Mn-Sr	Mg-Mn-Ta	Mg-Mn-Tb
Mg-Mn-Ti	Mg-Mn-V	Mg-Mn-W	Mg-Mn-Yb	Mg-Mn-Zn	Mg-Mn-Zr	Mg-Mo-Nb	Mg-Mo-Nd
Mg-Mo-Ni	Mg-Mo-P	Mg-Mo-Pb	Mg-Mo-Pd	Mg-Mo-Pr	Mg-Mo-Pt	Mg-Mo-Ru	Mg-Mo-Sb
Mg-Mo-Sc	Mg-Mo-Si	Mg-Mo-Sm	Mg-Mo-Sn	Mg-Mo-Sr	Mg-Mo-Ta	Mg-Mo-Tb	Mg-Mo-Ti
Mg-Mo-V	Mg-Mo-W	Mg-Mo-Y	Mg-Mo-Yb	Mg-Mo-Zn	Mg-Mo-Zr	Mg-Nb-Nd	Mg-Nb-Ni
Mg-Nb-P	Mg-Nb-Pb	Mg-Nb-Pd	Mg-Nb-Pr	Mg-Nb-Pt	Mg-Nb-Ru	Mg-Nb-Sb	Mg-Nb-Sc
Mg-Nb-Si	Mg-Nb-Sm	Mg-Nb-Sn	Mg-Nb-Sr	Mg-Nb-Ta	Mg-Nb-Tb	Mg-Nb-Ti	Mg-Nb-V
Mg-Nb-W	Mg-Nb-Y	Mg-Nb-Yb	Mg-Nb-Zn	Mg-Nb-Zr	Mg-Nd-P	Mg-Nd-Pb	Mg-Nd-Pd
Mg-Nd-Pr	Mg-Nd-Pt	Mg-Nd-Ru	Mg-Nd-Sb	Mg-Nd-Sc	Mg-Nd-Si	Mg-Nd-Sm	Mg-Nd-Sn
Mg-Nd-Sr	Mg-Nd-Ta	Mg-Nd-Tb	Mg-Nd-Ti	Mg-Nd-V	Mg-Nd-W	Mg-Nd-Y	Mg-Nd-Yb
Mg-Nd-Zn	Mg-Nd-Zr	Mg-Ni-P	Mg-Ni-Pb	Mg-Ni-Pt	Mg-Ni-Ru	Mg-Ni-Sb	Mg-Ni-Sc

Mg-Ni-Si	Mg-Ni-Sm	Mg-Ni-Sr	Mg-Ni-Ta	Mg-Ni-Tb	Mg-Ni-V	Mg-Ni-W	Mg-Ni-Yb
Mg-Ni-Zn	Mg-Ni-Zr	Mg-P-Pb	Mg-P-Pd	Mg-P-Pr	Mg-P-Pt	Mg-P-Ru	Mg-P-Sb
Mg-P-Sc	Mg-P-Si	Mg-P-Sm	Mg-P-Sn	Mg-P-Sr	Mg-P-Ta	Mg-P-Tb	Mg-P-Ti
Mg-P-V	Mg-P-W	Mg-P-Y	Mg-P-Yb	Mg-P-Zn	Mg-P-Zr	Mg-Pb-Pd	Mg-Pb-Pr
Mg-Pb-Pt	Mg-Pb-Ru	Mg-Pb-Sb	Mg-Pb-Sc	Mg-Pb-Si	Mg-Pb-Sm	Mg-Pb-Sn	Mg-Pb-Sr
Mg-Pb-Ta	Mg-Pb-Tb	Mg-Pb-Ti	Mg-Pb-V	Mg-Pb-W	Mg-Pb-Y	Mg-Pb-Yb	Mg-Pb-Zn
Mg-Pb-Zr	Mg-Pd-Pr	Mg-Pd-Pt	Mg-Pd-Ru	Mg-Pd-Sb	Mg-Pd-Sc	Mg-Pd-Si	Mg-Pd-Sm
Mg-Pd-Sn	Mg-Pd-Sr	Mg-Pd-Ta	Mg-Pd-Tb	Mg-Pd-V	Mg-Pd-W	Mg-Pd-Y	Mg-Pd-Zn
Mg-Pd-Zr	Mg-Pr-Pt	Mg-Pr-Ru	Mg-Pr-Sb	Mg-Pr-Sc	Mg-Pr-Si	Mg-Pr-Sm	Mg-Pr-Sn
Mg-Pr-Sr	Mg-Pr-Ta	Mg-Pr-Tb	Mg-Pr-Ti	Mg-Pr-V	Mg-Pr-W	Mg-Pr-Y	Mg-Pr-Yb
Mg-Pr-Zn	Mg-Pr-Zr	Mg-Pt-Ru	Mg-Pt-Sb	Mg-Pt-Sc	Mg-Pt-Si	Mg-Pt-Sm	Mg-Pt-Sn
Mg-Pt-Sr	Mg-Pt-Ta	Mg-Pt-Tb	Mg-Pt-Ti	Mg-Pt-V	Mg-Pt-W	Mg-Pt-Y	Mg-Pt-Yb
Mg-Pt-Zn	Mg-Pt-Zr	Mg-Ru-Sb	Mg-Ru-Sc	Mg-Ru-Si	Mg-Ru-Sm	Mg-Ru-Sn	Mg-Ru-Sr
Mg-Ru-Ta	Mg-Ru-Tb	Mg-Ru-Ti	Mg-Ru-V	Mg-Ru-W	Mg-Ru-Y	Mg-Ru-Yb	Mg-Ru-Zn
Mg-Ru-Zr	Mg-Sb-Sc	Mg-Sb-Si	Mg-Sb-Sm	Mg-Sb-Sn	Mg-Sb-Sr	Mg-Sb-Ta	Mg-Sb-Tb
Mg-Sb-Ti	Mg-Sb-V	Mg-Sb-W	Mg-Sb-Y	Mg-Sb-Yb	Mg-Sb-Zn	Mg-Sb-Zr	Mg-Sc-Si
Mg-Sc-Sm	Mg-Sc-Sn	Mg-Sc-Sr	Mg-Sc-Ta	Mg-Sc-Tb	Mg-Sc-Ti	Mg-Sc-V	Mg-Sc-W
Mg-Sc-Y	Mg-Sc-Yb	Mg-Sc-Zn	Mg-Sc-Zr	Mg-Si-Sm	Mg-Si-Sn	Mg-Si-Sr	Mg-Si-Ta
Mg-Si-Tb	Mg-Si-Ti	Mg-Si-V	Mg-Si-W	Mg-Si-Y	Mg-Si-Yb	Mg-Si-Zn	Mg-Si-Zr
Mg-Sm-Sn	Mg-Sm-Sr	Mg-Sm-Ta	Mg-Sm-Tb	Mg-Sm-Ti	Mg-Sm-V	Mg-Sm-W	Mg-Sm-Y
Mg-Sm-Yb	Mg-Sm-Zn	Mg-Sm-Zr	Mg-Sn-Sr	Mg-Sn-Ta	Mg-Sn-Tb	Mg-Sn-Ti	Mg-Sn-V
Mg-Sn-W	Mg-Sn-Y	Mg-Sn-Yb	Mg-Sn-Zr	Mg-Sr-Ta	Mg-Sr-Tb	Mg-Sr-Ti	Mg-Sr-V
Mg-Sr-W	Mg-Sr-Y	Mg-Sr-Yb	Mg-Sr-Zr	Mg-Ta-Tb	Mg-Ta-Ti	Mg-Ta-V	Mg-Ta-W
Mg-Ta-Y	Mg-Ta-Yb	Mg-Ta-Zn	Mg-Ta-Zr	Mg-Tb-Ti	Mg-Tb-V	Mg-Tb-W	Mg-Tb-Y
Mg-Tb-Yb	Mg-Tb-Zn	Mg-Tb-Zr	Mg-Ti-V	Mg-Ti-W	Mg-Ti-Y	Mg-Ti-Yb	Mg-Ti-Zn

Mg-Ti-Zr	Mg-V-W	Mg-V-Y	Mg-V-Yb	Mg-V-Zn	Mg-V-Zr	Mg-W-Y	Mg-W-Yb
Mg-W-Zn	Mg-W-Zr	Mg-Y-Yb	Mg-Y-Zn	Mg-Y-Zr	Mg-Yb-Zn	Mg-Yb-Zr	Mg-Zn-Zr
Mn-Mo-Nb	Mn-Mo-Nd	Mn-Mo-Ni	Mn-Mo-P	Mn-Mo-Pb	Mn-Mo-Pd	Mn-Mo-Pr	Mn-Mo-Pt
Mn-Mo-Ru	Mn-Mo-Sb	Mn-Mo-Sc	Mn-Mo-Si	Mn-Mo-Sm	Mn-Mo-Sn	Mn-Mo-Sr	Mn-Mo-Ta
Mn-Mo-Tb	Mn-Mo-Ti	Mn-Mo-V	Mn-Mo-W	Mn-Mo-Y	Mn-Mo-Yb	Mn-Mo-Zn	Mn-Mo-Zr
Mn-Nb-Nd	Mn-Nb-P	Mn-Nb-Pb	Mn-Nb-Pr	Mn-Nb-Pt	Mn-Nb-Ru	Mn-Nb-Sb	Mn-Nb-Sc
Mn-Nb-Sm	Mn-Nb-Sn	Mn-Nb-Sr	Mn-Nb-Ta	Mn-Nb-Tb	Mn-Nb-Ti	Mn-Nb-V	Mn-Nb-W
Mn-Nb-Y	Mn-Nb-Yb	Mn-Nb-Zn	Mn-Nd-Ni	Mn-Nd-P	Mn-Nd-Pb	Mn-Nd-Pd	Mn-Nd-Pr
Mn-Nd-Pt	Mn-Nd-Ru	Mn-Nd-Sb	Mn-Nd-Sc	Mn-Nd-Si	Mn-Nd-Sm	Mn-Nd-Sn	Mn-Nd-Sr
Mn-Nd-Ta	Mn-Nd-Tb	Mn-Nd-Ti	Mn-Nd-V	Mn-Nd-W	Mn-Nd-Y	Mn-Nd-Yb	Mn-Nd-Zn
Mn-Nd-Zr	Mn-Ni-Pb	Mn-Ni-Pr	Mn-Ni-Pt	Mn-Ni-Ru	Mn-Ni-Sb	Mn-Ni-Sc	Mn-Ni-Sm
Mn-Ni-Sn	Mn-Ni-Sr	Mn-Ni-Ta	Mn-Ni-Tb	Mn-Ni-Ti	Mn-Ni-V	Mn-Ni-W	Mn-Ni-Yb
Mn-Ni-Zn	Mn-P-Pb	Mn-P-Pr	Mn-P-Pt	Mn-P-Ru	Mn-P-Sb	Mn-P-Sc	Mn-P-Sm
Mn-P-Sn	Mn-P-Sr	Mn-P-Ta	Mn-P-Tb	Mn-P-Ti	Mn-P-V	Mn-P-W	Mn-P-Y
Mn-P-Yb	Mn-P-Zn	Mn-Pb-Pd	Mn-Pb-Pr	Mn-Pb-Pt	Mn-Pb-Ru	Mn-Pb-Sb	Mn-Pb-Sc
Mn-Pb-Si	Mn-Pb-Sm	Mn-Pb-Sn	Mn-Pb-Sr	Mn-Pb-Ta	Mn-Pb-Tb	Mn-Pb-Ti	Mn-Pb-V
Mn-Pb-W	Mn-Pb-Y	Mn-Pb-Yb	Mn-Pb-Zn	Mn-Pb-Zr	Mn-Pd-Pr	Mn-Pd-Pt	Mn-Pd-Ru
Mn-Pd-Sb	Mn-Pd-Sc	Mn-Pd-Sm	Mn-Pd-Sn	Mn-Pd-Sr	Mn-Pd-Ta	Mn-Pd-Tb	Mn-Pd-Ti
Mn-Pd-V	Mn-Pd-W	Mn-Pd-Y	Mn-Pd-Yb	Mn-Pd-Zn	Mn-Pr-Pt	Mn-Pr-Ru	Mn-Pr-Sb
Mn-Pr-Sc	Mn-Pr-Si	Mn-Pr-Sm	Mn-Pr-Sn	Mn-Pr-Sr	Mn-Pr-Ta	Mn-Pr-Tb	Mn-Pr-Ti
Mn-Pr-V	Mn-Pr-W	Mn-Pr-Y	Mn-Pr-Yb	Mn-Pr-Zn	Mn-Pr-Zr	Mn-Pt-Ru	Mn-Pt-Sb
Mn-Pt-Sc	Mn-Pt-Si	Mn-Pt-Sm	Mn-Pt-Sn	Mn-Pt-Sr	Mn-Pt-Ta	Mn-Pt-Tb	Mn-Pt-Ti
Mn-Pt-V	Mn-Pt-W	Mn-Pt-Y	Mn-Pt-Yb	Mn-Pt-Zn	Mn-Pt-Zr	Mn-Ru-Sb	Mn-Ru-Sc
Mn-Ru-Si	Mn-Ru-Sm	Mn-Ru-Sn	Mn-Ru-Sr	Mn-Ru-Ta	Mn-Ru-Tb	Mn-Ru-Ti	Mn-Ru-V
Mn-Ru-W	Mn-Ru-Y	Mn-Ru-Yb	Mn-Ru-Zn	Mn-Ru-Zr	Mn-Sb-Sc	Mn-Sb-Si	Mn-Sb-Sm

Mn-Sb-Sn	Mn-Sb-Sr	Mn-Sb-Ta	Mn-Sb-Tb	Mn-Sb-Ti	Mn-Sb-V	Mn-Sb-W	Mn-Sb-Y
Mn-Sb-Yb	Mn-Sb-Zn	Mn-Sb-Zr	Mn-Sc-Si	Mn-Sc-Sm	Mn-Sc-Sn	Mn-Sc-Sr	Mn-Sc-Ta
Mn-Sc-Tb	Mn-Sc-Ti	Mn-Sc-V	Mn-Sc-W	Mn-Sc-Y	Mn-Sc-Yb	Mn-Sc-Zn	Mn-Sc-Zr
Mn-Si-Sm	Mn-Si-Sn	Mn-Si-Sr	Mn-Si-Ta	Mn-Si-Tb	Mn-Si-Ti	Mn-Si-V	Mn-Si-W
Mn-Si-Y	Mn-Si-Yb	Mn-Si-Zn	Mn-Sm-Sn	Mn-Sm-Sr	Mn-Sm-Ta	Mn-Sm-Tb	Mn-Sm-Ti
Mn-Sm-V	Mn-Sm-W	Mn-Sm-Y	Mn-Sm-Yb	Mn-Sm-Zn	Mn-Sm-Zr	Mn-Sn-Sr	Mn-Sn-Ta
Mn-Sn-Tb	Mn-Sn-Ti	Mn-Sn-V	Mn-Sn-W	Mn-Sn-Y	Mn-Sn-Yb	Mn-Sn-Zn	Mn-Sn-Zr
Mn-Sr-Ta	Mn-Sr-Tb	Mn-Sr-Ti	Mn-Sr-V	Mn-Sr-W	Mn-Sr-Y	Mn-Sr-Yb	Mn-Sr-Zn
Mn-Sr-Zr	Mn-Ta-Tb	Mn-Ta-Ti	Mn-Ta-V	Mn-Ta-W	Mn-Ta-Y	Mn-Ta-Yb	Mn-Ta-Zn
Mn-Ta-Zr	Mn-Tb-Ti	Mn-Tb-V	Mn-Tb-W	Mn-Tb-Y	Mn-Tb-Yb	Mn-Tb-Zn	Mn-Tb-Zr
Mn-Ti-V	Mn-Ti-W	Mn-Ti-Y	Mn-Ti-Yb	Mn-Ti-Zn	Mn-Ti-Zr	Mn-V-W	Mn-V-Y
Mn-V-Yb	Mn-V-Zn	Mn-V-Zr	Mn-W-Y	Mn-W-Yb	Mn-W-Zn	Mn-W-Zr	Mn-Y-Yb
Mn-Y-Zn	Mn-Y-Zr	Mn-Yb-Zn	Mn-Yb-Zr	Mn-Zn-Zr	Mo-Nb-Nd	Mo-Nb-P	Mo-Nb-Pb
Mo-Nb-Pd	Mo-Nb-Pr	Mo-Nb-Pt	Mo-Nb-Ru	Mo-Nb-Sb	Mo-Nb-Sc	Mo-Nb-Sm	Mo-Nb-Sn
Mo-Nb-Sr	Mo-Nb-Ta	Mo-Nb-Tb	Mo-Nb-Ti	Mo-Nb-V	Mo-Nb-W	Mo-Nb-Y	Mo-Nb-Yb
Mo-Nb-Zn	Mo-Nd-Ni	Mo-Nd-P	Mo-Nd-Pb	Mo-Nd-Pd	Mo-Nd-Pr	Mo-Nd-Pt	Mo-Nd-Ru
Mo-Nd-Sb	Mo-Nd-Sc	Mo-Nd-Si	Mo-Nd-Sm	Mo-Nd-Sn	Mo-Nd-Sr	Mo-Nd-Ta	Mo-Nd-Tb
Mo-Nd-Ti	Mo-Nd-V	Mo-Nd-W	Mo-Nd-Y	Mo-Nd-Yb	Mo-Nd-Zn	Mo-Nd-Zr	Mo-Ni-Pb
Mo-Ni-Pd	Mo-Ni-Pr	Mo-Ni-Pt	Mo-Ni-Sb	Mo-Ni-Sc	Mo-Ni-Sm	Mo-Ni-Sn	Mo-Ni-Sr
Mo-Ni-Ta	Mo-Ni-Tb	Mo-Ni-Ti	Mo-Ni-V	Mo-Ni-W	Mo-Ni-Y	Mo-Ni-Yb	Mo-Ni-Zn
Mo-P-Pb	Mo-P-Pd	Mo-P-Pr	Mo-P-Pt	Mo-P-Sb	Mo-P-Sc	Mo-P-Sm	Mo-P-Sn
Mo-P-Sr	Mo-P-Ta	Mo-P-Tb	Mo-P-Ti	Mo-P-V	Mo-P-W	Mo-P-Y	Mo-P-Yb
Mo-P-Zn	Mo-Pb-Pd	Mo-Pb-Pr	Mo-Pb-Pt	Mo-Pb-Ru	Mo-Pb-Sb	Mo-Pb-Sc	Mo-Pb-Si
Mo-Pb-Sm	Mo-Pb-Sn	Mo-Pb-Sr	Mo-Pb-Ta	Mo-Pb-Tb	Mo-Pb-Ti	Mo-Pb-V	Mo-Pb-W
Mo-Pb-Y	Mo-Pb-Yb	Mo-Pb-Zn	Mo-Pb-Zr	Mo-Pd-Pr	Mo-Pd-Pt	Mo-Pd-Ru	Mo-Pd-Sb

Mo-Pd-Sc	Mo-Pd-Si	Mo-Pd-Sm	Mo-Pd-Sn	Mo-Pd-Sr	Mo-Pd-Ta	Mo-Pd-Tb	Mo-Pd-Ti
Mo-Pd-V	Mo-Pd-W	Mo-Pd-Y	Mo-Pd-Yb	Mo-Pd-Zn	Mo-Pd-Zr	Mo-Pr-Pt	Mo-Pr-Ru
Mo-Pr-Sb	Mo-Pr-Sc	Mo-Pr-Si	Mo-Pr-Sm	Mo-Pr-Sn	Mo-Pr-Sr	Mo-Pr-Ta	Mo-Pr-Tb
Mo-Pr-Ti	Mo-Pr-V	Mo-Pr-W	Mo-Pr-Y	Mo-Pr-Yb	Mo-Pr-Zn	Mo-Pr-Zr	Mo-Pt-Ru
Mo-Pt-Sb	Mo-Pt-Sc	Mo-Pt-Si	Mo-Pt-Sm	Mo-Pt-Sn	Mo-Pt-Sr	Mo-Pt-Ta	Mo-Pt-Tb
Mo-Pt-Ti	Mo-Pt-V	Mo-Pt-W	Mo-Pt-Y	Mo-Pt-Yb	Mo-Pt-Zn	Mo-Pt-Zr	Mo-Ru-Sb
Mo-Ru-Sc	Mo-Ru-Sm	Mo-Ru-Sn	Mo-Ru-Sr	Mo-Ru-Ta	Mo-Ru-Tb	Mo-Ru-Ti	Mo-Ru-V
Mo-Ru-W	Mo-Ru-Y	Mo-Ru-Yb	Mo-Ru-Zn	Mo-Sb-Sc	Mo-Sb-Si	Mo-Sb-Sm	Mo-Sb-Sn
Mo-Sb-Sr	Mo-Sb-Ta	Mo-Sb-Tb	Mo-Sb-Ti	Mo-Sb-V	Mo-Sb-W	Mo-Sb-Y	Mo-Sb-Yb
Mo-Sb-Zn	Mo-Sb-Zr	Mo-Sc-Si	Mo-Sc-Sm	Mo-Sc-Sn	Mo-Sc-Sr	Mo-Sc-Ta	Mo-Sc-Tb
Mo-Sc-Ti	Mo-Sc-V	Mo-Sc-W	Mo-Sc-Y	Mo-Sc-Yb	Mo-Sc-Zn	Mo-Sc-Zr	Mo-Si-Sm
Mo-Si-Sn	Mo-Si-Sr	Mo-Si-Ta	Mo-Si-Tb	Mo-Si-Ti	Mo-Si-V	Mo-Si-W	Mo-Si-Y
Mo-Si-Yb	Mo-Si-Zn	Mo-Sm-Sn	Mo-Sm-Sr	Mo-Sm-Ta	Mo-Sm-Tb	Mo-Sm-Ti	Mo-Sm-V
Mo-Sm-W	Mo-Sm-Y	Mo-Sm-Yb	Mo-Sm-Zn	Mo-Sm-Zr	Mo-Sn-Sr	Mo-Sn-Ta	Mo-Sn-Tb
Mo-Sn-Ti	Mo-Sn-V	Mo-Sn-W	Mo-Sn-Y	Mo-Sn-Yb	Mo-Sn-Zn	Mo-Sn-Zr	Mo-Sr-Ta
Mo-Sr-Tb	Mo-Sr-Ti	Mo-Sr-V	Mo-Sr-W	Mo-Sr-Y	Mo-Sr-Yb	Mo-Sr-Zn	Mo-Sr-Zr
Mo-Ta-Tb	Mo-Ta-Ti	Mo-Ta-V	Mo-Ta-W	Mo-Ta-Y	Mo-Ta-Yb	Mo-Ta-Zn	Mo-Ta-Zr
Mo-Tb-Ti	Mo-Tb-V	Mo-Tb-W	Mo-Tb-Y	Mo-Tb-Yb	Mo-Tb-Zn	Mo-Tb-Zr	Mo-Ti-V
Mo-Ti-W	Mo-Ti-Y	Mo-Ti-Yb	Mo-Ti-Zn	Mo-Ti-Zr	Mo-V-W	Mo-V-Y	Mo-V-Yb
Mo-V-Zn	Mo-V-Zr	Mo-W-Y	Mo-W-Yb	Mo-W-Zn	Mo-W-Zr	Mo-Y-Yb	Mo-Y-Zn
Mo-Y-Zr	Mo-Yb-Zn	Mo-Yb-Zr	Mo-Zn-Zr	Nb-Nd-Ni	Nb-Nd-P	Nb-Nd-Pb	Nb-Nd-Pd
Nb-Nd-Pr	Nb-Nd-Pt	Nb-Nd-Ru	Nb-Nd-Sb	Nb-Nd-Sc	Nb-Nd-Si	Nb-Nd-Sm	Nb-Nd-Sn
Nb-Nd-Sr	Nb-Nd-Ta	Nb-Nd-Tb	Nb-Nd-Ti	Nb-Nd-V	Nb-Nd-W	Nb-Nd-Y	Nb-Nd-Yb
Nb-Nd-Zn	Nb-Nd-Zr	Nb-Ni-P	Nb-Ni-Pb	Nb-Ni-Pr	Nb-Ni-Ru	Nb-Ni-Sb	Nb-Ni-Sc
Nb-Ni-Sm	Nb-Ni-Sr	Nb-Ni-Tb	Nb-Ni-Y	Nb-Ni-Yb	Nb-Ni-Zn	Nb-P-Pb	Nb-P-Pd

Nb-P-Pr	Nb-P-Pt	Nb-P-Ru	Nb-P-Sb	Nb-P-Sc	Nb-P-Si	Nb-P-Sm	Nb-P-Sn
Nb-P-Sr	Nb-P-Ta	Nb-P-Tb	Nb-P-Ti	Nb-P-V	Nb-P-W	Nb-P-Y	Nb-P-Yb
Nb-P-Zn	Nb-P-Zr	Nb-Pb-Pd	Nb-Pb-Pr	Nb-Pb-Pt	Nb-Pb-Ru	Nb-Pb-Sb	Nb-Pb-Sc
Nb-Pb-Si	Nb-Pb-Sm	Nb-Pb-Sn	Nb-Pb-Sr	Nb-Pb-Ta	Nb-Pb-Tb	Nb-Pb-Ti	Nb-Pb-V
Nb-Pb-W	Nb-Pb-Y	Nb-Pb-Yb	Nb-Pb-Zn	Nb-Pb-Zr	Nb-Pd-Pr	Nb-Pd-Pt	Nb-Pd-Ru
Nb-Pd-Sb	Nb-Pd-Sc	Nb-Pd-Sm	Nb-Pd-Sn	Nb-Pd-Sr	Nb-Pd-Tb	Nb-Pd-V	Nb-Pd-W
Nb-Pd-Y	Nb-Pd-Yb	Nb-Pd-Zn	Nb-Pr-Pt	Nb-Pr-Ru	Nb-Pr-Sb	Nb-Pr-Sc	Nb-Pr-Si
Nb-Pr-Sm	Nb-Pr-Sn	Nb-Pr-Sr	Nb-Pr-Ta	Nb-Pr-Tb	Nb-Pr-Ti	Nb-Pr-V	Nb-Pr-W
Nb-Pr-Y	Nb-Pr-Yb	Nb-Pr-Zn	Nb-Pr-Zr	Nb-Pt-Ru	Nb-Pt-Sb	Nb-Pt-Sc	Nb-Pt-Si
Nb-Pt-Sm	Nb-Pt-Sn	Nb-Pt-Sr	Nb-Pt-Ta	Nb-Pt-Tb	Nb-Pt-Ti	Nb-Pt-V	Nb-Pt-W
Nb-Pt-Y	Nb-Pt-Yb	Nb-Pt-Zn	Nb-Pt-Zr	Nb-Ru-Sb	Nb-Ru-Sc	Nb-Ru-Si	Nb-Ru-Sm
Nb-Ru-Sn	Nb-Ru-Sr	Nb-Ru-Ta	Nb-Ru-Tb	Nb-Ru-Ti	Nb-Ru-V	Nb-Ru-W	Nb-Ru-Y
Nb-Ru-Yb	Nb-Ru-Zn	Nb-Ru-Zr	Nb-Sb-Sc	Nb-Sb-Si	Nb-Sb-Sm	Nb-Sb-Sn	Nb-Sb-Sr
Nb-Sb-Ta	Nb-Sb-Tb	Nb-Sb-Ti	Nb-Sb-V	Nb-Sb-W	Nb-Sb-Y	Nb-Sb-Yb	Nb-Sb-Zn
Nb-Sb-Zr	Nb-Sc-Si	Nb-Sc-Sm	Nb-Sc-Sn	Nb-Sc-Sr	Nb-Sc-Ta	Nb-Sc-Tb	Nb-Sc-Ti
Nb-Sc-V	Nb-Sc-W	Nb-Sc-Y	Nb-Sc-Yb	Nb-Sc-Zn	Nb-Sc-Zr	Nb-Si-Sm	Nb-Si-Sr
Nb-Si-Tb	Nb-Si-Y	Nb-Si-Yb	Nb-Si-Zn	Nb-Sm-Sn	Nb-Sm-Sr	Nb-Sm-Ta	Nb-Sm-Tb
Nb-Sm-Ti	Nb-Sm-V	Nb-Sm-W	Nb-Sm-Y	Nb-Sm-Yb	Nb-Sm-Zn	Nb-Sm-Zr	Nb-Sn-Sr
Nb-Sn-Ta	Nb-Sn-Tb	Nb-Sn-Ti	Nb-Sn-V	Nb-Sn-W	Nb-Sn-Y	Nb-Sn-Yb	Nb-Sn-Zn
Nb-Sr-Ta	Nb-Sr-Tb	Nb-Sr-Ti	Nb-Sr-V	Nb-Sr-W	Nb-Sr-Y	Nb-Sr-Yb	Nb-Sr-Zn
Nb-Sr-Zr	Nb-Ta-Tb	Nb-Ta-V	Nb-Ta-W	Nb-Ta-Y	Nb-Ta-Yb	Nb-Ta-Zn	Nb-Tb-Ti
Nb-Tb-V	Nb-Tb-W	Nb-Tb-Y	Nb-Tb-Yb	Nb-Tb-Zn	Nb-Tb-Zr	Nb-Ti-W	Nb-Ti-Y
Nb-Ti-Yb	Nb-Ti-Zn	Nb-V-W	Nb-V-Y	Nb-V-Yb	Nb-V-Zn	Nb-W-Y	Nb-W-Yb
Nb-W-Zn	Nb-Y-Yb	Nb-Y-Zn	Nb-Y-Zr	Nb-Yb-Zn	Nb-Yb-Zr	Nb-Zn-Zr	Nd-Ni-P
Nd-Ni-Pb	Nd-Ni-Pd	Nd-Ni-Pr	Nd-Ni-Pt	Nd-Ni-Ru	Nd-Ni-Sb	Nd-Ni-Sc	Nd-Ni-Si

Nd-Ni-Sm	Nd-Ni-Sn	Nd-Ni-Sr	Nd-Ni-Ta	Nd-Ni-Tb	Nd-Ni-Ti	Nd-Ni-V	Nd-Ni-W
Nd-Ni-Y	Nd-Ni-Yb	Nd-Ni-Zn	Nd-Ni-Zr	Nd-P-Pb	Nd-P-Pd	Nd-P-Pr	Nd-P-Pt
Nd-P-Ru	Nd-P-Sb	Nd-P-Sc	Nd-P-Si	Nd-P-Sm	Nd-P-Sn	Nd-P-Sr	Nd-P-Ta
Nd-P-Tb	Nd-P-Ti	Nd-P-V	Nd-P-W	Nd-P-Y	Nd-P-Yb	Nd-P-Zn	Nd-P-Zr
Nd-Pb-Pd	Nd-Pb-Pr	Nd-Pb-Pt	Nd-Pb-Ru	Nd-Pb-Sb	Nd-Pb-Sc	Nd-Pb-Si	Nd-Pb-Sm
Nd-Pb-Sn	Nd-Pb-Sr	Nd-Pb-Ta	Nd-Pb-Tb	Nd-Pb-Ti	Nd-Pb-V	Nd-Pb-W	Nd-Pb-Y
Nd-Pb-Yb	Nd-Pb-Zn	Nd-Pb-Zr	Nd-Pd-Pr	Nd-Pd-Pt	Nd-Pd-Ru	Nd-Pd-Sb	Nd-Pd-Sc
Nd-Pd-Si	Nd-Pd-Sm	Nd-Pd-Sn	Nd-Pd-Sr	Nd-Pd-Ta	Nd-Pd-Tb	Nd-Pd-Ti	Nd-Pd-V
Nd-Pd-W	Nd-Pd-Y	Nd-Pd-Yb	Nd-Pd-Zn	Nd-Pd-Zr	Nd-Pr-Pt	Nd-Pr-Ru	Nd-Pr-Sb
Nd-Pr-Sc	Nd-Pr-Si	Nd-Pr-Sm	Nd-Pr-Sn	Nd-Pr-Sr	Nd-Pr-Ta	Nd-Pr-Tb	Nd-Pr-Ti
Nd-Pr-V	Nd-Pr-W	Nd-Pr-Y	Nd-Pr-Yb	Nd-Pr-Zn	Nd-Pr-Zr	Nd-Pt-Ru	Nd-Pt-Sb
Nd-Pt-Sc	Nd-Pt-Si	Nd-Pt-Sm	Nd-Pt-Sn	Nd-Pt-Sr	Nd-Pt-Ta	Nd-Pt-Tb	Nd-Pt-Ti
Nd-Pt-V	Nd-Pt-W	Nd-Pt-Y	Nd-Pt-Yb	Nd-Pt-Zn	Nd-Pt-Zr	Nd-Ru-Sb	Nd-Ru-Sc
Nd-Ru-Si	Nd-Ru-Sm	Nd-Ru-Sn	Nd-Ru-Sr	Nd-Ru-Ta	Nd-Ru-Tb	Nd-Ru-Ti	Nd-Ru-V
Nd-Ru-W	Nd-Ru-Y	Nd-Ru-Yb	Nd-Ru-Zn	Nd-Ru-Zr	Nd-Sb-Sc	Nd-Sb-Si	Nd-Sb-Sm
Nd-Sb-Sn	Nd-Sb-Sr	Nd-Sb-Ta	Nd-Sb-Tb	Nd-Sb-Ti	Nd-Sb-V	Nd-Sb-W	Nd-Sb-Y
Nd-Sb-Yb	Nd-Sb-Zn	Nd-Sb-Zr	Nd-Sc-Si	Nd-Sc-Sm	Nd-Sc-Sn	Nd-Sc-Sr	Nd-Sc-Ta
Nd-Sc-Tb	Nd-Sc-Ti	Nd-Sc-V	Nd-Sc-W	Nd-Sc-Y	Nd-Sc-Yb	Nd-Sc-Zn	Nd-Sc-Zr
Nd-Si-Sm	Nd-Si-Sn	Nd-Si-Sr	Nd-Si-Ta	Nd-Si-Tb	Nd-Si-Ti	Nd-Si-V	Nd-Si-W
Nd-Si-Y	Nd-Si-Yb	Nd-Si-Zn	Nd-Si-Zr	Nd-Sm-Sn	Nd-Sm-Sr	Nd-Sm-Ta	Nd-Sm-Tb
Nd-Sm-Ti	Nd-Sm-V	Nd-Sm-W	Nd-Sm-Y	Nd-Sm-Yb	Nd-Sm-Zn	Nd-Sm-Zr	Nd-Sn-Sr
Nd-Sn-Ta	Nd-Sn-Tb	Nd-Sn-Ti	Nd-Sn-V	Nd-Sn-W	Nd-Sn-Y	Nd-Sn-Yb	Nd-Sn-Zn
Nd-Sn-Zr	Nd-Sr-Ta	Nd-Sr-Tb	Nd-Sr-Ti	Nd-Sr-V	Nd-Sr-W	Nd-Sr-Y	Nd-Sr-Yb
Nd-Sr-Zn	Nd-Sr-Zr	Nd-Ta-Tb	Nd-Ta-Ti	Nd-Ta-V	Nd-Ta-W	Nd-Ta-Y	Nd-Ta-Yb
Nd-Ta-Zn	Nd-Ta-Zr	Nd-Tb-Ti	Nd-Tb-V	Nd-Tb-W	Nd-Tb-Y	Nd-Tb-Yb	Nd-Tb-Zn

Nd-Tb-Zr	Nd-Ti-V	Nd-Ti-W	Nd-Ti-Y	Nd-Ti-Yb	Nd-Ti-Zn	Nd-Ti-Zr	Nd-V-W
Nd-V-Y	Nd-V-Yb	Nd-V-Zn	Nd-V-Zr	Nd-W-Y	Nd-W-Yb	Nd-W-Zn	Nd-W-Zr
Nd-Y-Yb	Nd-Y-Zn	Nd-Y-Zr	Nd-Yb-Zn	Nd-Yb-Zr	Nd-Zn-Zr	Ni-P-Pb	Ni-P-Pr
Ni-P-Sb	Ni-P-Sc	Ni-P-Sm	Ni-P-Sn	Ni-P-Sr	Ni-P-Tb	Ni-P-Y	Ni-P-Yb
Ni-P-Zn	Ni-Pb-Pd	Ni-Pb-Pr	Ni-Pb-Pt	Ni-Pb-Ru	Ni-Pb-Sb	Ni-Pb-Sc	Ni-Pb-Si
Ni-Pb-Sm	Ni-Pb-Sn	Ni-Pb-Sr	Ni-Pb-Ta	Ni-Pb-Tb	Ni-Pb-Ti	Ni-Pb-V	Ni-Pb-W
Ni-Pb-Y	Ni-Pb-Yb	Ni-Pb-Zn	Ni-Pb-Zr	Ni-Pd-Pr	Ni-Pd-Pt	Ni-Pd-Ru	Ni-Pd-Sb
Ni-Pd-Sc	Ni-Pd-Sm	Ni-Pd-Sn	Ni-Pd-Sr	Ni-Pd-Tb	Ni-Pd-V	Ni-Pd-W	Ni-Pd-Y
Ni-Pd-Yb	Ni-Pd-Zn	Ni-Pr-Pt	Ni-Pr-Ru	Ni-Pr-Sb	Ni-Pr-Sc	Ni-Pr-Si	Ni-Pr-Sm
Ni-Pr-Sn	Ni-Pr-Sr	Ni-Pr-Ta	Ni-Pr-Tb	Ni-Pr-Ti	Ni-Pr-V	Ni-Pr-W	Ni-Pr-Y
Ni-Pr-Yb	Ni-Pr-Zn	Ni-Pt-Ru	Ni-Pt-Sb	Ni-Pt-Sc	Ni-Pt-Si	Ni-Pt-Sm	Ni-Pt-Sn
Ni-Pt-Sr	Ni-Pt-Ta	Ni-Pt-Tb	Ni-Pt-Ti	Ni-Pt-V	Ni-Pt-W	Ni-Pt-Y	Ni-Pt-Yb
Ni-Pt-Zn	Ni-Pt-Zr	Ni-Ru-Sb	Ni-Ru-Sc	Ni-Ru-Sm	Ni-Ru-Sn	Ni-Ru-Sr	Ni-Ru-Tb
Ni-Ru-Ti	Ni-Ru-V	Ni-Ru-Y	Ni-Ru-Yb	Ni-Ru-Zn	Ni-Sb-Sc	Ni-Sb-Si	Ni-Sb-Sm
Ni-Sb-Sn	Ni-Sb-Sr	Ni-Sb-Ta	Ni-Sb-Tb	Ni-Sb-Ti	Ni-Sb-V	Ni-Sb-W	Ni-Sb-Y
Ni-Sb-Yb	Ni-Sb-Zn	Ni-Sb-Zr	Ni-Sc-Si	Ni-Sc-Sm	Ni-Sc-Sn	Ni-Sc-Sr	Ni-Sc-Ta
Ni-Sc-Tb	Ni-Sc-Ti	Ni-Sc-V	Ni-Sc-W	Ni-Sc-Y	Ni-Sc-Yb	Ni-Sc-Zn	Ni-Sc-Zr
Ni-Si-Sm	Ni-Si-Sr	Ni-Si-Tb	Ni-Si-Y	Ni-Si-Yb	Ni-Si-Zn	Ni-Sm-Sn	Ni-Sm-Sr
Ni-Sm-Ta	Ni-Sm-Tb	Ni-Sm-Ti	Ni-Sm-V	Ni-Sm-W	Ni-Sm-Y	Ni-Sm-Yb	Ni-Sm-Zn
Ni-Sm-Zr	Ni-Sn-Sr	Ni-Sn-Ta	Ni-Sn-Tb	Ni-Sn-Ti	Ni-Sn-V	Ni-Sn-W	Ni-Sn-Y
Ni-Sn-Yb	Ni-Sn-Zn	Ni-Sr-Ta	Ni-Sr-Tb	Ni-Sr-Ti	Ni-Sr-V	Ni-Sr-W	Ni-Sr-Y
Ni-Sr-Yb	Ni-Sr-Zn	Ni-Sr-Zr	Ni-Ta-Tb	Ni-Ta-V	Ni-Ta-W	Ni-Ta-Y	Ni-Ta-Yb
Ni-Ta-Zn	Ni-Tb-Ti	Ni-Tb-V	Ni-Tb-W	Ni-Tb-Y	Ni-Tb-Yb	Ni-Tb-Zn	Ni-Tb-Zr
Ni-Ti-W	Ni-Ti-Y	Ni-Ti-Yb	Ni-Ti-Zn	Ni-V-W	Ni-V-Y	Ni-V-Yb	Ni-V-Zn
Ni-W-Y	Ni-W-Yb	Ni-W-Zn	Ni-Y-Yb	Ni-Y-Zn	Ni-Y-Zr	Ni-Yb-Zn	Ni-Yb-Zr

Ni-Zn-Zr	P-Pb-Pd	P-Pb-Pr	P-Pb-Pt	P-Pb-Ru	P-Pb-Sb	P-Pb-Sc	P-Pb-Si
P-Pb-Sm	P-Pb-Sn	P-Pb-Sr	P-Pb-Ta	P-Pb-Tb	P-Pb-Ti	P-Pb-V	P-Pb-W
P-Pb-Y	P-Pb-Yb	P-Pb-Zn	P-Pb-Zr	P-Pd-Pr	P-Pd-Pt	P-Pd-Ru	P-Pd-Sb
P-Pd-Sc	P-Pd-Sm	P-Pd-Sn	P-Pd-Sr	P-Pd-Tb	P-Pd-V	P-Pd-W	P-Pd-Y
P-Pd-Yb	P-Pd-Zn	P-Pr-Pt	P-Pr-Ru	P-Pr-Sb	P-Pr-Sc	P-Pr-Si	P-Pr-Sm
P-Pr-Sn	P-Pr-Sr	P-Pr-Ta	P-Pr-Tb	P-Pr-Ti	P-Pr-V	P-Pr-W	P-Pr-Y
P-Pr-Yb	P-Pr-Zn	P-Pr-Zr	P-Pt-Ru	P-Pt-Sb	P-Pt-Sc	P-Pt-Si	P-Pt-Sm
P-Pt-Sn	P-Pt-Sr	P-Pt-Ta	P-Pt-Tb	P-Pt-Ti	P-Pt-V	P-Pt-W	P-Pt-Y
P-Pt-Yb	P-Pt-Zn	P-Pt-Zr	P-Ru-Sb	P-Ru-Sc	P-Ru-Sm	P-Ru-Sn	P-Ru-Sr
P-Ru-Tb	P-Ru-Ti	P-Ru-V	P-Ru-Y	P-Ru-Yb	P-Ru-Zn	P-Sb-Sc	P-Sb-Si
P-Sb-Sm	P-Sb-Sn	P-Sb-Sr	P-Sb-Ta	P-Sb-Tb	P-Sb-Ti	P-Sb-V	P-Sb-W
P-Sb-Y	P-Sb-Yb	P-Sb-Zn	P-Sb-Zr	P-Sc-Si	P-Sc-Sm	P-Sc-Sn	P-Sc-Sr
P-Sc-Ta	P-Sc-Tb	P-Sc-Ti	P-Sc-V	P-Sc-W	P-Sc-Y	P-Sc-Yb	P-Sc-Zn
P-Sc-Zr	P-Si-Sm	P-Si-Sn	P-Si-Sr	P-Si-Tb	P-Si-Y	P-Si-Yb	P-Si-Zn
P-Sm-Sn	P-Sm-Sr	P-Sm-Ta	P-Sm-Tb	P-Sm-Ti	P-Sm-V	P-Sm-W	P-Sm-Y
P-Sm-Yb	P-Sm-Zn	P-Sm-Zr	P-Sn-Sr	P-Sn-Ta	P-Sn-Tb	P-Sn-Ti	P-Sn-V
P-Sn-W	P-Sn-Y	P-Sn-Yb	P-Sn-Zn	P-Sn-Zr	P-Sr-Ta	P-Sr-Tb	P-Sr-Ti
P-Sr-V	P-Sr-W	P-Sr-Y	P-Sr-Yb	P-Sr-Zn	P-Sr-Zr	P-Ta-Tb	P-Ta-V
P-Ta-W	P-Ta-Y	P-Ta-Yb	P-Ta-Zn	P-Tb-Ti	P-Tb-V	P-Tb-W	P-Tb-Y
P-Tb-Yb	P-Tb-Zn	P-Tb-Zr	P-Ti-W	P-Ti-Y	P-Ti-Yb	P-Ti-Zn	P-V-W
P-V-Y	P-V-Yb	P-V-Zn	P-W-Y	P-W-Yb	P-W-Zn	P-Y-Yb	P-Y-Zn
P-Y-Zr	P-Yb-Zn	P-Yb-Zr	P-Zn-Zr	Pb-Pd-Pr	Pb-Pd-Pt	Pb-Pd-Ru	Pb-Pd-Sb
Pb-Pd-Sc	Pb-Pd-Si	Pb-Pd-Sm	Pb-Pd-Sn	Pb-Pd-Sr	Pb-Pd-Ta	Pb-Pd-Tb	Pb-Pd-Ti
Pb-Pd-V	Pb-Pd-W	Pb-Pd-Y	Pb-Pd-Yb	Pb-Pd-Zn	Pb-Pd-Zr	Pb-Pr-Pt	Pb-Pr-Ru
Pb-Pr-Sb	Pb-Pr-Sc	Pb-Pr-Si	Pb-Pr-Sm	Pb-Pr-Sn	Pb-Pr-Sr	Pb-Pr-Ta	Pb-Pr-Tb

Pb-Pr-Ti	Pb-Pr-V	Pb-Pr-W	Pb-Pr-Y	Pb-Pr-Yb	Pb-Pr-Zn	Pb-Pr-Zr	Pb-Pt-Ru
Pb-Pt-Sb	Pb-Pt-Sc	Pb-Pt-Si	Pb-Pt-Sm	Pb-Pt-Sn	Pb-Pt-Sr	Pb-Pt-Ta	Pb-Pt-Tb
Pb-Pt-Ti	Pb-Pt-V	Pb-Pt-W	Pb-Pt-Y	Pb-Pt-Yb	Pb-Pt-Zn	Pb-Pt-Zr	Pb-Ru-Sb
Pb-Ru-Sc	Pb-Ru-Si	Pb-Ru-Sm	Pb-Ru-Sn	Pb-Ru-Sr	Pb-Ru-Ta	Pb-Ru-Tb	Pb-Ru-Ti
Pb-Ru-V	Pb-Ru-W	Pb-Ru-Y	Pb-Ru-Yb	Pb-Ru-Zn	Pb-Ru-Zr	Pb-Sb-Sc	Pb-Sb-Si
Pb-Sb-Sm	Pb-Sb-Sn	Pb-Sb-Sr	Pb-Sb-Ta	Pb-Sb-Tb	Pb-Sb-Ti	Pb-Sb-V	Pb-Sb-W
Pb-Sb-Y	Pb-Sb-Yb	Pb-Sb-Zn	Pb-Sb-Zr	Pb-Sc-Si	Pb-Sc-Sm	Pb-Sc-Sn	Pb-Sc-Sr
Pb-Sc-Ta	Pb-Sc-Tb	Pb-Sc-Ti	Pb-Sc-V	Pb-Sc-W	Pb-Sc-Y	Pb-Sc-Yb	Pb-Sc-Zn
Pb-Sc-Zr	Pb-Si-Sm	Pb-Si-Sn	Pb-Si-Sr	Pb-Si-Ta	Pb-Si-Tb	Pb-Si-Ti	Pb-Si-V
Pb-Si-W	Pb-Si-Y	Pb-Si-Yb	Pb-Si-Zn	Pb-Si-Zr	Pb-Sm-Sn	Pb-Sm-Sr	Pb-Sm-Ta
Pb-Sm-Tb	Pb-Sm-Ti	Pb-Sm-V	Pb-Sm-W	Pb-Sm-Y	Pb-Sm-Yb	Pb-Sm-Zn	Pb-Sm-Zr
Pb-Sn-Sr	Pb-Sn-Ta	Pb-Sn-Tb	Pb-Sn-Ti	Pb-Sn-V	Pb-Sn-W	Pb-Sn-Y	Pb-Sn-Yb
Pb-Sn-Zn	Pb-Sn-Zr	Pb-Sr-Ta	Pb-Sr-Tb	Pb-Sr-Ti	Pb-Sr-V	Pb-Sr-W	Pb-Sr-Y
Pb-Sr-Yb	Pb-Sr-Zn	Pb-Sr-Zr	Pb-Ta-Tb	Pb-Ta-Ti	Pb-Ta-V	Pb-Ta-W	Pb-Ta-Y
Pb-Ta-Yb	Pb-Ta-Zn	Pb-Ta-Zr	Pb-Tb-Ti	Pb-Tb-V	Pb-Tb-W	Pb-Tb-Y	Pb-Tb-Yb
Pb-Tb-Zn	Pb-Tb-Zr	Pb-Ti-V	Pb-Ti-W	Pb-Ti-Y	Pb-Ti-Yb	Pb-Ti-Zn	Pb-Ti-Zr
Pb-V-W	Pb-V-Y	Pb-V-Yb	Pb-V-Zn	Pb-V-Zr	Pb-W-Y	Pb-W-Yb	Pb-W-Zn
Pb-W-Zr	Pb-Y-Yb	Pb-Y-Zn	Pb-Y-Zr	Pb-Yb-Zn	Pb-Yb-Zr	Pb-Zn-Zr	Pd-Pr-Pt
Pd-Pr-Ru	Pd-Pr-Sb	Pd-Pr-Sc	Pd-Pr-Si	Pd-Pr-Sm	Pd-Pr-Sn	Pd-Pr-Sr	Pd-Pr-Ta
Pd-Pr-Tb	Pd-Pr-Ti	Pd-Pr-V	Pd-Pr-W	Pd-Pr-Y	Pd-Pr-Yb	Pd-Pr-Zn	Pd-Pr-Zr
Pd-Pt-Ru	Pd-Pt-Sb	Pd-Pt-Sc	Pd-Pt-Si	Pd-Pt-Sm	Pd-Pt-Sn	Pd-Pt-Sr	Pd-Pt-Ta
Pd-Pt-Tb	Pd-Pt-Ti	Pd-Pt-V	Pd-Pt-W	Pd-Pt-Y	Pd-Pt-Yb	Pd-Pt-Zn	Pd-Pt-Zr
Pd-Ru-Sb	Pd-Ru-Sc	Pd-Ru-Si	Pd-Ru-Sm	Pd-Ru-Sn	Pd-Ru-Sr	Pd-Ru-Ta	Pd-Ru-Tb
Pd-Ru-Ti	Pd-Ru-V	Pd-Ru-W	Pd-Ru-Y	Pd-Ru-Yb	Pd-Ru-Zn	Pd-Ru-Zr	Pd-Sb-Sc
Pd-Sb-Sm	Pd-Sb-Sn	Pd-Sb-Sr	Pd-Sb-Ta	Pd-Sb-Tb	Pd-Sb-Ti	Pd-Sb-V	Pd-Sb-W

Pd-Sb-Y	Pd-Sb-Yb	Pd-Sb-Zn	Pd-Sb-Zr	Pd-Sc-Si	Pd-Sc-Sm	Pd-Sc-Sn	Pd-Sc-Sr
Pd-Sc-Ta	Pd-Sc-Tb	Pd-Sc-Ti	Pd-Sc-V	Pd-Sc-W	Pd-Sc-Y	Pd-Sc-Yb	Pd-Sc-Zn
Pd-Sc-Zr	Pd-Si-Sm	Pd-Si-Sn	Pd-Si-Sr	Pd-Si-Tb	Pd-Si-V	Pd-Si-W	Pd-Si-Y
Pd-Si-Yb	Pd-Si-Zn	Pd-Sm-Sn	Pd-Sm-Sr	Pd-Sm-Ta	Pd-Sm-Tb	Pd-Sm-Ti	Pd-Sm-V
Pd-Sm-W	Pd-Sm-Y	Pd-Sm-Yb	Pd-Sm-Zn	Pd-Sm-Zr	Pd-Sn-Sr	Pd-Sn-Ta	Pd-Sn-Tb
Pd-Sn-Ti	Pd-Sn-V	Pd-Sn-W	Pd-Sn-Y	Pd-Sn-Yb	Pd-Sn-Zn	Pd-Sn-Zr	Pd-Sr-Ta
Pd-Sr-Tb	Pd-Sr-Ti	Pd-Sr-V	Pd-Sr-W	Pd-Sr-Y	Pd-Sr-Yb	Pd-Sr-Zn	Pd-Sr-Zr
Pd-Ta-Tb	Pd-Ta-V	Pd-Ta-W	Pd-Ta-Y	Pd-Ta-Yb	Pd-Ta-Zn	Pd-Tb-Ti	Pd-Tb-V
Pd-Tb-W	Pd-Tb-Y	Pd-Tb-Yb	Pd-Tb-Zn	Pd-Tb-Zr	Pd-Ti-V	Pd-Ti-W	Pd-Ti-Y
Pd-Ti-Yb	Pd-Ti-Zn	Pd-V-W	Pd-V-Y	Pd-V-Yb	Pd-V-Zn	Pd-V-Zr	Pd-W-Y
Pd-W-Yb	Pd-W-Zn	Pd-W-Zr	Pd-Y-Yb	Pd-Y-Zn	Pd-Y-Zr	Pd-Yb-Zn	Pd-Yb-Zr
Pd-Zn-Zr	Pr-Pt-Ru	Pr-Pt-Sb	Pr-Pt-Sc	Pr-Pt-Si	Pr-Pt-Sm	Pr-Pt-Sn	Pr-Pt-Sr
Pr-Pt-Ta	Pr-Pt-Tb	Pr-Pt-Ti	Pr-Pt-V	Pr-Pt-W	Pr-Pt-Y	Pr-Pt-Yb	Pr-Pt-Zn
Pr-Pt-Zr	Pr-Ru-Sb	Pr-Ru-Sc	Pr-Ru-Si	Pr-Ru-Sm	Pr-Ru-Sn	Pr-Ru-Sr	Pr-Ru-Ta
Pr-Ru-Tb	Pr-Ru-Ti	Pr-Ru-V	Pr-Ru-W	Pr-Ru-Y	Pr-Ru-Yb	Pr-Ru-Zn	Pr-Ru-Zr
Pr-Sb-Sc	Pr-Sb-Si	Pr-Sb-Sm	Pr-Sb-Sn	Pr-Sb-Sr	Pr-Sb-Ta	Pr-Sb-Tb	Pr-Sb-Ti
Pr-Sb-V	Pr-Sb-W	Pr-Sb-Y	Pr-Sb-Yb	Pr-Sb-Zn	Pr-Sb-Zr	Pr-Sc-Si	Pr-Sc-Sm
Pr-Sc-Sn	Pr-Sc-Sr	Pr-Sc-Ta	Pr-Sc-Tb	Pr-Sc-Ti	Pr-Sc-V	Pr-Sc-W	Pr-Sc-Y
Pr-Sc-Yb	Pr-Sc-Zn	Pr-Sc-Zr	Pr-Si-Sm	Pr-Si-Sn	Pr-Si-Sr	Pr-Si-Ta	Pr-Si-Tb
Pr-Si-Ti	Pr-Si-V	Pr-Si-W	Pr-Si-Y	Pr-Si-Yb	Pr-Si-Zn	Pr-Si-Zr	Pr-Sm-Sn
Pr-Sm-Sr	Pr-Sm-Ta	Pr-Sm-Tb	Pr-Sm-Ti	Pr-Sm-V	Pr-Sm-W	Pr-Sm-Y	Pr-Sm-Yb
Pr-Sm-Zn	Pr-Sm-Zr	Pr-Sn-Sr	Pr-Sn-Ta	Pr-Sn-Tb	Pr-Sn-Ti	Pr-Sn-V	Pr-Sn-W
Pr-Sn-Y	Pr-Sn-Yb	Pr-Sn-Zn	Pr-Sn-Zr	Pr-Sr-Ta	Pr-Sr-Tb	Pr-Sr-Ti	Pr-Sr-V
Pr-Sr-W	Pr-Sr-Y	Pr-Sr-Yb	Pr-Sr-Zn	Pr-Sr-Zr	Pr-Ta-Tb	Pr-Ta-Ti	Pr-Ta-V
Pr-Ta-W	Pr-Ta-Y	Pr-Ta-Yb	Pr-Ta-Zn	Pr-Ta-Zr	Pr-Tb-Ti	Pr-Tb-V	Pr-Tb-W

Pr-Tb-Y	Pr-Tb-Yb	Pr-Tb-Zn	Pr-Tb-Zr	Pr-Ti-V	Pr-Ti-W	Pr-Ti-Y	Pr-Ti-Yb
Pr-Ti-Zn	Pr-Ti-Zr	Pr-V-W	Pr-V-Y	Pr-V-Yb	Pr-V-Zn	Pr-V-Zr	Pr-W-Y
Pr-W-Yb	Pr-W-Zn	Pr-W-Zr	Pr-Y-Yb	Pr-Y-Zn	Pr-Y-Zr	Pr-Yb-Zn	Pr-Yb-Zr
Pr-Zn-Zr	Pt-Ru-Sb	Pt-Ru-Sc	Pt-Ru-Si	Pt-Ru-Sm	Pt-Ru-Sn	Pt-Ru-Sr	Pt-Ru-Ta
Pt-Ru-Tb	Pt-Ru-Ti	Pt-Ru-V	Pt-Ru-W	Pt-Ru-Y	Pt-Ru-Yb	Pt-Ru-Zn	Pt-Ru-Zr
Pt-Sb-Sc	Pt-Sb-Si	Pt-Sb-Sm	Pt-Sb-Sn	Pt-Sb-Sr	Pt-Sb-Ta	Pt-Sb-Tb	Pt-Sb-Ti
Pt-Sb-V	Pt-Sb-W	Pt-Sb-Y	Pt-Sb-Yb	Pt-Sb-Zn	Pt-Sb-Zr	Pt-Sc-Si	Pt-Sc-Sm
Pt-Sc-Sn	Pt-Sc-Sr	Pt-Sc-Ta	Pt-Sc-Tb	Pt-Sc-Ti	Pt-Sc-V	Pt-Sc-W	Pt-Sc-Y
Pt-Sc-Yb	Pt-Sc-Zn	Pt-Sc-Zr	Pt-Si-Sm	Pt-Si-Sn	Pt-Si-Sr	Pt-Si-Ta	Pt-Si-Tb
Pt-Si-Ti	Pt-Si-V	Pt-Si-W	Pt-Si-Y	Pt-Si-Yb	Pt-Si-Zn	Pt-Si-Zr	Pt-Sm-Sn
Pt-Sm-Sr	Pt-Sm-Ta	Pt-Sm-Tb	Pt-Sm-Ti	Pt-Sm-V	Pt-Sm-W	Pt-Sm-Y	Pt-Sm-Yb
Pt-Sm-Zn	Pt-Sm-Zr	Pt-Sn-Sr	Pt-Sn-Ta	Pt-Sn-Tb	Pt-Sn-Ti	Pt-Sn-V	Pt-Sn-W
Pt-Sn-Y	Pt-Sn-Yb	Pt-Sn-Zn	Pt-Sn-Zr	Pt-Sr-Ta	Pt-Sr-Tb	Pt-Sr-Ti	Pt-Sr-V
Pt-Sr-W	Pt-Sr-Y	Pt-Sr-Yb	Pt-Sr-Zn	Pt-Sr-Zr	Pt-Ta-Tb	Pt-Ta-Ti	Pt-Ta-V
Pt-Ta-W	Pt-Ta-Y	Pt-Ta-Yb	Pt-Ta-Zn	Pt-Ta-Zr	Pt-Tb-Ti	Pt-Tb-V	Pt-Tb-W
Pt-Tb-Y	Pt-Tb-Yb	Pt-Tb-Zn	Pt-Tb-Zr	Pt-Ti-V	Pt-Ti-W	Pt-Ti-Y	Pt-Ti-Yb
Pt-Ti-Zn	Pt-Ti-Zr	Pt-V-W	Pt-V-Y	Pt-V-Yb	Pt-V-Zn	Pt-V-Zr	Pt-W-Y
Pt-W-Yb	Pt-W-Zn	Pt-W-Zr	Pt-Y-Yb	Pt-Y-Zn	Pt-Y-Zr	Pt-Yb-Zn	Pt-Yb-Zr
Pt-Zn-Zr	Ru-Sb-Sc	Ru-Sb-Si	Ru-Sb-Sm	Ru-Sb-Sn	Ru-Sb-Sr	Ru-Sb-Ta	Ru-Sb-Tb
Ru-Sb-Ti	Ru-Sb-V	Ru-Sb-W	Ru-Sb-Y	Ru-Sb-Yb	Ru-Sb-Zn	Ru-Sb-Zr	Ru-Sc-Si
Ru-Sc-Sm	Ru-Sc-Sn	Ru-Sc-Sr	Ru-Sc-Ta	Ru-Sc-Tb	Ru-Sc-Ti	Ru-Sc-V	Ru-Sc-W
Ru-Sc-Y	Ru-Sc-Yb	Ru-Sc-Zn	Ru-Sc-Zr	Ru-Si-Sm	Ru-Si-Sn	Ru-Si-Sr	Ru-Si-Tb
Ru-Si-Ti	Ru-Si-V	Ru-Si-Y	Ru-Si-Yb	Ru-Si-Zn	Ru-Sm-Sn	Ru-Sm-Sr	Ru-Sm-Ta
Ru-Sm-Tb	Ru-Sm-Ti	Ru-Sm-V	Ru-Sm-W	Ru-Sm-Y	Ru-Sm-Yb	Ru-Sm-Zn	Ru-Sm-Zr
Ru-Sn-Sr	Ru-Sn-Ta	Ru-Sn-Tb	Ru-Sn-Ti	Ru-Sn-V	Ru-Sn-W	Ru-Sn-Y	Ru-Sn-Yb

Ru-Sn-Zn	Ru-Sn-Zr	Ru-Sr-Ta	Ru-Sr-Tb	Ru-Sr-Ti	Ru-Sr-V	Ru-Sr-W	Ru-Sr-Y
Ru-Sr-Yb	Ru-Sr-Zn	Ru-Sr-Zr	Ru-Ta-Tb	Ru-Ta-Ti	Ru-Ta-V	Ru-Ta-W	Ru-Ta-Y
Ru-Ta-Yb	Ru-Ta-Zn	Ru-Tb-Ti	Ru-Tb-V	Ru-Tb-W	Ru-Tb-Y	Ru-Tb-Yb	Ru-Tb-Zn
Ru-Tb-Zr	Ru-Ti-V	Ru-Ti-W	Ru-Ti-Y	Ru-Ti-Yb	Ru-Ti-Zn	Ru-Ti-Zr	Ru-V-W
Ru-V-Y	Ru-V-Yb	Ru-V-Zn	Ru-V-Zr	Ru-W-Y	Ru-W-Yb	Ru-W-Zn	Ru-Y-Yb
Ru-Y-Zn	Ru-Y-Zr	Ru-Yb-Zn	Ru-Yb-Zr	Ru-Zn-Zr	Sb-Sc-Si	Sb-Sc-Sm	Sb-Sc-Sn
Sb-Sc-Sr	Sb-Sc-Ta	Sb-Sc-Tb	Sb-Sc-Ti	Sb-Sc-V	Sb-Sc-W	Sb-Sc-Y	Sb-Sc-Yb
Sb-Sc-Zn	Sb-Sc-Zr	Sb-Si-Sm	Sb-Si-Sn	Sb-Si-Sr	Sb-Si-Ta	Sb-Si-Tb	Sb-Si-Ti
Sb-Si-V	Sb-Si-W	Sb-Si-Y	Sb-Si-Yb	Sb-Si-Zn	Sb-Si-Zr	Sb-Sm-Sn	Sb-Sm-Sr
Sb-Sm-Ta	Sb-Sm-Tb	Sb-Sm-Ti	Sb-Sm-V	Sb-Sm-W	Sb-Sm-Y	Sb-Sm-Yb	Sb-Sm-Zn
Sb-Sm-Zr	Sb-Sn-Sr	Sb-Sn-Ta	Sb-Sn-Tb	Sb-Sn-Ti	Sb-Sn-V	Sb-Sn-W	Sb-Sn-Y
Sb-Sn-Yb	Sb-Sn-Zn	Sb-Sn-Zr	Sb-Sr-Ta	Sb-Sr-Tb	Sb-Sr-Ti	Sb-Sr-V	Sb-Sr-W
Sb-Sr-Y	Sb-Sr-Yb	Sb-Sr-Zn	Sb-Sr-Zr	Sb-Ta-Tb	Sb-Ta-Ti	Sb-Ta-V	Sb-Ta-W
Sb-Ta-Y	Sb-Ta-Yb	Sb-Ta-Zn	Sb-Ta-Zr	Sb-Tb-Ti	Sb-Tb-V	Sb-Tb-W	Sb-Tb-Y
Sb-Tb-Yb	Sb-Tb-Zn	Sb-Tb-Zr	Sb-Ti-V	Sb-Ti-W	Sb-Ti-Y	Sb-Ti-Yb	Sb-Ti-Zn
Sb-Ti-Zr	Sb-V-W	Sb-V-Y	Sb-V-Yb	Sb-V-Zn	Sb-V-Zr	Sb-W-Y	Sb-W-Yb
Sb-W-Zn	Sb-W-Zr	Sb-Y-Yb	Sb-Y-Zn	Sb-Y-Zr	Sb-Yb-Zn	Sb-Yb-Zr	Sb-Zn-Zr
Sc-Si-Sm	Sc-Si-Sn	Sc-Si-Sr	Sc-Si-Ta	Sc-Si-Tb	Sc-Si-Ti	Sc-Si-V	Sc-Si-W
Sc-Si-Y	Sc-Si-Yb	Sc-Si-Zn	Sc-Si-Zr	Sc-Sm-Sn	Sc-Sm-Sr	Sc-Sm-Ta	Sc-Sm-Tb
Sc-Sm-Ti	Sc-Sm-V	Sc-Sm-W	Sc-Sm-Y	Sc-Sm-Yb	Sc-Sm-Zn	Sc-Sm-Zr	Sc-Sn-Sr
Sc-Sn-Ta	Sc-Sn-Tb	Sc-Sn-Ti	Sc-Sn-V	Sc-Sn-W	Sc-Sn-Y	Sc-Sn-Yb	Sc-Sn-Zn
Sc-Sn-Zr	Sc-Sr-Ta	Sc-Sr-Tb	Sc-Sr-Ti	Sc-Sr-V	Sc-Sr-W	Sc-Sr-Y	Sc-Sr-Yb
Sc-Sr-Zn	Sc-Sr-Zr	Sc-Ta-Tb	Sc-Ta-Ti	Sc-Ta-V	Sc-Ta-W	Sc-Ta-Y	Sc-Ta-Yb
Sc-Ta-Zn	Sc-Ta-Zr	Sc-Tb-Ti	Sc-Tb-V	Sc-Tb-W	Sc-Tb-Y	Sc-Tb-Yb	Sc-Tb-Zn
Sc-Tb-Zr	Sc-Ti-V	Sc-Ti-W	Sc-Ti-Y	Sc-Ti-Yb	Sc-Ti-Zn	Sc-Ti-Zr	Sc-V-W

Sc-V-Y	Sc-V-Yb	Sc-V-Zn	Sc-V-Zr	Sc-W-Y	Sc-W-Yb	Sc-W-Zn	Sc-W-Zr
Sc-Y-Yb	Sc-Y-Zn	Sc-Y-Zr	Sc-Yb-Zn	Sc-Yb-Zr	Sc-Zn-Zr	Si-Sm-Sn	Si-Sm-Sr
Si-Sm-Ta	Si-Sm-Tb	Si-Sm-Ti	Si-Sm-V	Si-Sm-W	Si-Sm-Y	Si-Sm-Yb	Si-Sm-Zn
Si-Sm-Zr	Si-Sn-Sr	Si-Sn-Ta	Si-Sn-Tb	Si-Sn-Ti	Si-Sn-V	Si-Sn-W	Si-Sn-Y
Si-Sn-Yb	Si-Sn-Zn	Si-Sr-Ta	Si-Sr-Tb	Si-Sr-Ti	Si-Sr-V	Si-Sr-W	Si-Sr-Y
Si-Sr-Yb	Si-Sr-Zn	Si-Sr-Zr	Si-Ta-Tb	Si-Ta-V	Si-Ta-W	Si-Ta-Y	Si-Ta-Yb
Si-Ta-Zn	Si-Tb-Ti	Si-Tb-V	Si-Tb-W	Si-Tb-Y	Si-Tb-Yb	Si-Tb-Zn	Si-Tb-Zr
Si-Ti-W	Si-Ti-Y	Si-Ti-Yb	Si-Ti-Zn	Si-V-W	Si-V-Y	Si-V-Yb	Si-V-Zn
Si-W-Y	Si-W-Yb	Si-W-Zn	Si-Y-Yb	Si-Y-Zn	Si-Y-Zr	Si-Yb-Zn	Si-Yb-Zr
Si-Zn-Zr	Sm-Sn-Sr	Sm-Sn-Ta	Sm-Sn-Tb	Sm-Sn-Ti	Sm-Sn-V	Sm-Sn-W	Sm-Sn-Y
Sm-Sn-Yb	Sm-Sn-Zn	Sm-Sn-Zr	Sm-Sr-Ta	Sm-Sr-Tb	Sm-Sr-Ti	Sm-Sr-V	Sm-Sr-W
Sm-Sr-Y	Sm-Sr-Yb	Sm-Sr-Zn	Sm-Sr-Zr	Sm-Ta-Tb	Sm-Ta-Ti	Sm-Ta-V	Sm-Ta-W
Sm-Ta-Y	Sm-Ta-Yb	Sm-Ta-Zn	Sm-Ta-Zr	Sm-Tb-Ti	Sm-Tb-V	Sm-Tb-W	Sm-Tb-Y
Sm-Tb-Yb	Sm-Tb-Zn	Sm-Tb-Zr	Sm-Ti-V	Sm-Ti-W	Sm-Ti-Y	Sm-Ti-Yb	Sm-Ti-Zn
Sm-Ti-Zr	Sm-V-W	Sm-V-Y	Sm-V-Yb	Sm-V-Zn	Sm-V-Zr	Sm-W-Y	Sm-W-Yb
Sm-W-Zn	Sm-W-Zr	Sm-Y-Yb	Sm-Y-Zn	Sm-Y-Zr	Sm-Yb-Zn	Sm-Yb-Zr	Sm-Zn-Zr
Sn-Sr-Ta	Sn-Sr-Tb	Sn-Sr-Ti	Sn-Sr-V	Sn-Sr-W	Sn-Sr-Y	Sn-Sr-Yb	Sn-Sr-Zn
Sn-Sr-Zr	Sn-Ta-Tb	Sn-Ta-Ti	Sn-Ta-V	Sn-Ta-W	Sn-Ta-Y	Sn-Ta-Yb	Sn-Ta-Zn
Sn-Ta-Zr	Sn-Tb-Ti	Sn-Tb-V	Sn-Tb-W	Sn-Tb-Y	Sn-Tb-Yb	Sn-Tb-Zn	Sn-Tb-Zr
Sn-Ti-V	Sn-Ti-W	Sn-Ti-Y	Sn-Ti-Yb	Sn-Ti-Zn	Sn-Ti-Zr	Sn-V-W	Sn-V-Y
Sn-V-Yb	Sn-V-Zn	Sn-V-Zr	Sn-W-Y	Sn-W-Yb	Sn-W-Zn	Sn-W-Zr	Sn-Y-Yb
Sn-Y-Zn	Sn-Y-Zr	Sn-Yb-Zn	Sn-Yb-Zr	Sn-Zn-Zr	Sr-Ta-Tb	Sr-Ta-Ti	Sr-Ta-V
Sr-Ta-W	Sr-Ta-Y	Sr-Ta-Yb	Sr-Ta-Zn	Sr-Ta-Zr	Sr-Tb-Ti	Sr-Tb-V	Sr-Tb-W
Sr-Tb-Y	Sr-Tb-Yb	Sr-Tb-Zn	Sr-Tb-Zr	Sr-Ti-V	Sr-Ti-W	Sr-Ti-Y	Sr-Ti-Yb
Sr-Ti-Zn	Sr-Ti-Zr	Sr-V-W	Sr-V-Y	Sr-V-Yb	Sr-V-Zn	Sr-V-Zr	Sr-W-Y

Sr-W-Yb	Sr-W-Zn	Sr-W-Zr	Sr-Y-Yb	Sr-Y-Zn	Sr-Y-Zr	Sr-Yb-Zn	Sr-Yb-Zr
Sr-Zn-Zr	Ta-Tb-Ti	Ta-Tb-V	Ta-Tb-W	Ta-Tb-Y	Ta-Tb-Yb	Ta-Tb-Zn	Ta-Tb-Zr
Ta-Ti-V	Ta-Ti-W	Ta-Ti-Y	Ta-Ti-Yb	Ta-Ti-Zn	Ta-V-W	Ta-V-Y	Ta-V-Yb
Ta-V-Zn	Ta-V-Zr	Ta-W-Y	Ta-W-Yb	Ta-W-Zn	Ta-W-Zr	Ta-Y-Yb	Ta-Y-Zn
Ta-Y-Zr	Ta-Yb-Zn	Ta-Yb-Zr	Ta-Zn-Zr	Tb-Ti-V	Tb-Ti-W	Tb-Ti-Y	Tb-Ti-Yb
Tb-Ti-Zn	Tb-Ti-Zr	Tb-V-W	Tb-V-Y	Tb-V-Yb	Tb-V-Zn	Tb-V-Zr	Tb-W-Y
Tb-W-Yb	Tb-W-Zn	Tb-W-Zr	Tb-Y-Yb	Tb-Y-Zn	Tb-Y-Zr	Tb-Yb-Zn	Tb-Yb-Zr
Tb-Zn-Zr	Ti-V-W	Ti-V-Y	Ti-V-Yb	Ti-V-Zn	Ti-W-Y	Ti-W-Yb	Ti-W-Zn
Ti-W-Zr	Ti-Y-Yb	Ti-Y-Zn	Ti-Y-Zr	Ti-Yb-Zn	Ti-Yb-Zr	Ti-Zn-Zr	V-W-Y
V-W-Yb	V-W-Zn	V-W-Zr	V-Y-Yb	V-Y-Zn	V-Y-Zr	V-Yb-Zn	V-Yb-Zr
V-Zn-Zr	W-Y-Yb	W-Y-Zn	W-Y-Zr	W-Yb-Zn	W-Yb-Zr	W-Zn-Zr	Y-Yb-Zn
Y-Yb-Zr	Y-Zn-Zr	Yb-Zn-Zr					

III. Existing unexplored triangles in the binary network

A. Auto (62 triagnles)

Ag-Ca-Cu	Al-Ca-Cu	Al-Co-Ti	Al-Co-Zr	Al-Cu-La	Al-Cu-Ti	Al-Cu-Y	Al-Cu-Zr
Al-Fe-Nd	Al-Fe-Ni	Al-Fe-Zr	Al-La-Ni	Al-Mn-Zr	Al-Mo-Ni	Al-Mo-Zr	Al-Nd-Ni
Al-Ni-Ti	Al-Ni-Y	Al-Ni-Zr	Al-Ti-Zr	Au-Gd-Si	Au-Ge-Hf	Au-Ge-Zr	Au-Hf-Si
Au-Si-Zr	B-Fe-Ni	Be-Ti-Zr	Ca-Cu-Mg	Ca-Mg-Zn	Co-Nb-Zr	Co-Ti-Zr	Cu-Mg-Y
Cu-Mg-Zr	Cu-Ti-Zr	Fe-Hf-Ni	Fe-Hf-Si	Fe-Nd-Ni	Fe-Ni-P	Fe-Ni-Si	Fe-Ni-Zr
Fe-Si-Zr	Ge-Pd-Zr	Ge-Pt-Zr	Hf-Ni-Si	Mg-Ni-Y	Mg-Ni-Zr	Mn-Si-Zr	Mo-Ni-Zr
Nb-Ni-Si	Nb-Ni-Zr	Nb-Si-Zr	Ni-P-Pd	Ni-Pd-Si	Ni-Pd-Zr	Ni-Si-Ti	Ni-Si-Zr
Ni-Ti-Zr	Pd-Si-Zr	Pt-Si-Ti	Pt-Si-Zr	Pt-Ti-Zr	Si-Ti-Zr		

B. Fake (8,374 triangles)

Ag-Al-Au	Ag-Al-B	Ag-Al-Be	Ag-Al-C	Ag-Al-Ca	Ag-Al-Ce	Ag-Al-Co	Ag-Al-Cr

Ag-Al-Cu	Ag-Al-Fe	Ag-Al-Gd	Ag-Al-Ge	Ag-Al-Hf	Ag-Al-La	Ag-Al-Mg	Ag-Al-Mn
Ag-Al-Mo	Ag-Al-Nb	Ag-Al-Nd	Ag-Al-Ni	Ag-Al-P	Ag-Al-Pd	Ag-Al-Pt	Ag-Al-Ru
Ag-Al-Sb	Ag-Al-Sc	Ag-Al-Si	Ag-Al-Sm	Ag-Al-Sn	Ag-Al-Ta	Ag-Al-Tb	Ag-Al-Ti
Ag-Al-Y	Ag-Al-Yb	Ag-Al-Zn	Ag-Al-Zr	Ag-Au-B	Ag-Au-Be	Ag-Au-C	Ag-Au-Ca
Ag-Au-Ce	Ag-Au-Co	Ag-Au-Cr	Ag-Au-Cu	Ag-Au-Fe	Ag-Au-Gd	Ag-Au-Ge	Ag-Au-Hf
Ag-Au-La	Ag-Au-Mg	Ag-Au-Mn	Ag-Au-Mo	Ag-Au-Nb	Ag-Au-Nd	Ag-Au-Ni	Ag-Au-P
Ag-Au-Pd	Ag-Au-Pt	Ag-Au-Ru	Ag-Au-Sb	Ag-Au-Sc	Ag-Au-Si	Ag-Au-Sm	Ag-Au-Sn
Ag-Au-Ta	Ag-Au-Tb	Ag-Au-Ti	Ag-Au-Y	Ag-Au-Yb	Ag-Au-Zn	Ag-Au-Zr	Ag-B-Be
Ag-B-C	Ag-B-Ca	Ag-B-Ce	Ag-B-Co	Ag-B-Cr	Ag-B-Cu	Ag-B-Fe	Ag-B-Gd
Ag-B-Ge	Ag-B-Hf	Ag-B-La	Ag-B-Mg	Ag-B-Mn	Ag-B-Mo	Ag-B-Nb	Ag-B-Nd
Ag-B-Ni	Ag-B-P	Ag-B-Pd	Ag-B-Pt	Ag-B-Ru	Ag-B-Sb	Ag-B-Sc	Ag-B-Si
Ag-B-Sm	Ag-B-Sn	Ag-B-Ta	Ag-B-Tb	Ag-B-Ti	Ag-B-Y	Ag-B-Yb	Ag-B-Zn
Ag-B-Zr	Ag-Be-C	Ag-Be-Ca	Ag-Be-Ce	Ag-Be-Co	Ag-Be-Cr	Ag-Be-Cu	Ag-Be-Fe
Ag-Be-Gd	Ag-Be-Ge	Ag-Be-Hf	Ag-Be-La	Ag-Be-Mg	Ag-Be-Mn	Ag-Be-Mo	Ag-Be-Nb
Ag-Be-Nd	Ag-Be-Ni	Ag-Be-P	Ag-Be-Pd	Ag-Be-Pt	Ag-Be-Ru	Ag-Be-Sb	Ag-Be-Sc
Ag-Be-Si	Ag-Be-Sm	Ag-Be-Sn	Ag-Be-Ta	Ag-Be-Tb	Ag-Be-Ti	Ag-Be-Y	Ag-Be-Yb
Ag-Be-Zn	Ag-Be-Zr	Ag-C-Ca	Ag-C-Ce	Ag-C-Co	Ag-C-Cr	Ag-C-Cu	Ag-C-Fe
Ag-C-Gd	Ag-C-Ge	Ag-C-Hf	Ag-C-La	Ag-C-Mg	Ag-C-Mn	Ag-C-Mo	Ag-C-Nb
Ag-C-Nd	Ag-C-Ni	Ag-C-P	Ag-C-Pd	Ag-C-Pt	Ag-C-Ru	Ag-C-Sb	Ag-C-Sc
Ag-C-Si	Ag-C-Sm	Ag-C-Sn	Ag-C-Ta	Ag-C-Tb	Ag-C-Ti	Ag-C-Y	Ag-C-Yb
Ag-C-Zn	Ag-C-Zr	Ag-Ca-Ce	Ag-Ca-Co	Ag-Ca-Cr	Ag-Ca-Fe	Ag-Ca-Gd	Ag-Ca-Ge
Ag-Ca-Hf	Ag-Ca-La	Ag-Ca-Mg	Ag-Ca-Mn	Ag-Ca-Mo	Ag-Ca-Nb	Ag-Ca-Nd	Ag-Ca-Ni
Ag-Ca-P	Ag-Ca-Pd	Ag-Ca-Pt	Ag-Ca-Ru	Ag-Ca-Sb	Ag-Ca-Sc	Ag-Ca-Si	Ag-Ca-Sm
Ag-Ca-Sn	Ag-Ca-Ta	Ag-Ca-Tb	Ag-Ca-Ti	Ag-Ca-Y	Ag-Ca-Yb	Ag-Ca-Zn	Ag-Ca-Zr
Ag-Ce-Co	Ag-Ce-Cr	Ag-Ce-Cu	Ag-Ce-Fe	Ag-Ce-Gd	Ag-Ce-Ge	Ag-Ce-Hf	Ag-Ce-La

Ag-Ce-Mg	Ag-Ce-Mn	Ag-Ce-Mo	Ag-Ce-Nb	Ag-Ce-Nd	Ag-Ce-Ni	Ag-Ce-P	Ag-Ce-Pd
Ag-Ce-Pt	Ag-Ce-Ru	Ag-Ce-Sb	Ag-Ce-Sc	Ag-Ce-Si	Ag-Ce-Sm	Ag-Ce-Sn	Ag-Ce-Ta
Ag-Ce-Tb	Ag-Ce-Ti	Ag-Ce-Y	Ag-Ce-Yb	Ag-Ce-Zn	Ag-Ce-Zr	Ag-Co-Cr	Ag-Co-Cu
Ag-Co-Fe	Ag-Co-Gd	Ag-Co-Ge	Ag-Co-Hf	Ag-Co-La	Ag-Co-Mg	Ag-Co-Mn	Ag-Co-Mo
Ag-Co-Nb	Ag-Co-Nd	Ag-Co-Ni	Ag-Co-P	Ag-Co-Pd	Ag-Co-Pt	Ag-Co-Ru	Ag-Co-Sb
Ag-Co-Sc	Ag-Co-Si	Ag-Co-Sm	Ag-Co-Sn	Ag-Co-Ta	Ag-Co-Tb	Ag-Co-Ti	Ag-Co-Y
Ag-Co-Yb	Ag-Co-Zn	Ag-Co-Zr	Ag-Cr-Cu	Ag-Cr-Fe	Ag-Cr-Gd	Ag-Cr-Ge	Ag-Cr-Hf
Ag-Cr-La	Ag-Cr-Mg	Ag-Cr-Mn	Ag-Cr-Mo	Ag-Cr-Nb	Ag-Cr-Nd	Ag-Cr-Ni	Ag-Cr-P
Ag-Cr-Pd	Ag-Cr-Pt	Ag-Cr-Ru	Ag-Cr-Sb	Ag-Cr-Sc	Ag-Cr-Si	Ag-Cr-Sm	Ag-Cr-Sn
Ag-Cr-Ta	Ag-Cr-Tb	Ag-Cr-Ti	Ag-Cr-Y	Ag-Cr-Yb	Ag-Cr-Zn	Ag-Cr-Zr	Ag-Cu-Fe
Ag-Cu-Gd	Ag-Cu-Ge	Ag-Cu-Hf	Ag-Cu-La	Ag-Cu-Mg	Ag-Cu-Mn	Ag-Cu-Mo	Ag-Cu-Nb
Ag-Cu-Nd	Ag-Cu-Ni	Ag-Cu-P	Ag-Cu-Pd	Ag-Cu-Pt	Ag-Cu-Ru	Ag-Cu-Sb	Ag-Cu-Sc
Ag-Cu-Si	Ag-Cu-Sm	Ag-Cu-Sn	Ag-Cu-Ta	Ag-Cu-Tb	Ag-Cu-Ti	Ag-Cu-Y	Ag-Cu-Yb
Ag-Cu-Zn	Ag-Cu-Zr	Ag-Fe-Gd	Ag-Fe-Ge	Ag-Fe-Hf	Ag-Fe-La	Ag-Fe-Mg	Ag-Fe-Mn
Ag-Fe-Mo	Ag-Fe-Nb	Ag-Fe-Nd	Ag-Fe-Ni	Ag-Fe-P	Ag-Fe-Pd	Ag-Fe-Pt	Ag-Fe-Ru
Ag-Fe-Sb	Ag-Fe-Sc	Ag-Fe-Si	Ag-Fe-Sm	Ag-Fe-Sn	Ag-Fe-Ta	Ag-Fe-Tb	Ag-Fe-Ti
Ag-Fe-Y	Ag-Fe-Yb	Ag-Fe-Zn	Ag-Fe-Zr	Ag-Gd-Ge	Ag-Gd-Hf	Ag-Gd-La	Ag-Gd-Mg
Ag-Gd-Mn	Ag-Gd-Mo	Ag-Gd-Nb	Ag-Gd-Nd	Ag-Gd-Ni	Ag-Gd-P	Ag-Gd-Pd	Ag-Gd-Pt
Ag-Gd-Ru	Ag-Gd-Sb	Ag-Gd-Sc	Ag-Gd-Si	Ag-Gd-Sm	Ag-Gd-Sn	Ag-Gd-Ta	Ag-Gd-Tb
Ag-Gd-Ti	Ag-Gd-Y	Ag-Gd-Yb	Ag-Gd-Zn	Ag-Gd-Zr	Ag-Ge-Hf	Ag-Ge-La	Ag-Ge-Mg
Ag-Ge-Mn	Ag-Ge-Mo	Ag-Ge-Nb	Ag-Ge-Nd	Ag-Ge-Ni	Ag-Ge-P	Ag-Ge-Pd	Ag-Ge-Pt
Ag-Ge-Ru	Ag-Ge-Sb	Ag-Ge-Sc	Ag-Ge-Si	Ag-Ge-Sm	Ag-Ge-Sn	Ag-Ge-Ta	Ag-Ge-Tb
Ag-Ge-Ti	Ag-Ge-Y	Ag-Ge-Yb	Ag-Ge-Zn	Ag-Ge-Zr	Ag-Hf-La	Ag-Hf-Mg	Ag-Hf-Mn
Ag-Hf-Mo	Ag-Hf-Nb	Ag-Hf-Nd	Ag-Hf-Ni	Ag-Hf-P	Ag-Hf-Pd	Ag-Hf-Pt	Ag-Hf-Ru
Ag-Hf-Sb	Ag-Hf-Sc	Ag-Hf-Si	Ag-Hf-Sm	Ag-Hf-Sn	Ag-Hf-Ta	Ag-Hf-Tb	Ag-Hf-Ti

Ag-Hf-Y	Ag-Hf-Yb	Ag-Hf-Zn	Ag-Hf-Zr	Ag-La-Mg	Ag-La-Mn	Ag-La-Mo	Ag-La-Nb
Ag-La-Nd	Ag-La-Ni	Ag-La-P	Ag-La-Pd	Ag-La-Pt	Ag-La-Ru	Ag-La-Sb	Ag-La-Sc
Ag-La-Si	Ag-La-Sm	Ag-La-Sn	Ag-La-Ta	Ag-La-Tb	Ag-La-Ti	Ag-La-Y	Ag-La-Yb
Ag-La-Zn	Ag-La-Zr	Ag-Mg-Mn	Ag-Mg-Mo	Ag-Mg-Nb	Ag-Mg-Nd	Ag-Mg-Ni	Ag-Mg-P
Ag-Mg-Pd	Ag-Mg-Pt	Ag-Mg-Ru	Ag-Mg-Sb	Ag-Mg-Sc	Ag-Mg-Si	Ag-Mg-Sm	Ag-Mg-Sn
Ag-Mg-Ta	Ag-Mg-Tb	Ag-Mg-Ti	Ag-Mg-Y	Ag-Mg-Yb	Ag-Mg-Zn	Ag-Mg-Zr	Ag-Mn-Mo
Ag-Mn-Nb	Ag-Mn-Nd	Ag-Mn-Ni	Ag-Mn-P	Ag-Mn-Pd	Ag-Mn-Pt	Ag-Mn-Ru	Ag-Mn-Sb
Ag-Mn-Sc	Ag-Mn-Si	Ag-Mn-Sm	Ag-Mn-Sn	Ag-Mn-Ta	Ag-Mn-Tb	Ag-Mn-Ti	Ag-Mn-Y
Ag-Mn-Yb	Ag-Mn-Zn	Ag-Mn-Zr	Ag-Mo-Nb	Ag-Mo-Nd	Ag-Mo-Ni	Ag-Mo-P	Ag-Mo-Pd
Ag-Mo-Pt	Ag-Mo-Ru	Ag-Mo-Sb	Ag-Mo-Sc	Ag-Mo-Si	Ag-Mo-Sm	Ag-Mo-Sn	Ag-Mo-Ta
Ag-Mo-Tb	Ag-Mo-Ti	Ag-Mo-Y	Ag-Mo-Yb	Ag-Mo-Zn	Ag-Mo-Zr	Ag-Nb-Nd	Ag-Nb-Ni
Ag-Nb-P	Ag-Nb-Pd	Ag-Nb-Pt	Ag-Nb-Ru	Ag-Nb-Sb	Ag-Nb-Sc	Ag-Nb-Si	Ag-Nb-Sm
Ag-Nb-Sn	Ag-Nb-Ta	Ag-Nb-Tb	Ag-Nb-Ti	Ag-Nb-Y	Ag-Nb-Yb	Ag-Nb-Zn	Ag-Nb-Zr
Ag-Nd-Ni	Ag-Nd-P	Ag-Nd-Pd	Ag-Nd-Pt	Ag-Nd-Ru	Ag-Nd-Sb	Ag-Nd-Sc	Ag-Nd-Si
Ag-Nd-Sm	Ag-Nd-Sn	Ag-Nd-Ta	Ag-Nd-Tb	Ag-Nd-Ti	Ag-Nd-Y	Ag-Nd-Yb	Ag-Nd-Zn
Ag-Nd-Zr	Ag-Ni-P	Ag-Ni-Pd	Ag-Ni-Pt	Ag-Ni-Ru	Ag-Ni-Sb	Ag-Ni-Sc	Ag-Ni-Si
Ag-Ni-Sm	Ag-Ni-Sn	Ag-Ni-Ta	Ag-Ni-Tb	Ag-Ni-Ti	Ag-Ni-Y	Ag-Ni-Yb	Ag-Ni-Zn
Ag-Ni-Zr	Ag-P-Pd	Ag-P-Pt	Ag-P-Ru	Ag-P-Sb	Ag-P-Sc	Ag-P-Si	Ag-P-Sm
Ag-P-Sn	Ag-P-Ta	Ag-P-Tb	Ag-P-Ti	Ag-P-Y	Ag-P-Yb	Ag-P-Zn	Ag-P-Zr
Ag-Pd-Pt	Ag-Pd-Ru	Ag-Pd-Sb	Ag-Pd-Sc	Ag-Pd-Si	Ag-Pd-Sm	Ag-Pd-Sn	Ag-Pd-Ta
Ag-Pd-Tb	Ag-Pd-Ti	Ag-Pd-Y	Ag-Pd-Yb	Ag-Pd-Zn	Ag-Pd-Zr	Ag-Pt-Ru	Ag-Pt-Sb
Ag-Pt-Sc	Ag-Pt-Si	Ag-Pt-Sm	Ag-Pt-Sn	Ag-Pt-Ta	Ag-Pt-Tb	Ag-Pt-Ti	Ag-Pt-Y
Ag-Pt-Yb	Ag-Pt-Zn	Ag-Pt-Zr	Ag-Ru-Sb	Ag-Ru-Sc	Ag-Ru-Si	Ag-Ru-Sm	Ag-Ru-Sn
Ag-Ru-Ta	Ag-Ru-Tb	Ag-Ru-Ti	Ag-Ru-Y	Ag-Ru-Yb	Ag-Ru-Zn	Ag-Ru-Zr	Ag-Sb-Sc
Ag-Sb-Si	Ag-Sb-Sm	Ag-Sb-Sn	Ag-Sb-Ta	Ag-Sb-Tb	Ag-Sb-Ti	Ag-Sb-Y	Ag-Sb-Yb

Ag-Sb-Zn	Ag-Sb-Zr	Ag-Sc-Si	Ag-Sc-Sm	Ag-Sc-Sn	Ag-Sc-Ta	Ag-Sc-Tb	Ag-Sc-Ti
Ag-Sc-Y	Ag-Sc-Yb	Ag-Sc-Zn	Ag-Sc-Zr	Ag-Si-Sm	Ag-Si-Sn	Ag-Si-Ta	Ag-Si-Tb
Ag-Si-Ti	Ag-Si-Y	Ag-Si-Yb	Ag-Si-Zn	Ag-Si-Zr	Ag-Sm-Sn	Ag-Sm-Ta	Ag-Sm-Tb
Ag-Sm-Ti	Ag-Sm-Y	Ag-Sm-Yb	Ag-Sm-Zn	Ag-Sm-Zr	Ag-Sn-Ta	Ag-Sn-Tb	Ag-Sn-Ti
Ag-Sn-Y	Ag-Sn-Yb	Ag-Sn-Zn	Ag-Sn-Zr	Ag-Ta-Tb	Ag-Ta-Ti	Ag-Ta-Y	Ag-Ta-Yb
Ag-Ta-Zn	Ag-Ta-Zr	Ag-Tb-Ti	Ag-Tb-Y	Ag-Tb-Yb	Ag-Tb-Zn	Ag-Tb-Zr	Ag-Ti-Y
Ag-Ti-Yb	Ag-Ti-Zn	Ag-Ti-Zr	Ag-Y-Yb	Ag-Y-Zn	Ag-Y-Zr	Ag-Yb-Zn	Ag-Yb-Zr
Ag-Zn-Zr	Al-Au-B	Al-Au-Be	Al-Au-C	Al-Au-Ca	Al-Au-Ce	Al-Au-Co	Al-Au-Cr
Al-Au-Cu	Al-Au-Fe	Al-Au-Gd	Al-Au-Ge	Al-Au-Hf	Al-Au-La	Al-Au-Mg	Al-Au-Mn
Al-Au-Mo	Al-Au-Nb	Al-Au-Nd	Al-Au-Ni	Al-Au-P	Al-Au-Pd	Al-Au-Pt	Al-Au-Ru
Al-Au-Sb	Al-Au-Sc	Al-Au-Si	Al-Au-Sm	Al-Au-Sn	Al-Au-Ta	Al-Au-Tb	Al-Au-Ti
Al-Au-Y	Al-Au-Yb	Al-Au-Zn	Al-Au-Zr	Al-B-Be	Al-B-C	Al-B-Ca	Al-B-Ce
Al-B-Co	Al-B-Cr	Al-B-Cu	Al-B-Fe	Al-B-Gd	Al-B-Ge	Al-B-Hf	Al-B-La
Al-B-Mg	Al-B-Mn	Al-B-Mo	Al-B-Nb	Al-B-Nd	Al-B-Ni	Al-B-P	Al-B-Pd
Al-B-Pt	Al-B-Ru	Al-B-Sb	Al-B-Sc	Al-B-Si	Al-B-Sm	Al-B-Sn	Al-B-Ta
Al-B-Tb	Al-B-Ti	Al-B-Y	Al-B-Yb	Al-B-Zn	Al-B-Zr	Al-Be-C	Al-Be-Ca
Al-Be-Ce	Al-Be-Co	Al-Be-Cr	Al-Be-Cu	Al-Be-Fe	Al-Be-Gd	Al-Be-Ge	Al-Be-Hf
Al-Be-La	Al-Be-Mg	Al-Be-Mn	Al-Be-Mo	Al-Be-Nb	Al-Be-Nd	Al-Be-Ni	Al-Be-P
Al-Be-Pd	Al-Be-Pt	Al-Be-Ru	Al-Be-Sb	Al-Be-Sc	Al-Be-Si	Al-Be-Sm	Al-Be-Sn
Al-Be-Ta	Al-Be-Tb	Al-Be-Ti	Al-Be-Y	Al-Be-Yb	Al-Be-Zn	Al-Be-Zr	Al-C-Ca
Al-C-Ce	Al-C-Co	Al-C-Cr	Al-C-Cu	Al-C-Fe	Al-C-Gd	Al-C-Ge	Al-C-Hf
Al-C-La	Al-C-Mg	Al-C-Mn	Al-C-Mo	Al-C-Nb	Al-C-Nd	Al-C-Ni	Al-C-P
Al-C-Pd	Al-C-Pt	Al-C-Ru	Al-C-Sb	Al-C-Sc	Al-C-Si	Al-C-Sm	Al-C-Sn
Al-C-Ta	Al-C-Tb	Al-C-Ti	Al-C-Y	Al-C-Yb	Al-C-Zn	Al-C-Zr	Al-Ca-Ce
Al-Ca-Co	Al-Ca-Cr	Al-Ca-Fe	Al-Ca-Gd	Al-Ca-Ge	Al-Ca-Hf	Al-Ca-La	Al-Ca-Mg

Al-Ca-Mn	Al-Ca-Mo	Al-Ca-Nb	Al-Ca-Nd	Al-Ca-Ni	Al-Ca-P	Al-Ca-Pd	Al-Ca-Pt
Al-Ca-Ru	Al-Ca-Sb	Al-Ca-Sc	Al-Ca-Si	Al-Ca-Sm	Al-Ca-Sn	Al-Ca-Ta	Al-Ca-Tb
Al-Ca-Ti	Al-Ca-Y	Al-Ca-Yb	Al-Ca-Zn	Al-Ca-Zr	Al-Ce-Co	Al-Ce-Cr	Al-Ce-Cu
Al-Ce-Fe	Al-Ce-Gd	Al-Ce-Ge	Al-Ce-Hf	Al-Ce-La	Al-Ce-Mg	Al-Ce-Mn	Al-Ce-Mo
Al-Ce-Nb	Al-Ce-Nd	Al-Ce-Ni	Al-Ce-P	Al-Ce-Pd	Al-Ce-Pt	Al-Ce-Ru	Al-Ce-Sb
Al-Ce-Sc	Al-Ce-Si	Al-Ce-Sm	Al-Ce-Sn	Al-Ce-Ta	Al-Ce-Tb	Al-Ce-Ti	Al-Ce-Y
Al-Ce-Yb	Al-Ce-Zn	Al-Ce-Zr	Al-Co-Cr	Al-Co-Cu	Al-Co-Fe	Al-Co-Gd	Al-Co-Ge
Al-Co-Hf	Al-Co-La	Al-Co-Mg	Al-Co-Mn	Al-Co-Mo	Al-Co-Nb	Al-Co-Nd	Al-Co-Ni
Al-Co-P	Al-Co-Pd	Al-Co-Pt	Al-Co-Ru	Al-Co-Sb	Al-Co-Sc	Al-Co-Si	Al-Co-Sm
Al-Co-Sn	Al-Co-Ta	Al-Co-Tb	Al-Co-Y	Al-Co-Yb	Al-Co-Zn	Al-Cr-Cu	Al-Cr-Fe
Al-Cr-Gd	Al-Cr-Ge	Al-Cr-Hf	Al-Cr-La	Al-Cr-Mg	Al-Cr-Mn	Al-Cr-Mo	Al-Cr-Nb
Al-Cr-Nd	Al-Cr-Ni	Al-Cr-P	Al-Cr-Pd	Al-Cr-Pt	Al-Cr-Ru	Al-Cr-Sb	Al-Cr-Sc
Al-Cr-Si	Al-Cr-Sm	Al-Cr-Sn	Al-Cr-Ta	Al-Cr-Tb	Al-Cr-Ti	Al-Cr-Y	Al-Cr-Yb
Al-Cr-Zn	Al-Cr-Zr	Al-Cu-Fe	Al-Cu-Gd	Al-Cu-Ge	Al-Cu-Hf	Al-Cu-Mg	Al-Cu-Mn
Al-Cu-Mo	Al-Cu-Nb	Al-Cu-Nd	Al-Cu-Ni	Al-Cu-P	Al-Cu-Pd	Al-Cu-Pt	Al-Cu-Ru
Al-Cu-Sb	Al-Cu-Sc	Al-Cu-Si	Al-Cu-Sm	Al-Cu-Sn	Al-Cu-Ta	Al-Cu-Tb	Al-Cu-Yb
Al-Cu-Zn	Al-Fe-Gd	Al-Fe-Ge	Al-Fe-Hf	Al-Fe-La	Al-Fe-Mg	Al-Fe-Mn	Al-Fe-Mo
Al-Fe-Nb	Al-Fe-P	Al-Fe-Pd	Al-Fe-Pt	Al-Fe-Ru	Al-Fe-Sb	Al-Fe-Sc	Al-Fe-Si
Al-Fe-Sm	Al-Fe-Sn	Al-Fe-Ta	Al-Fe-Tb	Al-Fe-Ti	Al-Fe-Y	Al-Fe-Yb	Al-Fe-Zn
Al-Gd-Ge	Al-Gd-Hf	Al-Gd-La	Al-Gd-Mg	Al-Gd-Mn	Al-Gd-Mo	Al-Gd-Nb	Al-Gd-Nd
Al-Gd-Ni	Al-Gd-P	Al-Gd-Pd	Al-Gd-Pt	Al-Gd-Ru	Al-Gd-Sb	Al-Gd-Sc	Al-Gd-Si
Al-Gd-Sm	Al-Gd-Sn	Al-Gd-Ta	Al-Gd-Tb	Al-Gd-Ti	Al-Gd-Y	Al-Gd-Yb	Al-Gd-Zn
Al-Gd-Zr	Al-Ge-Hf	Al-Ge-La	Al-Ge-Mg	Al-Ge-Mn	Al-Ge-Mo	Al-Ge-Nb	Al-Ge-Nd
Al-Ge-Ni	Al-Ge-P	Al-Ge-Pd	Al-Ge-Pt	Al-Ge-Ru	Al-Ge-Sb	Al-Ge-Sc	Al-Ge-Si
Al-Ge-Sm	Al-Ge-Sn	Al-Ge-Ta	Al-Ge-Tb	Al-Ge-Ti	Al-Ge-Y	Al-Ge-Yb	Al-Ge-Zn

Al-Ge-Zr	Al-Hf-La	Al-Hf-Mg	Al-Hf-Mn	Al-Hf-Mo	Al-Hf-Nb	Al-Hf-Nd	Al-Hf-Ni
Al-Hf-P	Al-Hf-Pd	Al-Hf-Pt	Al-Hf-Ru	Al-Hf-Sb	Al-Hf-Sc	Al-Hf-Si	Al-Hf-Sm
Al-Hf-Sn	Al-Hf-Ta	Al-Hf-Tb	Al-Hf-Ti	Al-Hf-Y	Al-Hf-Yb	Al-Hf-Zn	Al-Hf-Zr
Al-La-Mg	Al-La-Mn	Al-La-Mo	Al-La-Nb	Al-La-Nd	Al-La-P	Al-La-Pd	Al-La-Pt
Al-La-Ru	Al-La-Sb	Al-La-Sc	Al-La-Si	Al-La-Sm	Al-La-Sn	Al-La-Ta	Al-La-Tb
Al-La-Ti	Al-La-Y	Al-La-Yb	Al-La-Zn	Al-La-Zr	Al-Mg-Mn	Al-Mg-Mo	Al-Mg-Nb
Al-Mg-Nd	Al-Mg-Ni	Al-Mg-P	Al-Mg-Pd	Al-Mg-Pt	Al-Mg-Ru	Al-Mg-Sb	Al-Mg-Sc
Al-Mg-Si	Al-Mg-Sm	Al-Mg-Sn	Al-Mg-Ta	Al-Mg-Tb	Al-Mg-Ti	Al-Mg-Y	Al-Mg-Yb
Al-Mg-Zn	Al-Mg-Zr	Al-Mn-Mo	Al-Mn-Nb	Al-Mn-Nd	Al-Mn-Ni	Al-Mn-P	Al-Mn-Pd
Al-Mn-Pt	Al-Mn-Ru	Al-Mn-Sb	Al-Mn-Sc	Al-Mn-Si	Al-Mn-Sm	Al-Mn-Sn	Al-Mn-Ta
Al-Mn-Tb	Al-Mn-Ti	Al-Mn-Y	Al-Mn-Yb	Al-Mn-Zn	Al-Mo-Nb	Al-Mo-Nd	Al-Mo-P
Al-Mo-Pd	Al-Mo-Pt	Al-Mo-Ru	Al-Mo-Sb	Al-Mo-Sc	Al-Mo-Si	Al-Mo-Sm	Al-Mo-Sn
Al-Mo-Ta	Al-Mo-Tb	Al-Mo-Ti	Al-Mo-Y	Al-Mo-Yb	Al-Mo-Zn	Al-Nb-Nd	Al-Nb-Ni
Al-Nb-P	Al-Nb-Pd	Al-Nb-Pt	Al-Nb-Ru	Al-Nb-Sb	Al-Nb-Sc	Al-Nb-Si	Al-Nb-Sm
Al-Nb-Sn	Al-Nb-Ta	Al-Nb-Tb	Al-Nb-Ti	Al-Nb-Y	Al-Nb-Yb	Al-Nb-Zn	Al-Nb-Zr
Al-Nd-P	Al-Nd-Pd	Al-Nd-Pt	Al-Nd-Ru	Al-Nd-Sb	Al-Nd-Sc	Al-Nd-Si	Al-Nd-Sm
Al-Nd-Sn	Al-Nd-Ta	Al-Nd-Tb	Al-Nd-Ti	Al-Nd-Y	Al-Nd-Yb	Al-Nd-Zn	Al-Nd-Zr
Al-Ni-P	Al-Ni-Pd	Al-Ni-Pt	Al-Ni-Ru	Al-Ni-Sb	Al-Ni-Sc	Al-Ni-Si	Al-Ni-Sm
Al-Ni-Sn	Al-Ni-Ta	Al-Ni-Tb	Al-Ni-Yb	Al-Ni-Zn	Al-P-Pd	Al-P-Pt	Al-P-Ru
Al-P-Sb	Al-P-Sc	Al-P-Si	Al-P-Sm	Al-P-Sn	Al-P-Ta	Al-P-Tb	Al-P-Ti
Al-P-Y	Al-P-Yb	Al-P-Zn	Al-P-Zr	Al-Pd-Pt	Al-Pd-Ru	Al-Pd-Sb	Al-Pd-Sc
Al-Pd-Si	Al-Pd-Sm	Al-Pd-Sn	Al-Pd-Ta	Al-Pd-Tb	Al-Pd-Ti	Al-Pd-Y	Al-Pd-Yb
Al-Pd-Zn	Al-Pd-Zr	Al-Pt-Ru	Al-Pt-Sb	Al-Pt-Sc	Al-Pt-Si	Al-Pt-Sm	Al-Pt-Sn
Al-Pt-Ta	Al-Pt-Tb	Al-Pt-Ti	Al-Pt-Y	Al-Pt-Yb	Al-Pt-Zn	Al-Pt-Zr	Al-Ru-Sb
Al-Ru-Sc	Al-Ru-Si	Al-Ru-Sm	Al-Ru-Sn	Al-Ru-Ta	Al-Ru-Tb	Al-Ru-Ti	Al-Ru-Y

Al-Ru-Yb	Al-Ru-Zn	Al-Ru-Zr	Al-Sb-Sc	Al-Sb-Si	Al-Sb-Sm	Al-Sb-Sn	Al-Sb-Ta
Al-Sb-Tb	Al-Sb-Ti	Al-Sb-Y	Al-Sb-Yb	Al-Sb-Zn	Al-Sb-Zr	Al-Sc-Si	Al-Sc-Sm
Al-Sc-Sn	Al-Sc-Ta	Al-Sc-Tb	Al-Sc-Ti	Al-Sc-Y	Al-Sc-Yb	Al-Sc-Zn	Al-Sc-Zr
Al-Si-Sm	Al-Si-Sn	Al-Si-Ta	Al-Si-Tb	Al-Si-Ti	Al-Si-Y	Al-Si-Yb	Al-Si-Zn
Al-Si-Zr	Al-Sm-Sn	Al-Sm-Ta	Al-Sm-Tb	Al-Sm-Ti	Al-Sm-Y	Al-Sm-Yb	Al-Sm-Zn
Al-Sm-Zr	Al-Sn-Ta	Al-Sn-Tb	Al-Sn-Ti	Al-Sn-Y	Al-Sn-Yb	Al-Sn-Zn	Al-Sn-Zr
Al-Ta-Tb	Al-Ta-Ti	Al-Ta-Y	Al-Ta-Yb	Al-Ta-Zn	Al-Ta-Zr	Al-Tb-Ti	Al-Tb-Y
Al-Tb-Yb	Al-Tb-Zn	Al-Tb-Zr	Al-Ti-Y	Al-Ti-Yb	Al-Ti-Zn	Al-Y-Yb	Al-Y-Zn
Al-Y-Zr	Al-Yb-Zn	Al-Yb-Zr	Al-Zn-Zr	Au-B-Be	Au-B-C	Au-B-Ca	Au-B-Ce
Au-B-Co	Au-B-Cr	Au-B-Cu	Au-B-Fe	Au-B-Gd	Au-B-Ge	Au-B-Hf	Au-B-La
Au-B-Mg	Au-B-Mn	Au-B-Mo	Au-B-Nb	Au-B-Nd	Au-B-Ni	Au-B-P	Au-B-Pd
Au-B-Pt	Au-B-Ru	Au-B-Sb	Au-B-Sc	Au-B-Si	Au-B-Sm	Au-B-Sn	Au-B-Ta
Au-B-Tb	Au-B-Ti	Au-B-Y	Au-B-Yb	Au-B-Zn	Au-B-Zr	Au-Be-C	Au-Be-Ca
Au-Be-Ce	Au-Be-Co	Au-Be-Cr	Au-Be-Cu	Au-Be-Fe	Au-Be-Gd	Au-Be-Ge	Au-Be-Hf
Au-Be-La	Au-Be-Mg	Au-Be-Mn	Au-Be-Mo	Au-Be-Nb	Au-Be-Nd	Au-Be-Ni	Au-Be-P
Au-Be-Pd	Au-Be-Pt	Au-Be-Ru	Au-Be-Sb	Au-Be-Sc	Au-Be-Si	Au-Be-Sm	Au-Be-Sn
Au-Be-Ta	Au-Be-Tb	Au-Be-Ti	Au-Be-Y	Au-Be-Yb	Au-Be-Zn	Au-Be-Zr	Au-C-Ca
Au-C-Ce	Au-C-Co	Au-C-Cr	Au-C-Cu	Au-C-Fe	Au-C-Gd	Au-C-Ge	Au-C-Hf
Au-C-La	Au-C-Mg	Au-C-Mn	Au-C-Mo	Au-C-Nb	Au-C-Nd	Au-C-Ni	Au-C-P
Au-C-Pd	Au-C-Pt	Au-C-Ru	Au-C-Sb	Au-C-Sc	Au-C-Si	Au-C-Sm	Au-C-Sn
Au-C-Ta	Au-C-Tb	Au-C-Ti	Au-C-Y	Au-C-Yb	Au-C-Zn	Au-C-Zr	Au-Ca-Ce
Au-Ca-Co	Au-Ca-Cr	Au-Ca-Cu	Au-Ca-Fe	Au-Ca-Gd	Au-Ca-Ge	Au-Ca-Hf	Au-Ca-La
Au-Ca-Mg	Au-Ca-Mn	Au-Ca-Mo	Au-Ca-Nb	Au-Ca-Nd	Au-Ca-Ni	Au-Ca-P	Au-Ca-Pd
Au-Ca-Pt	Au-Ca-Ru	Au-Ca-Sb	Au-Ca-Sc	Au-Ca-Si	Au-Ca-Sm	Au-Ca-Sn	Au-Ca-Ta
Au-Ca-Tb	Au-Ca-Ti	Au-Ca-Y	Au-Ca-Yb	Au-Ca-Zn	Au-Ca-Zr	Au-Ce-Co	Au-Ce-Cr

Au-Ce-Cu	Au-Ce-Fe	Au-Ce-Gd	Au-Ce-Ge	Au-Ce-Hf	Au-Ce-La	Au-Ce-Mg	Au-Ce-Mn
Au-Ce-Mo	Au-Ce-Nb	Au-Ce-Nd	Au-Ce-Ni	Au-Ce-P	Au-Ce-Pd	Au-Ce-Pt	Au-Ce-Ru
Au-Ce-Sb	Au-Ce-Sc	Au-Ce-Si	Au-Ce-Sm	Au-Ce-Sn	Au-Ce-Ta	Au-Ce-Tb	Au-Ce-Ti
Au-Ce-Y	Au-Ce-Yb	Au-Ce-Zn	Au-Ce-Zr	Au-Co-Cr	Au-Co-Cu	Au-Co-Fe	Au-Co-Gd
Au-Co-Ge	Au-Co-Hf	Au-Co-La	Au-Co-Mg	Au-Co-Mn	Au-Co-Mo	Au-Co-Nb	Au-Co-Nd
Au-Co-Ni	Au-Co-P	Au-Co-Pd	Au-Co-Pt	Au-Co-Ru	Au-Co-Sb	Au-Co-Sc	Au-Co-Si
Au-Co-Sm	Au-Co-Sn	Au-Co-Ta	Au-Co-Tb	Au-Co-Ti	Au-Co-Y	Au-Co-Yb	Au-Co-Zn
Au-Co-Zr	Au-Cr-Cu	Au-Cr-Fe	Au-Cr-Gd	Au-Cr-Ge	Au-Cr-Hf	Au-Cr-La	Au-Cr-Mg
Au-Cr-Mn	Au-Cr-Mo	Au-Cr-Nb	Au-Cr-Nd	Au-Cr-Ni	Au-Cr-P	Au-Cr-Pd	Au-Cr-Pt
Au-Cr-Ru	Au-Cr-Sb	Au-Cr-Sc	Au-Cr-Si	Au-Cr-Sm	Au-Cr-Sn	Au-Cr-Ta	Au-Cr-Tb
Au-Cr-Ti	Au-Cr-Y	Au-Cr-Yb	Au-Cr-Zn	Au-Cr-Zr	Au-Cu-Fe	Au-Cu-Gd	Au-Cu-Ge
Au-Cu-Hf	Au-Cu-La	Au-Cu-Mg	Au-Cu-Mn	Au-Cu-Mo	Au-Cu-Nb	Au-Cu-Nd	Au-Cu-Ni
Au-Cu-P	Au-Cu-Pd	Au-Cu-Pt	Au-Cu-Ru	Au-Cu-Sb	Au-Cu-Sc	Au-Cu-Si	Au-Cu-Sm
Au-Cu-Sn	Au-Cu-Ta	Au-Cu-Tb	Au-Cu-Ti	Au-Cu-Y	Au-Cu-Yb	Au-Cu-Zn	Au-Cu-Zr
Au-Fe-Gd	Au-Fe-Ge	Au-Fe-Hf	Au-Fe-La	Au-Fe-Mg	Au-Fe-Mn	Au-Fe-Mo	Au-Fe-Nb
Au-Fe-Nd	Au-Fe-Ni	Au-Fe-P	Au-Fe-Pd	Au-Fe-Pt	Au-Fe-Ru	Au-Fe-Sb	Au-Fe-Sc
Au-Fe-Si	Au-Fe-Sm	Au-Fe-Sn	Au-Fe-Ta	Au-Fe-Tb	Au-Fe-Ti	Au-Fe-Y	Au-Fe-Yb
Au-Fe-Zn	Au-Fe-Zr	Au-Gd-Ge	Au-Gd-Hf	Au-Gd-La	Au-Gd-Mg	Au-Gd-Mn	Au-Gd-Mo
Au-Gd-Nb	Au-Gd-Nd	Au-Gd-Ni	Au-Gd-P	Au-Gd-Pd	Au-Gd-Pt	Au-Gd-Ru	Au-Gd-Sb
Au-Gd-Sc	Au-Gd-Sm	Au-Gd-Sn	Au-Gd-Ta	Au-Gd-Tb	Au-Gd-Ti	Au-Gd-Y	Au-Gd-Yb
Au-Gd-Zn	Au-Gd-Zr	Au-Ge-La	Au-Ge-Mg	Au-Ge-Mn	Au-Ge-Mo	Au-Ge-Nb	Au-Ge-Nd
Au-Ge-Ni	Au-Ge-P	Au-Ge-Pd	Au-Ge-Pt	Au-Ge-Ru	Au-Ge-Sb	Au-Ge-Sc	Au-Ge-Si
Au-Ge-Sm	Au-Ge-Sn	Au-Ge-Ta	Au-Ge-Tb	Au-Ge-Ti	Au-Ge-Y	Au-Ge-Yb	Au-Ge-Zn
Au-Hf-La	Au-Hf-Mg	Au-Hf-Mn	Au-Hf-Mo	Au-Hf-Nb	Au-Hf-Nd	Au-Hf-Ni	Au-Hf-P
Au-Hf-Pd	Au-Hf-Pt	Au-Hf-Ru	Au-Hf-Sb	Au-Hf-Sc	Au-Hf-Sm	Au-Hf-Sn	Au-Hf-Ta

Au-Hf-Tb	Au-Hf-Ti	Au-Hf-Y	Au-Hf-Yb	Au-Hf-Zn	Au-Hf-Zr	Au-La-Mg	Au-La-Mn
Au-La-Mo	Au-La-Nb	Au-La-Nd	Au-La-Ni	Au-La-P	Au-La-Pd	Au-La-Pt	Au-La-Ru
Au-La-Sb	Au-La-Sc	Au-La-Si	Au-La-Sm	Au-La-Sn	Au-La-Ta	Au-La-Tb	Au-La-Ti
Au-La-Y	Au-La-Yb	Au-La-Zn	Au-La-Zr	Au-Mg-Mn	Au-Mg-Mo	Au-Mg-Nb	Au-Mg-Nd
Au-Mg-Ni	Au-Mg-P	Au-Mg-Pd	Au-Mg-Pt	Au-Mg-Ru	Au-Mg-Sb	Au-Mg-Sc	Au-Mg-Si
Au-Mg-Sm	Au-Mg-Sn	Au-Mg-Ta	Au-Mg-Tb	Au-Mg-Ti	Au-Mg-Y	Au-Mg-Yb	Au-Mg-Zn
Au-Mg-Zr	Au-Mn-Mo	Au-Mn-Nb	Au-Mn-Nd	Au-Mn-Ni	Au-Mn-P	Au-Mn-Pd	Au-Mn-Pt
Au-Mn-Ru	Au-Mn-Sb	Au-Mn-Sc	Au-Mn-Si	Au-Mn-Sm	Au-Mn-Sn	Au-Mn-Ta	Au-Mn-Tb
Au-Mn-Ti	Au-Mn-Y	Au-Mn-Yb	Au-Mn-Zn	Au-Mn-Zr	Au-Mo-Nb	Au-Mo-Nd	Au-Mo-Ni
Au-Mo-P	Au-Mo-Pd	Au-Mo-Pt	Au-Mo-Ru	Au-Mo-Sb	Au-Mo-Sc	Au-Mo-Si	Au-Mo-Sm
Au-Mo-Sn	Au-Mo-Ta	Au-Mo-Tb	Au-Mo-Ti	Au-Mo-Y	Au-Mo-Yb	Au-Mo-Zn	Au-Mo-Zr
Au-Nb-Nd	Au-Nb-Ni	Au-Nb-P	Au-Nb-Pd	Au-Nb-Pt	Au-Nb-Ru	Au-Nb-Sb	Au-Nb-Sc
Au-Nb-Si	Au-Nb-Sm	Au-Nb-Sn	Au-Nb-Ta	Au-Nb-Tb	Au-Nb-Ti	Au-Nb-Y	Au-Nb-Yb
Au-Nb-Zn	Au-Nb-Zr	Au-Nd-Ni	Au-Nd-P	Au-Nd-Pd	Au-Nd-Pt	Au-Nd-Ru	Au-Nd-Sb
Au-Nd-Sc	Au-Nd-Si	Au-Nd-Sm	Au-Nd-Sn	Au-Nd-Ta	Au-Nd-Tb	Au-Nd-Ti	Au-Nd-Y
Au-Nd-Yb	Au-Nd-Zn	Au-Nd-Zr	Au-Ni-P	Au-Ni-Pd	Au-Ni-Pt	Au-Ni-Ru	Au-Ni-Sb
Au-Ni-Sc	Au-Ni-Si	Au-Ni-Sm	Au-Ni-Sn	Au-Ni-Ta	Au-Ni-Tb	Au-Ni-Ti	Au-Ni-Y
Au-Ni-Yb	Au-Ni-Zn	Au-Ni-Zr	Au-P-Pd	Au-P-Pt	Au-P-Ru	Au-P-Sb	Au-P-Sc
Au-P-Si	Au-P-Sm	Au-P-Sn	Au-P-Ta	Au-P-Tb	Au-P-Ti	Au-P-Y	Au-P-Yb
Au-P-Zn	Au-P-Zr	Au-Pd-Pt	Au-Pd-Ru	Au-Pd-Sb	Au-Pd-Sc	Au-Pd-Si	Au-Pd-Sm
Au-Pd-Sn	Au-Pd-Ta	Au-Pd-Tb	Au-Pd-Ti	Au-Pd-Y	Au-Pd-Yb	Au-Pd-Zn	Au-Pd-Zr
Au-Pt-Ru	Au-Pt-Sb	Au-Pt-Sc	Au-Pt-Si	Au-Pt-Sm	Au-Pt-Sn	Au-Pt-Ta	Au-Pt-Tb
Au-Pt-Ti	Au-Pt-Y	Au-Pt-Yb	Au-Pt-Zn	Au-Pt-Zr	Au-Ru-Sb	Au-Ru-Sc	Au-Ru-Si
Au-Ru-Sm	Au-Ru-Sn	Au-Ru-Ta	Au-Ru-Tb	Au-Ru-Ti	Au-Ru-Y	Au-Ru-Yb	Au-Ru-Zn
Au-Ru-Zr	Au-Sb-Sc	Au-Sb-Si	Au-Sb-Sm	Au-Sb-Sn	Au-Sb-Ta	Au-Sb-Tb	Au-Sb-Ti

Au-Sb-Y	Au-Sb-Yb	Au-Sb-Zn	Au-Sb-Zr	Au-Sc-Si	Au-Sc-Sm	Au-Sc-Sn	Au-Sc-Ta
Au-Sc-Tb	Au-Sc-Ti	Au-Sc-Y	Au-Sc-Yb	Au-Sc-Zn	Au-Sc-Zr	Au-Si-Sm	Au-Si-Sn
Au-Si-Ta	Au-Si-Tb	Au-Si-Ti	Au-Si-Y	Au-Si-Yb	Au-Si-Zn	Au-Sm-Sn	Au-Sm-Ta
Au-Sm-Tb	Au-Sm-Ti	Au-Sm-Y	Au-Sm-Yb	Au-Sm-Zn	Au-Sm-Zr	Au-Sn-Ta	Au-Sn-Tb
Au-Sn-Ti	Au-Sn-Y	Au-Sn-Yb	Au-Sn-Zn	Au-Sn-Zr	Au-Ta-Tb	Au-Ta-Ti	Au-Ta-Y
Au-Ta-Yb	Au-Ta-Zn	Au-Ta-Zr	Au-Tb-Ti	Au-Tb-Y	Au-Tb-Yb	Au-Tb-Zn	Au-Tb-Zr
Au-Ti-Y	Au-Ti-Yb	Au-Ti-Zn	Au-Ti-Zr	Au-Y-Yb	Au-Y-Zn	Au-Y-Zr	Au-Yb-Zn
Au-Yb-Zr	Au-Zn-Zr	B-Be-C	B-Be-Ca	B-Be-Ce	B-Be-Co	B-Be-Cr	B-Be-Cu
B-Be-Fe	B-Be-Gd	B-Be-Ge	B-Be-Hf	B-Be-La	B-Be-Mg	B-Be-Mn	B-Be-Mo
B-Be-Nb	B-Be-Nd	B-Be-Ni	B-Be-P	B-Be-Pd	B-Be-Pt	B-Be-Ru	B-Be-Sb
B-Be-Sc	B-Be-Si	B-Be-Sm	B-Be-Sn	B-Be-Ta	B-Be-Tb	B-Be-Ti	B-Be-Y
B-Be-Yb	B-Be-Zn	B-Be-Zr	B-C-Ca	B-C-Ce	B-C-Co	B-C-Cr	B-C-Cu
B-C-Fe	B-C-Gd	B-C-Ge	B-C-Hf	B-C-La	B-C-Mg	B-C-Mn	B-C-Mo
B-C-Nb	B-C-Nd	B-C-Ni	B-C-P	B-C-Pd	B-C-Pt	B-C-Ru	B-C-Sb
B-C-Sc	B-C-Si	B-C-Sm	B-C-Sn	B-C-Ta	B-C-Tb	B-C-Ti	B-C-Y
B-C-Yb	B-C-Zn	B-C-Zr	B-Ca-Ce	B-Ca-Co	B-Ca-Cr	B-Ca-Cu	B-Ca-Fe
B-Ca-Gd	B-Ca-Ge	B-Ca-Hf	B-Ca-La	B-Ca-Mg	B-Ca-Mn	B-Ca-Mo	B-Ca-Nb
B-Ca-Nd	B-Ca-Ni	B-Ca-P	B-Ca-Pd	B-Ca-Pt	B-Ca-Ru	B-Ca-Sb	B-Ca-Sc
B-Ca-Si	B-Ca-Sm	B-Ca-Sn	B-Ca-Ta	B-Ca-Tb	B-Ca-Ti	B-Ca-Y	B-Ca-Yb
B-Ca-Zn	B-Ca-Zr	B-Ce-Co	B-Ce-Cr	B-Ce-Cu	B-Ce-Fe	B-Ce-Gd	B-Ce-Ge
B-Ce-Hf	B-Ce-La	B-Ce-Mg	B-Ce-Mn	B-Ce-Mo	B-Ce-Nb	B-Ce-Nd	B-Ce-Ni
B-Ce-P	B-Ce-Pd	B-Ce-Pt	B-Ce-Ru	B-Ce-Sb	B-Ce-Sc	B-Ce-Si	B-Ce-Sm
B-Ce-Sn	B-Ce-Ta	B-Ce-Tb	B-Ce-Ti	B-Ce-Y	B-Ce-Yb	B-Ce-Zn	B-Ce-Zr
B-Co-Cr	B-Co-Cu	B-Co-Fe	B-Co-Gd	B-Co-Ge	B-Co-Hf	B-Co-La	B-Co-Mg
B-Co-Mn	B-Co-Mo	B-Co-Nb	B-Co-Nd	B-Co-Ni	B-Co-P	B-Co-Pd	B-Co-Pt

B-Co-Ru	B-Co-Sb	B-Co-Sc	B-Co-Si	B-Co-Sm	B-Co-Sn	B-Co-Ta	B-Co-Tb
B-Co-Ti	B-Co-Y	B-Co-Yb	B-Co-Zn	B-Co-Zr	B-Cr-Cu	B-Cr-Fe	B-Cr-Gd
B-Cr-Ge	B-Cr-Hf	B-Cr-La	B-Cr-Mg	B-Cr-Mn	B-Cr-Mo	B-Cr-Nb	B-Cr-Nd
B-Cr-Ni	B-Cr-P	B-Cr-Pd	B-Cr-Pt	B-Cr-Ru	B-Cr-Sb	B-Cr-Sc	B-Cr-Si
B-Cr-Sm	B-Cr-Sn	B-Cr-Ta	B-Cr-Tb	B-Cr-Ti	B-Cr-Y	B-Cr-Yb	B-Cr-Zn
B-Cr-Zr	B-Cu-Fe	B-Cu-Gd	B-Cu-Ge	B-Cu-Hf	B-Cu-La	B-Cu-Mg	B-Cu-Mn
B-Cu-Mo	B-Cu-Nb	B-Cu-Nd	B-Cu-Ni	B-Cu-P	B-Cu-Pd	B-Cu-Pt	B-Cu-Ru
B-Cu-Sb	B-Cu-Sc	B-Cu-Si	B-Cu-Sm	B-Cu-Sn	B-Cu-Ta	B-Cu-Tb	B-Cu-Ti
B-Cu-Y	B-Cu-Yb	B-Cu-Zn	B-Cu-Zr	B-Fe-Gd	B-Fe-Ge	B-Fe-Hf	B-Fe-La
B-Fe-Mg	B-Fe-Mn	B-Fe-Mo	B-Fe-Nb	B-Fe-Nd	B-Fe-P	B-Fe-Pd	B-Fe-Pt
B-Fe-Ru	B-Fe-Sb	B-Fe-Sc	B-Fe-Si	B-Fe-Sm	B-Fe-Sn	B-Fe-Ta	B-Fe-Tb
B-Fe-Ti	B-Fe-Y	B-Fe-Yb	B-Fe-Zn	B-Fe-Zr	B-Gd-Ge	B-Gd-Hf	B-Gd-La
B-Gd-Mg	B-Gd-Mn	B-Gd-Mo	B-Gd-Nb	B-Gd-Nd	B-Gd-Ni	B-Gd-P	B-Gd-Pd
B-Gd-Pt	B-Gd-Ru	B-Gd-Sb	B-Gd-Sc	B-Gd-Si	B-Gd-Sm	B-Gd-Sn	B-Gd-Ta
B-Gd-Tb	B-Gd-Ti	B-Gd-Y	B-Gd-Yb	B-Gd-Zn	B-Gd-Zr	B-Ge-Hf	B-Ge-La
B-Ge-Mg	B-Ge-Mn	B-Ge-Mo	B-Ge-Nb	B-Ge-Nd	B-Ge-Ni	B-Ge-P	B-Ge-Pd
B-Ge-Pt	B-Ge-Ru	B-Ge-Sb	B-Ge-Sc	B-Ge-Si	B-Ge-Sm	B-Ge-Sn	B-Ge-Ta
B-Ge-Tb	B-Ge-Ti	B-Ge-Y	B-Ge-Yb	B-Ge-Zn	B-Ge-Zr	B-Hf-La	B-Hf-Mg
B-Hf-Mn	B-Hf-Mo	B-Hf-Nb	B-Hf-Nd	B-Hf-Ni	B-Hf-P	B-Hf-Pd	B-Hf-Pt
B-Hf-Ru	B-Hf-Sb	B-Hf-Sc	B-Hf-Si	B-Hf-Sm	B-Hf-Sn	B-Hf-Ta	B-Hf-Tb
B-Hf-Ti	B-Hf-Y	B-Hf-Yb	B-Hf-Zn	B-Hf-Zr	B-La-Mg	B-La-Mn	B-La-Mo
B-La-Nb	B-La-Nd	B-La-Ni	B-La-P	B-La-Pd	B-La-Pt	B-La-Ru	B-La-Sb
B-La-Sc	B-La-Si	B-La-Sm	B-La-Sn	B-La-Ta	B-La-Tb	B-La-Ti	B-La-Y
B-La-Yb	B-La-Zn	B-La-Zr	B-Mg-Mn	B-Mg-Mo	B-Mg-Nb	B-Mg-Nd	B-Mg-Ni
B-Mg-P	B-Mg-Pd	B-Mg-Pt	B-Mg-Ru	B-Mg-Sb	B-Mg-Sc	B-Mg-Si	B-Mg-Sm

B-Mg-Sn	B-Mg-Ta	B-Mg-Tb	B-Mg-Ti	B-Mg-Y	B-Mg-Yb	B-Mg-Zn	B-Mg-Zr
B-Mn-Mo	B-Mn-Nb	B-Mn-Nd	B-Mn-Ni	B-Mn-P	B-Mn-Pd	B-Mn-Pt	B-Mn-Ru
B-Mn-Sb	B-Mn-Sc	B-Mn-Si	B-Mn-Sm	B-Mn-Sn	B-Mn-Ta	B-Mn-Tb	B-Mn-Ti
B-Mn-Y	B-Mn-Yb	B-Mn-Zn	B-Mn-Zr	B-Mo-Nb	B-Mo-Nd	B-Mo-Ni	B-Mo-P
B-Mo-Pd	B-Mo-Pt	B-Mo-Ru	B-Mo-Sb	B-Mo-Sc	B-Mo-Si	B-Mo-Sm	B-Mo-Sn
B-Mo-Ta	B-Mo-Tb	B-Mo-Ti	B-Mo-Y	B-Mo-Yb	B-Mo-Zn	B-Mo-Zr	B-Nb-Nd
B-Nb-Ni	B-Nb-P	B-Nb-Pd	B-Nb-Pt	B-Nb-Ru	B-Nb-Sb	B-Nb-Sc	B-Nb-Si
B-Nb-Sm	B-Nb-Sn	B-Nb-Ta	B-Nb-Tb	B-Nb-Ti	B-Nb-Y	B-Nb-Yb	B-Nb-Zn
B-Nb-Zr	B-Nd-Ni	B-Nd-P	B-Nd-Pd	B-Nd-Pt	B-Nd-Ru	B-Nd-Sb	B-Nd-Sc
B-Nd-Si	B-Nd-Sm	B-Nd-Sn	B-Nd-Ta	B-Nd-Tb	B-Nd-Ti	B-Nd-Y	B-Nd-Yb
B-Nd-Zn	B-Nd-Zr	B-Ni-P	B-Ni-Pd	B-Ni-Pt	B-Ni-Ru	B-Ni-Sb	B-Ni-Sc
B-Ni-Si	B-Ni-Sm	B-Ni-Sn	B-Ni-Ta	B-Ni-Tb	B-Ni-Ti	B-Ni-Y	B-Ni-Yb
B-Ni-Zn	B-Ni-Zr	B-P-Pd	B-P-Pt	B-P-Ru	B-P-Sb	B-P-Sc	B-P-Si
B-P-Sm	B-P-Sn	B-P-Ta	B-P-Tb	B-P-Ti	B-P-Y	B-P-Yb	B-P-Zn
B-P-Zr	B-Pd-Pt	B-Pd-Ru	B-Pd-Sb	B-Pd-Sc	B-Pd-Si	B-Pd-Sm	B-Pd-Sn
B-Pd-Ta	B-Pd-Tb	B-Pd-Ti	B-Pd-Y	B-Pd-Yb	B-Pd-Zn	B-Pd-Zr	B-Pt-Ru
B-Pt-Sb	B-Pt-Sc	B-Pt-Si	B-Pt-Sm	B-Pt-Sn	B-Pt-Ta	B-Pt-Tb	B-Pt-Ti
B-Pt-Y	B-Pt-Yb	B-Pt-Zn	B-Pt-Zr	B-Ru-Sb	B-Ru-Sc	B-Ru-Si	B-Ru-Sm
B-Ru-Sn	B-Ru-Ta	B-Ru-Tb	B-Ru-Ti	B-Ru-Y	B-Ru-Yb	B-Ru-Zn	B-Ru-Zr
B-Sb-Sc	B-Sb-Si	B-Sb-Sm	B-Sb-Sn	B-Sb-Ta	B-Sb-Tb	B-Sb-Ti	B-Sb-Y
B-Sb-Yb	B-Sb-Zn	B-Sb-Zr	B-Sc-Si	B-Sc-Sm	B-Sc-Sn	B-Sc-Ta	B-Sc-Tb
B-Sc-Ti	B-Sc-Y	B-Sc-Yb	B-Sc-Zn	B-Sc-Zr	B-Si-Sm	B-Si-Sn	B-Si-Ta
B-Si-Tb	B-Si-Ti	B-Si-Y	B-Si-Yb	B-Si-Zn	B-Si-Zr	B-Sm-Sn	B-Sm-Ta
B-Sm-Tb	B-Sm-Ti	B-Sm-Y	B-Sm-Yb	B-Sm-Zn	B-Sm-Zr	B-Sn-Ta	B-Sn-Tb
B-Sn-Ti	B-Sn-Y	B-Sn-Yb	B-Sn-Zn	B-Sn-Zr	B-Ta-Tb	B-Ta-Ti	B-Ta-Y

B-Ta-Yb	B-Ta-Zn	B-Ta-Zr	B-Tb-Ti	B-Tb-Y	B-Tb-Yb	B-Tb-Zn	B-Tb-Zr
B-Ti-Y	B-Ti-Yb	B-Ti-Zn	B-Ti-Zr	B-Y-Yb	B-Y-Zn	B-Y-Zr	B-Yb-Zn
B-Yb-Zr	B-Zn-Zr	Be-C-Ca	Be-C-Ce	Be-C-Co	Be-C-Cr	Be-C-Cu	Be-C-Fe
Be-C-Gd	Be-C-Ge	Be-C-Hf	Be-C-La	Be-C-Mg	Be-C-Mn	Be-C-Mo	Be-C-Nb
Be-C-Nd	Be-C-Ni	Be-C-P	Be-C-Pd	Be-C-Pt	Be-C-Ru	Be-C-Sb	Be-C-Sc
Be-C-Si	Be-C-Sm	Be-C-Sn	Be-C-Ta	Be-C-Tb	Be-C-Ti	Be-C-Y	Be-C-Yb
Be-C-Zn	Be-C-Zr	Be-Ca-Ce	Be-Ca-Co	Be-Ca-Cr	Be-Ca-Cu	Be-Ca-Fe	Be-Ca-Gd
Be-Ca-Ge	Be-Ca-Hf	Be-Ca-La	Be-Ca-Mg	Be-Ca-Mn	Be-Ca-Mo	Be-Ca-Nb	Be-Ca-Nd
Be-Ca-Ni	Be-Ca-P	Be-Ca-Pd	Be-Ca-Pt	Be-Ca-Ru	Be-Ca-Sb	Be-Ca-Sc	Be-Ca-Si
Be-Ca-Sm	Be-Ca-Sn	Be-Ca-Ta	Be-Ca-Tb	Be-Ca-Ti	Be-Ca-Y	Be-Ca-Yb	Be-Ca-Zn
Be-Ca-Zr	Be-Ce-Co	Be-Ce-Cr	Be-Ce-Cu	Be-Ce-Fe	Be-Ce-Gd	Be-Ce-Ge	Be-Ce-Hf
Be-Ce-La	Be-Ce-Mg	Be-Ce-Mn	Be-Ce-Mo	Be-Ce-Nb	Be-Ce-Nd	Be-Ce-Ni	Be-Ce-P
Be-Ce-Pd	Be-Ce-Pt	Be-Ce-Ru	Be-Ce-Sb	Be-Ce-Sc	Be-Ce-Si	Be-Ce-Sm	Be-Ce-Sn
Be-Ce-Ta	Be-Ce-Tb	Be-Ce-Ti	Be-Ce-Y	Be-Ce-Yb	Be-Ce-Zn	Be-Ce-Zr	Be-Co-Cr
Be-Co-Cu	Be-Co-Fe	Be-Co-Gd	Be-Co-Ge	Be-Co-Hf	Be-Co-La	Be-Co-Mg	Be-Co-Mn
Be-Co-Mo	Be-Co-Nb	Be-Co-Nd	Be-Co-Ni	Be-Co-P	Be-Co-Pd	Be-Co-Pt	Be-Co-Ru
Be-Co-Sb	Be-Co-Sc	Be-Co-Si	Be-Co-Sm	Be-Co-Sn	Be-Co-Ta	Be-Co-Tb	Be-Co-Ti
Be-Co-Y	Be-Co-Yb	Be-Co-Zn	Be-Co-Zr	Be-Cr-Cu	Be-Cr-Fe	Be-Cr-Gd	Be-Cr-Ge
Be-Cr-Hf	Be-Cr-La	Be-Cr-Mg	Be-Cr-Mn	Be-Cr-Mo	Be-Cr-Nb	Be-Cr-Nd	Be-Cr-Ni
Be-Cr-P	Be-Cr-Pd	Be-Cr-Pt	Be-Cr-Ru	Be-Cr-Sb	Be-Cr-Sc	Be-Cr-Si	Be-Cr-Sm
Be-Cr-Sn	Be-Cr-Ta	Be-Cr-Tb	Be-Cr-Ti	Be-Cr-Y	Be-Cr-Yb	Be-Cr-Zn	Be-Cr-Zr
Be-Cu-Fe	Be-Cu-Gd	Be-Cu-Ge	Be-Cu-Hf	Be-Cu-La	Be-Cu-Mg	Be-Cu-Mn	Be-Cu-Mo
Be-Cu-Nb	Be-Cu-Nd	Be-Cu-Ni	Be-Cu-P	Be-Cu-Pd	Be-Cu-Pt	Be-Cu-Ru	Be-Cu-Sb
Be-Cu-Sc	Be-Cu-Si	Be-Cu-Sm	Be-Cu-Sn	Be-Cu-Ta	Be-Cu-Tb	Be-Cu-Ti	Be-Cu-Y
Be-Cu-Yb	Be-Cu-Zn	Be-Cu-Zr	Be-Fe-Gd	Be-Fe-Ge	Be-Fe-Hf	Be-Fe-La	Be-Fe-Mg

Be-Fe-Mn	Be-Fe-Mo	Be-Fe-Nb	Be-Fe-Nd	Be-Fe-Ni	Be-Fe-P	Be-Fe-Pd	Be-Fe-Pt
Be-Fe-Ru	Be-Fe-Sb	Be-Fe-Sc	Be-Fe-Si	Be-Fe-Sm	Be-Fe-Sn	Be-Fe-Ta	Be-Fe-Tb
Be-Fe-Ti	Be-Fe-Y	Be-Fe-Yb	Be-Fe-Zn	Be-Fe-Zr	Be-Gd-Ge	Be-Gd-Hf	Be-Gd-La
Be-Gd-Mg	Be-Gd-Mn	Be-Gd-Mo	Be-Gd-Nb	Be-Gd-Nd	Be-Gd-Ni	Be-Gd-P	Be-Gd-Pd
Be-Gd-Pt	Be-Gd-Ru	Be-Gd-Sb	Be-Gd-Sc	Be-Gd-Si	Be-Gd-Sm	Be-Gd-Sn	Be-Gd-Ta
Be-Gd-Tb	Be-Gd-Ti	Be-Gd-Y	Be-Gd-Yb	Be-Gd-Zn	Be-Gd-Zr	Be-Ge-Hf	Be-Ge-La
Be-Ge-Mg	Be-Ge-Mn	Be-Ge-Mo	Be-Ge-Nb	Be-Ge-Nd	Be-Ge-Ni	Be-Ge-P	Be-Ge-Pd
Be-Ge-Pt	Be-Ge-Ru	Be-Ge-Sb	Be-Ge-Sc	Be-Ge-Si	Be-Ge-Sm	Be-Ge-Sn	Be-Ge-Ta
Be-Ge-Tb	Be-Ge-Ti	Be-Ge-Y	Be-Ge-Yb	Be-Ge-Zn	Be-Ge-Zr	Be-Hf-La	Be-Hf-Mg
Be-Hf-Mn	Be-Hf-Mo	Be-Hf-Nb	Be-Hf-Nd	Be-Hf-Ni	Be-Hf-P	Be-Hf-Pd	Be-Hf-Pt
Be-Hf-Ru	Be-Hf-Sb	Be-Hf-Sc	Be-Hf-Si	Be-Hf-Sm	Be-Hf-Sn	Be-Hf-Ta	Be-Hf-Tb
Be-Hf-Ti	Be-Hf-Y	Be-Hf-Yb	Be-Hf-Zn	Be-Hf-Zr	Be-La-Mg	Be-La-Mn	Be-La-Mo
Be-La-Nb	Be-La-Nd	Be-La-Ni	Be-La-P	Be-La-Pd	Be-La-Pt	Be-La-Ru	Be-La-Sb
Be-La-Sc	Be-La-Si	Be-La-Sm	Be-La-Sn	Be-La-Ta	Be-La-Tb	Be-La-Ti	Be-La-Y
Be-La-Yb	Be-La-Zn	Be-La-Zr	Be-Mg-Mn	Be-Mg-Mo	Be-Mg-Nb	Be-Mg-Nd	Be-Mg-Ni
Be-Mg-P	Be-Mg-Pd	Be-Mg-Pt	Be-Mg-Ru	Be-Mg-Sb	Be-Mg-Sc	Be-Mg-Si	Be-Mg-Sm
Be-Mg-Sn	Be-Mg-Ta	Be-Mg-Tb	Be-Mg-Ti	Be-Mg-Y	Be-Mg-Yb	Be-Mg-Zn	Be-Mg-Zr
Be-Mn-Mo	Be-Mn-Nb	Be-Mn-Nd	Be-Mn-Ni	Be-Mn-P	Be-Mn-Pd	Be-Mn-Pt	Be-Mn-Ru
Be-Mn-Sb	Be-Mn-Sc	Be-Mn-Si	Be-Mn-Sm	Be-Mn-Sn	Be-Mn-Ta	Be-Mn-Tb	Be-Mn-Ti
Be-Mn-Y	Be-Mn-Yb	Be-Mn-Zn	Be-Mn-Zr	Be-Mo-Nb	Be-Mo-Nd	Be-Mo-Ni	Be-Mo-P
Be-Mo-Pd	Be-Mo-Pt	Be-Mo-Ru	Be-Mo-Sb	Be-Mo-Sc	Be-Mo-Si	Be-Mo-Sm	Be-Mo-Sn
Be-Mo-Ta	Be-Mo-Tb	Be-Mo-Ti	Be-Mo-Y	Be-Mo-Yb	Be-Mo-Zn	Be-Mo-Zr	Be-Nb-Nd
Be-Nb-Ni	Be-Nb-P	Be-Nb-Pd	Be-Nb-Pt	Be-Nb-Ru	Be-Nb-Sb	Be-Nb-Sc	Be-Nb-Si
Be-Nb-Sm	Be-Nb-Sn	Be-Nb-Ta	Be-Nb-Tb	Be-Nb-Ti	Be-Nb-Y	Be-Nb-Yb	Be-Nb-Zn
Be-Nb-Zr	Be-Nd-Ni	Be-Nd-P	Be-Nd-Pd	Be-Nd-Pt	Be-Nd-Ru	Be-Nd-Sb	Be-Nd-Sc

Be-Nd-Si	Be-Nd-Sm	Be-Nd-Sn	Be-Nd-Ta	Be-Nd-Tb	Be-Nd-Ti	Be-Nd-Y	Be-Nd-Yb
Be-Nd-Zn	Be-Nd-Zr	Be-Ni-P	Be-Ni-Pd	Be-Ni-Pt	Be-Ni-Ru	Be-Ni-Sb	Be-Ni-Sc
Be-Ni-Si	Be-Ni-Sm	Be-Ni-Sn	Be-Ni-Ta	Be-Ni-Tb	Be-Ni-Ti	Be-Ni-Y	Be-Ni-Yb
Be-Ni-Zn	Be-Ni-Zr	Be-P-Pd	Be-P-Pt	Be-P-Ru	Be-P-Sb	Be-P-Sc	Be-P-Si
Be-P-Sm	Be-P-Sn	Be-P-Ta	Be-P-Tb	Be-P-Ti	Be-P-Y	Be-P-Yb	Be-P-Zn
Be-P-Zr	Be-Pd-Pt	Be-Pd-Ru	Be-Pd-Sb	Be-Pd-Sc	Be-Pd-Si	Be-Pd-Sm	Be-Pd-Sn
Be-Pd-Ta	Be-Pd-Tb	Be-Pd-Ti	Be-Pd-Y	Be-Pd-Yb	Be-Pd-Zn	Be-Pd-Zr	Be-Pt-Ru
Be-Pt-Sb	Be-Pt-Sc	Be-Pt-Si	Be-Pt-Sm	Be-Pt-Sn	Be-Pt-Ta	Be-Pt-Tb	Be-Pt-Ti
Be-Pt-Y	Be-Pt-Yb	Be-Pt-Zn	Be-Pt-Zr	Be-Ru-Sb	Be-Ru-Sc	Be-Ru-Si	Be-Ru-Sm
Be-Ru-Sn	Be-Ru-Ta	Be-Ru-Tb	Be-Ru-Ti	Be-Ru-Y	Be-Ru-Yb	Be-Ru-Zn	Be-Ru-Zr
Be-Sb-Sc	Be-Sb-Si	Be-Sb-Sm	Be-Sb-Sn	Be-Sb-Ta	Be-Sb-Tb	Be-Sb-Ti	Be-Sb-Y
Be-Sb-Yb	Be-Sb-Zn	Be-Sb-Zr	Be-Sc-Si	Be-Sc-Sm	Be-Sc-Sn	Be-Sc-Ta	Be-Sc-Tb
Be-Sc-Ti	Be-Sc-Y	Be-Sc-Yb	Be-Sc-Zn	Be-Sc-Zr	Be-Si-Sm	Be-Si-Sn	Be-Si-Ta
Be-Si-Tb	Be-Si-Ti	Be-Si-Y	Be-Si-Yb	Be-Si-Zn	Be-Si-Zr	Be-Sm-Sn	Be-Sm-Ta
Be-Sm-Tb	Be-Sm-Ti	Be-Sm-Y	Be-Sm-Yb	Be-Sm-Zn	Be-Sm-Zr	Be-Sn-Ta	Be-Sn-Tb
Be-Sn-Ti	Be-Sn-Y	Be-Sn-Yb	Be-Sn-Zn	Be-Sn-Zr	Be-Ta-Tb	Be-Ta-Ti	Be-Ta-Y
Be-Ta-Yb	Be-Ta-Zn	Be-Ta-Zr	Be-Tb-Ti	Be-Tb-Y	Be-Tb-Yb	Be-Tb-Zn	Be-Tb-Zr
Be-Ti-Y	Be-Ti-Yb	Be-Ti-Zn	Be-Y-Yb	Be-Y-Zn	Be-Y-Zr	Be-Yb-Zn	Be-Yb-Zr
Be-Zn-Zr	C-Ca-Ce	C-Ca-Co	C-Ca-Cr	C-Ca-Cu	C-Ca-Fe	C-Ca-Gd	C-Ca-Ge
C-Ca-Hf	C-Ca-La	C-Ca-Mg	C-Ca-Mn	C-Ca-Mo	C-Ca-Nb	C-Ca-Nd	C-Ca-Ni
C-Ca-P	C-Ca-Pd	C-Ca-Pt	C-Ca-Ru	C-Ca-Sb	C-Ca-Sc	C-Ca-Si	C-Ca-Sm
C-Ca-Sn	C-Ca-Ta	C-Ca-Tb	C-Ca-Ti	C-Ca-Y	C-Ca-Yb	C-Ca-Zn	C-Ca-Zr
C-Ce-Co	C-Ce-Cr	C-Ce-Cu	C-Ce-Fe	C-Ce-Gd	C-Ce-Ge	C-Ce-Hf	C-Ce-La
C-Ce-Mg	C-Ce-Mn	C-Ce-Mo	C-Ce-Nb	C-Ce-Nd	C-Ce-Ni	C-Ce-P	C-Ce-Pd
C-Ce-Pt	C-Ce-Ru	C-Ce-Sb	C-Ce-Sc	C-Ce-Si	C-Ce-Sm	C-Ce-Sn	C-Ce-Ta

C-Ce-Tb	C-Ce-Ti	C-Ce-Y	C-Ce-Yb	C-Ce-Zn	C-Ce-Zr	C-Co-Cr	C-Co-Cu
C-Co-Fe	C-Co-Gd	C-Co-Ge	C-Co-Hf	C-Co-La	C-Co-Mg	C-Co-Mn	C-Co-Mo
C-Co-Nb	C-Co-Nd	C-Co-Ni	C-Co-P	C-Co-Pd	C-Co-Pt	C-Co-Ru	C-Co-Sb
C-Co-Sc	C-Co-Si	C-Co-Sm	C-Co-Sn	C-Co-Ta	C-Co-Tb	C-Co-Ti	C-Co-Y
C-Co-Yb	C-Co-Zn	C-Co-Zr	C-Cr-Cu	C-Cr-Fe	C-Cr-Gd	C-Cr-Ge	C-Cr-Hf
C-Cr-La	C-Cr-Mg	C-Cr-Mn	C-Cr-Mo	C-Cr-Nb	C-Cr-Nd	C-Cr-Ni	C-Cr-P
C-Cr-Pd	C-Cr-Pt	C-Cr-Ru	C-Cr-Sb	C-Cr-Sc	C-Cr-Si	C-Cr-Sm	C-Cr-Sn
C-Cr-Ta	C-Cr-Tb	C-Cr-Ti	C-Cr-Y	C-Cr-Yb	C-Cr-Zn	C-Cr-Zr	C-Cu-Fe
C-Cu-Gd	C-Cu-Ge	C-Cu-Hf	C-Cu-La	C-Cu-Mg	C-Cu-Mn	C-Cu-Mo	C-Cu-Nb
C-Cu-Nd	C-Cu-Ni	C-Cu-P	C-Cu-Pd	C-Cu-Pt	C-Cu-Ru	C-Cu-Sb	C-Cu-Sc
C-Cu-Si	C-Cu-Sm	C-Cu-Sn	C-Cu-Ta	C-Cu-Tb	C-Cu-Ti	C-Cu-Y	C-Cu-Yb
C-Cu-Zn	C-Cu-Zr	C-Fe-Gd	C-Fe-Ge	C-Fe-Hf	C-Fe-La	C-Fe-Mg	C-Fe-Mn
C-Fe-Mo	C-Fe-Nb	C-Fe-Nd	C-Fe-Ni	C-Fe-P	C-Fe-Pd	C-Fe-Pt	C-Fe-Ru
C-Fe-Sb	C-Fe-Sc	C-Fe-Si	C-Fe-Sm	C-Fe-Sn	C-Fe-Ta	C-Fe-Tb	C-Fe-Ti
C-Fe-Y	C-Fe-Yb	C-Fe-Zn	C-Fe-Zr	C-Gd-Ge	C-Gd-Hf	C-Gd-La	C-Gd-Mg
C-Gd-Mn	C-Gd-Mo	C-Gd-Nb	C-Gd-Nd	C-Gd-Ni	C-Gd-P	C-Gd-Pd	C-Gd-Pt
C-Gd-Ru	C-Gd-Sb	C-Gd-Sc	C-Gd-Si	C-Gd-Sm	C-Gd-Sn	C-Gd-Ta	C-Gd-Tb
C-Gd-Ti	C-Gd-Y	C-Gd-Yb	C-Gd-Zn	C-Gd-Zr	C-Ge-Hf	C-Ge-La	C-Ge-Mg
C-Ge-Mn	C-Ge-Mo	C-Ge-Nb	C-Ge-Nd	C-Ge-Ni	C-Ge-P	C-Ge-Pd	C-Ge-Pt
C-Ge-Ru	C-Ge-Sb	C-Ge-Sc	C-Ge-Si	C-Ge-Sm	C-Ge-Sn	C-Ge-Ta	C-Ge-Tb
C-Ge-Ti	C-Ge-Y	C-Ge-Yb	C-Ge-Zn	C-Ge-Zr	C-Hf-La	C-Hf-Mg	C-Hf-Mn
C-Hf-Mo	C-Hf-Nb	C-Hf-Nd	C-Hf-Ni	C-Hf-P	C-Hf-Pd	C-Hf-Pt	C-Hf-Ru
C-Hf-Sb	C-Hf-Sc	C-Hf-Si	C-Hf-Sm	C-Hf-Sn	C-Hf-Ta	C-Hf-Tb	C-Hf-Ti
C-Hf-Y	C-Hf-Yb	C-Hf-Zn	C-Hf-Zr	C-La-Mg	C-La-Mn	C-La-Mo	C-La-Nb
C-La-Nd	C-La-Ni	C-La-P	C-La-Pd	C-La-Pt	C-La-Ru	C-La-Sb	C-La-Sc

C-La-Si	C-La-Sm	C-La-Sn	C-La-Ta	C-La-Tb	C-La-Ti	C-La-Y	C-La-Yb
C-La-Zn	C-La-Zr	C-Mg-Mn	C-Mg-Mo	C-Mg-Nb	C-Mg-Nd	C-Mg-Ni	C-Mg-P
C-Mg-Pd	C-Mg-Pt	C-Mg-Ru	C-Mg-Sb	C-Mg-Sc	C-Mg-Si	C-Mg-Sm	C-Mg-Sn
C-Mg-Ta	C-Mg-Tb	C-Mg-Ti	C-Mg-Y	C-Mg-Yb	C-Mg-Zn	C-Mg-Zr	C-Mn-Mo
C-Mn-Nb	C-Mn-Nd	C-Mn-Ni	C-Mn-P	C-Mn-Pd	C-Mn-Pt	C-Mn-Ru	C-Mn-Sb
C-Mn-Sc	C-Mn-Si	C-Mn-Sm	C-Mn-Sn	C-Mn-Ta	C-Mn-Tb	C-Mn-Ti	C-Mn-Y
C-Mn-Yb	C-Mn-Zn	C-Mn-Zr	C-Mo-Nb	C-Mo-Nd	C-Mo-Ni	C-Mo-P	C-Mo-Pd
C-Mo-Pt	C-Mo-Ru	C-Mo-Sb	C-Mo-Sc	C-Mo-Si	C-Mo-Sm	C-Mo-Sn	C-Mo-Ta
C-Mo-Tb	C-Mo-Ti	C-Mo-Y	C-Mo-Yb	C-Mo-Zn	C-Mo-Zr	C-Nb-Nd	C-Nb-Ni
C-Nb-P	C-Nb-Pd	C-Nb-Pt	C-Nb-Ru	C-Nb-Sb	C-Nb-Sc	C-Nb-Si	C-Nb-Sm
C-Nb-Sn	C-Nb-Ta	C-Nb-Tb	C-Nb-Ti	C-Nb-Y	C-Nb-Yb	C-Nb-Zn	C-Nb-Zr
C-Nd-Ni	C-Nd-P	C-Nd-Pd	C-Nd-Pt	C-Nd-Ru	C-Nd-Sb	C-Nd-Sc	C-Nd-Si
C-Nd-Sm	C-Nd-Sn	C-Nd-Ta	C-Nd-Tb	C-Nd-Ti	C-Nd-Y	C-Nd-Yb	C-Nd-Zn
C-Nd-Zr	C-Ni-P	C-Ni-Pd	C-Ni-Pt	C-Ni-Ru	C-Ni-Sb	C-Ni-Sc	C-Ni-Si
C-Ni-Sm	C-Ni-Sn	C-Ni-Ta	C-Ni-Tb	C-Ni-Ti	C-Ni-Y	C-Ni-Yb	C-Ni-Zn
C-Ni-Zr	C-P-Pd	C-P-Pt	C-P-Ru	C-P-Sb	C-P-Sc	C-P-Si	C-P-Sm
C-P-Sn	C-P-Ta	C-P-Tb	C-P-Ti	C-P-Y	C-P-Yb	C-P-Zn	C-P-Zr
C-Pd-Pt	C-Pd-Ru	C-Pd-Sb	C-Pd-Sc	C-Pd-Si	C-Pd-Sm	C-Pd-Sn	C-Pd-Ta
C-Pd-Tb	C-Pd-Ti	C-Pd-Y	C-Pd-Yb	C-Pd-Zn	C-Pd-Zr	C-Pt-Ru	C-Pt-Sb
C-Pt-Sc	C-Pt-Si	C-Pt-Sm	C-Pt-Sn	C-Pt-Ta	C-Pt-Tb	C-Pt-Ti	C-Pt-Y
C-Pt-Yb	C-Pt-Zn	C-Pt-Zr	C-Ru-Sb	C-Ru-Sc	C-Ru-Si	C-Ru-Sm	C-Ru-Sn
C-Ru-Ta	C-Ru-Tb	C-Ru-Ti	C-Ru-Y	C-Ru-Yb	C-Ru-Zn	C-Ru-Zr	C-Sb-Sc
C-Sb-Si	C-Sb-Sm	C-Sb-Sn	C-Sb-Ta	C-Sb-Tb	C-Sb-Ti	C-Sb-Y	C-Sb-Yb
C-Sb-Zn	C-Sb-Zr	C-Sc-Si	C-Sc-Sm	C-Sc-Sn	C-Sc-Ta	C-Sc-Tb	C-Sc-Ti
C-Sc-Y	C-Sc-Yb	C-Sc-Zn	C-Sc-Zr	C-Si-Sm	C-Si-Sn	C-Si-Ta	C-Si-Tb

C-Si-Ti	C-Si-Y	C-Si-Yb	C-Si-Zn	C-Si-Zr	C-Sm-Sn	C-Sm-Ta	C-Sm-Tb
C-Sm-Ti	C-Sm-Y	C-Sm-Yb	C-Sm-Zn	C-Sm-Zr	C-Sn-Ta	C-Sn-Tb	C-Sn-Ti
C-Sn-Y	C-Sn-Yb	C-Sn-Zn	C-Sn-Zr	C-Ta-Tb	C-Ta-Ti	C-Ta-Y	C-Ta-Yb
C-Ta-Zn	C-Ta-Zr	C-Tb-Ti	C-Tb-Y	C-Tb-Yb	C-Tb-Zn	C-Tb-Zr	C-Ti-Y
C-Ti-Yb	C-Ti-Zn	C-Ti-Zr	C-Y-Yb	C-Y-Zn	C-Y-Zr	C-Yb-Zn	C-Yb-Zr
C-Zn-Zr	Ca-Ce-Co	Ca-Ce-Cr	Ca-Ce-Cu	Ca-Ce-Fe	Ca-Ce-Gd	Ca-Ce-Ge	Ca-Ce-Hf
Ca-Ce-La	Ca-Ce-Mg	Ca-Ce-Mn	Ca-Ce-Mo	Ca-Ce-Nb	Ca-Ce-Nd	Ca-Ce-Ni	Ca-Ce-P
Ca-Ce-Pd	Ca-Ce-Pt	Ca-Ce-Ru	Ca-Ce-Sb	Ca-Ce-Sc	Ca-Ce-Si	Ca-Ce-Sm	Ca-Ce-Sn
Ca-Ce-Ta	Ca-Ce-Tb	Ca-Ce-Ti	Ca-Ce-Y	Ca-Ce-Yb	Ca-Ce-Zn	Ca-Ce-Zr	Ca-Co-Cr
Ca-Co-Cu	Ca-Co-Fe	Ca-Co-Gd	Ca-Co-Ge	Ca-Co-Hf	Ca-Co-La	Ca-Co-Mg	Ca-Co-Mn
Ca-Co-Mo	Ca-Co-Nb	Ca-Co-Nd	Ca-Co-Ni	Ca-Co-P	Ca-Co-Pd	Ca-Co-Pt	Ca-Co-Ru
Ca-Co-Sb	Ca-Co-Sc	Ca-Co-Si	Ca-Co-Sm	Ca-Co-Sn	Ca-Co-Ta	Ca-Co-Tb	Ca-Co-Ti
Ca-Co-Y	Ca-Co-Yb	Ca-Co-Zn	Ca-Co-Zr	Ca-Cr-Cu	Ca-Cr-Fe	Ca-Cr-Gd	Ca-Cr-Ge
Ca-Cr-Hf	Ca-Cr-La	Ca-Cr-Mg	Ca-Cr-Mn	Ca-Cr-Mo	Ca-Cr-Nb	Ca-Cr-Nd	Ca-Cr-Ni
Ca-Cr-P	Ca-Cr-Pd	Ca-Cr-Pt	Ca-Cr-Ru	Ca-Cr-Sb	Ca-Cr-Sc	Ca-Cr-Si	Ca-Cr-Sm
Ca-Cr-Sn	Ca-Cr-Ta	Ca-Cr-Tb	Ca-Cr-Ti	Ca-Cr-Y	Ca-Cr-Yb	Ca-Cr-Zn	Ca-Cr-Zr
Ca-Cu-Fe	Ca-Cu-Gd	Ca-Cu-Ge	Ca-Cu-Hf	Ca-Cu-La	Ca-Cu-Mn	Ca-Cu-Mo	Ca-Cu-Nb
Ca-Cu-Nd	Ca-Cu-Ni	Ca-Cu-P	Ca-Cu-Pd	Ca-Cu-Pt	Ca-Cu-Ru	Ca-Cu-Sb	Ca-Cu-Sc
Ca-Cu-Si	Ca-Cu-Sm	Ca-Cu-Sn	Ca-Cu-Ta	Ca-Cu-Tb	Ca-Cu-Ti	Ca-Cu-Y	Ca-Cu-Yb
Ca-Cu-Zn	Ca-Cu-Zr	Ca-Fe-Gd	Ca-Fe-Ge	Ca-Fe-Hf	Ca-Fe-La	Ca-Fe-Mg	Ca-Fe-Mn
Ca-Fe-Mo	Ca-Fe-Nb	Ca-Fe-Nd	Ca-Fe-Ni	Ca-Fe-P	Ca-Fe-Pd	Ca-Fe-Pt	Ca-Fe-Ru
Ca-Fe-Sb	Ca-Fe-Sc	Ca-Fe-Si	Ca-Fe-Sm	Ca-Fe-Sn	Ca-Fe-Ta	Ca-Fe-Tb	Ca-Fe-Ti
Ca-Fe-Y	Ca-Fe-Yb	Ca-Fe-Zn	Ca-Fe-Zr	Ca-Gd-Ge	Ca-Gd-Hf	Ca-Gd-La	Ca-Gd-Mg
Ca-Gd-Mn	Ca-Gd-Mo	Ca-Gd-Nb	Ca-Gd-Nd	Ca-Gd-Ni	Ca-Gd-P	Ca-Gd-Pd	Ca-Gd-Pt
Ca-Gd-Ru	Ca-Gd-Sb	Ca-Gd-Sc	Ca-Gd-Si	Ca-Gd-Sm	Ca-Gd-Sn	Ca-Gd-Ta	Ca-Gd-Tb

Ca-Gd-Ti	Ca-Gd-Y	Ca-Gd-Yb	Ca-Gd-Zn	Ca-Gd-Zr	Ca-Ge-Hf	Ca-Ge-La	Ca-Ge-Mg
Ca-Ge-Mn	Ca-Ge-Mo	Ca-Ge-Nb	Ca-Ge-Nd	Ca-Ge-Ni	Ca-Ge-P	Ca-Ge-Pd	Ca-Ge-Pt
Ca-Ge-Ru	Ca-Ge-Sb	Ca-Ge-Sc	Ca-Ge-Si	Ca-Ge-Sm	Ca-Ge-Sn	Ca-Ge-Ta	Ca-Ge-Tb
Ca-Ge-Ti	Ca-Ge-Y	Ca-Ge-Yb	Ca-Ge-Zn	Ca-Ge-Zr	Ca-Hf-La	Ca-Hf-Mg	Ca-Hf-Mn
Ca-Hf-Mo	Ca-Hf-Nb	Ca-Hf-Nd	Ca-Hf-Ni	Ca-Hf-P	Ca-Hf-Pd	Ca-Hf-Pt	Ca-Hf-Ru
Ca-Hf-Sb	Ca-Hf-Sc	Ca-Hf-Si	Ca-Hf-Sm	Ca-Hf-Sn	Ca-Hf-Ta	Ca-Hf-Tb	Ca-Hf-Ti
Ca-Hf-Y	Ca-Hf-Yb	Ca-Hf-Zn	Ca-Hf-Zr	Ca-La-Mg	Ca-La-Mn	Ca-La-Mo	Ca-La-Nb
Ca-La-Nd	Ca-La-Ni	Ca-La-P	Ca-La-Pd	Ca-La-Pt	Ca-La-Ru	Ca-La-Sb	Ca-La-Sc
Ca-La-Si	Ca-La-Sm	Ca-La-Sn	Ca-La-Ta	Ca-La-Tb	Ca-La-Ti	Ca-La-Y	Ca-La-Yb
Ca-La-Zn	Ca-La-Zr	Ca-Mg-Mn	Ca-Mg-Mo	Ca-Mg-Nb	Ca-Mg-Nd	Ca-Mg-Ni	Ca-Mg-P
Ca-Mg-Pd	Ca-Mg-Pt	Ca-Mg-Ru	Ca-Mg-Sb	Ca-Mg-Sc	Ca-Mg-Si	Ca-Mg-Sm	Ca-Mg-Sn
Ca-Mg-Ta	Ca-Mg-Tb	Ca-Mg-Ti	Ca-Mg-Y	Ca-Mg-Yb	Ca-Mg-Zr	Ca-Mn-Mo	Ca-Mn-Nb
Ca-Mn-Nd	Ca-Mn-Ni	Ca-Mn-P	Ca-Mn-Pd	Ca-Mn-Pt	Ca-Mn-Ru	Ca-Mn-Sb	Ca-Mn-Sc
Ca-Mn-Si	Ca-Mn-Sm	Ca-Mn-Sn	Ca-Mn-Ta	Ca-Mn-Tb	Ca-Mn-Ti	Ca-Mn-Y	Ca-Mn-Yb
Ca-Mn-Zn	Ca-Mn-Zr	Ca-Mo-Nb	Ca-Mo-Nd	Ca-Mo-Ni	Ca-Mo-P	Ca-Mo-Pd	Ca-Mo-Pt
Ca-Mo-Ru	Ca-Mo-Sb	Ca-Mo-Sc	Ca-Mo-Si	Ca-Mo-Sm	Ca-Mo-Sn	Ca-Mo-Ta	Ca-Mo-Tb
Ca-Mo-Ti	Ca-Mo-Y	Ca-Mo-Yb	Ca-Mo-Zn	Ca-Mo-Zr	Ca-Nb-Nd	Ca-Nb-Ni	Ca-Nb-P
Ca-Nb-Pd	Ca-Nb-Pt	Ca-Nb-Ru	Ca-Nb-Sb	Ca-Nb-Sc	Ca-Nb-Si	Ca-Nb-Sm	Ca-Nb-Sn
Ca-Nb-Ta	Ca-Nb-Tb	Ca-Nb-Ti	Ca-Nb-Y	Ca-Nb-Yb	Ca-Nb-Zn	Ca-Nb-Zr	Ca-Nd-Ni
Ca-Nd-P	Ca-Nd-Pd	Ca-Nd-Pt	Ca-Nd-Ru	Ca-Nd-Sb	Ca-Nd-Sc	Ca-Nd-Si	Ca-Nd-Sm
Ca-Nd-Sn	Ca-Nd-Ta	Ca-Nd-Tb	Ca-Nd-Ti	Ca-Nd-Y	Ca-Nd-Yb	Ca-Nd-Zn	Ca-Nd-Zr
Ca-Ni-P	Ca-Ni-Pd	Ca-Ni-Pt	Ca-Ni-Ru	Ca-Ni-Sb	Ca-Ni-Sc	Ca-Ni-Si	Ca-Ni-Sm
Ca-Ni-Sn	Ca-Ni-Ta	Ca-Ni-Tb	Ca-Ni-Ti	Ca-Ni-Y	Ca-Ni-Yb	Ca-Ni-Zn	Ca-Ni-Zr
Ca-P-Pd	Ca-P-Pt	Ca-P-Ru	Ca-P-Sb	Ca-P-Sc	Ca-P-Si	Ca-P-Sm	Ca-P-Sn
Ca-P-Ta	Ca-P-Tb	Ca-P-Ti	Ca-P-Y	Ca-P-Yb	Ca-P-Zn	Ca-P-Zr	Ca-Pd-Pt

Ca-Pd-Ru	Ca-Pd-Sb	Ca-Pd-Sc	Ca-Pd-Si	Ca-Pd-Sm	Ca-Pd-Sn	Ca-Pd-Ta	Ca-Pd-Tb
Ca-Pd-Ti	Ca-Pd-Y	Ca-Pd-Yb	Ca-Pd-Zn	Ca-Pd-Zr	Ca-Pt-Ru	Ca-Pt-Sb	Ca-Pt-Sc
Ca-Pt-Si	Ca-Pt-Sm	Ca-Pt-Sn	Ca-Pt-Ta	Ca-Pt-Tb	Ca-Pt-Ti	Ca-Pt-Y	Ca-Pt-Yb
Ca-Pt-Zn	Ca-Pt-Zr	Ca-Ru-Sb	Ca-Ru-Sc	Ca-Ru-Si	Ca-Ru-Sm	Ca-Ru-Sn	Ca-Ru-Ta
Ca-Ru-Tb	Ca-Ru-Ti	Ca-Ru-Y	Ca-Ru-Yb	Ca-Ru-Zn	Ca-Ru-Zr	Ca-Sb-Sc	Ca-Sb-Si
Ca-Sb-Sm	Ca-Sb-Sn	Ca-Sb-Ta	Ca-Sb-Tb	Ca-Sb-Ti	Ca-Sb-Y	Ca-Sb-Yb	Ca-Sb-Zn
Ca-Sb-Zr	Ca-Sc-Si	Ca-Sc-Sm	Ca-Sc-Sn	Ca-Sc-Ta	Ca-Sc-Tb	Ca-Sc-Ti	Ca-Sc-Y
Ca-Sc-Yb	Ca-Sc-Zn	Ca-Sc-Zr	Ca-Si-Sm	Ca-Si-Sn	Ca-Si-Ta	Ca-Si-Tb	Ca-Si-Ti
Ca-Si-Y	Ca-Si-Yb	Ca-Si-Zn	Ca-Si-Zr	Ca-Sm-Sn	Ca-Sm-Ta	Ca-Sm-Tb	Ca-Sm-Ti
Ca-Sm-Y	Ca-Sm-Yb	Ca-Sm-Zn	Ca-Sm-Zr	Ca-Sn-Ta	Ca-Sn-Tb	Ca-Sn-Ti	Ca-Sn-Y
Ca-Sn-Yb	Ca-Sn-Zn	Ca-Sn-Zr	Ca-Ta-Tb	Ca-Ta-Ti	Ca-Ta-Y	Ca-Ta-Yb	Ca-Ta-Zn
Ca-Ta-Zr	Ca-Tb-Ti	Ca-Tb-Y	Ca-Tb-Yb	Ca-Tb-Zn	Ca-Tb-Zr	Ca-Ti-Y	Ca-Ti-Yb
Ca-Ti-Zn	Ca-Ti-Zr	Ca-Y-Yb	Ca-Y-Zn	Ca-Y-Zr	Ca-Yb-Zn	Ca-Yb-Zr	Ca-Zn-Zr
Ce-Co-Cr	Ce-Co-Cu	Ce-Co-Fe	Ce-Co-Gd	Ce-Co-Ge	Ce-Co-Hf	Ce-Co-La	Ce-Co-Mg
Ce-Co-Mn	Ce-Co-Mo	Ce-Co-Nb	Ce-Co-Nd	Ce-Co-Ni	Ce-Co-P	Ce-Co-Pd	Ce-Co-Pt
Ce-Co-Ru	Ce-Co-Sb	Ce-Co-Sc	Ce-Co-Si	Ce-Co-Sm	Ce-Co-Sn	Ce-Co-Ta	Ce-Co-Tb
Ce-Co-Ti	Ce-Co-Y	Ce-Co-Yb	Ce-Co-Zn	Ce-Co-Zr	Ce-Cr-Cu	Ce-Cr-Fe	Ce-Cr-Gd
Ce-Cr-Ge	Ce-Cr-Hf	Ce-Cr-La	Ce-Cr-Mg	Ce-Cr-Mn	Ce-Cr-Mo	Ce-Cr-Nb	Ce-Cr-Nd
Ce-Cr-Ni	Ce-Cr-P	Ce-Cr-Pd	Ce-Cr-Pt	Ce-Cr-Ru	Ce-Cr-Sb	Ce-Cr-Sc	Ce-Cr-Si
Ce-Cr-Sm	Ce-Cr-Sn	Ce-Cr-Ta	Ce-Cr-Tb	Ce-Cr-Ti	Ce-Cr-Y	Ce-Cr-Yb	Ce-Cr-Zn
Ce-Cr-Zr	Ce-Cu-Fe	Ce-Cu-Gd	Ce-Cu-Ge	Ce-Cu-Hf	Ce-Cu-La	Ce-Cu-Mg	Ce-Cu-Mn
Ce-Cu-Mo	Ce-Cu-Nb	Ce-Cu-Nd	Ce-Cu-Ni	Ce-Cu-P	Ce-Cu-Pd	Ce-Cu-Pt	Ce-Cu-Ru
Ce-Cu-Sb	Ce-Cu-Sc	Ce-Cu-Si	Ce-Cu-Sm	Ce-Cu-Sn	Ce-Cu-Ta	Ce-Cu-Tb	Ce-Cu-Ti
Ce-Cu-Y	Ce-Cu-Yb	Ce-Cu-Zn	Ce-Cu-Zr	Ce-Fe-Gd	Ce-Fe-Ge	Ce-Fe-Hf	Ce-Fe-La
Ce-Fe-Mg	Ce-Fe-Mn	Ce-Fe-Mo	Ce-Fe-Nb	Ce-Fe-Nd	Ce-Fe-Ni	Ce-Fe-P	Ce-Fe-Pd

Ce-Fe-Pt	Ce-Fe-Ru	Ce-Fe-Sb	Ce-Fe-Sc	Ce-Fe-Si	Ce-Fe-Sm	Ce-Fe-Sn	Ce-Fe-Ta
Ce-Fe-Tb	Ce-Fe-Ti	Ce-Fe-Y	Ce-Fe-Yb	Ce-Fe-Zn	Ce-Fe-Zr	Ce-Gd-Ge	Ce-Gd-Hf
Ce-Gd-La	Ce-Gd-Mg	Ce-Gd-Mn	Ce-Gd-Mo	Ce-Gd-Nb	Ce-Gd-Nd	Ce-Gd-Ni	Ce-Gd-P
Ce-Gd-Pd	Ce-Gd-Pt	Ce-Gd-Ru	Ce-Gd-Sb	Ce-Gd-Sc	Ce-Gd-Si	Ce-Gd-Sm	Ce-Gd-Sn
Ce-Gd-Ta	Ce-Gd-Tb	Ce-Gd-Ti	Ce-Gd-Y	Ce-Gd-Yb	Ce-Gd-Zn	Ce-Gd-Zr	Ce-Ge-Hf
Ce-Ge-La	Ce-Ge-Mg	Ce-Ge-Mn	Ce-Ge-Mo	Ce-Ge-Nb	Ce-Ge-Nd	Ce-Ge-Ni	Ce-Ge-P
Ce-Ge-Pd	Ce-Ge-Pt	Ce-Ge-Ru	Ce-Ge-Sb	Ce-Ge-Sc	Ce-Ge-Si	Ce-Ge-Sm	Ce-Ge-Sn
Ce-Ge-Ta	Ce-Ge-Tb	Ce-Ge-Ti	Ce-Ge-Y	Ce-Ge-Yb	Ce-Ge-Zn	Ce-Ge-Zr	Ce-Hf-La
Ce-Hf-Mg	Ce-Hf-Mn	Ce-Hf-Mo	Ce-Hf-Nb	Ce-Hf-Nd	Ce-Hf-Ni	Ce-Hf-P	Ce-Hf-Pd
Ce-Hf-Pt	Ce-Hf-Ru	Ce-Hf-Sb	Ce-Hf-Sc	Ce-Hf-Si	Ce-Hf-Sm	Ce-Hf-Sn	Ce-Hf-Ta
Ce-Hf-Tb	Ce-Hf-Ti	Ce-Hf-Y	Ce-Hf-Yb	Ce-Hf-Zn	Ce-Hf-Zr	Ce-La-Mg	Ce-La-Mn
Ce-La-Mo	Ce-La-Nb	Ce-La-Nd	Ce-La-Ni	Ce-La-P	Ce-La-Pd	Ce-La-Pt	Ce-La-Ru
Ce-La-Sb	Ce-La-Sc	Ce-La-Si	Ce-La-Sm	Ce-La-Sn	Ce-La-Ta	Ce-La-Tb	Ce-La-Ti
Ce-La-Y	Ce-La-Yb	Ce-La-Zn	Ce-La-Zr	Ce-Mg-Mn	Ce-Mg-Mo	Ce-Mg-Nb	Ce-Mg-Nd
Ce-Mg-Ni	Ce-Mg-P	Ce-Mg-Pd	Ce-Mg-Pt	Ce-Mg-Ru	Ce-Mg-Sb	Ce-Mg-Sc	Ce-Mg-Si
Ce-Mg-Sm	Ce-Mg-Sn	Ce-Mg-Ta	Ce-Mg-Tb	Ce-Mg-Ti	Ce-Mg-Y	Ce-Mg-Yb	Ce-Mg-Zn
Ce-Mg-Zr	Ce-Mn-Mo	Ce-Mn-Nb	Ce-Mn-Nd	Ce-Mn-Ni	Ce-Mn-P	Ce-Mn-Pd	Ce-Mn-Pt
Ce-Mn-Ru	Ce-Mn-Sb	Ce-Mn-Sc	Ce-Mn-Si	Ce-Mn-Sm	Ce-Mn-Sn	Ce-Mn-Ta	Ce-Mn-Tb
Ce-Mn-Ti	Ce-Mn-Y	Ce-Mn-Yb	Ce-Mn-Zn	Ce-Mn-Zr	Ce-Mo-Nb	Ce-Mo-Nd	Ce-Mo-Ni
Ce-Mo-P	Ce-Mo-Pd	Ce-Mo-Pt	Ce-Mo-Ru	Ce-Mo-Sb	Ce-Mo-Sc	Ce-Mo-Si	Ce-Mo-Sm
Ce-Mo-Sn	Ce-Mo-Ta	Ce-Mo-Tb	Ce-Mo-Ti	Ce-Mo-Y	Ce-Mo-Yb	Ce-Mo-Zn	Ce-Mo-Zr
Ce-Nb-Nd	Ce-Nb-Ni	Ce-Nb-P	Ce-Nb-Pd	Ce-Nb-Pt	Ce-Nb-Ru	Ce-Nb-Sb	Ce-Nb-Sc
Ce-Nb-Si	Ce-Nb-Sm	Ce-Nb-Sn	Ce-Nb-Ta	Ce-Nb-Tb	Ce-Nb-Ti	Ce-Nb-Y	Ce-Nb-Yb
Ce-Nb-Zn	Ce-Nb-Zr	Ce-Nd-Ni	Ce-Nd-P	Ce-Nd-Pd	Ce-Nd-Pt	Ce-Nd-Ru	Ce-Nd-Sb
Ce-Nd-Sc	Ce-Nd-Si	Ce-Nd-Sm	Ce-Nd-Sn	Ce-Nd-Ta	Ce-Nd-Tb	Ce-Nd-Ti	Ce-Nd-Y

Ce-Nd-Yb	Ce-Nd-Zn	Ce-Nd-Zr	Ce-Ni-P	Ce-Ni-Pd	Ce-Ni-Pt	Ce-Ni-Ru	Ce-Ni-Sb
Ce-Ni-Sc	Ce-Ni-Si	Ce-Ni-Sm	Ce-Ni-Sn	Ce-Ni-Ta	Ce-Ni-Tb	Ce-Ni-Ti	Ce-Ni-Y
Ce-Ni-Yb	Ce-Ni-Zn	Ce-Ni-Zr	Ce-P-Pd	Ce-P-Pt	Ce-P-Ru	Ce-P-Sb	Ce-P-Sc
Ce-P-Si	Ce-P-Sm	Ce-P-Sn	Ce-P-Ta	Ce-P-Tb	Ce-P-Ti	Ce-P-Y	Ce-P-Yb
Ce-P-Zn	Ce-P-Zr	Ce-Pd-Pt	Ce-Pd-Ru	Ce-Pd-Sb	Ce-Pd-Sc	Ce-Pd-Si	Ce-Pd-Sm
Ce-Pd-Sn	Ce-Pd-Ta	Ce-Pd-Tb	Ce-Pd-Ti	Ce-Pd-Y	Ce-Pd-Yb	Ce-Pd-Zn	Ce-Pd-Zr
Ce-Pt-Ru	Ce-Pt-Sb	Ce-Pt-Sc	Ce-Pt-Si	Ce-Pt-Sm	Ce-Pt-Sn	Ce-Pt-Ta	Ce-Pt-Tb
Ce-Pt-Ti	Ce-Pt-Y	Ce-Pt-Yb	Ce-Pt-Zn	Ce-Pt-Zr	Ce-Ru-Sb	Ce-Ru-Sc	Ce-Ru-Si
Ce-Ru-Sm	Ce-Ru-Sn	Ce-Ru-Ta	Ce-Ru-Tb	Ce-Ru-Ti	Ce-Ru-Y	Ce-Ru-Yb	Ce-Ru-Zn
Ce-Ru-Zr	Ce-Sb-Sc	Ce-Sb-Si	Ce-Sb-Sm	Ce-Sb-Sn	Ce-Sb-Ta	Ce-Sb-Tb	Ce-Sb-Ti
Ce-Sb-Y	Ce-Sb-Yb	Ce-Sb-Zn	Ce-Sb-Zr	Ce-Sc-Si	Ce-Sc-Sm	Ce-Sc-Sn	Ce-Sc-Ta
Ce-Sc-Tb	Ce-Sc-Ti	Ce-Sc-Y	Ce-Sc-Yb	Ce-Sc-Zn	Ce-Sc-Zr	Ce-Si-Sm	Ce-Si-Sn
Ce-Si-Ta	Ce-Si-Tb	Ce-Si-Ti	Ce-Si-Y	Ce-Si-Yb	Ce-Si-Zn	Ce-Si-Zr	Ce-Sm-Sn
Ce-Sm-Ta	Ce-Sm-Tb	Ce-Sm-Ti	Ce-Sm-Y	Ce-Sm-Yb	Ce-Sm-Zn	Ce-Sm-Zr	Ce-Sn-Ta
Ce-Sn-Tb	Ce-Sn-Ti	Ce-Sn-Y	Ce-Sn-Yb	Ce-Sn-Zn	Ce-Sn-Zr	Ce-Ta-Tb	Ce-Ta-Ti
Ce-Ta-Y	Ce-Ta-Yb	Ce-Ta-Zn	Ce-Ta-Zr	Ce-Tb-Ti	Ce-Tb-Y	Ce-Tb-Yb	Ce-Tb-Zn
Ce-Tb-Zr	Ce-Ti-Y	Ce-Ti-Yb	Ce-Ti-Zn	Ce-Ti-Zr	Ce-Y-Yb	Ce-Y-Zn	Ce-Y-Zr
Ce-Yb-Zn	Ce-Yb-Zr	Ce-Zn-Zr	Co-Cr-Cu	Co-Cr-Fe	Co-Cr-Gd	Co-Cr-Ge	Co-Cr-Hf
Co-Cr-La	Co-Cr-Mg	Co-Cr-Mn	Co-Cr-Mo	Co-Cr-Nb	Co-Cr-Nd	Co-Cr-Ni	Co-Cr-P
Co-Cr-Pd	Co-Cr-Pt	Co-Cr-Ru	Co-Cr-Sb	Co-Cr-Sc	Co-Cr-Si	Co-Cr-Sm	Co-Cr-Sn
Co-Cr-Ta	Co-Cr-Tb	Co-Cr-Ti	Co-Cr-Y	Co-Cr-Yb	Co-Cr-Zn	Co-Cr-Zr	Co-Cu-Fe
Co-Cu-Gd	Co-Cu-Ge	Co-Cu-Hf	Co-Cu-La	Co-Cu-Mg	Co-Cu-Mn	Co-Cu-Mo	Co-Cu-Nb
Co-Cu-Nd	Co-Cu-Ni	Co-Cu-P	Co-Cu-Pd	Co-Cu-Pt	Co-Cu-Ru	Co-Cu-Sb	Co-Cu-Sc
Co-Cu-Si	Co-Cu-Sm	Co-Cu-Sn	Co-Cu-Ta	Co-Cu-Tb	Co-Cu-Ti	Co-Cu-Y	Co-Cu-Yb
Co-Cu-Zn	Co-Cu-Zr	Co-Fe-Gd	Co-Fe-Ge	Co-Fe-Hf	Co-Fe-La	Co-Fe-Mg	Co-Fe-Mn

Co-Fe-Mo	Co-Fe-Nb	Co-Fe-Nd	Co-Fe-Ni	Co-Fe-P	Co-Fe-Pd	Co-Fe-Pt	Co-Fe-Ru
Co-Fe-Sb	Co-Fe-Sc	Co-Fe-Si	Co-Fe-Sm	Co-Fe-Sn	Co-Fe-Ta	Co-Fe-Tb	Co-Fe-Ti
Co-Fe-Y	Co-Fe-Yb	Co-Fe-Zn	Co-Fe-Zr	Co-Gd-Ge	Co-Gd-Hf	Co-Gd-La	Co-Gd-Mg
Co-Gd-Mn	Co-Gd-Mo	Co-Gd-Nb	Co-Gd-Nd	Co-Gd-Ni	Co-Gd-P	Co-Gd-Pd	Co-Gd-Pt
Co-Gd-Ru	Co-Gd-Sb	Co-Gd-Sc	Co-Gd-Si	Co-Gd-Sm	Co-Gd-Sn	Co-Gd-Ta	Co-Gd-Tb
Co-Gd-Ti	Co-Gd-Y	Co-Gd-Yb	Co-Gd-Zn	Co-Gd-Zr	Co-Ge-Hf	Co-Ge-La	Co-Ge-Mg
Co-Ge-Mn	Co-Ge-Mo	Co-Ge-Nb	Co-Ge-Nd	Co-Ge-Ni	Co-Ge-P	Co-Ge-Pd	Co-Ge-Pt
Co-Ge-Ru	Co-Ge-Sb	Co-Ge-Sc	Co-Ge-Si	Co-Ge-Sm	Co-Ge-Sn	Co-Ge-Ta	Co-Ge-Tb
Co-Ge-Ti	Co-Ge-Y	Co-Ge-Yb	Co-Ge-Zn	Co-Ge-Zr	Co-Hf-La	Co-Hf-Mg	Co-Hf-Mn
Co-Hf-Mo	Co-Hf-Nb	Co-Hf-Nd	Co-Hf-Ni	Co-Hf-P	Co-Hf-Pd	Co-Hf-Pt	Co-Hf-Ru
Co-Hf-Sb	Co-Hf-Sc	Co-Hf-Si	Co-Hf-Sm	Co-Hf-Sn	Co-Hf-Ta	Co-Hf-Tb	Co-Hf-Ti
Co-Hf-Y	Co-Hf-Yb	Co-Hf-Zn	Co-Hf-Zr	Co-La-Mg	Co-La-Mn	Co-La-Mo	Co-La-Nb
Co-La-Nd	Co-La-Ni	Co-La-P	Co-La-Pd	Co-La-Pt	Co-La-Ru	Co-La-Sb	Co-La-Sc
Co-La-Si	Co-La-Sm	Co-La-Sn	Co-La-Ta	Co-La-Tb	Co-La-Ti	Co-La-Y	Co-La-Yb
Co-La-Zn	Co-La-Zr	Co-Mg-Mn	Co-Mg-Mo	Co-Mg-Nb	Co-Mg-Nd	Co-Mg-Ni	Co-Mg-P
Co-Mg-Pd	Co-Mg-Pt	Co-Mg-Ru	Co-Mg-Sb	Co-Mg-Sc	Co-Mg-Si	Co-Mg-Sm	Co-Mg-Sn
Co-Mg-Ta	Co-Mg-Tb	Co-Mg-Ti	Co-Mg-Y	Co-Mg-Yb	Co-Mg-Zn	Co-Mg-Zr	Co-Mn-Mo
Co-Mn-Nb	Co-Mn-Nd	Co-Mn-Ni	Co-Mn-P	Co-Mn-Pd	Co-Mn-Pt	Co-Mn-Ru	Co-Mn-Sb
Co-Mn-Sc	Co-Mn-Si	Co-Mn-Sm	Co-Mn-Sn	Co-Mn-Ta	Co-Mn-Tb	Co-Mn-Ti	Co-Mn-Y
Co-Mn-Yb	Co-Mn-Zn	Co-Mn-Zr	Co-Mo-Nb	Co-Mo-Nd	Co-Mo-Ni	Co-Mo-P	Co-Mo-Pd
Co-Mo-Pt	Co-Mo-Ru	Co-Mo-Sb	Co-Mo-Sc	Co-Mo-Si	Co-Mo-Sm	Co-Mo-Sn	Co-Mo-Ta
Co-Mo-Tb	Co-Mo-Ti	Co-Mo-Y	Co-Mo-Yb	Co-Mo-Zn	Co-Mo-Zr	Co-Nb-Nd	Co-Nb-Ni
Co-Nb-P	Co-Nb-Pd	Co-Nb-Pt	Co-Nb-Ru	Co-Nb-Sb	Co-Nb-Sc	Co-Nb-Si	Co-Nb-Sm
Co-Nb-Sn	Co-Nb-Ta	Co-Nb-Tb	Co-Nb-Ti	Co-Nb-Y	Co-Nb-Yb	Co-Nb-Zn	Co-Nd-Ni
Co-Nd-P	Co-Nd-Pd	Co-Nd-Pt	Co-Nd-Ru	Co-Nd-Sb	Co-Nd-Sc	Co-Nd-Si	Co-Nd-Sm

Co-Nd-Sn	Co-Nd-Ta	Co-Nd-Tb	Co-Nd-Ti	Co-Nd-Y	Co-Nd-Yb	Co-Nd-Zn	Co-Nd-Zr
Co-Ni-P	Co-Ni-Pd	Co-Ni-Pt	Co-Ni-Ru	Co-Ni-Sb	Co-Ni-Sc	Co-Ni-Si	Co-Ni-Sm
Co-Ni-Sn	Co-Ni-Ta	Co-Ni-Tb	Co-Ni-Ti	Co-Ni-Y	Co-Ni-Yb	Co-Ni-Zn	Co-Ni-Zr
Co-P-Pd	Co-P-Pt	Co-P-Ru	Co-P-Sb	Co-P-Sc	Co-P-Si	Co-P-Sm	Co-P-Sn
Co-P-Ta	Co-P-Tb	Co-P-Ti	Co-P-Y	Co-P-Yb	Co-P-Zn	Co-P-Zr	Co-Pd-Pt
Co-Pd-Ru	Co-Pd-Sb	Co-Pd-Sc	Co-Pd-Si	Co-Pd-Sm	Co-Pd-Sn	Co-Pd-Ta	Co-Pd-Tb
Co-Pd-Ti	Co-Pd-Y	Co-Pd-Yb	Co-Pd-Zn	Co-Pd-Zr	Co-Pt-Ru	Co-Pt-Sb	Co-Pt-Sc
Co-Pt-Si	Co-Pt-Sm	Co-Pt-Sn	Co-Pt-Ta	Co-Pt-Tb	Co-Pt-Ti	Co-Pt-Y	Co-Pt-Yb
Co-Pt-Zn	Co-Pt-Zr	Co-Ru-Sb	Co-Ru-Sc	Co-Ru-Si	Co-Ru-Sm	Co-Ru-Sn	Co-Ru-Ta
Co-Ru-Tb	Co-Ru-Ti	Co-Ru-Y	Co-Ru-Yb	Co-Ru-Zn	Co-Ru-Zr	Co-Sb-Sc	Co-Sb-Si
Co-Sb-Sm	Co-Sb-Sn	Co-Sb-Ta	Co-Sb-Tb	Co-Sb-Ti	Co-Sb-Y	Co-Sb-Yb	Co-Sb-Zn
Co-Sb-Zr	Co-Sc-Si	Co-Sc-Sm	Co-Sc-Sn	Co-Sc-Ta	Co-Sc-Tb	Co-Sc-Ti	Co-Sc-Y
Co-Sc-Yb	Co-Sc-Zn	Co-Sc-Zr	Co-Si-Sm	Co-Si-Sn	Co-Si-Ta	Co-Si-Tb	Co-Si-Ti
Co-Si-Y	Co-Si-Yb	Co-Si-Zn	Co-Si-Zr	Co-Sm-Sn	Co-Sm-Ta	Co-Sm-Tb	Co-Sm-Ti
Co-Sm-Y	Co-Sm-Yb	Co-Sm-Zn	Co-Sm-Zr	Co-Sn-Ta	Co-Sn-Tb	Co-Sn-Ti	Co-Sn-Y
Co-Sn-Yb	Co-Sn-Zn	Co-Sn-Zr	Co-Ta-Tb	Co-Ta-Ti	Co-Ta-Y	Co-Ta-Yb	Co-Ta-Zn
Co-Ta-Zr	Co-Tb-Ti	Co-Tb-Y	Co-Tb-Yb	Co-Tb-Zn	Co-Tb-Zr	Co-Ti-Y	Co-Ti-Yb
Co-Ti-Zn	Co-Y-Yb	Co-Y-Zn	Co-Y-Zr	Co-Yb-Zn	Co-Yb-Zr	Co-Zn-Zr	Cr-Cu-Fe
Cr-Cu-Gd	Cr-Cu-Ge	Cr-Cu-Hf	Cr-Cu-La	Cr-Cu-Mg	Cr-Cu-Mn	Cr-Cu-Mo	Cr-Cu-Nb
Cr-Cu-Nd	Cr-Cu-Ni	Cr-Cu-P	Cr-Cu-Pd	Cr-Cu-Pt	Cr-Cu-Ru	Cr-Cu-Sb	Cr-Cu-Sc
Cr-Cu-Si	Cr-Cu-Sm	Cr-Cu-Sn	Cr-Cu-Ta	Cr-Cu-Tb	Cr-Cu-Ti	Cr-Cu-Y	Cr-Cu-Yb
Cr-Cu-Zn	Cr-Cu-Zr	Cr-Fe-Gd	Cr-Fe-Ge	Cr-Fe-Hf	Cr-Fe-La	Cr-Fe-Mg	Cr-Fe-Mn
Cr-Fe-Mo	Cr-Fe-Nb	Cr-Fe-Nd	Cr-Fe-Ni	Cr-Fe-P	Cr-Fe-Pd	Cr-Fe-Pt	Cr-Fe-Ru
Cr-Fe-Sb	Cr-Fe-Sc	Cr-Fe-Si	Cr-Fe-Sm	Cr-Fe-Sn	Cr-Fe-Ta	Cr-Fe-Tb	Cr-Fe-Ti
Cr-Fe-Y	Cr-Fe-Yb	Cr-Fe-Zn	Cr-Fe-Zr	Cr-Gd-Ge	Cr-Gd-Hf	Cr-Gd-La	Cr-Gd-Mg

Cr-Gd-Mn	Cr-Gd-Mo	Cr-Gd-Nb	Cr-Gd-Nd	Cr-Gd-Ni	Cr-Gd-P	Cr-Gd-Pd	Cr-Gd-Pt
Cr-Gd-Ru	Cr-Gd-Sb	Cr-Gd-Sc	Cr-Gd-Si	Cr-Gd-Sm	Cr-Gd-Sn	Cr-Gd-Ta	Cr-Gd-Tb
Cr-Gd-Ti	Cr-Gd-Y	Cr-Gd-Yb	Cr-Gd-Zn	Cr-Gd-Zr	Cr-Ge-Hf	Cr-Ge-La	Cr-Ge-Mg
Cr-Ge-Mn	Cr-Ge-Mo	Cr-Ge-Nb	Cr-Ge-Nd	Cr-Ge-Ni	Cr-Ge-P	Cr-Ge-Pd	Cr-Ge-Pt
Cr-Ge-Ru	Cr-Ge-Sb	Cr-Ge-Sc	Cr-Ge-Si	Cr-Ge-Sm	Cr-Ge-Sn	Cr-Ge-Ta	Cr-Ge-Tb
Cr-Ge-Ti	Cr-Ge-Y	Cr-Ge-Yb	Cr-Ge-Zn	Cr-Ge-Zr	Cr-Hf-La	Cr-Hf-Mg	Cr-Hf-Mn
Cr-Hf-Mo	Cr-Hf-Nb	Cr-Hf-Nd	Cr-Hf-Ni	Cr-Hf-P	Cr-Hf-Pd	Cr-Hf-Pt	Cr-Hf-Ru
Cr-Hf-Sb	Cr-Hf-Sc	Cr-Hf-Si	Cr-Hf-Sm	Cr-Hf-Sn	Cr-Hf-Ta	Cr-Hf-Tb	Cr-Hf-Ti
Cr-Hf-Y	Cr-Hf-Yb	Cr-Hf-Zn	Cr-Hf-Zr	Cr-La-Mg	Cr-La-Mn	Cr-La-Mo	Cr-La-Nb
Cr-La-Nd	Cr-La-Ni	Cr-La-P	Cr-La-Pd	Cr-La-Pt	Cr-La-Ru	Cr-La-Sb	Cr-La-Sc
Cr-La-Si	Cr-La-Sm	Cr-La-Sn	Cr-La-Ta	Cr-La-Tb	Cr-La-Ti	Cr-La-Y	Cr-La-Yb
Cr-La-Zn	Cr-La-Zr	Cr-Mg-Mn	Cr-Mg-Mo	Cr-Mg-Nb	Cr-Mg-Nd	Cr-Mg-Ni	Cr-Mg-P
Cr-Mg-Pd	Cr-Mg-Pt	Cr-Mg-Ru	Cr-Mg-Sb	Cr-Mg-Sc	Cr-Mg-Si	Cr-Mg-Sm	Cr-Mg-Sn
Cr-Mg-Ta	Cr-Mg-Tb	Cr-Mg-Ti	Cr-Mg-Y	Cr-Mg-Yb	Cr-Mg-Zn	Cr-Mg-Zr	Cr-Mn-Mo
Cr-Mn-Nb	Cr-Mn-Nd	Cr-Mn-Ni	Cr-Mn-P	Cr-Mn-Pd	Cr-Mn-Pt	Cr-Mn-Ru	Cr-Mn-Sb
Cr-Mn-Sc	Cr-Mn-Si	Cr-Mn-Sm	Cr-Mn-Sn	Cr-Mn-Ta	Cr-Mn-Tb	Cr-Mn-Ti	Cr-Mn-Y
Cr-Mn-Yb	Cr-Mn-Zn	Cr-Mn-Zr	Cr-Mo-Nb	Cr-Mo-Nd	Cr-Mo-Ni	Cr-Mo-P	Cr-Mo-Pd
Cr-Mo-Pt	Cr-Mo-Ru	Cr-Mo-Sb	Cr-Mo-Sc	Cr-Mo-Si	Cr-Mo-Sm	Cr-Mo-Sn	Cr-Mo-Ta
Cr-Mo-Tb	Cr-Mo-Ti	Cr-Mo-Y	Cr-Mo-Yb	Cr-Mo-Zn	Cr-Mo-Zr	Cr-Nb-Nd	Cr-Nb-Ni
Cr-Nb-P	Cr-Nb-Pd	Cr-Nb-Pt	Cr-Nb-Ru	Cr-Nb-Sb	Cr-Nb-Sc	Cr-Nb-Si	Cr-Nb-Sm
Cr-Nb-Sn	Cr-Nb-Ta	Cr-Nb-Tb	Cr-Nb-Ti	Cr-Nb-Y	Cr-Nb-Yb	Cr-Nb-Zn	Cr-Nb-Zr
Cr-Nd-Ni	Cr-Nd-P	Cr-Nd-Pd	Cr-Nd-Pt	Cr-Nd-Ru	Cr-Nd-Sb	Cr-Nd-Sc	Cr-Nd-Si
Cr-Nd-Sm	Cr-Nd-Sn	Cr-Nd-Ta	Cr-Nd-Tb	Cr-Nd-Ti	Cr-Nd-Y	Cr-Nd-Yb	Cr-Nd-Zn
Cr-Nd-Zr	Cr-Ni-P	Cr-Ni-Pd	Cr-Ni-Pt	Cr-Ni-Ru	Cr-Ni-Sb	Cr-Ni-Sc	Cr-Ni-Si
Cr-Ni-Sm	Cr-Ni-Sn	Cr-Ni-Ta	Cr-Ni-Tb	Cr-Ni-Ti	Cr-Ni-Y	Cr-Ni-Yb	Cr-Ni-Zn

Cr-Ni-Zr	Cr-P-Pd	Cr-P-Pt	Cr-P-Ru	Cr-P-Sb	Cr-P-Sc	Cr-P-Si	Cr-P-Sm
Cr-P-Sn	Cr-P-Ta	Cr-P-Tb	Cr-P-Ti	Cr-P-Y	Cr-P-Yb	Cr-P-Zn	Cr-P-Zr
Cr-Pd-Pt	Cr-Pd-Ru	Cr-Pd-Sb	Cr-Pd-Sc	Cr-Pd-Si	Cr-Pd-Sm	Cr-Pd-Sn	Cr-Pd-Ta
Cr-Pd-Tb	Cr-Pd-Ti	Cr-Pd-Y	Cr-Pd-Yb	Cr-Pd-Zn	Cr-Pd-Zr	Cr-Pt-Ru	Cr-Pt-Sb
Cr-Pt-Sc	Cr-Pt-Si	Cr-Pt-Sm	Cr-Pt-Sn	Cr-Pt-Ta	Cr-Pt-Tb	Cr-Pt-Ti	Cr-Pt-Y
Cr-Pt-Yb	Cr-Pt-Zn	Cr-Pt-Zr	Cr-Ru-Sb	Cr-Ru-Sc	Cr-Ru-Si	Cr-Ru-Sm	Cr-Ru-Sn
Cr-Ru-Ta	Cr-Ru-Tb	Cr-Ru-Ti	Cr-Ru-Y	Cr-Ru-Yb	Cr-Ru-Zn	Cr-Ru-Zr	Cr-Sb-Sc
Cr-Sb-Si	Cr-Sb-Sm	Cr-Sb-Sn	Cr-Sb-Ta	Cr-Sb-Tb	Cr-Sb-Ti	Cr-Sb-Y	Cr-Sb-Yb
Cr-Sb-Zn	Cr-Sb-Zr	Cr-Sc-Si	Cr-Sc-Sm	Cr-Sc-Sn	Cr-Sc-Ta	Cr-Sc-Tb	Cr-Sc-Ti
Cr-Sc-Y	Cr-Sc-Yb	Cr-Sc-Zn	Cr-Sc-Zr	Cr-Si-Sm	Cr-Si-Sn	Cr-Si-Ta	Cr-Si-Tb
Cr-Si-Ti	Cr-Si-Y	Cr-Si-Yb	Cr-Si-Zn	Cr-Si-Zr	Cr-Sm-Sn	Cr-Sm-Ta	Cr-Sm-Tb
Cr-Sm-Ti	Cr-Sm-Y	Cr-Sm-Yb	Cr-Sm-Zn	Cr-Sm-Zr	Cr-Sn-Ta	Cr-Sn-Tb	Cr-Sn-Ti
Cr-Sn-Y	Cr-Sn-Yb	Cr-Sn-Zn	Cr-Sn-Zr	Cr-Ta-Tb	Cr-Ta-Ti	Cr-Ta-Y	Cr-Ta-Yb
Cr-Ta-Zn	Cr-Ta-Zr	Cr-Tb-Ti	Cr-Tb-Y	Cr-Tb-Yb	Cr-Tb-Zn	Cr-Tb-Zr	Cr-Ti-Y
Cr-Ti-Yb	Cr-Ti-Zn	Cr-Ti-Zr	Cr-Y-Yb	Cr-Y-Zn	Cr-Y-Zr	Cr-Yb-Zn	Cr-Yb-Zr
Cr-Zn-Zr	Cu-Fe-Gd	Cu-Fe-Ge	Cu-Fe-Hf	Cu-Fe-La	Cu-Fe-Mg	Cu-Fe-Mn	Cu-Fe-Mo
Cu-Fe-Nb	Cu-Fe-Nd	Cu-Fe-Ni	Cu-Fe-P	Cu-Fe-Pd	Cu-Fe-Pt	Cu-Fe-Ru	Cu-Fe-Sb
Cu-Fe-Sc	Cu-Fe-Si	Cu-Fe-Sm	Cu-Fe-Sn	Cu-Fe-Ta	Cu-Fe-Tb	Cu-Fe-Ti	Cu-Fe-Y
Cu-Fe-Yb	Cu-Fe-Zn	Cu-Fe-Zr	Cu-Gd-Ge	Cu-Gd-Hf	Cu-Gd-La	Cu-Gd-Mg	Cu-Gd-Mn
Cu-Gd-Mo	Cu-Gd-Nb	Cu-Gd-Nd	Cu-Gd-Ni	Cu-Gd-P	Cu-Gd-Pd	Cu-Gd-Pt	Cu-Gd-Ru
Cu-Gd-Sb	Cu-Gd-Sc	Cu-Gd-Si	Cu-Gd-Sm	Cu-Gd-Sn	Cu-Gd-Ta	Cu-Gd-Tb	Cu-Gd-Ti
Cu-Gd-Y	Cu-Gd-Yb	Cu-Gd-Zn	Cu-Gd-Zr	Cu-Ge-Hf	Cu-Ge-La	Cu-Ge-Mg	Cu-Ge-Mn
Cu-Ge-Mo	Cu-Ge-Nb	Cu-Ge-Nd	Cu-Ge-Ni	Cu-Ge-P	Cu-Ge-Pd	Cu-Ge-Pt	Cu-Ge-Ru
Cu-Ge-Sb	Cu-Ge-Sc	Cu-Ge-Si	Cu-Ge-Sm	Cu-Ge-Sn	Cu-Ge-Ta	Cu-Ge-Tb	Cu-Ge-Ti
Cu-Ge-Y	Cu-Ge-Yb	Cu-Ge-Zn	Cu-Ge-Zr	Cu-Hf-La	Cu-Hf-Mg	Cu-Hf-Mn	Cu-Hf-Mo

Cu-Hf-Nb	Cu-Hf-Nd	Cu-Hf-Ni	Cu-Hf-P	Cu-Hf-Pd	Cu-Hf-Pt	Cu-Hf-Ru	Cu-Hf-Sb
Cu-Hf-Sc	Cu-Hf-Si	Cu-Hf-Sm	Cu-Hf-Sn	Cu-Hf-Ta	Cu-Hf-Tb	Cu-Hf-Ti	Cu-Hf-Y
Cu-Hf-Yb	Cu-Hf-Zn	Cu-Hf-Zr	Cu-La-Mg	Cu-La-Mn	Cu-La-Mo	Cu-La-Nb	Cu-La-Nd
Cu-La-Ni	Cu-La-P	Cu-La-Pd	Cu-La-Pt	Cu-La-Ru	Cu-La-Sb	Cu-La-Sc	Cu-La-Si
Cu-La-Sm	Cu-La-Sn	Cu-La-Ta	Cu-La-Tb	Cu-La-Ti	Cu-La-Y	Cu-La-Yb	Cu-La-Zn
Cu-La-Zr	Cu-Mg-Mn	Cu-Mg-Mo	Cu-Mg-Nb	Cu-Mg-Nd	Cu-Mg-Ni	Cu-Mg-P	Cu-Mg-Pd
Cu-Mg-Pt	Cu-Mg-Ru	Cu-Mg-Sb	Cu-Mg-Sc	Cu-Mg-Si	Cu-Mg-Sm	Cu-Mg-Sn	Cu-Mg-Ta
Cu-Mg-Tb	Cu-Mg-Ti	Cu-Mg-Yb	Cu-Mg-Zn	Cu-Mn-Mo	Cu-Mn-Nb	Cu-Mn-Nd	Cu-Mn-Ni
Cu-Mn-P	Cu-Mn-Pd	Cu-Mn-Pt	Cu-Mn-Ru	Cu-Mn-Sb	Cu-Mn-Sc	Cu-Mn-Si	Cu-Mn-Sm
Cu-Mn-Sn	Cu-Mn-Ta	Cu-Mn-Tb	Cu-Mn-Ti	Cu-Mn-Y	Cu-Mn-Yb	Cu-Mn-Zn	Cu-Mn-Zr
Cu-Mo-Nb	Cu-Mo-Nd	Cu-Mo-Ni	Cu-Mo-P	Cu-Mo-Pd	Cu-Mo-Pt	Cu-Mo-Ru	Cu-Mo-Sb
Cu-Mo-Sc	Cu-Mo-Si	Cu-Mo-Sm	Cu-Mo-Sn	Cu-Mo-Ta	Cu-Mo-Tb	Cu-Mo-Ti	Cu-Mo-Y
Cu-Mo-Yb	Cu-Mo-Zn	Cu-Mo-Zr	Cu-Nb-Nd	Cu-Nb-Ni	Cu-Nb-P	Cu-Nb-Pd	Cu-Nb-Pt
Cu-Nb-Ru	Cu-Nb-Sb	Cu-Nb-Sc	Cu-Nb-Si	Cu-Nb-Sm	Cu-Nb-Sn	Cu-Nb-Ta	Cu-Nb-Tb
Cu-Nb-Ti	Cu-Nb-Y	Cu-Nb-Yb	Cu-Nb-Zn	Cu-Nb-Zr	Cu-Nd-Ni	Cu-Nd-P	Cu-Nd-Pd
Cu-Nd-Pt	Cu-Nd-Ru	Cu-Nd-Sb	Cu-Nd-Sc	Cu-Nd-Si	Cu-Nd-Sm	Cu-Nd-Sn	Cu-Nd-Ta
Cu-Nd-Tb	Cu-Nd-Ti	Cu-Nd-Y	Cu-Nd-Yb	Cu-Nd-Zn	Cu-Nd-Zr	Cu-Ni-P	Cu-Ni-Pd
Cu-Ni-Pt	Cu-Ni-Ru	Cu-Ni-Sb	Cu-Ni-Sc	Cu-Ni-Si	Cu-Ni-Sm	Cu-Ni-Sn	Cu-Ni-Ta
Cu-Ni-Tb	Cu-Ni-Ti	Cu-Ni-Y	Cu-Ni-Yb	Cu-Ni-Zn	Cu-Ni-Zr	Cu-P-Pd	Cu-P-Pt
Cu-P-Ru	Cu-P-Sb	Cu-P-Sc	Cu-P-Si	Cu-P-Sm	Cu-P-Sn	Cu-P-Ta	Cu-P-Tb
Cu-P-Ti	Cu-P-Y	Cu-P-Yb	Cu-P-Zn	Cu-P-Zr	Cu-Pd-Pt	Cu-Pd-Ru	Cu-Pd-Sb
Cu-Pd-Sc	Cu-Pd-Si	Cu-Pd-Sm	Cu-Pd-Sn	Cu-Pd-Ta	Cu-Pd-Tb	Cu-Pd-Ti	Cu-Pd-Y
Cu-Pd-Yb	Cu-Pd-Zn	Cu-Pd-Zr	Cu-Pt-Ru	Cu-Pt-Sb	Cu-Pt-Sc	Cu-Pt-Si	Cu-Pt-Sm
Cu-Pt-Sn	Cu-Pt-Ta	Cu-Pt-Tb	Cu-Pt-Ti	Cu-Pt-Y	Cu-Pt-Yb	Cu-Pt-Zn	Cu-Pt-Zr
Cu-Ru-Sb	Cu-Ru-Sc	Cu-Ru-Si	Cu-Ru-Sm	Cu-Ru-Sn	Cu-Ru-Ta	Cu-Ru-Tb	Cu-Ru-Ti

Cu-Ru-Y	Cu-Ru-Yb	Cu-Ru-Zn	Cu-Ru-Zr	Cu-Sb-Sc	Cu-Sb-Si	Cu-Sb-Sm	Cu-Sb-Sn
Cu-Sb-Ta	Cu-Sb-Tb	Cu-Sb-Ti	Cu-Sb-Y	Cu-Sb-Yb	Cu-Sb-Zn	Cu-Sb-Zr	Cu-Sc-Si
Cu-Sc-Sm	Cu-Sc-Sn	Cu-Sc-Ta	Cu-Sc-Tb	Cu-Sc-Ti	Cu-Sc-Y	Cu-Sc-Yb	Cu-Sc-Zn
Cu-Sc-Zr	Cu-Si-Sm	Cu-Si-Sn	Cu-Si-Ta	Cu-Si-Tb	Cu-Si-Ti	Cu-Si-Y	Cu-Si-Yb
Cu-Si-Zn	Cu-Si-Zr	Cu-Sm-Sn	Cu-Sm-Ta	Cu-Sm-Tb	Cu-Sm-Ti	Cu-Sm-Y	Cu-Sm-Yb
Cu-Sm-Zn	Cu-Sm-Zr	Cu-Sn-Ta	Cu-Sn-Tb	Cu-Sn-Ti	Cu-Sn-Y	Cu-Sn-Yb	Cu-Sn-Zn
Cu-Sn-Zr	Cu-Ta-Tb	Cu-Ta-Ti	Cu-Ta-Y	Cu-Ta-Yb	Cu-Ta-Zn	Cu-Ta-Zr	Cu-Tb-Ti
Cu-Tb-Y	Cu-Tb-Yb	Cu-Tb-Zn	Cu-Tb-Zr	Cu-Ti-Y	Cu-Ti-Yb	Cu-Ti-Zn	Cu-Y-Yb
Cu-Y-Zn	Cu-Y-Zr	Cu-Yb-Zn	Cu-Yb-Zr	Cu-Zn-Zr	Fe-Gd-Ge	Fe-Gd-Hf	Fe-Gd-La
Fe-Gd-Mg	Fe-Gd-Mn	Fe-Gd-Mo	Fe-Gd-Nb	Fe-Gd-Nd	Fe-Gd-Ni	Fe-Gd-P	Fe-Gd-Pd
Fe-Gd-Pt	Fe-Gd-Ru	Fe-Gd-Sb	Fe-Gd-Sc	Fe-Gd-Si	Fe-Gd-Sm	Fe-Gd-Sn	Fe-Gd-Ta
Fe-Gd-Tb	Fe-Gd-Ti	Fe-Gd-Y	Fe-Gd-Yb	Fe-Gd-Zn	Fe-Gd-Zr	Fe-Ge-Hf	Fe-Ge-La
Fe-Ge-Mg	Fe-Ge-Mn	Fe-Ge-Mo	Fe-Ge-Nb	Fe-Ge-Nd	Fe-Ge-Ni	Fe-Ge-P	Fe-Ge-Pd
Fe-Ge-Pt	Fe-Ge-Ru	Fe-Ge-Sb	Fe-Ge-Sc	Fe-Ge-Si	Fe-Ge-Sm	Fe-Ge-Sn	Fe-Ge-Ta
Fe-Ge-Tb	Fe-Ge-Ti	Fe-Ge-Y	Fe-Ge-Yb	Fe-Ge-Zn	Fe-Ge-Zr	Fe-Hf-La	Fe-Hf-Mg
Fe-Hf-Mn	Fe-Hf-Mo	Fe-Hf-Nb	Fe-Hf-Nd	Fe-Hf-P	Fe-Hf-Pd	Fe-Hf-Pt	Fe-Hf-Ru
Fe-Hf-Sb	Fe-Hf-Sc	Fe-Hf-Sm	Fe-Hf-Sn	Fe-Hf-Ta	Fe-Hf-Tb	Fe-Hf-Ti	Fe-Hf-Y
Fe-Hf-Yb	Fe-Hf-Zn	Fe-Hf-Zr	Fe-La-Mg	Fe-La-Mn	Fe-La-Mo	Fe-La-Nb	Fe-La-Nd
Fe-La-Ni	Fe-La-P	Fe-La-Pd	Fe-La-Pt	Fe-La-Ru	Fe-La-Sb	Fe-La-Sc	Fe-La-Si
Fe-La-Sm	Fe-La-Sn	Fe-La-Ta	Fe-La-Tb	Fe-La-Ti	Fe-La-Y	Fe-La-Yb	Fe-La-Zn
Fe-La-Zr	Fe-Mg-Mn	Fe-Mg-Mo	Fe-Mg-Nb	Fe-Mg-Nd	Fe-Mg-Ni	Fe-Mg-P	Fe-Mg-Pd
Fe-Mg-Pt	Fe-Mg-Ru	Fe-Mg-Sb	Fe-Mg-Sc	Fe-Mg-Si	Fe-Mg-Sm	Fe-Mg-Sn	Fe-Mg-Ta
Fe-Mg-Tb	Fe-Mg-Ti	Fe-Mg-Y	Fe-Mg-Yb	Fe-Mg-Zn	Fe-Mg-Zr	Fe-Mn-Mo	Fe-Mn-Nb
Fe-Mn-Nd	Fe-Mn-Ni	Fe-Mn-P	Fe-Mn-Pd	Fe-Mn-Pt	Fe-Mn-Ru	Fe-Mn-Sb	Fe-Mn-Sc
Fe-Mn-Si	Fe-Mn-Sm	Fe-Mn-Sn	Fe-Mn-Ta	Fe-Mn-Tb	Fe-Mn-Ti	Fe-Mn-Y	Fe-Mn-Yb

Fe-Mn-Zn	Fe-Mn-Zr	Fe-Mo-Nb	Fe-Mo-Nd	Fe-Mo-Ni	Fe-Mo-P	Fe-Mo-Pd	Fe-Mo-Pt
Fe-Mo-Ru	Fe-Mo-Sb	Fe-Mo-Sc	Fe-Mo-Si	Fe-Mo-Sm	Fe-Mo-Sn	Fe-Mo-Ta	Fe-Mo-Tb
Fe-Mo-Ti	Fe-Mo-Y	Fe-Mo-Yb	Fe-Mo-Zn	Fe-Mo-Zr	Fe-Nb-Nd	Fe-Nb-Ni	Fe-Nb-P
Fe-Nb-Pd	Fe-Nb-Pt	Fe-Nb-Ru	Fe-Nb-Sb	Fe-Nb-Sc	Fe-Nb-Si	Fe-Nb-Sm	Fe-Nb-Sn
Fe-Nb-Ta	Fe-Nb-Tb	Fe-Nb-Ti	Fe-Nb-Y	Fe-Nb-Yb	Fe-Nb-Zn	Fe-Nb-Zr	Fe-Nd-P
Fe-Nd-Pd	Fe-Nd-Pt	Fe-Nd-Ru	Fe-Nd-Sb	Fe-Nd-Sc	Fe-Nd-Si	Fe-Nd-Sm	Fe-Nd-Sn
Fe-Nd-Ta	Fe-Nd-Tb	Fe-Nd-Ti	Fe-Nd-Y	Fe-Nd-Yb	Fe-Nd-Zn	Fe-Nd-Zr	Fe-Ni-Pd
Fe-Ni-Pt	Fe-Ni-Ru	Fe-Ni-Sb	Fe-Ni-Sc	Fe-Ni-Sm	Fe-Ni-Sn	Fe-Ni-Ta	Fe-Ni-Tb
Fe-Ni-Ti	Fe-Ni-Y	Fe-Ni-Yb	Fe-Ni-Zn	Fe-P-Pd	Fe-P-Pt	Fe-P-Ru	Fe-P-Sb
Fe-P-Sc	Fe-P-Si	Fe-P-Sm	Fe-P-Sn	Fe-P-Ta	Fe-P-Tb	Fe-P-Ti	Fe-P-Y
Fe-P-Yb	Fe-P-Zn	Fe-P-Zr	Fe-Pd-Pt	Fe-Pd-Ru	Fe-Pd-Sb	Fe-Pd-Sc	Fe-Pd-Si
Fe-Pd-Sm	Fe-Pd-Sn	Fe-Pd-Ta	Fe-Pd-Tb	Fe-Pd-Ti	Fe-Pd-Y	Fe-Pd-Yb	Fe-Pd-Zn
Fe-Pd-Zr	Fe-Pt-Ru	Fe-Pt-Sb	Fe-Pt-Sc	Fe-Pt-Si	Fe-Pt-Sm	Fe-Pt-Sn	Fe-Pt-Ta
Fe-Pt-Tb	Fe-Pt-Ti	Fe-Pt-Y	Fe-Pt-Yb	Fe-Pt-Zn	Fe-Pt-Zr	Fe-Ru-Sb	Fe-Ru-Sc
Fe-Ru-Si	Fe-Ru-Sm	Fe-Ru-Sn	Fe-Ru-Ta	Fe-Ru-Tb	Fe-Ru-Ti	Fe-Ru-Y	Fe-Ru-Yb
Fe-Ru-Zn	Fe-Ru-Zr	Fe-Sb-Sc	Fe-Sb-Si	Fe-Sb-Sm	Fe-Sb-Sn	Fe-Sb-Ta	Fe-Sb-Tb
Fe-Sb-Ti	Fe-Sb-Y	Fe-Sb-Yb	Fe-Sb-Zn	Fe-Sb-Zr	Fe-Sc-Si	Fe-Sc-Sm	Fe-Sc-Sn
Fe-Sc-Ta	Fe-Sc-Tb	Fe-Sc-Ti	Fe-Sc-Y	Fe-Sc-Yb	Fe-Sc-Zn	Fe-Sc-Zr	Fe-Si-Sm
Fe-Si-Sn	Fe-Si-Ta	Fe-Si-Tb	Fe-Si-Ti	Fe-Si-Y	Fe-Si-Yb	Fe-Si-Zn	Fe-Sm-Sn
Fe-Sm-Ta	Fe-Sm-Tb	Fe-Sm-Ti	Fe-Sm-Y	Fe-Sm-Yb	Fe-Sm-Zn	Fe-Sm-Zr	Fe-Sn-Ta
Fe-Sn-Tb	Fe-Sn-Ti	Fe-Sn-Y	Fe-Sn-Yb	Fe-Sn-Zn	Fe-Sn-Zr	Fe-Ta-Tb	Fe-Ta-Ti
Fe-Ta-Y	Fe-Ta-Yb	Fe-Ta-Zn	Fe-Ta-Zr	Fe-Tb-Ti	Fe-Tb-Y	Fe-Tb-Yb	Fe-Tb-Zn
Fe-Tb-Zr	Fe-Ti-Y	Fe-Ti-Yb	Fe-Ti-Zn	Fe-Ti-Zr	Fe-Y-Yb	Fe-Y-Zn	Fe-Y-Zr
Fe-Yb-Zn	Fe-Yb-Zr	Fe-Zn-Zr	Gd-Ge-Hf	Gd-Ge-La	Gd-Ge-Mg	Gd-Ge-Mn	Gd-Ge-Mo
Gd-Ge-Nb	Gd-Ge-Nd	Gd-Ge-Ni	Gd-Ge-P	Gd-Ge-Pd	Gd-Ge-Pt	Gd-Ge-Ru	Gd-Ge-Sb

Gd-Ge-Sc	Gd-Ge-Si	Gd-Ge-Sm	Gd-Ge-Sn	Gd-Ge-Ta	Gd-Ge-Tb	Gd-Ge-Ti	Gd-Ge-Y
Gd-Ge-Yb	Gd-Ge-Zn	Gd-Ge-Zr	Gd-Hf-La	Gd-Hf-Mg	Gd-Hf-Mn	Gd-Hf-Mo	Gd-Hf-Nb
Gd-Hf-Nd	Gd-Hf-Ni	Gd-Hf-P	Gd-Hf-Pd	Gd-Hf-Pt	Gd-Hf-Ru	Gd-Hf-Sb	Gd-Hf-Sc
Gd-Hf-Si	Gd-Hf-Sm	Gd-Hf-Sn	Gd-Hf-Ta	Gd-Hf-Tb	Gd-Hf-Ti	Gd-Hf-Y	Gd-Hf-Yb
Gd-Hf-Zn	Gd-Hf-Zr	Gd-La-Mg	Gd-La-Mn	Gd-La-Mo	Gd-La-Nb	Gd-La-Nd	Gd-La-Ni
Gd-La-P	Gd-La-Pd	Gd-La-Pt	Gd-La-Ru	Gd-La-Sb	Gd-La-Sc	Gd-La-Si	Gd-La-Sm
Gd-La-Sn	Gd-La-Ta	Gd-La-Tb	Gd-La-Ti	Gd-La-Y	Gd-La-Yb	Gd-La-Zn	Gd-La-Zr
Gd-Mg-Mn	Gd-Mg-Mo	Gd-Mg-Nb	Gd-Mg-Nd	Gd-Mg-Ni	Gd-Mg-P	Gd-Mg-Pd	Gd-Mg-Pt
Gd-Mg-Ru	Gd-Mg-Sb	Gd-Mg-Sc	Gd-Mg-Si	Gd-Mg-Sm	Gd-Mg-Sn	Gd-Mg-Ta	Gd-Mg-Tb
Gd-Mg-Ti	Gd-Mg-Y	Gd-Mg-Yb	Gd-Mg-Zn	Gd-Mg-Zr	Gd-Mn-Mo	Gd-Mn-Nb	Gd-Mn-Nd
Gd-Mn-Ni	Gd-Mn-P	Gd-Mn-Pd	Gd-Mn-Pt	Gd-Mn-Ru	Gd-Mn-Sb	Gd-Mn-Sc	Gd-Mn-Si
Gd-Mn-Sm	Gd-Mn-Sn	Gd-Mn-Ta	Gd-Mn-Tb	Gd-Mn-Ti	Gd-Mn-Y	Gd-Mn-Yb	Gd-Mn-Zn
Gd-Mn-Zr	Gd-Mo-Nb	Gd-Mo-Nd	Gd-Mo-Ni	Gd-Mo-P	Gd-Mo-Pd	Gd-Mo-Pt	Gd-Mo-Ru
Gd-Mo-Sb	Gd-Mo-Sc	Gd-Mo-Si	Gd-Mo-Sm	Gd-Mo-Sn	Gd-Mo-Ta	Gd-Mo-Tb	Gd-Mo-Ti
Gd-Mo-Y	Gd-Mo-Yb	Gd-Mo-Zn	Gd-Mo-Zr	Gd-Nb-Nd	Gd-Nb-Ni	Gd-Nb-P	Gd-Nb-Pd
Gd-Nb-Pt	Gd-Nb-Ru	Gd-Nb-Sb	Gd-Nb-Sc	Gd-Nb-Si	Gd-Nb-Sm	Gd-Nb-Sn	Gd-Nb-Ta
Gd-Nb-Tb	Gd-Nb-Ti	Gd-Nb-Y	Gd-Nb-Yb	Gd-Nb-Zn	Gd-Nb-Zr	Gd-Nd-Ni	Gd-Nd-P
Gd-Nd-Pd	Gd-Nd-Pt	Gd-Nd-Ru	Gd-Nd-Sb	Gd-Nd-Sc	Gd-Nd-Si	Gd-Nd-Sm	Gd-Nd-Sn
Gd-Nd-Ta	Gd-Nd-Tb	Gd-Nd-Ti	Gd-Nd-Y	Gd-Nd-Yb	Gd-Nd-Zn	Gd-Nd-Zr	Gd-Ni-P
Gd-Ni-Pd	Gd-Ni-Pt	Gd-Ni-Ru	Gd-Ni-Sb	Gd-Ni-Sc	Gd-Ni-Si	Gd-Ni-Sm	Gd-Ni-Sn
Gd-Ni-Ta	Gd-Ni-Tb	Gd-Ni-Ti	Gd-Ni-Y	Gd-Ni-Yb	Gd-Ni-Zn	Gd-Ni-Zr	Gd-P-Pd
Gd-P-Pt	Gd-P-Ru	Gd-P-Sb	Gd-P-Sc	Gd-P-Si	Gd-P-Sm	Gd-P-Sn	Gd-P-Ta
Gd-P-Tb	Gd-P-Ti	Gd-P-Y	Gd-P-Yb	Gd-P-Zn	Gd-P-Zr	Gd-Pd-Pt	Gd-Pd-Ru
Gd-Pd-Sb	Gd-Pd-Sc	Gd-Pd-Si	Gd-Pd-Sm	Gd-Pd-Sn	Gd-Pd-Ta	Gd-Pd-Tb	Gd-Pd-Ti
Gd-Pd-Y	Gd-Pd-Yb	Gd-Pd-Zn	Gd-Pd-Zr	Gd-Pt-Ru	Gd-Pt-Sb	Gd-Pt-Sc	Gd-Pt-Si

Gd-Pt-Sm	Gd-Pt-Sn	Gd-Pt-Ta	Gd-Pt-Tb	Gd-Pt-Ti	Gd-Pt-Y	Gd-Pt-Yb	Gd-Pt-Zn
Gd-Pt-Zr	Gd-Ru-Sb	Gd-Ru-Sc	Gd-Ru-Si	Gd-Ru-Sm	Gd-Ru-Sn	Gd-Ru-Ta	Gd-Ru-Tb
Gd-Ru-Ti	Gd-Ru-Y	Gd-Ru-Yb	Gd-Ru-Zn	Gd-Ru-Zr	Gd-Sb-Sc	Gd-Sb-Si	Gd-Sb-Sm
Gd-Sb-Sn	Gd-Sb-Ta	Gd-Sb-Tb	Gd-Sb-Ti	Gd-Sb-Y	Gd-Sb-Yb	Gd-Sb-Zn	Gd-Sb-Zr
Gd-Sc-Si	Gd-Sc-Sm	Gd-Sc-Sn	Gd-Sc-Ta	Gd-Sc-Tb	Gd-Sc-Ti	Gd-Sc-Y	Gd-Sc-Yb
Gd-Sc-Zn	Gd-Sc-Zr	Gd-Si-Sm	Gd-Si-Sn	Gd-Si-Ta	Gd-Si-Tb	Gd-Si-Ti	Gd-Si-Y
Gd-Si-Yb	Gd-Si-Zn	Gd-Si-Zr	Gd-Sm-Sn	Gd-Sm-Ta	Gd-Sm-Tb	Gd-Sm-Ti	Gd-Sm-Y
Gd-Sm-Yb	Gd-Sm-Zn	Gd-Sm-Zr	Gd-Sn-Ta	Gd-Sn-Tb	Gd-Sn-Ti	Gd-Sn-Y	Gd-Sn-Yb
Gd-Sn-Zn	Gd-Sn-Zr	Gd-Ta-Tb	Gd-Ta-Ti	Gd-Ta-Y	Gd-Ta-Yb	Gd-Ta-Zn	Gd-Ta-Zr
Gd-Tb-Ti	Gd-Tb-Y	Gd-Tb-Yb	Gd-Tb-Zn	Gd-Tb-Zr	Gd-Ti-Y	Gd-Ti-Yb	Gd-Ti-Zn
Gd-Ti-Zr	Gd-Y-Yb	Gd-Y-Zn	Gd-Y-Zr	Gd-Yb-Zn	Gd-Yb-Zr	Gd-Zn-Zr	Ge-Hf-La
Ge-Hf-Mg	Ge-Hf-Mn	Ge-Hf-Mo	Ge-Hf-Nb	Ge-Hf-Nd	Ge-Hf-Ni	Ge-Hf-P	Ge-Hf-Pd
Ge-Hf-Pt	Ge-Hf-Ru	Ge-Hf-Sb	Ge-Hf-Sc	Ge-Hf-Si	Ge-Hf-Sm	Ge-Hf-Sn	Ge-Hf-Ta
Ge-Hf-Tb	Ge-Hf-Ti	Ge-Hf-Y	Ge-Hf-Yb	Ge-Hf-Zn	Ge-Hf-Zr	Ge-La-Mg	Ge-La-Mn
Ge-La-Mo	Ge-La-Nb	Ge-La-Nd	Ge-La-Ni	Ge-La-P	Ge-La-Pd	Ge-La-Pt	Ge-La-Ru
Ge-La-Sb	Ge-La-Sc	Ge-La-Si	Ge-La-Sm	Ge-La-Sn	Ge-La-Ta	Ge-La-Tb	Ge-La-Ti
Ge-La-Y	Ge-La-Yb	Ge-La-Zn	Ge-La-Zr	Ge-Mg-Mn	Ge-Mg-Mo	Ge-Mg-Nb	Ge-Mg-Nd
Ge-Mg-Ni	Ge-Mg-P	Ge-Mg-Pd	Ge-Mg-Pt	Ge-Mg-Ru	Ge-Mg-Sb	Ge-Mg-Sc	Ge-Mg-Si
Ge-Mg-Sm	Ge-Mg-Sn	Ge-Mg-Ta	Ge-Mg-Tb	Ge-Mg-Ti	Ge-Mg-Y	Ge-Mg-Yb	Ge-Mg-Zn
Ge-Mg-Zr	Ge-Mn-Mo	Ge-Mn-Nb	Ge-Mn-Nd	Ge-Mn-Ni	Ge-Mn-P	Ge-Mn-Pd	Ge-Mn-Pt
Ge-Mn-Ru	Ge-Mn-Sb	Ge-Mn-Sc	Ge-Mn-Si	Ge-Mn-Sm	Ge-Mn-Sn	Ge-Mn-Ta	Ge-Mn-Tb
Ge-Mn-Ti	Ge-Mn-Y	Ge-Mn-Yb	Ge-Mn-Zn	Ge-Mn-Zr	Ge-Mo-Nb	Ge-Mo-Nd	Ge-Mo-Ni
Ge-Mo-P	Ge-Mo-Pd	Ge-Mo-Pt	Ge-Mo-Ru	Ge-Mo-Sb	Ge-Mo-Sc	Ge-Mo-Si	Ge-Mo-Sm
Ge-Mo-Sn	Ge-Mo-Ta	Ge-Mo-Tb	Ge-Mo-Ti	Ge-Mo-Y	Ge-Mo-Yb	Ge-Mo-Zn	Ge-Mo-Zr
Ge-Nb-Nd	Ge-Nb-Ni	Ge-Nb-P	Ge-Nb-Pd	Ge-Nb-Pt	Ge-Nb-Ru	Ge-Nb-Sb	Ge-Nb-Sc

Ge-Nb-Si	Ge-Nb-Sm	Ge-Nb-Sn	Ge-Nb-Ta	Ge-Nb-Tb	Ge-Nb-Ti	Ge-Nb-Y	Ge-Nb-Yb
Ge-Nb-Zn	Ge-Nb-Zr	Ge-Nd-Ni	Ge-Nd-P	Ge-Nd-Pd	Ge-Nd-Pt	Ge-Nd-Ru	Ge-Nd-Sb
Ge-Nd-Sc	Ge-Nd-Si	Ge-Nd-Sm	Ge-Nd-Sn	Ge-Nd-Ta	Ge-Nd-Tb	Ge-Nd-Ti	Ge-Nd-Y
Ge-Nd-Yb	Ge-Nd-Zn	Ge-Nd-Zr	Ge-Ni-P	Ge-Ni-Pd	Ge-Ni-Pt	Ge-Ni-Ru	Ge-Ni-Sb
Ge-Ni-Sc	Ge-Ni-Si	Ge-Ni-Sm	Ge-Ni-Sn	Ge-Ni-Ta	Ge-Ni-Tb	Ge-Ni-Ti	Ge-Ni-Y
Ge-Ni-Yb	Ge-Ni-Zn	Ge-Ni-Zr	Ge-P-Pd	Ge-P-Pt	Ge-P-Ru	Ge-P-Sb	Ge-P-Sc
Ge-P-Si	Ge-P-Sm	Ge-P-Sn	Ge-P-Ta	Ge-P-Tb	Ge-P-Ti	Ge-P-Y	Ge-P-Yb
Ge-P-Zn	Ge-P-Zr	Ge-Pd-Pt	Ge-Pd-Ru	Ge-Pd-Sb	Ge-Pd-Sc	Ge-Pd-Si	Ge-Pd-Sm
Ge-Pd-Sn	Ge-Pd-Ta	Ge-Pd-Tb	Ge-Pd-Ti	Ge-Pd-Y	Ge-Pd-Yb	Ge-Pd-Zn	Ge-Pt-Ru
Ge-Pt-Sb	Ge-Pt-Sc	Ge-Pt-Si	Ge-Pt-Sm	Ge-Pt-Sn	Ge-Pt-Ta	Ge-Pt-Tb	Ge-Pt-Ti
Ge-Pt-Y	Ge-Pt-Yb	Ge-Pt-Zn	Ge-Ru-Sb	Ge-Ru-Sc	Ge-Ru-Si	Ge-Ru-Sm	Ge-Ru-Sn
Ge-Ru-Ta	Ge-Ru-Tb	Ge-Ru-Ti	Ge-Ru-Y	Ge-Ru-Yb	Ge-Ru-Zn	Ge-Ru-Zr	Ge-Sb-Sc
Ge-Sb-Si	Ge-Sb-Sm	Ge-Sb-Sn	Ge-Sb-Ta	Ge-Sb-Tb	Ge-Sb-Ti	Ge-Sb-Y	Ge-Sb-Yb
Ge-Sb-Zn	Ge-Sb-Zr	Ge-Sc-Si	Ge-Sc-Sm	Ge-Sc-Sn	Ge-Sc-Ta	Ge-Sc-Tb	Ge-Sc-Ti
Ge-Sc-Y	Ge-Sc-Yb	Ge-Sc-Zn	Ge-Sc-Zr	Ge-Si-Sm	Ge-Si-Sn	Ge-Si-Ta	Ge-Si-Tb
Ge-Si-Ti	Ge-Si-Y	Ge-Si-Yb	Ge-Si-Zn	Ge-Si-Zr	Ge-Sm-Sn	Ge-Sm-Ta	Ge-Sm-Tb
Ge-Sm-Ti	Ge-Sm-Y	Ge-Sm-Yb	Ge-Sm-Zn	Ge-Sm-Zr	Ge-Sn-Ta	Ge-Sn-Tb	Ge-Sn-Ti
Ge-Sn-Y	Ge-Sn-Yb	Ge-Sn-Zn	Ge-Sn-Zr	Ge-Ta-Tb	Ge-Ta-Ti	Ge-Ta-Y	Ge-Ta-Yb
Ge-Ta-Zn	Ge-Ta-Zr	Ge-Tb-Ti	Ge-Tb-Y	Ge-Tb-Yb	Ge-Tb-Zn	Ge-Tb-Zr	Ge-Ti-Y
Ge-Ti-Yb	Ge-Ti-Zn	Ge-Ti-Zr	Ge-Y-Yb	Ge-Y-Zn	Ge-Y-Zr	Ge-Yb-Zn	Ge-Yb-Zr
Ge-Zn-Zr	Hf-La-Mg	Hf-La-Mn	Hf-La-Mo	Hf-La-Nb	Hf-La-Nd	Hf-La-Ni	Hf-La-P
Hf-La-Pd	Hf-La-Pt	Hf-La-Ru	Hf-La-Sb	Hf-La-Sc	Hf-La-Si	Hf-La-Sm	Hf-La-Sn
Hf-La-Ta	Hf-La-Tb	Hf-La-Ti	Hf-La-Y	Hf-La-Yb	Hf-La-Zn	Hf-La-Zr	Hf-Mg-Mn
Hf-Mg-Mo	Hf-Mg-Nb	Hf-Mg-Nd	Hf-Mg-Ni	Hf-Mg-P	Hf-Mg-Pd	Hf-Mg-Pt	Hf-Mg-Ru
Hf-Mg-Sb	Hf-Mg-Sc	Hf-Mg-Si	Hf-Mg-Sm	Hf-Mg-Sn	Hf-Mg-Ta	Hf-Mg-Tb	Hf-Mg-Ti

Hf-Mg-Y	Hf-Mg-Yb	Hf-Mg-Zn	Hf-Mg-Zr	Hf-Mn-Mo	Hf-Mn-Nb	Hf-Mn-Nd	Hf-Mn-Ni
Hf-Mn-P	Hf-Mn-Pd	Hf-Mn-Pt	Hf-Mn-Ru	Hf-Mn-Sb	Hf-Mn-Sc	Hf-Mn-Si	Hf-Mn-Sm
Hf-Mn-Sn	Hf-Mn-Ta	Hf-Mn-Tb	Hf-Mn-Ti	Hf-Mn-Y	Hf-Mn-Yb	Hf-Mn-Zn	Hf-Mn-Zr
Hf-Mo-Nb	Hf-Mo-Nd	Hf-Mo-Ni	Hf-Mo-P	Hf-Mo-Pd	Hf-Mo-Pt	Hf-Mo-Ru	Hf-Mo-Sb
Hf-Mo-Sc	Hf-Mo-Si	Hf-Mo-Sm	Hf-Mo-Sn	Hf-Mo-Ta	Hf-Mo-Tb	Hf-Mo-Ti	Hf-Mo-Y
Hf-Mo-Yb	Hf-Mo-Zn	Hf-Mo-Zr	Hf-Nb-Nd	Hf-Nb-Ni	Hf-Nb-P	Hf-Nb-Pd	Hf-Nb-Pt
Hf-Nb-Ru	Hf-Nb-Sb	Hf-Nb-Sc	Hf-Nb-Si	Hf-Nb-Sm	Hf-Nb-Sn	Hf-Nb-Ta	Hf-Nb-Tb
Hf-Nb-Ti	Hf-Nb-Y	Hf-Nb-Yb	Hf-Nb-Zn	Hf-Nb-Zr	Hf-Nd-Ni	Hf-Nd-P	Hf-Nd-Pd
Hf-Nd-Pt	Hf-Nd-Ru	Hf-Nd-Sb	Hf-Nd-Sc	Hf-Nd-Si	Hf-Nd-Sm	Hf-Nd-Sn	Hf-Nd-Ta
Hf-Nd-Tb	Hf-Nd-Ti	Hf-Nd-Y	Hf-Nd-Yb	Hf-Nd-Zn	Hf-Nd-Zr	Hf-Ni-P	Hf-Ni-Pd
Hf-Ni-Pt	Hf-Ni-Ru	Hf-Ni-Sb	Hf-Ni-Sc	Hf-Ni-Sm	Hf-Ni-Sn	Hf-Ni-Ta	Hf-Ni-Tb
Hf-Ni-Ti	Hf-Ni-Y	Hf-Ni-Yb	Hf-Ni-Zn	Hf-Ni-Zr	Hf-P-Pd	Hf-P-Pt	Hf-P-Ru
Hf-P-Sb	Hf-P-Sc	Hf-P-Si	Hf-P-Sm	Hf-P-Sn	Hf-P-Ta	Hf-P-Tb	Hf-P-Ti
Hf-P-Y	Hf-P-Yb	Hf-P-Zn	Hf-P-Zr	Hf-Pd-Pt	Hf-Pd-Ru	Hf-Pd-Sb	Hf-Pd-Sc
Hf-Pd-Si	Hf-Pd-Sm	Hf-Pd-Sn	Hf-Pd-Ta	Hf-Pd-Tb	Hf-Pd-Ti	Hf-Pd-Y	Hf-Pd-Yb
Hf-Pd-Zn	Hf-Pd-Zr	Hf-Pt-Ru	Hf-Pt-Sb	Hf-Pt-Sc	Hf-Pt-Si	Hf-Pt-Sm	Hf-Pt-Sn
Hf-Pt-Ta	Hf-Pt-Tb	Hf-Pt-Ti	Hf-Pt-Y	Hf-Pt-Yb	Hf-Pt-Zn	Hf-Pt-Zr	Hf-Ru-Sb
Hf-Ru-Sc	Hf-Ru-Si	Hf-Ru-Sm	Hf-Ru-Sn	Hf-Ru-Ta	Hf-Ru-Tb	Hf-Ru-Ti	Hf-Ru-Y
Hf-Ru-Yb	Hf-Ru-Zn	Hf-Ru-Zr	Hf-Sb-Sc	Hf-Sb-Si	Hf-Sb-Sm	Hf-Sb-Sn	Hf-Sb-Ta
Hf-Sb-Tb	Hf-Sb-Ti	Hf-Sb-Y	Hf-Sb-Yb	Hf-Sb-Zn	Hf-Sb-Zr	Hf-Sc-Si	Hf-Sc-Sm
Hf-Sc-Sn	Hf-Sc-Ta	Hf-Sc-Tb	Hf-Sc-Ti	Hf-Sc-Y	Hf-Sc-Yb	Hf-Sc-Zn	Hf-Sc-Zr
Hf-Si-Sm	Hf-Si-Sn	Hf-Si-Ta	Hf-Si-Tb	Hf-Si-Ti	Hf-Si-Y	Hf-Si-Yb	Hf-Si-Zn
Hf-Si-Zr	Hf-Sm-Sn	Hf-Sm-Ta	Hf-Sm-Tb	Hf-Sm-Ti	Hf-Sm-Y	Hf-Sm-Yb	Hf-Sm-Zn
Hf-Sm-Zr	Hf-Sn-Ta	Hf-Sn-Tb	Hf-Sn-Ti	Hf-Sn-Y	Hf-Sn-Yb	Hf-Sn-Zn	Hf-Sn-Zr
Hf-Ta-Tb	Hf-Ta-Ti	Hf-Ta-Y	Hf-Ta-Yb	Hf-Ta-Zn	Hf-Ta-Zr	Hf-Tb-Ti	Hf-Tb-Y

Hf-Tb-Yb	Hf-Tb-Zn	Hf-Tb-Zr	Hf-Ti-Y	Hf-Ti-Yb	Hf-Ti-Zn	Hf-Ti-Zr	Hf-Y-Yb
Hf-Y-Zn	Hf-Y-Zr	Hf-Yb-Zn	Hf-Yb-Zr	Hf-Zn-Zr	La-Mg-Mn	La-Mg-Mo	La-Mg-Nb
La-Mg-Nd	La-Mg-Ni	La-Mg-P	La-Mg-Pd	La-Mg-Pt	La-Mg-Ru	La-Mg-Sb	La-Mg-Sc
La-Mg-Si	La-Mg-Sm	La-Mg-Sn	La-Mg-Ta	La-Mg-Tb	La-Mg-Ti	La-Mg-Y	La-Mg-Yb
La-Mg-Zn	La-Mg-Zr	La-Mn-Mo	La-Mn-Nb	La-Mn-Nd	La-Mn-Ni	La-Mn-P	La-Mn-Pd
La-Mn-Pt	La-Mn-Ru	La-Mn-Sb	La-Mn-Sc	La-Mn-Si	La-Mn-Sm	La-Mn-Sn	La-Mn-Ta
La-Mn-Tb	La-Mn-Ti	La-Mn-Y	La-Mn-Yb	La-Mn-Zn	La-Mn-Zr	La-Mo-Nb	La-Mo-Nd
La-Mo-Ni	La-Mo-P	La-Mo-Pd	La-Mo-Pt	La-Mo-Ru	La-Mo-Sb	La-Mo-Sc	La-Mo-Si
La-Mo-Sm	La-Mo-Sn	La-Mo-Ta	La-Mo-Tb	La-Mo-Ti	La-Mo-Y	La-Mo-Yb	La-Mo-Zn
La-Mo-Zr	La-Nb-Nd	La-Nb-Ni	La-Nb-P	La-Nb-Pd	La-Nb-Pt	La-Nb-Ru	La-Nb-Sb
La-Nb-Sc	La-Nb-Si	La-Nb-Sm	La-Nb-Sn	La-Nb-Ta	La-Nb-Tb	La-Nb-Ti	La-Nb-Y
La-Nb-Yb	La-Nb-Zn	La-Nb-Zr	La-Nd-Ni	La-Nd-P	La-Nd-Pd	La-Nd-Pt	La-Nd-Ru
La-Nd-Sb	La-Nd-Sc	La-Nd-Si	La-Nd-Sm	La-Nd-Sn	La-Nd-Ta	La-Nd-Tb	La-Nd-Ti
La-Nd-Y	La-Nd-Yb	La-Nd-Zn	La-Nd-Zr	La-Ni-P	La-Ni-Pd	La-Ni-Pt	La-Ni-Ru
La-Ni-Sb	La-Ni-Sc	La-Ni-Si	La-Ni-Sm	La-Ni-Sn	La-Ni-Ta	La-Ni-Tb	La-Ni-Ti
La-Ni-Y	La-Ni-Yb	La-Ni-Zn	La-Ni-Zr	La-P-Pd	La-P-Pt	La-P-Ru	La-P-Sb
La-P-Sc	La-P-Si	La-P-Sm	La-P-Sn	La-P-Ta	La-P-Tb	La-P-Ti	La-P-Y
La-P-Yb	La-P-Zn	La-P-Zr	La-Pd-Pt	La-Pd-Ru	La-Pd-Sb	La-Pd-Sc	La-Pd-Si
La-Pd-Sm	La-Pd-Sn	La-Pd-Ta	La-Pd-Tb	La-Pd-Ti	La-Pd-Y	La-Pd-Yb	La-Pd-Zn
La-Pd-Zr	La-Pt-Ru	La-Pt-Sb	La-Pt-Sc	La-Pt-Si	La-Pt-Sm	La-Pt-Sn	La-Pt-Ta
La-Pt-Tb	La-Pt-Ti	La-Pt-Y	La-Pt-Yb	La-Pt-Zn	La-Pt-Zr	La-Ru-Sb	La-Ru-Sc
La-Ru-Si	La-Ru-Sm	La-Ru-Sn	La-Ru-Ta	La-Ru-Tb	La-Ru-Ti	La-Ru-Y	La-Ru-Yb
La-Ru-Zn	La-Ru-Zr	La-Sb-Sc	La-Sb-Si	La-Sb-Sm	La-Sb-Sn	La-Sb-Ta	La-Sb-Tb
La-Sb-Ti	La-Sb-Y	La-Sb-Yb	La-Sb-Zn	La-Sb-Zr	La-Sc-Si	La-Sc-Sm	La-Sc-Sn
La-Sc-Ta	La-Sc-Tb	La-Sc-Ti	La-Sc-Y	La-Sc-Yb	La-Sc-Zn	La-Sc-Zr	La-Si-Sm

La-Si-Sn	La-Si-Ta	La-Si-Tb	La-Si-Ti	La-Si-Y	La-Si-Yb	La-Si-Zn	La-Si-Zr
La-Sm-Sn	La-Sm-Ta	La-Sm-Tb	La-Sm-Ti	La-Sm-Y	La-Sm-Yb	La-Sm-Zn	La-Sm-Zr
La-Sn-Ta	La-Sn-Tb	La-Sn-Ti	La-Sn-Y	La-Sn-Yb	La-Sn-Zn	La-Sn-Zr	La-Ta-Tb
La-Ta-Ti	La-Ta-Y	La-Ta-Yb	La-Ta-Zn	La-Ta-Zr	La-Tb-Ti	La-Tb-Y	La-Tb-Yb
La-Tb-Zn	La-Tb-Zr	La-Ti-Y	La-Ti-Yb	La-Ti-Zn	La-Ti-Zr	La-Y-Yb	La-Y-Zn
La-Y-Zr	La-Yb-Zn	La-Yb-Zr	La-Zn-Zr	Mg-Mn-Mo	Mg-Mn-Nb	Mg-Mn-Nd	Mg-Mn-Ni
Mg-Mn-P	Mg-Mn-Pd	Mg-Mn-Pt	Mg-Mn-Ru	Mg-Mn-Sb	Mg-Mn-Sc	Mg-Mn-Si	Mg-Mn-Sm
Mg-Mn-Sn	Mg-Mn-Ta	Mg-Mn-Tb	Mg-Mn-Ti	Mg-Mn-Y	Mg-Mn-Yb	Mg-Mn-Zn	Mg-Mn-Zr
Mg-Mo-Nb	Mg-Mo-Nd	Mg-Mo-Ni	Mg-Mo-P	Mg-Mo-Pd	Mg-Mo-Pt	Mg-Mo-Ru	Mg-Mo-Sb
Mg-Mo-Sc	Mg-Mo-Si	Mg-Mo-Sm	Mg-Mo-Sn	Mg-Mo-Ta	Mg-Mo-Tb	Mg-Mo-Ti	Mg-Mo-Y
Mg-Mo-Yb	Mg-Mo-Zn	Mg-Mo-Zr	Mg-Nb-Nd	Mg-Nb-Ni	Mg-Nb-P	Mg-Nb-Pd	Mg-Nb-Pt
Mg-Nb-Ru	Mg-Nb-Sb	Mg-Nb-Sc	Mg-Nb-Si	Mg-Nb-Sm	Mg-Nb-Sn	Mg-Nb-Ta	Mg-Nb-Tb
Mg-Nb-Ti	Mg-Nb-Y	Mg-Nb-Yb	Mg-Nb-Zn	Mg-Nb-Zr	Mg-Nd-Ni	Mg-Nd-P	Mg-Nd-Pd
Mg-Nd-Pt	Mg-Nd-Ru	Mg-Nd-Sb	Mg-Nd-Sc	Mg-Nd-Si	Mg-Nd-Sm	Mg-Nd-Sn	Mg-Nd-Ta
Mg-Nd-Tb	Mg-Nd-Ti	Mg-Nd-Y	Mg-Nd-Yb	Mg-Nd-Zn	Mg-Nd-Zr	Mg-Ni-P	Mg-Ni-Pd
Mg-Ni-Pt	Mg-Ni-Ru	Mg-Ni-Sb	Mg-Ni-Sc	Mg-Ni-Si	Mg-Ni-Sm	Mg-Ni-Sn	Mg-Ni-Ta
Mg-Ni-Tb	Mg-Ni-Ti	Mg-Ni-Yb	Mg-Ni-Zn	Mg-P-Pd	Mg-P-Pt	Mg-P-Ru	Mg-P-Sb
Mg-P-Sc	Mg-P-Si	Mg-P-Sm	Mg-P-Sn	Mg-P-Ta	Mg-P-Tb	Mg-P-Ti	Mg-P-Y
Mg-P-Yb	Mg-P-Zn	Mg-P-Zr	Mg-Pd-Pt	Mg-Pd-Ru	Mg-Pd-Sb	Mg-Pd-Sc	Mg-Pd-Si
Mg-Pd-Sm	Mg-Pd-Sn	Mg-Pd-Ta	Mg-Pd-Tb	Mg-Pd-Ti	Mg-Pd-Y	Mg-Pd-Yb	Mg-Pd-Zn
Mg-Pd-Zr	Mg-Pt-Ru	Mg-Pt-Sb	Mg-Pt-Sc	Mg-Pt-Si	Mg-Pt-Sm	Mg-Pt-Sn	Mg-Pt-Ta
Mg-Pt-Tb	Mg-Pt-Ti	Mg-Pt-Y	Mg-Pt-Yb	Mg-Pt-Zn	Mg-Pt-Zr	Mg-Ru-Sb	Mg-Ru-Sc
Mg-Ru-Si	Mg-Ru-Sm	Mg-Ru-Sn	Mg-Ru-Ta	Mg-Ru-Tb	Mg-Ru-Ti	Mg-Ru-Y	Mg-Ru-Yb
Mg-Ru-Zn	Mg-Ru-Zr	Mg-Sb-Sc	Mg-Sb-Si	Mg-Sb-Sm	Mg-Sb-Sn	Mg-Sb-Ta	Mg-Sb-Tb
Mg-Sb-Ti	Mg-Sb-Y	Mg-Sb-Yb	Mg-Sb-Zn	Mg-Sb-Zr	Mg-Sc-Si	Mg-Sc-Sm	Mg-Sc-Sn

Mg-Sc-Ta	Mg-Sc-Tb	Mg-Sc-Ti	Mg-Sc-Y	Mg-Sc-Yb	Mg-Sc-Zn	Mg-Sc-Zr	Mg-Si-Sm
Mg-Si-Sn	Mg-Si-Ta	Mg-Si-Tb	Mg-Si-Ti	Mg-Si-Y	Mg-Si-Yb	Mg-Si-Zn	Mg-Si-Zr
Mg-Sm-Sn	Mg-Sm-Ta	Mg-Sm-Tb	Mg-Sm-Ti	Mg-Sm-Y	Mg-Sm-Yb	Mg-Sm-Zn	Mg-Sm-Zr
Mg-Sn-Ta	Mg-Sn-Tb	Mg-Sn-Ti	Mg-Sn-Y	Mg-Sn-Yb	Mg-Sn-Zn	Mg-Sn-Zr	Mg-Ta-Tb
Mg-Ta-Ti	Mg-Ta-Y	Mg-Ta-Yb	Mg-Ta-Zn	Mg-Ta-Zr	Mg-Tb-Ti	Mg-Tb-Y	Mg-Tb-Yb
Mg-Tb-Zn	Mg-Tb-Zr	Mg-Ti-Y	Mg-Ti-Yb	Mg-Ti-Zn	Mg-Ti-Zr	Mg-Y-Yb	Mg-Y-Zn
Mg-Y-Zr	Mg-Yb-Zn	Mg-Yb-Zr	Mg-Zn-Zr	Mn-Mo-Nb	Mn-Mo-Nd	Mn-Mo-Ni	Mn-Mo-P
Mn-Mo-Pd	Mn-Mo-Pt	Mn-Mo-Ru	Mn-Mo-Sb	Mn-Mo-Sc	Mn-Mo-Si	Mn-Mo-Sm	Mn-Mo-Sn
Mn-Mo-Ta	Mn-Mo-Tb	Mn-Mo-Ti	Mn-Mo-Y	Mn-Mo-Yb	Mn-Mo-Zn	Mn-Mo-Zr	Mn-Nb-Nd
Mn-Nb-Ni	Mn-Nb-P	Mn-Nb-Pd	Mn-Nb-Pt	Mn-Nb-Ru	Mn-Nb-Sb	Mn-Nb-Sc	Mn-Nb-Si
Mn-Nb-Sm	Mn-Nb-Sn	Mn-Nb-Ta	Mn-Nb-Tb	Mn-Nb-Ti	Mn-Nb-Y	Mn-Nb-Yb	Mn-Nb-Zn
Mn-Nb-Zr	Mn-Nd-Ni	Mn-Nd-P	Mn-Nd-Pd	Mn-Nd-Pt	Mn-Nd-Ru	Mn-Nd-Sb	Mn-Nd-Sc
Mn-Nd-Si	Mn-Nd-Sm	Mn-Nd-Sn	Mn-Nd-Ta	Mn-Nd-Tb	Mn-Nd-Ti	Mn-Nd-Y	Mn-Nd-Yb
Mn-Nd-Zn	Mn-Nd-Zr	Mn-Ni-P	Mn-Ni-Pd	Mn-Ni-Pt	Mn-Ni-Ru	Mn-Ni-Sb	Mn-Ni-Sc
Mn-Ni-Si	Mn-Ni-Sm	Mn-Ni-Sn	Mn-Ni-Ta	Mn-Ni-Tb	Mn-Ni-Ti	Mn-Ni-Y	Mn-Ni-Yb
Mn-Ni-Zn	Mn-Ni-Zr	Mn-P-Pd	Mn-P-Pt	Mn-P-Ru	Mn-P-Sb	Mn-P-Sc	Mn-P-Si
Mn-P-Sm	Mn-P-Sn	Mn-P-Ta	Mn-P-Tb	Mn-P-Ti	Mn-P-Y	Mn-P-Yb	Mn-P-Zn
Mn-P-Zr	Mn-Pd-Pt	Mn-Pd-Ru	Mn-Pd-Sb	Mn-Pd-Sc	Mn-Pd-Si	Mn-Pd-Sm	Mn-Pd-Sn
Mn-Pd-Ta	Mn-Pd-Tb	Mn-Pd-Ti	Mn-Pd-Y	Mn-Pd-Yb	Mn-Pd-Zn	Mn-Pd-Zr	Mn-Pt-Ru
Mn-Pt-Sb	Mn-Pt-Sc	Mn-Pt-Si	Mn-Pt-Sm	Mn-Pt-Sn	Mn-Pt-Ta	Mn-Pt-Tb	Mn-Pt-Ti
Mn-Pt-Y	Mn-Pt-Yb	Mn-Pt-Zn	Mn-Pt-Zr	Mn-Ru-Sb	Mn-Ru-Sc	Mn-Ru-Si	Mn-Ru-Sm
Mn-Ru-Sn	Mn-Ru-Ta	Mn-Ru-Tb	Mn-Ru-Ti	Mn-Ru-Y	Mn-Ru-Yb	Mn-Ru-Zn	Mn-Ru-Zr
Mn-Sb-Sc	Mn-Sb-Si	Mn-Sb-Sm	Mn-Sb-Sn	Mn-Sb-Ta	Mn-Sb-Tb	Mn-Sb-Ti	Mn-Sb-Y
Mn-Sb-Yb	Mn-Sb-Zn	Mn-Sb-Zr	Mn-Sc-Si	Mn-Sc-Sm	Mn-Sc-Sn	Mn-Sc-Ta	Mn-Sc-Tb
Mn-Sc-Ti	Mn-Sc-Y	Mn-Sc-Yb	Mn-Sc-Zn	Mn-Sc-Zr	Mn-Si-Sm	Mn-Si-Sn	Mn-Si-Ta

Mn-Si-Tb	Mn-Si-Ti	Mn-Si-Y	Mn-Si-Yb	Mn-Si-Zn	Mn-Sm-Sn	Mn-Sm-Ta	Mn-Sm-Tb
Mn-Sm-Ti	Mn-Sm-Y	Mn-Sm-Yb	Mn-Sm-Zn	Mn-Sm-Zr	Mn-Sn-Ta	Mn-Sn-Tb	Mn-Sn-Ti
Mn-Sn-Y	Mn-Sn-Yb	Mn-Sn-Zn	Mn-Sn-Zr	Mn-Ta-Tb	Mn-Ta-Ti	Mn-Ta-Y	Mn-Ta-Yb
Mn-Ta-Zn	Mn-Ta-Zr	Mn-Tb-Ti	Mn-Tb-Y	Mn-Tb-Yb	Mn-Tb-Zn	Mn-Tb-Zr	Mn-Ti-Y
Mn-Ti-Yb	Mn-Ti-Zn	Mn-Ti-Zr	Mn-Y-Yb	Mn-Y-Zn	Mn-Y-Zr	Mn-Yb-Zn	Mn-Yb-Zr
Mn-Zn-Zr	Mo-Nb-Nd	Mo-Nb-Ni	Mo-Nb-P	Mo-Nb-Pd	Mo-Nb-Pt	Mo-Nb-Ru	Mo-Nb-Sb
Mo-Nb-Sc	Mo-Nb-Si	Mo-Nb-Sm	Mo-Nb-Sn	Mo-Nb-Ta	Mo-Nb-Tb	Mo-Nb-Ti	Mo-Nb-Y
Mo-Nb-Yb	Mo-Nb-Zn	Mo-Nb-Zr	Mo-Nd-Ni	Mo-Nd-P	Mo-Nd-Pd	Mo-Nd-Pt	Mo-Nd-Ru
Mo-Nd-Sb	Mo-Nd-Sc	Mo-Nd-Si	Mo-Nd-Sm	Mo-Nd-Sn	Mo-Nd-Ta	Mo-Nd-Tb	Mo-Nd-Ti
Mo-Nd-Y	Mo-Nd-Yb	Mo-Nd-Zn	Mo-Nd-Zr	Mo-Ni-P	Mo-Ni-Pd	Mo-Ni-Pt	Mo-Ni-Ru
Mo-Ni-Sb	Mo-Ni-Sc	Mo-Ni-Si	Mo-Ni-Sm	Mo-Ni-Sn	Mo-Ni-Ta	Mo-Ni-Tb	Mo-Ni-Ti
Mo-Ni-Y	Mo-Ni-Yb	Mo-Ni-Zn	Mo-P-Pd	Mo-P-Pt	Mo-P-Ru	Mo-P-Sb	Mo-P-Sc
Mo-P-Si	Mo-P-Sm	Mo-P-Sn	Mo-P-Ta	Mo-P-Tb	Mo-P-Ti	Mo-P-Y	Mo-P-Yb
Mo-P-Zn	Mo-P-Zr	Mo-Pd-Pt	Mo-Pd-Ru	Mo-Pd-Sb	Mo-Pd-Sc	Mo-Pd-Si	Mo-Pd-Sm
Mo-Pd-Sn	Mo-Pd-Ta	Mo-Pd-Tb	Mo-Pd-Ti	Mo-Pd-Y	Mo-Pd-Yb	Mo-Pd-Zn	Mo-Pd-Zr
Mo-Pt-Ru	Mo-Pt-Sb	Mo-Pt-Sc	Mo-Pt-Si	Mo-Pt-Sm	Mo-Pt-Sn	Mo-Pt-Ta	Mo-Pt-Tb
Mo-Pt-Ti	Mo-Pt-Y	Mo-Pt-Yb	Mo-Pt-Zn	Mo-Pt-Zr	Mo-Ru-Sb	Mo-Ru-Sc	Mo-Ru-Si
Mo-Ru-Sm	Mo-Ru-Sn	Mo-Ru-Ta	Mo-Ru-Tb	Mo-Ru-Ti	Mo-Ru-Y	Mo-Ru-Yb	Mo-Ru-Zn
Mo-Ru-Zr	Mo-Sb-Sc	Mo-Sb-Si	Mo-Sb-Sm	Mo-Sb-Sn	Mo-Sb-Ta	Mo-Sb-Tb	Mo-Sb-Ti
Mo-Sb-Y	Mo-Sb-Yb	Mo-Sb-Zn	Mo-Sb-Zr	Mo-Sc-Si	Mo-Sc-Sm	Mo-Sc-Sn	Mo-Sc-Ta
Mo-Sc-Tb	Mo-Sc-Ti	Mo-Sc-Y	Mo-Sc-Yb	Mo-Sc-Zn	Mo-Sc-Zr	Mo-Si-Sm	Mo-Si-Sn
Mo-Si-Ta	Mo-Si-Tb	Mo-Si-Ti	Mo-Si-Y	Mo-Si-Yb	Mo-Si-Zn	Mo-Si-Zr	Mo-Sm-Sn
Mo-Sm-Ta	Mo-Sm-Tb	Mo-Sm-Ti	Mo-Sm-Y	Mo-Sm-Yb	Mo-Sm-Zn	Mo-Sm-Zr	Mo-Sn-Ta
Mo-Sn-Tb	Mo-Sn-Ti	Mo-Sn-Y	Mo-Sn-Yb	Mo-Sn-Zn	Mo-Sn-Zr	Mo-Ta-Tb	Mo-Ta-Ti
Mo-Ta-Y	Mo-Ta-Yb	Mo-Ta-Zn	Mo-Ta-Zr	Mo-Tb-Ti	Mo-Tb-Y	Mo-Tb-Yb	Mo-Tb-Zn

Mo-Tb-Zr	Mo-Ti-Y	Mo-Ti-Yb	Mo-Ti-Zn	Mo-Ti-Zr	Mo-Y-Yb	Mo-Y-Zn	Mo-Y-Zr
Mo-Yb-Zn	Mo-Yb-Zr	Mo-Zn-Zr	Nb-Nd-Ni	Nb-Nd-P	Nb-Nd-Pd	Nb-Nd-Pt	Nb-Nd-Ru
Nb-Nd-Sb	Nb-Nd-Sc	Nb-Nd-Si	Nb-Nd-Sm	Nb-Nd-Sn	Nb-Nd-Ta	Nb-Nd-Tb	Nb-Nd-Ti
Nb-Nd-Y	Nb-Nd-Yb	Nb-Nd-Zn	Nb-Nd-Zr	Nb-Ni-P	Nb-Ni-Pd	Nb-Ni-Pt	Nb-Ni-Ru
Nb-Ni-Sb	Nb-Ni-Sc	Nb-Ni-Sm	Nb-Ni-Sn	Nb-Ni-Ta	Nb-Ni-Tb	Nb-Ni-Ti	Nb-Ni-Y
Nb-Ni-Yb	Nb-Ni-Zn	Nb-P-Pd	Nb-P-Pt	Nb-P-Ru	Nb-P-Sb	Nb-P-Sc	Nb-P-Si
Nb-P-Sm	Nb-P-Sn	Nb-P-Ta	Nb-P-Tb	Nb-P-Ti	Nb-P-Y	Nb-P-Yb	Nb-P-Zn
Nb-P-Zr	Nb-Pd-Pt	Nb-Pd-Ru	Nb-Pd-Sb	Nb-Pd-Sc	Nb-Pd-Si	Nb-Pd-Sm	Nb-Pd-Sn
Nb-Pd-Ta	Nb-Pd-Tb	Nb-Pd-Ti	Nb-Pd-Y	Nb-Pd-Yb	Nb-Pd-Zn	Nb-Pd-Zr	Nb-Pt-Ru
Nb-Pt-Sb	Nb-Pt-Sc	Nb-Pt-Si	Nb-Pt-Sm	Nb-Pt-Sn	Nb-Pt-Ta	Nb-Pt-Tb	Nb-Pt-Ti
Nb-Pt-Y	Nb-Pt-Yb	Nb-Pt-Zn	Nb-Pt-Zr	Nb-Ru-Sb	Nb-Ru-Sc	Nb-Ru-Si	Nb-Ru-Sm
Nb-Ru-Sn	Nb-Ru-Ta	Nb-Ru-Tb	Nb-Ru-Ti	Nb-Ru-Y	Nb-Ru-Yb	Nb-Ru-Zn	Nb-Ru-Zr
Nb-Sb-Sc	Nb-Sb-Si	Nb-Sb-Sm	Nb-Sb-Sn	Nb-Sb-Ta	Nb-Sb-Tb	Nb-Sb-Ti	Nb-Sb-Y
Nb-Sb-Yb	Nb-Sb-Zn	Nb-Sb-Zr	Nb-Sc-Si	Nb-Sc-Sm	Nb-Sc-Sn	Nb-Sc-Ta	Nb-Sc-Tb
Nb-Sc-Ti	Nb-Sc-Y	Nb-Sc-Yb	Nb-Sc-Zn	Nb-Sc-Zr	Nb-Si-Sm	Nb-Si-Sn	Nb-Si-Ta
Nb-Si-Tb	Nb-Si-Ti	Nb-Si-Y	Nb-Si-Yb	Nb-Si-Zn	Nb-Sm-Sn	Nb-Sm-Ta	Nb-Sm-Tb
Nb-Sm-Ti	Nb-Sm-Y	Nb-Sm-Yb	Nb-Sm-Zn	Nb-Sm-Zr	Nb-Sn-Ta	Nb-Sn-Tb	Nb-Sn-Ti
Nb-Sn-Y	Nb-Sn-Yb	Nb-Sn-Zn	Nb-Sn-Zr	Nb-Ta-Tb	Nb-Ta-Ti	Nb-Ta-Y	Nb-Ta-Yb
Nb-Ta-Zn	Nb-Ta-Zr	Nb-Tb-Ti	Nb-Tb-Y	Nb-Tb-Yb	Nb-Tb-Zn	Nb-Tb-Zr	Nb-Ti-Y
Nb-Ti-Yb	Nb-Ti-Zn	Nb-Ti-Zr	Nb-Y-Yb	Nb-Y-Zn	Nb-Y-Zr	Nb-Yb-Zn	Nb-Yb-Zr
Nb-Zn-Zr	Nd-Ni-P	Nd-Ni-Pd	Nd-Ni-Pt	Nd-Ni-Ru	Nd-Ni-Sb	Nd-Ni-Sc	Nd-Ni-Si
Nd-Ni-Sm	Nd-Ni-Sn	Nd-Ni-Ta	Nd-Ni-Tb	Nd-Ni-Ti	Nd-Ni-Y	Nd-Ni-Yb	Nd-Ni-Zn
Nd-Ni-Zr	Nd-P-Pd	Nd-P-Pt	Nd-P-Ru	Nd-P-Sb	Nd-P-Sc	Nd-P-Si	Nd-P-Sm
Nd-P-Sn	Nd-P-Ta	Nd-P-Tb	Nd-P-Ti	Nd-P-Y	Nd-P-Yb	Nd-P-Zn	Nd-P-Zr
Nd-Pd-Pt	Nd-Pd-Ru	Nd-Pd-Sb	Nd-Pd-Sc	Nd-Pd-Si	Nd-Pd-Sm	Nd-Pd-Sn	Nd-Pd-Ta

Nd-Pd-Tb	Nd-Pd-Ti	Nd-Pd-Y	Nd-Pd-Yb	Nd-Pd-Zn	Nd-Pd-Zr	Nd-Pt-Ru	Nd-Pt-Sb
Nd-Pt-Sc	Nd-Pt-Si	Nd-Pt-Sm	Nd-Pt-Sn	Nd-Pt-Ta	Nd-Pt-Tb	Nd-Pt-Ti	Nd-Pt-Y
Nd-Pt-Yb	Nd-Pt-Zn	Nd-Pt-Zr	Nd-Ru-Sb	Nd-Ru-Sc	Nd-Ru-Si	Nd-Ru-Sm	Nd-Ru-Sn
Nd-Ru-Ta	Nd-Ru-Tb	Nd-Ru-Ti	Nd-Ru-Y	Nd-Ru-Yb	Nd-Ru-Zn	Nd-Ru-Zr	Nd-Sb-Sc
Nd-Sb-Si	Nd-Sb-Sm	Nd-Sb-Sn	Nd-Sb-Ta	Nd-Sb-Tb	Nd-Sb-Ti	Nd-Sb-Y	Nd-Sb-Yb
Nd-Sb-Zn	Nd-Sb-Zr	Nd-Sc-Si	Nd-Sc-Sm	Nd-Sc-Sn	Nd-Sc-Ta	Nd-Sc-Tb	Nd-Sc-Ti
Nd-Sc-Y	Nd-Sc-Yb	Nd-Sc-Zn	Nd-Sc-Zr	Nd-Si-Sm	Nd-Si-Sn	Nd-Si-Ta	Nd-Si-Tb
Nd-Si-Ti	Nd-Si-Y	Nd-Si-Yb	Nd-Si-Zn	Nd-Si-Zr	Nd-Sm-Sn	Nd-Sm-Ta	Nd-Sm-Tb
Nd-Sm-Ti	Nd-Sm-Y	Nd-Sm-Yb	Nd-Sm-Zn	Nd-Sm-Zr	Nd-Sn-Ta	Nd-Sn-Tb	Nd-Sn-Ti
Nd-Sn-Y	Nd-Sn-Yb	Nd-Sn-Zn	Nd-Sn-Zr	Nd-Ta-Tb	Nd-Ta-Ti	Nd-Ta-Y	Nd-Ta-Yb
Nd-Ta-Zn	Nd-Ta-Zr	Nd-Tb-Ti	Nd-Tb-Y	Nd-Tb-Yb	Nd-Tb-Zn	Nd-Tb-Zr	Nd-Ti-Y
Nd-Ti-Yb	Nd-Ti-Zn	Nd-Ti-Zr	Nd-Y-Yb	Nd-Y-Zn	Nd-Y-Zr	Nd-Yb-Zn	Nd-Yb-Zr
Nd-Zn-Zr	Ni-P-Pt	Ni-P-Ru	Ni-P-Sb	Ni-P-Sc	Ni-P-Si	Ni-P-Sm	Ni-P-Sn
Ni-P-Ta	Ni-P-Tb	Ni-P-Ti	Ni-P-Y	Ni-P-Yb	Ni-P-Zn	Ni-P-Zr	Ni-Pd-Pt
Ni-Pd-Ru	Ni-Pd-Sb	Ni-Pd-Sc	Ni-Pd-Sm	Ni-Pd-Sn	Ni-Pd-Ta	Ni-Pd-Tb	Ni-Pd-Ti
Ni-Pd-Y	Ni-Pd-Yb	Ni-Pd-Zn	Ni-Pt-Ru	Ni-Pt-Sb	Ni-Pt-Sc	Ni-Pt-Si	Ni-Pt-Sm
Ni-Pt-Sn	Ni-Pt-Ta	Ni-Pt-Tb	Ni-Pt-Ti	Ni-Pt-Y	Ni-Pt-Yb	Ni-Pt-Zn	Ni-Pt-Zr
Ni-Ru-Sb	Ni-Ru-Sc	Ni-Ru-Si	Ni-Ru-Sm	Ni-Ru-Sn	Ni-Ru-Ta	Ni-Ru-Tb	Ni-Ru-Ti
Ni-Ru-Y	Ni-Ru-Yb	Ni-Ru-Zn	Ni-Ru-Zr	Ni-Sb-Sc	Ni-Sb-Si	Ni-Sb-Sm	Ni-Sb-Sn
Ni-Sb-Ta	Ni-Sb-Tb	Ni-Sb-Ti	Ni-Sb-Y	Ni-Sb-Yb	Ni-Sb-Zn	Ni-Sb-Zr	Ni-Sc-Si
Ni-Sc-Sm	Ni-Sc-Sn	Ni-Sc-Ta	Ni-Sc-Tb	Ni-Sc-Ti	Ni-Sc-Y	Ni-Sc-Yb	Ni-Sc-Zn
Ni-Sc-Zr	Ni-Si-Sm	Ni-Si-Sn	Ni-Si-Ta	Ni-Si-Tb	Ni-Si-Y	Ni-Si-Yb	Ni-Si-Zn
Ni-Sm-Sn	Ni-Sm-Ta	Ni-Sm-Tb	Ni-Sm-Ti	Ni-Sm-Y	Ni-Sm-Yb	Ni-Sm-Zn	Ni-Sm-Zr
Ni-Sn-Ta	Ni-Sn-Tb	Ni-Sn-Ti	Ni-Sn-Y	Ni-Sn-Yb	Ni-Sn-Zn	Ni-Sn-Zr	Ni-Ta-Tb
Ni-Ta-Ti	Ni-Ta-Y	Ni-Ta-Yb	Ni-Ta-Zn	Ni-Ta-Zr	Ni-Tb-Ti	Ni-Tb-Y	Ni-Tb-Yb

Ni-Tb-Zn	Ni-Tb-Zr	Ni-Ti-Y	Ni-Ti-Yb	Ni-Ti-Zn	Ni-Y-Yb	Ni-Y-Zn	Ni-Y-Zr
Ni-Yb-Zn	Ni-Yb-Zr	Ni-Zn-Zr	P-Pd-Pt	P-Pd-Ru	P-Pd-Sb	P-Pd-Sc	P-Pd-Si
P-Pd-Sm	P-Pd-Sn	P-Pd-Ta	P-Pd-Tb	P-Pd-Ti	P-Pd-Y	P-Pd-Yb	P-Pd-Zn
P-Pd-Zr	P-Pt-Ru	P-Pt-Sb	P-Pt-Sc	P-Pt-Si	P-Pt-Sm	P-Pt-Sn	P-Pt-Ta
P-Pt-Tb	P-Pt-Ti	P-Pt-Y	P-Pt-Yb	P-Pt-Zn	P-Pt-Zr	P-Ru-Sb	P-Ru-Sc
P-Ru-Si	P-Ru-Sm	P-Ru-Sn	P-Ru-Ta	P-Ru-Tb	P-Ru-Ti	P-Ru-Y	P-Ru-Yb
P-Ru-Zn	P-Ru-Zr	P-Sb-Sc	P-Sb-Si	P-Sb-Sm	P-Sb-Sn	P-Sb-Ta	P-Sb-Tb
P-Sb-Ti	P-Sb-Y	P-Sb-Yb	P-Sb-Zn	P-Sb-Zr	P-Sc-Si	P-Sc-Sm	P-Sc-Sn
P-Sc-Ta	P-Sc-Tb	P-Sc-Ti	P-Sc-Y	P-Sc-Yb	P-Sc-Zn	P-Sc-Zr	P-Si-Sm
P-Si-Sn	P-Si-Ta	P-Si-Tb	P-Si-Ti	P-Si-Y	P-Si-Yb	P-Si-Zn	P-Si-Zr
P-Sm-Sn	P-Sm-Ta	P-Sm-Tb	P-Sm-Ti	P-Sm-Y	P-Sm-Yb	P-Sm-Zn	P-Sm-Zr
P-Sn-Ta	P-Sn-Tb	P-Sn-Ti	P-Sn-Y	P-Sn-Yb	P-Sn-Zn	P-Sn-Zr	P-Ta-Tb
P-Ta-Ti	P-Ta-Y	P-Ta-Yb	P-Ta-Zn	P-Ta-Zr	P-Tb-Ti	P-Tb-Y	P-Tb-Yb
P-Tb-Zn	P-Tb-Zr	P-Ti-Y	P-Ti-Yb	P-Ti-Zn	P-Ti-Zr	P-Y-Yb	P-Y-Zn
P-Y-Zr	P-Yb-Zn	P-Yb-Zr	P-Zn-Zr	Pd-Pt-Ru	Pd-Pt-Sb	Pd-Pt-Sc	Pd-Pt-Si
Pd-Pt-Sm	Pd-Pt-Sn	Pd-Pt-Ta	Pd-Pt-Tb	Pd-Pt-Ti	Pd-Pt-Y	Pd-Pt-Yb	Pd-Pt-Zn
Pd-Pt-Zr	Pd-Ru-Sb	Pd-Ru-Sc	Pd-Ru-Si	Pd-Ru-Sm	Pd-Ru-Sn	Pd-Ru-Ta	Pd-Ru-Tb
Pd-Ru-Ti	Pd-Ru-Y	Pd-Ru-Yb	Pd-Ru-Zn	Pd-Ru-Zr	Pd-Sb-Sc	Pd-Sb-Si	Pd-Sb-Sm
Pd-Sb-Sn	Pd-Sb-Ta	Pd-Sb-Tb	Pd-Sb-Ti	Pd-Sb-Y	Pd-Sb-Yb	Pd-Sb-Zn	Pd-Sb-Zr
Pd-Sc-Si	Pd-Sc-Sm	Pd-Sc-Sn	Pd-Sc-Ta	Pd-Sc-Tb	Pd-Sc-Ti	Pd-Sc-Y	Pd-Sc-Yb
Pd-Sc-Zn	Pd-Sc-Zr	Pd-Si-Sm	Pd-Si-Sn	Pd-Si-Ta	Pd-Si-Tb	Pd-Si-Ti	Pd-Si-Y
Pd-Si-Yb	Pd-Si-Zn	Pd-Sm-Sn	Pd-Sm-Ta	Pd-Sm-Tb	Pd-Sm-Ti	Pd-Sm-Y	Pd-Sm-Yb
Pd-Sm-Zn	Pd-Sm-Zr	Pd-Sn-Ta	Pd-Sn-Tb	Pd-Sn-Ti	Pd-Sn-Y	Pd-Sn-Yb	Pd-Sn-Zn
Pd-Sn-Zr	Pd-Ta-Tb	Pd-Ta-Ti	Pd-Ta-Y	Pd-Ta-Yb	Pd-Ta-Zn	Pd-Ta-Zr	Pd-Tb-Ti
Pd-Tb-Y	Pd-Tb-Yb	Pd-Tb-Zn	Pd-Tb-Zr	Pd-Ti-Y	Pd-Ti-Yb	Pd-Ti-Zn	Pd-Ti-Zr

Pd-Y-Yb	Pd-Y-Zn	Pd-Y-Zr	Pd-Yb-Zn	Pd-Yb-Zr	Pd-Zn-Zr	Pt-Ru-Sb	Pt-Ru-Sc
Pt-Ru-Si	Pt-Ru-Sm	Pt-Ru-Sn	Pt-Ru-Ta	Pt-Ru-Tb	Pt-Ru-Ti	Pt-Ru-Y	Pt-Ru-Yb
Pt-Ru-Zn	Pt-Ru-Zr	Pt-Sb-Sc	Pt-Sb-Si	Pt-Sb-Sm	Pt-Sb-Sn	Pt-Sb-Ta	Pt-Sb-Tb
Pt-Sb-Ti	Pt-Sb-Y	Pt-Sb-Yb	Pt-Sb-Zn	Pt-Sb-Zr	Pt-Sc-Si	Pt-Sc-Sm	Pt-Sc-Sn
Pt-Sc-Ta	Pt-Sc-Tb	Pt-Sc-Ti	Pt-Sc-Y	Pt-Sc-Yb	Pt-Sc-Zn	Pt-Sc-Zr	Pt-Si-Sm
Pt-Si-Sn	Pt-Si-Ta	Pt-Si-Tb	Pt-Si-Y	Pt-Si-Yb	Pt-Si-Zn	Pt-Sm-Sn	Pt-Sm-Ta
Pt-Sm-Tb	Pt-Sm-Ti	Pt-Sm-Y	Pt-Sm-Yb	Pt-Sm-Zn	Pt-Sm-Zr	Pt-Sn-Ta	Pt-Sn-Tb
Pt-Sn-Ti	Pt-Sn-Y	Pt-Sn-Yb	Pt-Sn-Zn	Pt-Sn-Zr	Pt-Ta-Tb	Pt-Ta-Ti	Pt-Ta-Y
Pt-Ta-Yb	Pt-Ta-Zn	Pt-Ta-Zr	Pt-Tb-Ti	Pt-Tb-Y	Pt-Tb-Yb	Pt-Tb-Zn	Pt-Tb-Zr
Pt-Ti-Y	Pt-Ti-Yb	Pt-Ti-Zn	Pt-Y-Yb	Pt-Y-Zn	Pt-Y-Zr	Pt-Yb-Zn	Pt-Yb-Zr
Pt-Zn-Zr	Ru-Sb-Sc	Ru-Sb-Si	Ru-Sb-Sm	Ru-Sb-Sn	Ru-Sb-Ta	Ru-Sb-Tb	Ru-Sb-Ti
Ru-Sb-Y	Ru-Sb-Yb	Ru-Sb-Zn	Ru-Sb-Zr	Ru-Sc-Si	Ru-Sc-Sm	Ru-Sc-Sn	Ru-Sc-Ta
Ru-Sc-Tb	Ru-Sc-Ti	Ru-Sc-Y	Ru-Sc-Yb	Ru-Sc-Zn	Ru-Sc-Zr	Ru-Si-Sm	Ru-Si-Sn
Ru-Si-Ta	Ru-Si-Tb	Ru-Si-Ti	Ru-Si-Y	Ru-Si-Yb	Ru-Si-Zn	Ru-Si-Zr	Ru-Sm-Sn
Ru-Sm-Ta	Ru-Sm-Tb	Ru-Sm-Ti	Ru-Sm-Y	Ru-Sm-Yb	Ru-Sm-Zn	Ru-Sm-Zr	Ru-Sn-Ta
Ru-Sn-Tb	Ru-Sn-Ti	Ru-Sn-Y	Ru-Sn-Yb	Ru-Sn-Zn	Ru-Sn-Zr	Ru-Ta-Tb	Ru-Ta-Ti
Ru-Ta-Y	Ru-Ta-Yb	Ru-Ta-Zn	Ru-Ta-Zr	Ru-Tb-Ti	Ru-Tb-Y	Ru-Tb-Yb	Ru-Tb-Zn
Ru-Tb-Zr	Ru-Ti-Y	Ru-Ti-Yb	Ru-Ti-Zn	Ru-Ti-Zr	Ru-Y-Yb	Ru-Y-Zn	Ru-Y-Zr
Ru-Yb-Zn	Ru-Yb-Zr	Ru-Zn-Zr	Sb-Sc-Si	Sb-Sc-Sm	Sb-Sc-Sn	Sb-Sc-Ta	Sb-Sc-Tb
Sb-Sc-Ti	Sb-Sc-Y	Sb-Sc-Yb	Sb-Sc-Zn	Sb-Sc-Zr	Sb-Si-Sm	Sb-Si-Sn	Sb-Si-Ta
Sb-Si-Tb	Sb-Si-Ti	Sb-Si-Y	Sb-Si-Yb	Sb-Si-Zn	Sb-Si-Zr	Sb-Sm-Sn	Sb-Sm-Ta
Sb-Sm-Tb	Sb-Sm-Ti	Sb-Sm-Y	Sb-Sm-Yb	Sb-Sm-Zn	Sb-Sm-Zr	Sb-Sn-Ta	Sb-Sn-Tb
Sb-Sn-Ti	Sb-Sn-Y	Sb-Sn-Yb	Sb-Sn-Zn	Sb-Sn-Zr	Sb-Ta-Tb	Sb-Ta-Ti	Sb-Ta-Y
Sb-Ta-Yb	Sb-Ta-Zn	Sb-Ta-Zr	Sb-Tb-Ti	Sb-Tb-Y	Sb-Tb-Yb	Sb-Tb-Zn	Sb-Tb-Zr
Sb-Ti-Y	Sb-Ti-Yb	Sb-Ti-Zn	Sb-Ti-Zr	Sb-Y-Yb	Sb-Y-Zn	Sb-Y-Zr	Sb-Yb-Zn

Sb-Yb-Zr	Sb-Zn-Zr	Sc-Si-Sm	Sc-Si-Sn	Sc-Si-Ta	Sc-Si-Tb	Sc-Si-Ti	Sc-Si-Y
Sc-Si-Yb	Sc-Si-Zn	Sc-Si-Zr	Sc-Sm-Sn	Sc-Sm-Ta	Sc-Sm-Tb	Sc-Sm-Ti	Sc-Sm-Y
Sc-Sm-Yb	Sc-Sm-Zn	Sc-Sm-Zr	Sc-Sn-Ta	Sc-Sn-Tb	Sc-Sn-Ti	Sc-Sn-Y	Sc-Sn-Yb
Sc-Sn-Zn	Sc-Sn-Zr	Sc-Ta-Tb	Sc-Ta-Ti	Sc-Ta-Y	Sc-Ta-Yb	Sc-Ta-Zn	Sc-Ta-Zr
Sc-Tb-Ti	Sc-Tb-Y	Sc-Tb-Yb	Sc-Tb-Zn	Sc-Tb-Zr	Sc-Ti-Y	Sc-Ti-Yb	Sc-Ti-Zn
Sc-Ti-Zr	Sc-Y-Yb	Sc-Y-Zn	Sc-Y-Zr	Sc-Yb-Zn	Sc-Yb-Zr	Sc-Zn-Zr	Si-Sm-Sn
Si-Sm-Ta	Si-Sm-Tb	Si-Sm-Ti	Si-Sm-Y	Si-Sm-Yb	Si-Sm-Zn	Si-Sm-Zr	Si-Sn-Ta
Si-Sn-Tb	Si-Sn-Ti	Si-Sn-Y	Si-Sn-Yb	Si-Sn-Zn	Si-Sn-Zr	Si-Ta-Tb	Si-Ta-Ti
Si-Ta-Y	Si-Ta-Yb	Si-Ta-Zn	Si-Ta-Zr	Si-Tb-Ti	Si-Tb-Y	Si-Tb-Yb	Si-Tb-Zn
Si-Tb-Zr	Si-Ti-Y	Si-Ti-Yb	Si-Ti-Zn	Si-Y-Yb	Si-Y-Zn	Si-Y-Zr	Si-Yb-Zn
Si-Yb-Zr	Si-Zn-Zr	Sm-Sn-Ta	Sm-Sn-Tb	Sm-Sn-Ti	Sm-Sn-Y	Sm-Sn-Yb	Sm-Sn-Zn
Sm-Sn-Zr	Sm-Ta-Tb	Sm-Ta-Ti	Sm-Ta-Y	Sm-Ta-Yb	Sm-Ta-Zn	Sm-Ta-Zr	Sm-Tb-Ti
Sm-Tb-Y	Sm-Tb-Yb	Sm-Tb-Zn	Sm-Tb-Zr	Sm-Ti-Y	Sm-Ti-Yb	Sm-Ti-Zn	Sm-Ti-Zr
Sm-Y-Yb	Sm-Y-Zn	Sm-Y-Zr	Sm-Yb-Zn	Sm-Yb-Zr	Sm-Zn-Zr	Sn-Ta-Tb	Sn-Ta-Ti
Sn-Ta-Y	Sn-Ta-Yb	Sn-Ta-Zn	Sn-Ta-Zr	Sn-Tb-Ti	Sn-Tb-Y	Sn-Tb-Yb	Sn-Tb-Zn
Sn-Tb-Zr	Sn-Ti-Y	Sn-Ti-Yb	Sn-Ti-Zn	Sn-Ti-Zr	Sn-Y-Yb	Sn-Y-Zn	Sn-Y-Zr
Sn-Yb-Zn	Sn-Yb-Zr	Sn-Zn-Zr	Ta-Tb-Ti	Ta-Tb-Y	Ta-Tb-Yb	Ta-Tb-Zn	Ta-Tb-Zr
Ta-Ti-Y	Ta-Ti-Yb	Ta-Ti-Zn	Ta-Ti-Zr	Ta-Y-Yb	Ta-Y-Zn	Ta-Y-Zr	Ta-Yb-Zn
Ta-Yb-Zr	Ta-Zn-Zr	Tb-Ti-Y	Tb-Ti-Yb	Tb-Ti-Zn	Tb-Ti-Zr	Tb-Y-Yb	Tb-Y-Zn
Tb-Y-Zr	Tb-Yb-Zn	Tb-Yb-Zr	Tb-Zn-Zr	Ti-Y-Yb	Ti-Y-Zn	Ti-Y-Zr	Ti-Yb-Zn
Ti-Yb-Zr	Ti-Zn-Zr	Y-Yb-Zn	Y-Yb-Zr	Y-Zn-Zr	Yb-Zn-Zr		
